# A Series of New Pyrrole Alkaloids with ALR2 Inhibitory Activities from the Sponge *Stylissa massa*

**DOI:** 10.3390/md20070454

**Published:** 2022-07-12

**Authors:** Qi Wang, Chunhua Gao, Zhun Wei, Xiaowen Tang, Lixia Ji, Xiangchao Luo, Xiaoping Peng, Gang Li, Hongxiang Lou

**Affiliations:** 1 Department of Natural Medicinal Chemistry and Pharmacognosy, School of Pharmacy, Qingdao University, Qingdao 266021, China; wangqi@hmfl.ac.cn (Q.W.); gaochunhua@qdu.edu.cn (C.G.); biowei@qdu.edu.cn (Z.W.); xwtang1219@qdu.edu.cn (X.T.); lixiaji@qdu.edu.cn (L.J.); pengxiaoping@qdu.edu.cn (X.P.); gang.li@qdu.edu.cn (G.L.); 2 Research Center for Marine Drugs, Department of Pharmacy, Ren Ji Hospital, School of Medicine, Shanghai Jiao Tong University, Shanghai, 200127, China; luoxiangchao@renji.com

**Keywords:** pyrrole-imidazole alkaloids, ALR2 inhibitory activities, sponge, *Stylissa massa*, spongiacidin

## Abstract

Twelve new and four known alkaloids including five different structural scaffolds were isolated from the sponge *Stylissa massa* collected in the South China Sea. Compound **1** is the first identified precursor metabolite of the classic 5/7/5 tricyclic skeleton with unesterified guanidine and carboxyl groups, compounds **2**–**5** and **13**–**15** belong to the spongiacidin-type pyrrole imidazole alkaloids (PIAs). *Z*- and *E*-configurations of the spongiacidin-type PIAs often appeared concomitantly and were distinguished by the chemical shift analysis of ^13^C NMR spectra. The structures of all twelve new compounds were determined by NMR, MS, and ECD analysis combined with single-crystal data of compounds **1**, **5**, and **10**. In the aldose reductase (ALR2) inhibitory assay, six 5/7/5 tricyclic compounds (**2**–**5**, **13**–**15**) displayed significant activities. Compounds **13** and **14**, as the representative members of spongiacidin-PIAs, demonstrated their ALR2-targeted activities in SPR experiments with K_D_ values of 12.5 and 6.9 µM, respectively.

## 1. Introduction

Pyrrole-imidazole alkaloids (PIAs) and simple pyrrole alkaloids represent a specific structural class of compounds isolated from sponges including those from the genus *Agelas*, *Axinella*, *Hymeniacidon*, *Phakellia* and *Stylissa* [1,2,3,4,5]. PIAs can be divided into monomeric and polymeric groups. Like sceptrin^1^, palau’amine [6], ageliferin [7], and stylissadine [8] represent members of polymeric PIAs. Biosynthesis of mono-PIAs originates from proline and lysine [9], evolving to form several skeletons such as oroidin [10], phakellin [11], ugibohlin [12], and spongiacidin types [13], which have 5/5 bicyclic, 5/6/5/5 tetracyclic, 5/6/5 tricyclic, and 5/7/5 tricyclic systems, respectively. Currently, although hundreds of PIAs have been discovered from sponges, the structural diversity of this alkaloid family, especially for the monomeric ones, is relatively conservative.

We collected the *Stylissa massa* sponge from the Xisha Islands (Paracel island) in the South China Sea. Targeted isolation for methanol extraction yielded twelve new and four known compounds. Five biosynthetic-related PIA skeletons including the 5/7 imidazole-acyclic compound **1**, 5/7/5 spongiacidins (**2**–**5**, and **13**–**15**), 5/6/5/5 phakellins (**6** and **16**), 5/7 bicyclic (**7**–**9**), and pyrrole single ring alkaloids (**10**–**12**) were obtained from *Stylissa massa*. Compound **1** is the first identified precursor metabolite of the classic 5/7/5 tricyclic skeleton with unesterified guanidine and carboxyl groups. Compounds **2a** and **2b** are a scalemic mixture with a hydroxyl group positioned at C-9. Compounds **3a** and **3b** are also scalemic mixture compounds with the *E*-configuration of ∆^10,11^ versus the *Z*-configuration of **2**. Compound **4** has a hydroxide at C-9 with a double bond ∆^9,10^ at a different position, and the new compound **5** has an extra methyl group at 13-NH.

**Figure 1:**
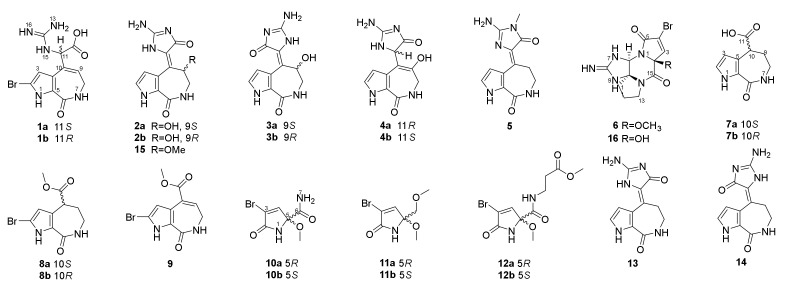
The structures of compounds **1**–**16**.

Guanidine compounds often exhibit effective diabetes related activities [14]. ALR2 is a key limiting enzyme of the glucose polyol metabolic pathway, which is the key target for the treatment of diabetes complications [15]. In the ALR2 enzyme activity assay in vitro, spongiacidin-type PIAs (compounds **2**–**5**, and **13**–**15**) presented superior inhibitory activities than the other skeletons at 20 µM. The IC_50_ values ranged from 8.6 to 13.6 µM, respectively (Figure S23). The SPR experiments verified the interaction between ALR2 and compound **14**, with the K_D_ value of 6.9 µM. In summary, spongiacidin-PIAs are efficient ALR2-targeted inhibitors with 5/7/5 tricyclic skeletons. Thus, the structural elucidation and the ALR2 inhibitory activities of these PIAs are concluded below (Figure 1).

## 2. Results

**Figure 2:**
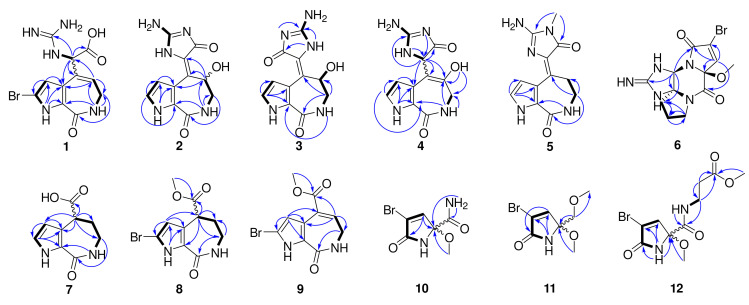
The key COSY (bolds), and HMBC (arrows) correlations of **1**–**12**.

**Figure 3:**
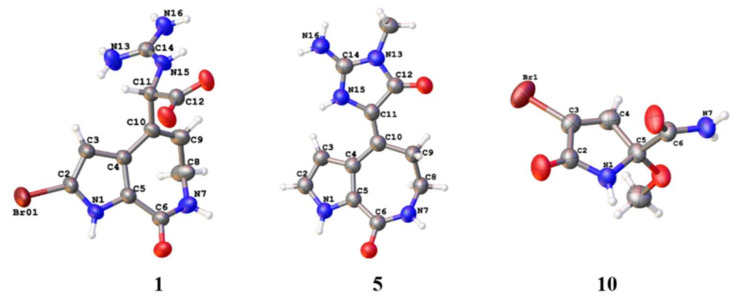
The X-ray structures of compounds **1**, **5**, and **10**.

Compound **1** was obtained as a colorless bulk crystal. Its molecular formula C_11_H_12_BrN_5_O_3_ was determined by HRESIMS with the ion peak at *m*/*z* 342.0197, and 344.0170 with the proportion of 1:1 (calcd. for [M + H]^+^ *m*/*z* 342.0196, and 344.0176), with the unsaturation degree of 8. The ^13^C NMR and DEPT spectra revealed 11 resonances (Table S1) including one methylene, three methines, and seven non-protonated carbons. The chemical shifts ranging from *δ*_C_ 104.3 to 133.6 ppm showed six olefinic carbons, which included two methines and four non-protonated carbons. In the low field of the ^13^C NMR spectrum with the chemical shifts of *δ*_C_ 170.5, 162.1, and 156.4, there could be two carbonyl groups and one guanidyl group that exist in this molecule. The ^1^H NMR and HSQC spectra (Table S2) showed the presence of two olefinic protons at *δ*_H_ 6.36 (1H, d, *J* = 2.3 Hz), and *δ*_H_ 6.04 (1H, t, *J* = 6.8 Hz), one methine proton at *δ*_H_ 5.21 (1H, d, *J* = 8.5 Hz), and one methylene proton at *δ*_H_ 3.42 (2H, m), and there were three additional heteroatomic protons at *δ*_H_ 12.71 (brs), *δ*_H_ 7.80 (1H, d, *J* = 8.3 Hz), and *δ*_H_ 7.79 (1H, q, *J* = 4.7 Hz). The ^1^H-^1^H COSY spectrum (Figure 2) revealed a pyrrole conjugate ring system and another spin system with the correlations between 1-NH and H-3, between 15-NH and H-11, between 7-NH and H_2_-8, and between H_2_-8 and H-9. Key HMBC correlations (Figure 2) of 1-NH/C-3, C-4, and C-5, H-3/C-2, and C-4, 7-NH/C-5, and C-9, H_2_-8/C-6, C-9, and C-10, H-9/C-4, C-8, and C-10 determined the existence of a 5/7 bicyclic 2-bromo-6,7-dihydropyrrolo [2,3-*c*] azepin-8(1*H*)-one skeleton (Figure 2). The already existential bicyclic skeleton and four double bonds occupied six degrees of unsaturation, and the remaining two degrees pointed out that there was no other ring systems in compound **1**. The final planar structure of compound **1** was settled down by the HMBC correlations from H-11 to C-4, C-9, C-10, C-12, and C-14, together with the correlations from H-9 to C-11 as well as 15-NH to C-12. A suitable bulk single crystal of compound **1** was obtained to perform the X-ray diffraction experiment, which ensured the planar structure of **1** (Figure 3). The space group of **1** indicated that it was a scalemic mixture, and ECD calculation finally determined the absolute configurations of **1a** and **1b** isolated through chiral HPLC by comparison with each of their ECD spectra (Figures S1 and S2).

Compound **2** was obtained as a yellow oil and its molecular formula was determined as C_11_H_11_N_5_O_3_ by the [M + H]^+^ ion peak presented at *m*/*z* 262.0936 (calcd. C_11_H_12_N_5_O_3_ for *m*/*z* 262.0935) in the HRESIMS spectrum with nine unsaturation degrees. One more unsaturation degree and the similar chemical shifts of the carbons (Table S1) with **1** indicated that compound **2** may have a 5/7/5 tricyclic spongiacidin-PIA skeleton, close to the known compound **13**. The HMBC correlations (Figure 2) of 1-NH/C-3, and C-4, H-2/C-3, C-4, and C-5, H-3/C-2, C-4, and C-5, 7-NH/C-5, C-8, and C-9, H_2_-8/C-6, C-9, and C-10, and H-9/C-2, C-4, C-10, and C-11, together with the COSY correlations of 1-NH/H-2/H-3, and 7-NH/H_2_-8/H-9, constructed the classic 5/7/5 tricyclic structure with the hydroxyl group substituted at C-9, taking the chemical shift of C-9 (*δ*_C_ 62.8) into consideration. Thus, the planar structure of **2** was established.

**Figure 4:**
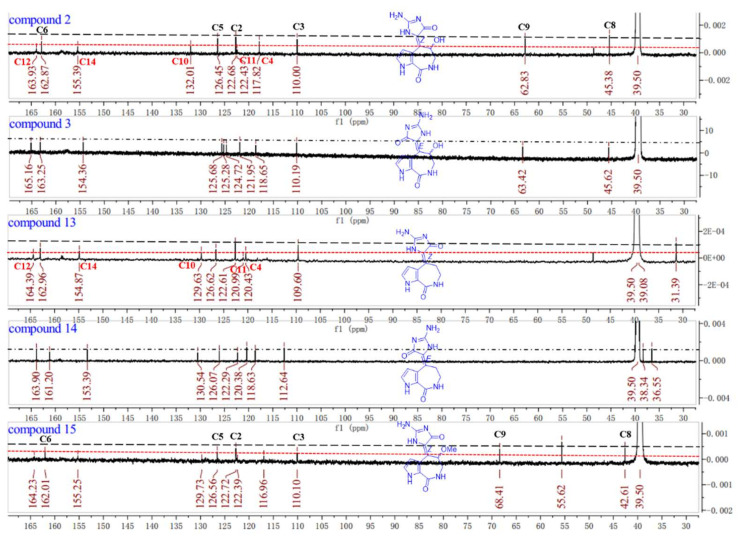
The comparison of the ^13^C NMR spectra of compounds **2**, **3**, **13**, **14**, and **15** (125 MHz, DMSO-*d*_6_).

The 1D and 2D NMR spectra as well as the HRESIMS spectrum indicated that compound **3** possessed the same planar structure with **2** (Figure 2). The *Z*- and *E*-configuration of double bond ∆^10,11^ in spongiacidin-type PIAs often concomitantly appeared and their differences can be attributed to the anisotropic effect of the carbonyl at C-12 [16]. Two configurations of known compounds **13** and **14** could be distinguished by the chemical shift values of H-3 and H_2_-9, but the existence of 9-OH substituted in compounds **2** and **3** made it so that the judgement rule did not work [compound 2: *δ*_H_ 6.45 (H-3), 5.80 (H-9); compound **3**: *δ*_H_ 6.43 (H-3), 5.83 (H-9)]. Through careful analysis of their ^13^C NMR spectra, we found the double bonds of compounds **2** and **13** could be in the *Z*-configuration [16] because the signals for C-4, C-10, C-11, C-12, and C-14 were weak compared with the stronger signals for C-2, C-3, C-5, C-6, C-8, and C-9, while the carbon signals of compounds **3** and **14** were distributed on average comparatively (Figure 4). Known compound **15** co-isolated was also determined to be of a *Z*-configuration with the evidence of its carbon signal analysis in the ^13^C NMR spectrum. Thus, the double bond of ∆^10,11^ in compound **2** was determined as the *Z*-configuration and compound **3** was the *E*-configuration. C-9′s absolute configurations of compounds **2a**, **2b** and **3a**, **3b** were all identified based on the ECD calculations together with the chiral HPLC method (Figures S3–S6).

Compound **4** is a molecule similar to compounds **2** and **3**, with the same molecular formula C_11_H_11_N_5_O_3_ by the [M + H]^+^ ion peak presented at *m*/*z* 262.0931 (calcd. for *m*/*z* C_11_H_12_N_5_O_3_ 262.0935) in the HRESIMS spectrum. The key ^1^H-^1^H COSY correlations between 7-NH and H_2_-8, and between H-11 and 15-NH uncovered the different structure of **4**, and its final planar structure was determined by the HMBC correlations from 1-NH to C-2, C-3, C-4, and C-5, from H-2 to C-3, C-4, and C-5, from H-3 to C-2, C-4, C-5, and C-10, from 7-NH to C-5, C-8, and C-9, from H-8 to C-6, C-9, and C-10, from H-11 to C-4, C-9, C-10, C-12, and C-14, from 15-NH to C-10, and from 9-OH to C-8, C-9, and C-10 (Figure 2). ECD calculation was also carried out to determine the absolute configurations of **4a** and **4b** (Figures S7 and S8).

Compound **5** was obtained as colorless bulk crystals. Its molecular formula was determined to be C_12_H_13_N_5_O_2_ by HRESIMS (*m*/*z* 260.1148, calcd. [M + H]^+^ for *m*/*z* 260.1142), which required nine degrees of unsaturation. The 1D and 2D NMR data revealed its similarity with the known compound **13**, with the only difference at 13-NMe with the extra signals of *δ*_H_ 3.11 and *δ*_C_ 25.8 in the ^1^H and ^13^C NMR spectra (Tables S1 and S2). The HMBC correlations from 13-NMe to C-12 and C-14 further confirmed the planar structure of **5** (Figure 2). Fortunately, a suitable bulk crystal was obtained followed by X-ray diffraction (Figure 3), and the result showed that the previously proposed rule of distinguishing *Z*/*E* configurations of double bond ∆^10,11^ in 5/7/5 spongiacidin-type PIAs was trustworthy.

The molecular formula of compound **6**, obtained as a yellow oil, was determined to be C_12_H_14_BrN_5_O_3_, according to the HRESIMS results, which showed a protonated molecular ion at *m*/*z* 356.0357, 358.0329 (calcd. for [M + H]^+^, *m*/*z* 356.0353, 358.0332). The analysis of the 1D and 2D NMR spectra (Tables S3 and S4) of **6** indicated that it had a very similar structure with the reported compound (**16**) obtained by organic synthesis [17], with the only difference of 2-OMe rather than 2-OH. The relative configuration of **6** was determined by DP4+ analysis, where the results showed that the only possibility was 2R*6R*10S*, and further ECD calculations confirmed the absolute configuration of **6** was 2*S*6*S*10*R* (Figures S9 and S10).

Compounds **7**–**9** were 5/7 bicyclic pyrrole alkaloids, in which compounds **8** and **9** were 2-bromo substituted ones. Their structures were confirmed by HRESIMS and NMR data (Tables S3 and S4). Compounds **7** and **8** were two pairs of scalemic mixture, and their absolute configurations were solved by the chiral HPLC and ECD calculation method (Figures S11–S14).

Compounds **10**–**12** were simple pyrrole alkaloids with the 2-carboxyl and 3-bromo characteristic, which were pairs of scalemic mixture. Their planar structures were determined by HRESIMS, NMR (Tables S3 and S4), and single crystal X-ray diffraction (Figure 3), and the absolute configurations were confirmed by chiral HPLC and ECD calculations (Figures S15–S20).

Five characteristic skeletons of the alkaloids (**1**–**12**) above-mentioned were isolated from the sponge *Stylissa massa*. Compound **1** was the first identified precursor metabolite of the classic 5/7/5 tricyclic skeleton with unesterified guanidine and carboxyl groups. Through the NMR data analysis of compounds **2**, **3**, **13**, **14**, and **15**, an experience rule to determine the *Z*/*E* configurations of double bond ∆^10,11^ was summarized based on the signal intensity in the ^13^C NMR spectra (Figure 4).

**Figure 5:**
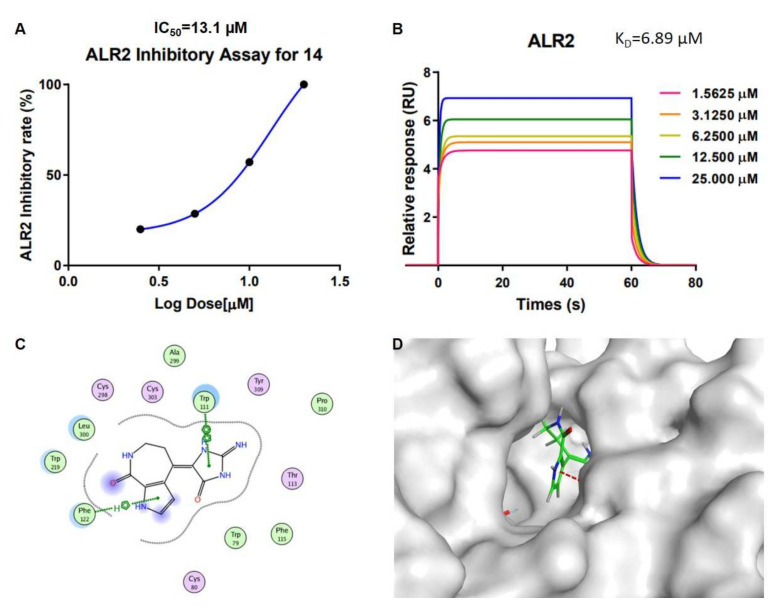
The ALR2 inhibitory activity for compound **14**. (**A**) Concentration dependent curve of the ALR2 inhibitory assay for **14**. (**B**) The result of the surface plasmon resonance (SPR) binding assay of **14** and ALR2 with a K_D_ value of 6.89 µM. (**C**) Ligand interactions between ALR2 and **14**. (**D**) The 3D binding model of compound **14** with ALR2, the surface of the protein is shown in grey, and the interaction bond is shown in the red dotted line.

Some guanidine compounds were reported to exhibit advantageous biological activities on diabetes [14], which indicated the following aldose reductase (ALR2) assay in vitro. We successfully obtained the protein AKR1B1 (ALR2) by genetic engineering methods (Figure S21) and compounds **1**–**16** were tested. Compounds **2**–**5** and **13**–**15**, representative of 5/7/5 tricyclic spogiacidin-type PIA compounds, displayed superior inhibitory activities compared with other compounds (Figure S22) with epalrestat as the positive control. Further concentration gradient experiments carried out to calculate their IC_50_ values showed results that ranged from 8.6 to 13.6 µM (Figure S23). The analysis of the structure–activity relationships indicated that 9-OH and 13-NMe may enhance the ALR2 inhibitory activities of this spongiacidin-alkaloid family. Compounds **13** and **14** with the basic 5/7/5 spongiacidin skeleton without stereoscopic configuration were isolated as the major metabolites in sponge *Stylissa massa*. In order to research the interaction mechanism between spongiacidin-skeleton compounds and ALR2, we carried out surface plasmon resonance (SPR) binding assays of compounds **13** and **14** under the Biacore T200 instrument, where the results showed the binding power (K_D_ value) between ALR2 and compound **13** was 12.5 µM (Figure S24), and **14** was 6.9 µM (Figure 5 and Figure S25 ). Molecular docking using the GBVI/WSA ΔG rescoring method was applied to screen the best docking pose between ALR2 and compound **14**. The results showed that the pyrrole and imidazole ring systems were binding to the pocket of ALR2 by H–π and π–π conjugate bonds, respectively (Figure 5).

## 3. Materials and Methods

### 3.1. General Experimental Procedures

Optical rotations were measured on a JASCO P-1020 digital polarimeter. UV and ECD spectra were obtained on a Jasco J-810 spectropolarimeter (Tokyo, Japan). The NMR spectra were measured by a Bruker AVANCE III 500 MHz spectrometer (Bruker company, Fällanden, Switzerland). The 2.50 ppm and 39.5 ppm resonances of DMSO-*d*_6_ were used as internal references for the ^1^H and ^13^C NMR spectra, respectively. HRESIMS data were measured on Micromass Q-Tof Ultima Global GAA076LC (Waters, Milford, CT, USA) and Thermo Scientific LTQ Orbitrap Exploris 480 mass spectrometers (Waltham, MA, USA). X-ray data were obtained by a Rigaku Xtalab Synergy using Cu-Kα radiation (Tokyo, Japan). Semi-preparative HPLC utilized an ODS column (Agilent XDB C-18, 9.6 × 250 mm, 5 µm). Silica gel (200–400 mesh, Qingdao, China) was used for column chromatography, precoated silica gel plates (GF254, Qingdao, China) were used for TLC, and spots were visualized by heating SiO_2_ plates sprayed with 10% H_2_SO_4_ in EtOH.

### 3.2. Sponge Material

The marine sponge *Stylissa massa* was collected from the Xisha Islands of the South China Sea in June 2013, and was frozen immediately after collection. The specimen was identified by Nicole J. de Voogd, National Museum of Natural History, Leiden, The Netherlands. The voucher specimen (No. XS-2013-07) was deposited at lab A1007, School of Pharmacy, Qingdao University, P. R. China.

### 3.3. Extraction and Isolation

*Stylissa massa* (8.0 kg, wet weight) was crushed and then extracted with MeOH four times (3 days each time) at room temperature. The combined solutions were concentrated in vacuo and the residue was subsequently desalted to yield the organic extract (191.0 g). The extract was subjected to silica gel vacuum liquid chromatography (VLC), eluting with a gradient of petroleum ether/EtOAc (from 10:1 to 0:1, *v*:*v*) and subsequently CH_2_Cl_2_/MeOH (from 10:1 to 0:1, *v*:*v*) to obtain 17 fractions (Fr.1–Fr.17). Fr.5 (0.5 g) was subjected to a silica gel CC (CH_2_Cl_2_/MeOH, 20:1, *v*:*v*) to give three fractions Fr.5-1–Fr.5-3. Fr.5-1 (200 mg) was then subjected to a silica gel CC (petroleum ether/EtOAc, from 5:1 to 1:1, *v*:*v*) to give five fractions Fr.5-1-1–Fr.5-1-5. Fr.5-1-2 was then purified by semi-preparative HPLC (ODS, 5 µm, 250 × 9.6 mm; MeOH/H_2_O, 25:75, *v*/*v*; 2.0 mL/min, 33 min) to afford compound **12** (*t*_R_ = 21.8 min, 1.0 mg) and compound **11** (*t*_R_ = 24.3 min, 1.0 mg). Fr.7 (3.0 g) was subjected to an ODS CC (MeOH/H_2_O, from 5:95 to 100:0, *v*:*v*) to give six fractions Fr.7-1–Fr.7-6. Fr.7-2 was then purified by semi-preparative HPLC (ODS, 5 µm, 250 × 9.6 mm; MeOH/H_2_O, 20:80–60:40, *v*/*v*; 2.0 mL/min, 45 min) to afford compound **10** (*t*_R_ = 19.5 min, 3.0 mg). Fr.7-6 was purified by semi-preparative HPLC (ODS, 5 µm, 250 × 9.6 mm; MeOH/H_2_O, 5:95–100:0, *v*/*v*; 2.0 mL/min, 48 min) to afford compound **8** (*t*_R_ = 40.3 min, 5.8 mg) and compound **9** (*t*_R_ = 45.0 min, 4.0 mg). Fr.8 (14.5 g) was subjected to a silica gel CC (CH_2_Cl_2_/MeOH, 50:1–1:1, *v*:*v*) to give nine fractions Fr.8-1–Fr.8-9. Fr.8-1 (5.0 g) was then subjected to a ODS CC (MeOH/H_2_O, 50:1–1:1, *v*:*v*) to give five fractions Fr.8-1-1–Fr.8-1-5. Fr.8-1-3 was then purified by semi-preparative HPLC (ODS, 5 µm, 250 × 9.6 mm; MeOH/H_2_O, 5:95–100:0, *v*/*v*; 2.0 mL/min, 35 min) to afford compound **16** (*t*_R_ = 19.2 min, 2.0 mg), compound **6** (*t*_R_ = 23.8 min, 3.7 mg) and compound **7** (*t*_R_ = 23.9 min, 3.0 mg). Fr.8-1-5 (4.0 g) was purified subjected to an ODS CC (MeOH/H_2_O, from 5:95 to 100:0, *v*:*v*) to give six fractions Fr.8-1-5-1–Fr.8-1-5-6. Fr.8-1-5-4 (200 mg) was purified by semi-preparative HPLC (ODS, 5 µm, 250 × 9.6 mm; MeOH/H_2_O, 5:95–100:0, *v*/*v*; 2.0 mL/min, 36 min) to afford compound **3** (*t*_R_ = 21.1 min, 2.6 mg), compound **5** (*t*_R_ = 26.1 min, 3.7 mg), and compound **13** (*t*_R_ = 24.7 min, 200 mg). Fr.15 (17.2 g) was subjected to a silica gel CC (CH_2_Cl_2_/MeOH, from 10:1 to 1:1, *v*:*v*) to give four fractions Fr.15-1–Fr.15-4. Fr.15-2 (1.1 g) was then subjected to an ODS CC (MeOH/H_2_O, from 5:95 to 100:0, *v*:*v*) to give six fractions Fr.15-2-1–Fr.15-2-6. Fr.15-2-4 (300 mg) was then purified by semi-preparative HPLC (ODS, 5 µm, 250 × 9.6 mm; MeOH/H_2_O, 10:90, *v*/*v*; 2.0 mL/min, 55 min) to afford compound **2** (*t*_R_ = 29.1 min, 16.0 mg), compound **15** (*t*_R_ = 44.3 min, 6.0 mg), and compound **14** (*t*_R_ = 47.5 min, 150 mg). Fr.17 (20.0 g) was subjected to an ODS CC (MeOH/H_2_O, from 5:95 to 100:0, *v*:*v*) to give seven fractions Fr.17-1–Fr.17-7. Fr.17-3 (800 mg) was then subjected to a silica gel CC (CH_2_Cl_2_/MeOH, from 10:1 to 1:1, *v*:*v*) to give six fractions Fr.17-3-1–Fr.17-3-6. Fr.17-3-3 was then purified by semi-preparative HPLC (ODS, 5 µm, 250 × 9.6 mm; MeOH/H_2_O, 5:90–100:0, *v*/*v*; 2.0 mL/min, 40 min) to afford compound **4** (*t*_R_ = 18.5 min, 6.8 mg). Fr.17-3-4 was then purified by semi-preparative HPLC (ODS, 5 µm, 250 × 9.6 mm; MeOH/H_2_O, 5:90–100:0, *v*/*v*; 2.0 mL/min, 38min) to afford compound **1** (*t*_R_ = 21.2 min, 5.9 mg).

Compound **1:** Colorless crystals; UV (MeOH) λ_max_ 226 nm; ^1^H and ^13^C NMR (DMSO-*d*_6_) data, see Tables S1 and S2; HRESIMS *m*/*z* 342.0184, 344.0162 ([M + H]^+^ (calcd. for C_11_H_13_BrN_5_O_3_, 342.0184, 344.0165); compound **1a**: ${\left[\alpha \right]}_{D}^{20}$ −19.7 (c 0.1, MeOH), compound **1b**: ${\left[\alpha \right]}_{D}^{20}$ 27.3 (c 0.1, MeOH).

Compound **2:** Yellow oil; UV (MeOH) λ_max_ 354 nm; ^1^H and ^13^C NMR (DMSO-*d*_6_) data, see Tables S1 and S2; HRESIMS *m*/*z* 262.0936 ([M + H]^+^ (calcd. for C_11_H_12_N_5_O_3_, 262.0935); compound **2a**: ${\left[\alpha \right]}_{D}^{20}$ −40.3 (c 0.1, MeOH), compound **2b**: ${\left[\alpha \right]}_{D}^{20}$ 34.5 (c 0.1, MeOH).

Compound **3:** Yellow oil; UV (MeOH) λ_max_ 346 nm; ^1^H and ^13^C NMR (DMSO-*d*_6_) data, see Tables S1 and S2; HRESIMS *m*/*z* 262.0933 ([M + H]^+^ (calcd. for C_11_H_12_N_5_O_3_, 262.0935); compound **3a**: ${\left[\alpha \right]}_{D}^{20}$ −29.8 (c 0.1, MeOH), compound **3b**: ${\left[\alpha \right]}_{D}^{20}$ 36.3 (c 0.1, MeOH).

Compound **4:** Yellow oil; UV (MeOH) λ_max_ 346 nm; ^1^H and ^13^C NMR (DMSO-*d*_6_) data, see Tables S1 and S2; HRESIMS *m*/*z* 262.0931 ([M + H]^+^ (calcd. for C_11_H_12_N_5_O_3_, 262.0935); compound **4a**: ${\left[\alpha \right]}_{D}^{20}$ 30.1 (c 0.1, MeOH), compound **4b**: ${\left[\alpha \right]}_{D}^{20}$ −18.4 (c 0.1, MeOH).

Compound **5:** Colorless crystals; UV (MeOH) λ_max_ 354 nm; ^1^H and ^13^C NMR (DMSO-*d*_6_) data, see Tables S1 and S2; HRESIMS *m*/*z* 260.1148 ([M + H]^+^ (calcd. for C_12_H_14_N_5_O_2_, 260.1142).

Compound **6:** Yellow oil; UV (MeOH) λ_max_ 216 nm; ^1^H and ^13^C NMR (DMSO-*d*_6_) data, see Tables S3 and S4; HRESIMS *m*/*z* 356.0357, 358.0353 ([M + H]^+^ (calcd. for C_12_H_15_BrN_5_O_3_, 356.0353, 358.0332); ${\left[\alpha \right]}_{D}^{20}$ −19.5 (c 0.1, MeOH).

Compound **7:** Yellow oil; UV (MeOH) λ_max_ 216 nm; ^1^H and ^13^C NMR (DMSO-*d*_6_) data, see Tables S3 and S4; HRESIMS *m*/*z* 195.0770 ([M + H]^+^ (calcd. for C_9_H_11_N_2_O_3_, 195.0764); compound **7a**: ${\left[\alpha \right]}_{D}^{20}$ 27.7 (c 0.1, MeOH), compound **7b**: ${\left[\alpha \right]}_{D}^{20}$ −24.1 (c 0.1, MeOH).

Compound **8:** Yellow oil; UV (MeOH) λ_max_ 276 nm; ^1^H and ^13^C NMR (DMSO-*d*_6_) data, see Tables S3 and S4; HRESIMS *m*/*z* 287.0025, 289.0003 ([M + H]^+^ (calcd. for C_10_H_12_BrN_2_O_3_, 287.0026, 289.0005); compound **8a**: ${\left[\alpha \right]}_{D}^{20}$ 23.3 (c 0.1, MeOH), compound **8b**: ${\left[\alpha \right]}_{D}^{20}$ −20.8 (c 0.1, MeOH).

Compound **9:** Yellow oil; UV (MeOH) λ_max_ 250 nm; ^1^H and ^13^C NMR (DMSO-*d*_6_) data, see Tables S3 and S4; HRESIMS *m*/*z* 284.9867, 286.9845 ([M + H]^+^ (calcd. for C_10_H_10_BrN_2_O_3_, 284.9869, 286.9849).

Compound **10:** Colorless crystals; UV (MeOH) λ_max_ 240 nm; ^1^H and ^13^C NMR (DMSO-*d*_6_) data, see Tables S3 and S4; HRESIMS *m*/*z* 234.9715, 236.9695 ([M + H]^+^ (calcd. for C_6_H_8_BrN_2_O_3_, 234.9713, 236.9692); compound **10a**: ${\left[\alpha \right]}_{D}^{20}$ 26.8 (c 0.1, MeOH), compound **10b**: ${\left[\alpha \right]}_{D}^{20}$ −32.4 (c 0.1, MeOH).

Compound **11:** Yellow oil; UV (MeOH) λ_max_ 216 nm; ^1^H and ^13^C NMR (DMSO-*d*_6_) data, see Tables S3 and S4; HRESIMS *m*/*z* 235.9920, 237.9899 ([M + H]^+^ (calcd. for C_7_H_11_BrNO_3_, 235.9920, 237.9899); compound **11a**: ${\left[\alpha \right]}_{D}^{20}$ 52.9 (c 0.1, MeOH), compound **11b**: ${\left[\alpha \right]}_{D}^{20}$ −46.5 (c 0.1, MeOH).

Compound **12:** Yellow oil; UV (MeOH) λ_max_ 212 nm; ^1^H and ^13^C NMR (DMSO-*d*_6_) data, see Tables S3 and S4; HRESIMS *m*/*z* 321.0073, 323.0053 ([M + H]^+^ (calcd. for C_10_H_14_BrN_2_O_5_, 321.0081, 323.0060); compound **12a**: ${\left[\alpha \right]}_{D}^{20}$ 20.0 (c 0.1, MeOH), compound **12b**: ${\left[\alpha \right]}_{D}^{20}$ −13.3 (c 0.1, MeOH).

Compound **13:** Yellow oil; UV (MeOH) λ_max_ 352 nm; C_11_H_11_N_5_O_2_; ^1^H NMR (DMSO-*d*_6_) data, *δ*_H_ 12.1 (brs, 1-NH), 8.04 (t, *J* = 4.3, 7-NH), 7.11 (t, *J* = 2.4, H-2), 6.54 (t, *J* = 2.4, H-3), 3.28 (m, H_2_-8 and H_2_-9); ^13^C NMR (DMSO-*d*_6_) data, *δ*_C_ 164.4, 163.0, 154.9, 129.6, 126.6, 122.6, 121.0, 120.4, 109.6, 39.1, and 31.4. [18]

Compound **14:** Yellow oil; UV (MeOH) λ_max_ 352 nm; C_11_H_11_N_5_O_2_; ^1^H NMR (DMSO-*d*_6_) data, *δ*_H_ 11.9 (brs, 1-NH), 7.98 (t, *J* = 4.3, 7-NH), 6.91 (t, *J* = 2.3, H-2), 6.79 (t, *J* = 2.3, H-3), 3.26 (q, *J* = 4.5, H_2_-8), 2.85 (q, *J* = 4.5, H_2_-9); ^13^C NMR (DMSO-*d*_6_) data, *δ*_C_ 163.9, 161.2, 153.4, 130.5, 126.1, 122.3, 120.4, 118.6, 112.6, 38.3, and 36.6. [16]

Compound **15:** Yellow oil; UV (MeOH) λ_max_ 360 nm; C_12_H_13_N_5_O_3_; ^1^H NMR (DMSO-*d*_6_) data, *δ*_H_ 12.0 (brs, 1-NH), 7.76 (dd, *J* = 6.5, 1.7, 7-NH), 7.09 (t, *J* = 2.8, H-2), 6.52 (m, H-3), 5.73 (d, *J* = 6.7, H-9), 3.57, 3.28 (m, H_2_-8), 3.21 (s, 9-OMe); ^13^C NMR (DMSO-*d*_6_) data, *δ*_C_ 164.8, 162.6, 155.8, 130.3, 123.3, 122.9, 127.1, 123.3, 117.5, 110.6, 69.0, and 43.2. [19]

Compound **16:** Yellow oil; UV (MeOH) λ_max_ 214 nm; C_12_H_14_BrN_5_O_3_; ^1^H NMR (DMSO-*d*_6_) data, *δ*_H_ 9.78 (brs, 9-NH), 9.44 (brs, 7-NH), 8.10 (brs, 16-NH_2_), 7.68 (s, H-3), 5.74 (s, H-6), 3.47, 3.36 (m, H_2_-13), 2.24 (m, H_2_-11), 1.98 (m, H_2_-12); ^13^C NMR (DMSO-*d*_6_) data, *δ*_C_ 163.6, 163.1, 156.5, 146.0, 118.4, 85.8, 81.4, 63.9, 44.8, 39.5, 19.7 [17].

### 3.4. Computational Section

Conformational analyses were carried out in the MMFF minimization force field by the Spartan 10 v1.2.4 software package (Microsoft, Redmond, WA, USA). The resulting conformers were optimized using DFT at the B3LYP/6-31+G(d,p) level in the gas phase by the GAUSSIAN 09 C.03 program (Gaussian, Inc. Wallingford, CT, USA). The optimized conformations, whose Boltzmann distributions of Gibbs free energies were more than 1.0 percent, were used for the ECD calculations using the TD-DFT method with the basis set RB3LYP/DGDZVP, or the NMR calculations using the GIAO method at the PCM/b3lyp/6-311+G(d,p) level.

### 3.5. Molecular Docking

The initial receptor structure was constructed based on the crystal structure of aldose reductase in complex with cofactor NADP+ and the inhibitor idd594 (PDB code: 1US0) from the Protein Data Bank [20]. All nonstandard groups (HETATM) were deleted except for the inhibitor and cofactor when preparing for the receptor structure. The Protonate 3D module in the Molecular Operating Environment (MOE) program was used to estimate the protonated state of titratable residues and add hydrogen atoms. Meanwhile, MOE was also used to construct the ligand structures (compound **14**). The subsequent molecular docking was performed with an induced fit protocol. During the process, the protein–cofactor complex was defined as the receptor and the position of the inhibitor idd594 was defined as the docking site. The triangle matcher placement with the London ΔG initial scoring methodology was set for conformational sampling and 100 poses were recorded, then the forcefield post-placement refinement with GBVI/WSA ΔG rescoring methodology was utilized to further screen the best docking pose. In addition, the MMFF94x force field was adopted for the whole process.

### 3.6. ALR2 Expression and Purification

For the AKR1B1 enzyme assay and SPR measurements, the protein was expressed and purified using the protocol related to the one reported previously [15]. The plasmid (pET28b, Novagen) containing the open reading frame of the human ALR2 gene was kindly provided by Atagenix, Wuhan, Hubei, China. The E. coli strain BL21 gold (DE3) (Novagen) was used to express the hexa-histidine tagged protein after induction with IPTG (Roth) for 16 h at 293 K. The pellet from a 4 L culture was resuspended in a buffer containing 20 mM Tris and 500 mM NaCl (pH 8.0) before being sonicated and centrifuged. A HiTrap chelating HP column (GE Healthcare) was loaded with the supernatant. After a short washing step with a low imidazole concentration, the fusion protein was eluted by applying a gradient of imidazole. The buffer was exchanged with 20mM Tris-HCl, 10 mM NaCl, 1mM EDTA, and the tag cleaved by thrombin (yuanyeBio-Technology Co. Ltd. Shanghai, China). A Hiprep DEAE FastFlow 16/10 column (GE Healthcare) was loaded with the remaining solution. A NaCl gradient was used to elute the ALR2 from the column.

### 3.7. ALR2 Enzyme Assay (In Vitro)

In the first step of the polyol pathway, glucose reduction is accompanied by the conversation of NADPH to NADP, where only NADPH has an obvious spectral absorption around 340 nm and NADP has none. Thus, the decrease in OD_340_nm can represent the consumption of NADPH. Therefore, we detect the change of OD_340_nm before and after reaction to screen the effective ARIs or evaluate the AR activity; assays with epalrestat were used as positive controls. Briefly, the incubation system contains 10 µL ALR2 enzyme (20 µg/mL), 80 µL DL-glyceraldehyde (0.16 mmol/L) as the substrate, 1.0 mmol/L NADPH·4Na as a coenzyme, and 0.1 mol/L PBS (pH = 6.2). The incubation mixture was minimized to a total volume of 100 µL and ongoing in a 96-well ultraviolet plate. Then, test wells were treated with the tested compounds for 10 min at 25 °C. Then, the absorbance was measured using FlexStation 3 Reader (Molecular Devices, San Francisco, CA, USA) at 340 nm. The absorbance of wells treated with PBS and NADPH was considered as 100% (OD_1_) and the absorbance of wells treated with PBS and DL-glyceraldehyde was considered as 0% (OD_2_); the inhibitory rate was calculated by the formula OD_compounds_ − OD_2_/OD_1_ − OD_2_. IC_50_ represents the concentration that inhibits the ALR2 enzyme by 50%.

### 3.8. Surface Plasmon Resonance (SPR) Binding Assay

SPR binding analysis methodology can be used to study molecular interactions. Herein, SPR was applied to measure the interactions between compounds **13**/**14** and ALR2. Initially, ALR2 was prepared in 10 mM sodium acetate (pH 5.0) and then immobilized covalently by an amine-coupling reaction on a CM5 sensor chip. The remaining binding sites of the sensor chip were then blocked by ethanolamine. The addition of compound **13**/**14**, the flow-through analyte, to the chamber resulted in binding to the immobilized protein ligand, producing a small change in the refractive index at the gold surface. In this step, the compound was diluted in PBS-P^+^ buffer to the desired concentration and was injected over the chip with a flow rate of 10 μL/min. All of the above buffers, solutions, and sensor chips were placed at room temperature before the run. The association time and dissociation time were both set at 60 s. Binding affinities were obtained from the ratio of rate constants to directly characterize the protein-molecular interactions. Data analysis was completed via the state model in T200 evaluation software (Cytiva Danaher, Marlborough, MA, USA).

## 4. Conclusions

In conclusion, a series of new pyrrole alkaloids including different structural scaffolds were isolated from the sponge *Stylissa massa* collected in the South China Sea, which enriched the structural diversity of this alkaloid family. Aldose reductase (ALR2), which participates in the glucose polyol metabolic pathway and cell inflammatory reaction, is an important target for the treatment of diabetes complications, and 5/7/5 tricyclic spongiacidin-PIAs isolated from sponge *Stylissa massa* provided a new skeleton targeted to ALR2 which have never been previously reported.

## Data Availability

The original contributions presented in the study are included in the article/Supplementary Materials; further inquiries can be directed to the corresponding author.

## Supplementary Materials

The following supporting information can be downloaded at: https://www.mdpi.com/article/10.3390/md20070454/s1, Tables S1–S4: 1H (500 MHz) and 13C (125 MHz) NMR data for 1–14 acquired in DMSO-d6. Tables S5–S7: X-ray diffraction analysis of compounds 1, 5, and 10. Figures S1–S20: The chiral HPLC data and ECD spectra of compounds 1–12; Figures S21–S25: The ALR2 inhibitory activities of related compounds. Figures S26–S117: The spectroscopic data (including HRESIMS, 1H NMR, 13C NMR, and 2D NMR) of compounds 1–16.
