# Supplementary material for: A Series of New Pyrrole Alkaloids with ALR2 Inhibitory Activities from the Sponge Stylissa massa
Source: Mar Drugs. 2022 Jul 12;20(7):454. doi: 10.3390/md20070454 (PMC9320028; doi:10.3390/md20070454)
Supplement: Supplementary file 1 [file marinedrugs-20-00454-s001.zip › marinedrugs-1802470 Supporting information-Marine Drugs.pdf]

Supporting Information

A Series of new Pyrrole Alkaloids with ALR2 Inhibitory Activities from the Sponge *Stylissa massa*

Qi Wang,[†,▽] Chunhua Gao,[†,▽] Zhun Wei,[†] Xiaowen Tang,[†] Lixia Ji,[†] Xiangchao Luo,[‡] Xiaoping Peng,[†] Gang Li,[†] and Hongxiang Lou[*,†]

[†]Department of Natural Medicinal Chemistry and Pharmacognosy, School of Pharmacy, Qingdao University, Qingdao 266021, People's Republic of China.

[‡]Research Center for Marine Drugs, Department of Pharmacy, Ren Ji Hospital, School of Medicine, Shanghai Jiao Tong University, Shanghai, 200127, People's Republic of China.

*E-mail: louhongxiang@sdu.edu.cn.

Table of Contents

Table S1	^{13}C (125 MHz) NMR Data for **1−5** and **13-14** acquired in DMSO-d_6.	6
Table S2	^{1}H (500 MHz) NMR Data for **1−5** and **13-14** acquired in DMSO-d_6.	6
Table S3	^{13}C (125 MHz) NMR Data for **6-12** acquired in DMSO-d_6.	7
Table S4	^{1}H (500 MHz) NMR Data for **6-12** acquired in DMSO-d_6.	7
Table S5	X-ray diffraction analysis of compound **1**.	8
Table S6	X-ray diffraction analysis of compound **5**.	9
Table S7	X-ray diffraction analysis of compound **10**.	10
Figure S1	The LC-CD analysis figure of compounds **1a** (R_f=16.2 min), **1b** (R_f=18.8 min) on chiral Daicel Chiralpack AD-H column (250 × 4.6 mm, 5 μm) under the HPLC condition of n-hexane/ isopropanol (75:25) solution.	11
Figure S2	Calculated and experimental ECD spectra of compounds **1a** and **1b**.	11
Figure S3	The LC-CD analysis figure of compounds **2a** (R_f=9.2 min), **2b** (R_f=13.5 min) on chiral Daicel Chiralpack AD-H column (250 × 4.6 mm, 5 μm) under the HPLC condition of n-hexane/ isopropanol (65:35) solution.	12
Figure S4	Calculated and experimental ECD spectra of compounds **2a** and **2b**.	12
Figure S5	The LC-CD analysis figure of compounds **3a** (R_f=13.1 min), **3b** (R_f=17.6 min) on chiral Daicel Chiralpack AD-H column (250 × 4.6 mm, 5 μm) under the HPLC condition of n-hexane/ isopropanol (90:10) solution.	13
Figure S6	Calculated and experimental ECD spectra of compounds **3a** and **3b**.	13
Figure S7	The LC-CD analysis figure of compounds **4a** (R_f=6.8 min), **4b** (R_f=9.6 min) on chiral Daicel Chiralpack AD-H column (250 × 4.6 mm, 5 μm) under the HPLC condition of n-hexane/ isopropanol (65:35) solution.	14
Figure S8	Calculated and experimental ECD spectra of compounds **4a** and **4b**.	14
Figure S9	Detailed DP4+ probability (calculated at PCM/b3lyp/6-311+G(d,p) level) for compounds **6** and **16** (Isomer 1: 2*R*6*R*10*R*, Isomer 2: 2*R*6*R*10*S*, Isomer 3: 2*R*6*S*10*R*, Isomer 4: 2*R*6*S*10*S*).	15
Figure S10	Calculated and experimental ECD spectra of compounds **6** and **16**.	15
Figure S11	The LC-CD analysis figure of compounds **7a** (R_f=52.2 min), **7b** (R_f=60.5 min) on chiral Daicel Chiralpack AD-H column (250 × 4.6 mm, 5 μm) under the HPLC condition of n-hexane/ isopropanol (90:10) solution.	16
Figure S12	Calculated and experimental ECD spectra of compounds **7a** and **7b**.	16
Figure S13	The LC-CD analysis figure of compounds **8a** (R_f=30.6 min), **8b** (R_f=36.2 min) on chiral Daicel Chiralpack AD-H column (250 × 4.6 mm, 5 μm) under the HPLC condition of n-hexane/ isopropanol (90:10) solution.	17
Figure S14	Calculated and experimental ECD spectra of compounds **8a** and **8b**.	17
Figure S15	The LC-CD analysis figure of compounds **10a** (R_f=19.8 min), **10b** (R_f=26.6 min) on chiral Daicel Chiralpack AD-H column (250 × 4.6 mm, 5 μm) under the HPLC condition of n-hexane/ isopropanol (90:10) solution.	18
Figure S16	Calculated and experimental ECD spectra of compounds **10a** and **10b**.	18
Figure S17	The LC-CD analysis figure of compounds **11a** (R_f=14.9 min), **11b** (R_f=17.8 min) on chiral Daicel Chiralpack AD-H column (250 × 4.6 mm, 5 μm) under	19

	the HPLC condition of n-hexane/ isopropanol (90:10) solution.	
Figure S18	Calculated and experimental ECD spectra of compounds **10a** and **10b**.	19
Figure S19	The LC-CD analysis figure of compounds **12a** (R_f=8.1 min), **12b** (R_f=11.8 min) on chiral Daicel Chiralpack AD-H column (250 × 4.6 mm, 5 μm) under the HPLC condition of n-hexane/ isopropanol (80:20) solution.	20
Figure S20	Calculated and experimental ECD spectra of compounds **12a** and **12b**.	20
Figure S21	SDS PAGE of the ALR2 stability investigation for surface plasmon resonance (SPR) experiment during 0-8h at room temperature.	21
Figure S22	Compounds **2-5** and **13-15**, representative of 5/7/5 tricyclic spogiacidin-type PIA compounds, displayed the superior activities (>50% inhibition rate) at 20 μM.	21
Figure S23	IC_{50} values of ALR2 inhibitory activities for spongiacidin-type PIAs (compounds **2-5** and **13-15**).	22
Figure S24	SPR experiment of compound **13** using the Biacore T200 instrument.	22
Figure S25	SPR experiment of compound **14** using the Biacore T200 instrument.	23
Figure S26	^{1}H NMR spectrum of compound **1** in DMSO-d_6 (500 MHz).	24
Figure S27	^{13}C NMR spectrum of compound **1** in DMSO-d_6 (125 MHz).	25
Figure S28	^{13}C NMR and DEPT spectrum of compound **1** in DMSO-d_6 (125 MHz).	26
Figure S29	^{1}H-^{1}H COSY spectrum of compound **1** in DMSO-d_6 (500 MHz).	27
Figure S30	HSQC spectrum of compound **1** in DMSO-d_6 (500 MHz).	28
Figure S31	HMBC spectrum of compound **1** in DMSO-d_6 (500 MHz).	29
Figure S32	HRESIMS data of compound **1**.	30
Figure S33	^{1}H NMR spectrum of compound **2** in DMSO-d_6 (500 MHz).	31
Figure S34	^{13}C NMR spectrum of compound **2** in DMSO-d_6 (125 MHz).	32
Figure S35	^{13}C NMR and DEPT spectrum of compound **2** in DMSO-d_6 (125 MHz).	33
Figure S36	^{1}H-^{1}H COSY spectrum of compound **2** in DMSO-d_6 (500 MHz).	34
Figure S37	HSQC spectrum of compound **2** in DMSO-d_6 (500 MHz).	35
Figure S38	HMBC spectrum of compound **2** in DMSO-d_6 (500 MHz).	36
Figure S39	HRESIMS data of compound **2**.	37
Figure S40	^{1}H NMR spectrum of compound **3** in DMSO-d_6 (500 MHz).	38
Figure S41	^{13}C NMR spectrum of compound **3** in DMSO-d_6 (125 MHz).	39
Figure S42	^{13}C NMR and DEPT spectrum of compound **3** in DMSO-d_6 (125 MHz).	40
Figure S43	^{1}H-^{1}H COSY spectrum of compound **3** in DMSO-d_6 (500 MHz).	41
Figure S44	HSQC spectrum of compound **3** in DMSO-d_6 (500 MHz).	42
Figure S45	HMBC spectrum of compound **3** in DMSO-d_6 (500 MHz).	43
Figure S46	HRESIMS data of compound **3**.	44
Figure S47	^{1}H NMR spectrum of compound **4** in DMSO-d_6 (500 MHz).	45
Figure S48	^{13}C NMR spectrum of compound **4** in DMSO-d_6 (125 MHz).	46
Figure S49	^{13}C NMR and DEPT spectrum of compound **4** in DMSO-d_6 (125 MHz).	47
Figure S50	^{1}H-^{1}H COSY spectrum of compound **4** in DMSO-d_6 (500 MHz).	48

Figure S51	HSQC spectrum of compound **4** in DMSO-d_6 (500 MHz).	49
Figure S52	HMBC spectrum of compound **4** in DMSO-d_6 (500 MHz).	50
Figure S53	HRESIMS data of compound **4**.	51
Figure S54	^{1}H NMR spectrum of compound **5** in DMSO-d_6 (500 MHz).	52
Figure S55	^{13}C NMR spectrum of compound **5** in DMSO-d_6 (125 MHz).	53
Figure S56	^{13}C NMR and DEPT spectrum of compound **5** in DMSO-d_6 (125 MHz).	54
Figure S57	^{1}H-^{1}H COSY spectrum of compound **5** in DMSO-d_6 (500 MHz).	55
Figure S58	HSQC spectrum of compound **5** in DMSO-d_6 (500 MHz).	56
Figure S59	HMBC spectrum of compound **5** in DMSO-d_6 (500 MHz).	57
Figure S60	HRESIMS data of compound **5**.	58
Figure S61	^{1}H NMR spectrum of compound **6** in DMSO-d_6 (500 MHz).	59
Figure S62	^{13}C NMR spectrum of compound **6** in DMSO-d_6 (125 MHz).	60
Figure S63	^{13}C NMR and DEPT spectrum of compound **6** in DMSO-d_6 (125 MHz).	61
Figure S64	^{1}H-^{1}H COSY spectrum of compound **6** in DMSO-d_6 (500 MHz).	62
Figure S65	HSQC spectrum of compound **6** in DMSO-d_6 (500 MHz).	63
Figure S66	HMBC spectrum of compound **6** in DMSO-d_6 (500 MHz).	64
Figure S67	HRESIMS data of compound **6**.	65
Figure S68	^{1}H NMR spectrum of compound **7** in DMSO-d_6 (500 MHz).	66
Figure S69	^{13}C NMR spectrum of compound **7** in DMSO-d_6 (125 MHz).	67
Figure S70	^{13}C NMR and DEPT spectrum of compound **7** in DMSO-d_6 (125 MHz).	68
Figure S71	^{1}H-^{1}H COSY spectrum of compound **7** in DMSO-d_6 (500 MHz).	69
Figure S72	HSQC spectrum of compound **7** in DMSO-d_6 (500 MHz).	70
Figure S73	HMBC spectrum of compound **7** in DMSO-d_6 (500 MHz).	71
Figure S74	HRESIMS data of compound **7**.	72
Figure S75	^{1}H NMR spectrum of compound **8** in DMSO-d_6 (500 MHz).	73
Figure S76	^{13}C NMR spectrum of compound **8** in DMSO-d_6 (125 MHz).	74
Figure S77	^{13}C NMR and DEPT spectrum of compound **8** in DMSO-d_6 (125 MHz).	75
Figure S78	^{1}H-^{1}H COSY spectrum of compound **8** in DMSO-d_6 (500 MHz).	76
Figure S79	HSQC spectrum of compound **8** in DMSO-d_6 (500 MHz).	77
Figure S80	HMBC spectrum of compound **8** in DMSO-d_6 (500 MHz).	78
Figure S81	HRESIMS data of compound **8**.	79
Figure S82	^{1}H NMR spectrum of compound **9** in DMSO-d_6 (500 MHz).	80
Figure S83	^{13}C NMR spectrum of compound **9** in DMSO-d_6 (125 MHz).	81
Figure S84	^{13}C NMR and DEPT spectrum of compound **9** in DMSO-d_6 (125 MHz).	82
Figure S85	^{1}H-^{1}H COSY spectrum of compound **9** in DMSO-d_6 (500 MHz).	83
Figure S86	HSQC spectrum of compound **9** in DMSO-d_6 (500 MHz).	84
Figure S87	HMBC spectrum of compound **9** in DMSO-d_6 (500 MHz).	85
Figure S88	HRESIMS data of compound **9**.	86
Figure S89	^{1}H NMR spectrum of compound **10** in DMSO-d_6 (500 MHz).	87
Figure S90	^{13}C NMR spectrum of compound **10** in DMSO-d_6 (125 MHz).	88

Figure S91	^{13}C NMR and DEPT spectrum of compound **10** in DMSO-d_6 (125 MHz).	89
Figure S92	^{1}H-^{1}H COSY spectrum of compound **10** in DMSO-d_6 (500 MHz).	90
Figure S93	HSQC spectrum of compound **10** in DMSO-d_6 (500 MHz).	91
Figure S94	HMBC spectrum of compound **10** in DMSO-d_6 (500 MHz).	92
Figure S95	HRESIMS data of compound **10**.	93
Figure S96	^{1}H NMR spectrum of compound **11** in DMSO-d_6 (500 MHz).	94
Figure S97	^{13}C NMR spectrum of compound **11** in DMSO-d_6 (125 MHz).	95
Figure S98	^{13}C NMR and DEPT spectrum of compound **11** in DMSO-d_6 (125 MHz).	96
Figure S99	^{1}H-^{1}H COSY spectrum of compound **11** in DMSO-d_6 (500 MHz).	97
Figure S100	HSQC spectrum of compound **11** in DMSO-d_6 (500 MHz).	98
Figure S101	HMBC spectrum of compound **11** in DMSO-d_6 (500 MHz).	99
Figure S102	HRESIMS data of compound **11**.	100
Figure S103	^{1}H NMR spectrum of compound **12** in DMSO-d_6 (500 MHz).	101
Figure S104	^{13}C NMR spectrum of compound **12** in DMSO-d_6 (125 MHz).	102
Figure S105	^{13}C NMR and DEPT spectrum of compound **12** in DMSO-d_6 (125 MHz).	103
Figure S106	^{1}H-^{1}H COSY spectrum of compound **12** in DMSO-d_6 (500 MHz).	104
Figure S107	HSQC spectrum of compound **12** in DMSO-d_6 (500 MHz).	105
Figure S108	HMBC spectrum of compound **12** in DMSO-d_6 (500 MHz).	106
Figure S109	HRESIMS data of compound **12**.	107
Figure S110	^{1}H NMR spectrum of compound **13** in DMSO-d_6 (500 MHz).	108
Figure S111	^{13}C NMR spectrum of compound **13** in DMSO-d_6 (125 MHz).	109
Figure S112	^{1}H NMR spectrum of compound **14** in DMSO-d_6 (500 MHz).	110
Figure S113	^{13}C NMR spectrum of compound **14** in DMSO-d_6 (125 MHz).	111
Figure S114	^{1}H NMR spectrum of compound **15** in DMSO-d_6 (500 MHz).	112
Figure S115	^{13}C NMR spectrum of compound **15** in DMSO-d_6 (125 MHz).	113
Figure S116	^{1}H NMR spectrum of compound **16** in DMSO-d_6 (500 MHz).	114
Figure S117	^{13}C NMR spectrum of compound **16** in DMSO-d_6 (125 MHz).	115

Table S1. ^{13}C (125 MHz) NMR Data for **1−5** and **13−14** acquired in DMSO-d_6.

no.	**1**	**2**	**3**	**4**	**5**	**13**	**14**
	δ_C, type	δ_C, type	δ_C, type	δ_C, type	δ_C, type	δ_C, type	δ_C, type
2	104.3, C	122.7, CH	122.0, CH	122.6, CH	122.4, CH	122.6, CH	120.4, CH
3	108.5, CH	110.0, CH	110.2, CH	107.2, CH	110.4, CH	109.6, CH	112.6, CH
4	123.3, C	117.8, C	118.7, C	121.9, C	120.4, C	120.4, C	118.6, C
5	127.9, C	126.5, C	125.3, C	123.6, C	126.6, C	126.6, C	126.1, C
6	162.1, C	162.9, C	163.3, C	163.4, C	163.0, C	163.0, C	163.9, C
8	37.4, CH_2	45.4, CH_2	45.6, CH_2	39.9, CH_2	39.0, CH_2	39.1, CH_2	38.3, CH_2
9	125.0, CH	62.8, CH	63.4, CH	126.7, CH	31.5, CH_2	31.4, CH_2	36.6, CH_2
10	133.6, C	132.0, C	125.7, C	105.8, C	132.0, C	129.6, C	130.5, C
11	57.2, CH	122.4, C	124.7, C	53.3, CH	122.4, C	121.0, C	122.3, C
12	170.5, C	163.9, C	165.2, C	171.2, C	163.0, C	164.4, C	161.2, C
14	156.4, C	155.4, C	154.4, C	152.3, C	154.7, C	154.9, C	153.4, C

Table S2. ^{1}H (500 MHz) NMR Data for **1−5** and **13−14** acquired in DMSO-d_6.

no.	**1**	**2**	**3**	**4**	**5**	**13**	**14**
	δ_H (J in Hz)	δ_H (J in Hz)	δ_H (J in Hz)	δ_H (J in Hz)	δ_H (J in Hz)	δ_H (J in Hz)	δ_H (J in Hz)
1−NH	12.71, brs	12.05, brs	11.75, brs	11.79, s	12.05, brs	12.10, brs	11.90, brs
2		7.09, t (2.8)	6.96, t (2.8)	7.00, t (3.5)	7.09, t (2.3)	7.13, t (2.4)	6.91, t (2.3)
3	6.36, d (2.3)	6.45, t (2.4)	6.43, t (2.5)	6.44, t (3.1)	6.71, m	6.50, t (2.4)	6.79, t (2.2)
4							
5							
6							
7-NH	7.79, t (4.7)	7.75, dd (5.5, 3.9)	7.59, t (4.3)	7.83, t (5.0)	8.03, t (4.5)	8.05, t (4.3)	7.98, t (4.3)
8	3.42, m	3.29, m; 3.24, dq (14.3, 2.1)	3.26, m; 3.19, m	3.69, 3.60, dd (15.5, 5.0)	3.28, m	3.28, m	3.26, q (4.5)
9	6.04, t (6.8)	5.80, dd (7.4, 2.0)	5.83, d (6.6)		3.34, m	3.28, m	2.85, q (4.5)
10							
11	5.21, d (8.5)			4.97, d (3.6)			
12							
13-NH			11.16, s				
14							
15-NH	7.80, d (8.3)		9.45, s	8.90, brs			
16-NH				8.12, brs			9.23, brs
13-NMe					3.11, s		
9-OH			4.99, brs	10.89, brs			

Table S3. ^{13}C (125 MHz) NMR Data for **6-12** acquired in DMSO-d_6.

no.	**6**	**7**	**8**	**9**	**10**	**11**	**12**
	δ_C, type	δ_C, type	δ_C, type	δ_C, type	δ_C, type	δ_C, type	δ_C, type
2	89.7, C	121.5, CH	103.6, C	104.0, C	167.0, C	166.0, C	167.0, C
3	144.2, CH	110.0, CH	112.1, CH	111.2, CH	121.2, C	120.5, C	121.4, C
4	120.3, C	122.6, C	123.7, C	121.3, C	144.5, CH	145.9, CH	144.4, CH
5	163.1, C	122.7, C	124.9, C	127.9, C	92.0, C	91.9, C	91.9, C
6	63.9, CH	163.1, C	162.0, C	162.2, C	167.5, C	74.1, CH_2	165.5, C
8	156.4, C	38.6, CH_2	38.4, CH_2	37.4, CH_2			35.3, CH_2
9		30.4, CH_2	30.3, CH_2	134.3, CH			33.2, CH_2
10	81.3, C	43.8, CH	43.4, CH	130.3, CH			171.6, C
11	39.5, CH_2	175.0, C	173.6, C	166.0, C			
12	19.7, CH_2						
13	45.0, CH_2						
15	161.9, C						
2-OMe	50.6, CH_3						
5-OMe					51.1, CH_3	49.7, CH_3	51.0, CH_3
6-OMe						59.1, CH_3	
10-OMe							51.4, CH_3
11-OMe			52.0, CH_3	52.1, CH_3			

Table S4. ^{1}H (500 MHz) NMR Data for **6-12** acquired in DMSO-d_6.

no.	**6**	**7**	**8**	**9**	**10**	**11**	**12**
	δ_H (J in Hz)	δ_H (J in Hz)	δ_H (J in Hz)	δ_H (J in Hz)	δ_H (J in Hz)	δ_H (J in Hz)	δ_H (J in Hz)
1−NH		11.17, brs	12.05, brs		9.07, brs	8.81, brs	9.13, brs
2		6.83, t (2.7)					
3	7.79, s	6.06, t (2.4)	6.06, s	6.54, s			
4					7.35, s	7.32, d (1.6)	7.31, d (0.9)
5							
6	5.80, s					3.56, 3.42, d (10.0)	
7-NH	9.53, brs	7.65, brs	7.80, t (4.5)	7.83, t (4.9)	7.64, d (15.8)		8.31, t (5.9)
8		3.27, m;	3.22, m; 3.14, m	3.50, dd (6.8, 5.3)			3.33, m
9	9.88, brs	2.08, q (6.0)	2.08, q (6.2)	6.91, t (7.0)			2.51, m
10		3.79, t (6.3)	3.92, t (6.6)				
11	2.25, m						
12	1.98, m						
13	3.47, tt (8.0, 2.7);						
14							
15							
16-NH	8.23, brs						
2-OMe	3.15, s						
5-OMe					3.17, s	3.07, s	3.18, s
6-OMe						3.27, s	
10-OMe							3.59, s
11-OMe			3.64, s	3.75, s			

Table S5. X-ray diffraction analysis of compound **1**.

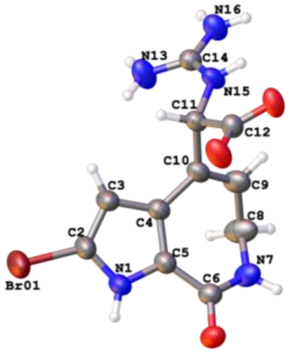

Empirical formula	$C_{13}H_{18}BrN_5O_4$
Formula weight	388.23
Temperature/K	298
Crystal system	monoclinic
Space group	P21/n
a/Å	9.0283(3)
b/Å	15.5478(3)
c/Å	11.7424(3)
α/°	90
β/°	90.939(2)
γ/°	90
Volume/Å^3	1648.06(8)
Z	4
ρ_{calc}g/cm^3	1.565
μ/mm^{-1}	3.656
F(000)	792.0
Crystal size/mm^3	0.2 × 0.1 × 0.1
Radiation	CuKα (λ = 1.54184)
2Θ range for data collection/°	9.44 to 152.61
Index ranges	-11 ≤ h ≤ 9, -19 ≤ k ≤ 18, -14 ≤ l ≤ 14
Reflections collected	11402
Independent reflections	3322 [Rint = 0.0858, Rsigma = 0.0906]
Data/restraints/parameters	3322/0/210
Goodness-of-fit on F^2	1.077
Final R indexes [I>=2σ (I)]	R1 = 0.0556, wR2 = 0.1518
Final R indexes [all data]	R1 = 0.0886, wR2 = 0.1736
Largest diff. peak/hole / e Å^{-3}	0.55/-0.79

Table S6. X-ray diffraction analysis of compound **5**.

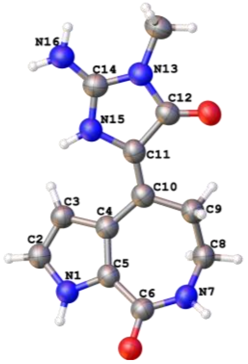

Empirical formula	$C_{14}H_{16}F_3N_5O_5$
Formula weight	391.32
Temperature/K	150.0
Crystal system	monoclinic
Space group	P21/n
a/Å	20.7232(6)
b/Å	8.2740(2)
c/Å	20.9101(6)
α/°	90
β/°	117.162(2)
γ/°	90
Volume/Å^3	3189.93(16)
Z	8
ρ_{calc}g/cm^3	1.630
μ/mm^{-1}	1.291
F(000)	1616.0
Crystal size/mm^3	0.2 × 0.15 × 0.1
Radiation	CuKα (λ = 1.54178)
2Θ range for data collection/°	4.974 to 144.002
Index ranges	-25 ≤ h ≤ 25, -10 ≤ k ≤ 9, -25 ≤ l ≤ 25
Reflections collected	26228
Independent reflections	6243 [Rint = 0.0421, Rsigma = 0.0301]
Data/restraints/parameters	6243/2/495
Goodness-of-fit on F^2	1.049
Final R indexes [I>=2σ (I)]	R1 = 0.0781, wR2 = 0.2177
Final R indexes [all data]	R1 = 0.0866, wR2 = 0.2296
Largest diff. peak/hole / e Å^{-3}	0.80/-0.51

Table S7. X-ray diffraction analysis of compound **10**.

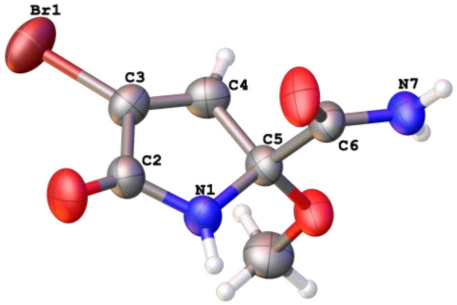

Empirical formula	$C_6H_7BrN_2O_3$
Formula weight	235.05
Temperature/K	293(2)
Crystal system	monoclinic
Space group	P21/c
a/Å	9.8547(3)
b/Å	8.4945(2)
c/Å	10.1134(3)
α/°	90
β/°	92.843(3)
γ/°	90
Volume/Å^3	845.56(4)
Z	4
ρ_{calc}g/cm^3	1.846
μ/mm^{-1}	6.435
F(000)	464.0
Crystal size/mm^3	0.31 × 0.25 × 0.17
Radiation	CuKα (λ = 1.54184)
2Θ range for data collection/°	8.984 to 156.222
Index ranges	-12 ≤ h ≤ 11, -4 ≤ k ≤ 10, -12 ≤ l ≤ 10
Reflections collected	5155
Independent reflections	1706 [Rint = 0.0967, Rsigma = 0.0604]
Data/restraints/parameters	1706/0/110
Goodness-of-fit on F^2	1.026
Final R indexes [I>=2σ (I)]	R1 = 0.0928, wR2 = 0.2292
Final R indexes [all data]	R1 = 0.0961, wR2 = 0.2374
Largest diff. peak/hole / e Å^{-3}	1.24/-1.86

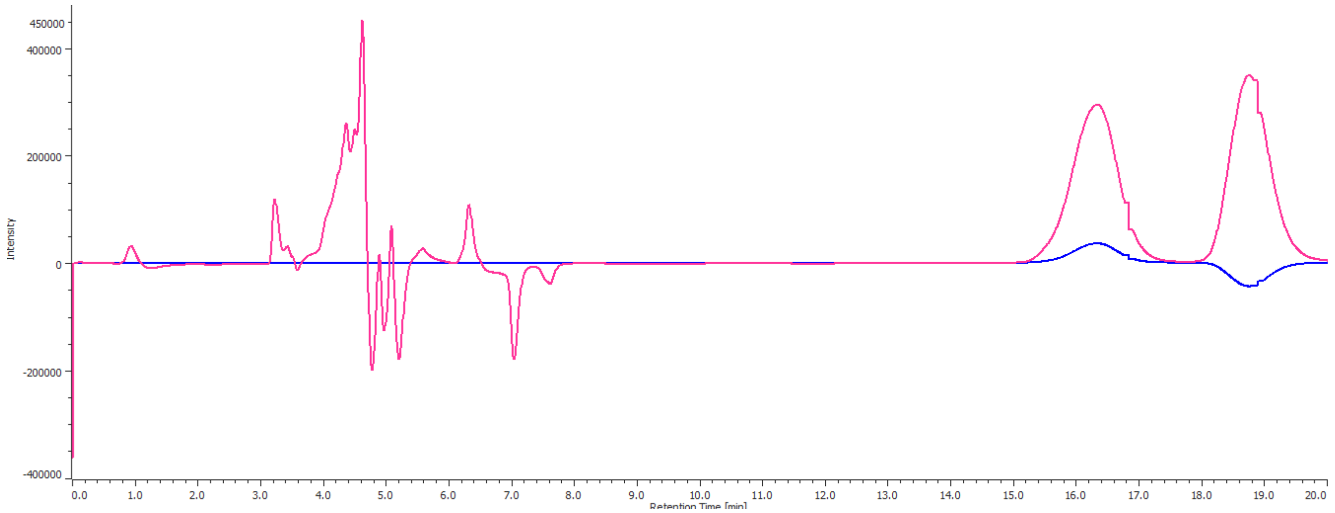

Figure S1. The LC-CD analysis figure of compounds **1a** (R_f=16.2 min), **1b** (R_f=18.8 min) on chiral Daicel Chiralpack AD-H column (250 × 4.6 mm, 5 μm) under the HPLC condition of n-hexane/ isopropanol (75:25) solution.

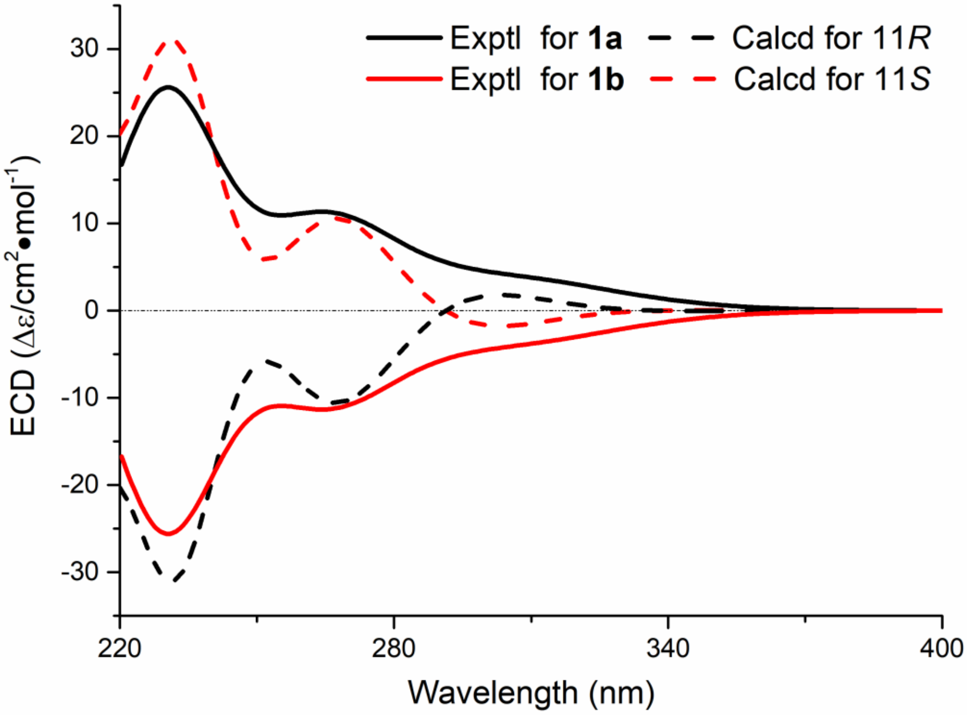

Figure S2. Calculated and experimental ECD spectra of compounds **1a** and **1b**.

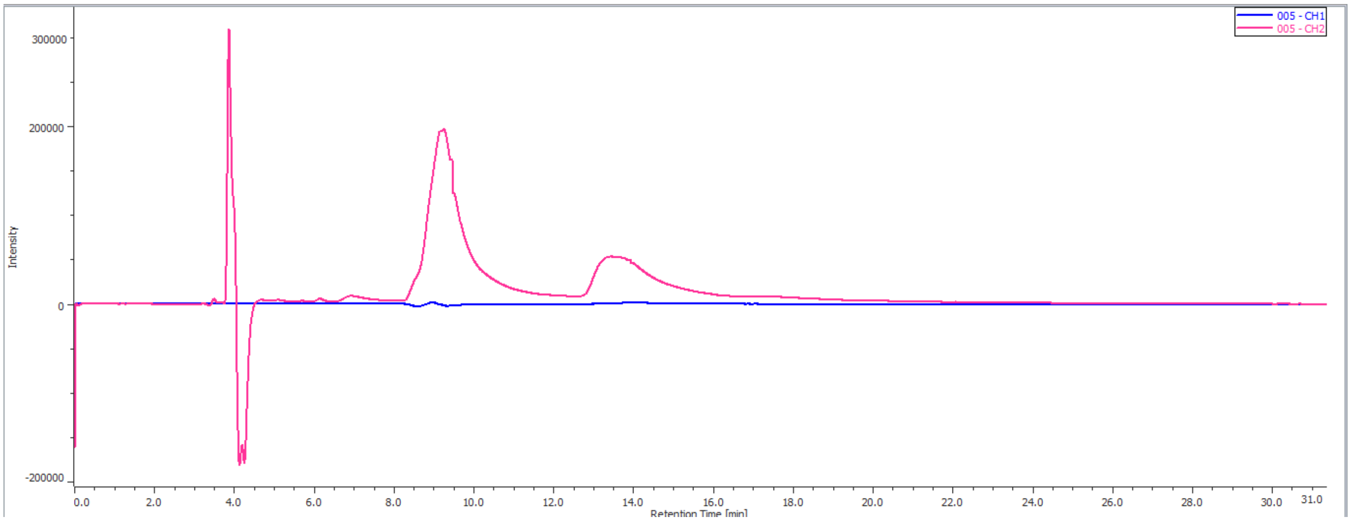

Figure S3. The LC-CD analysis figure of compounds **2a** (R_f=9.2 min), **2b** (R_f=13.5 min) on chiral Daicel Chiralpack AD-H column (250 × 4.6 mm, 5 μm) under the HPLC condition of n-hexane/ isopropanol (65:35) solution.

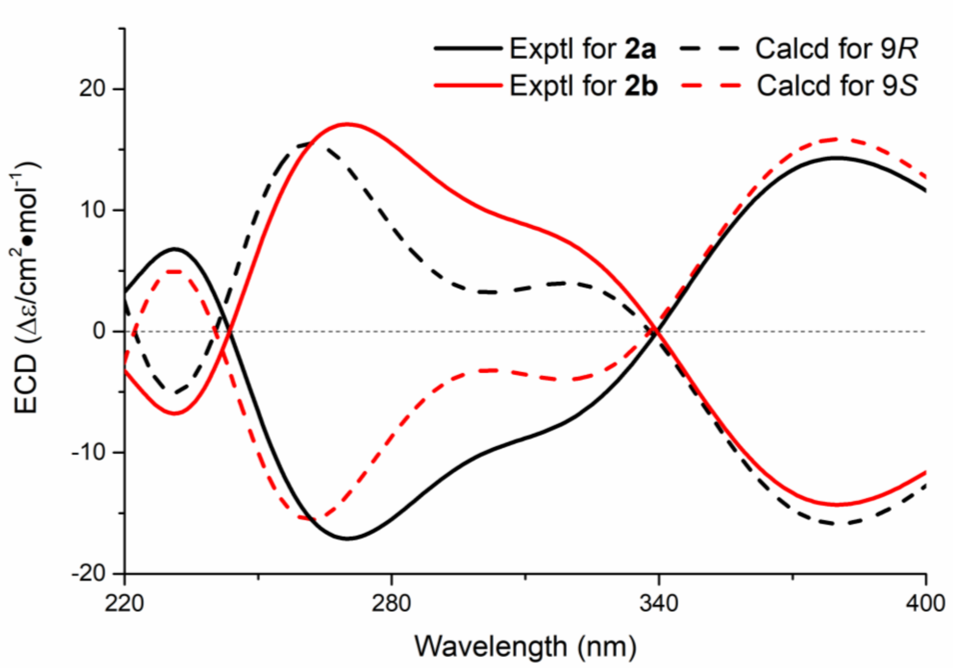

Figure S4. Calculated and experimental ECD spectra of compounds **2a** and **2b**.

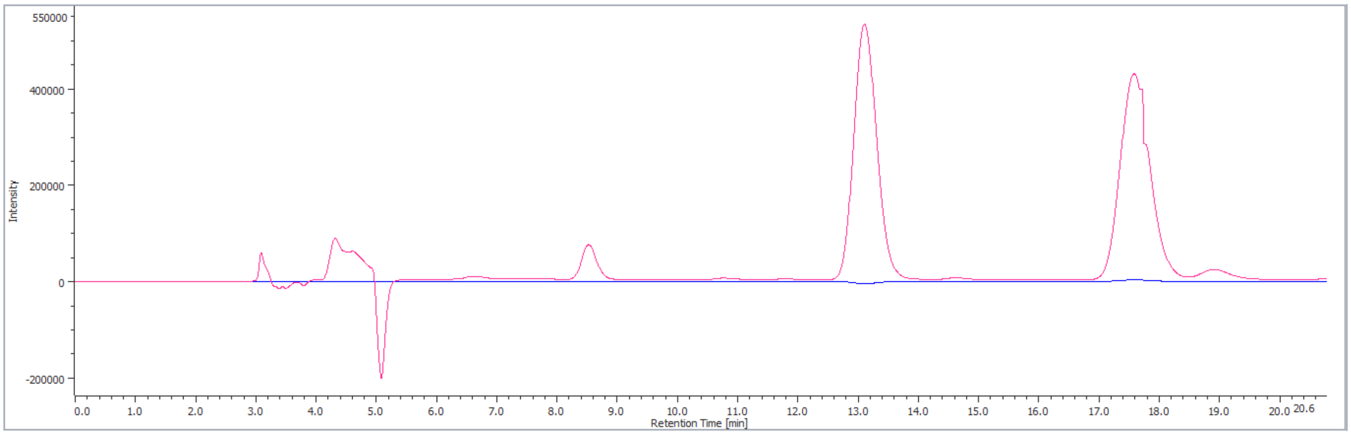

Figure S5. The LC-CD analysis figure of compounds **3a** (R_f=13.1 min), **3b** (R_f=17.6 min) on chiral Daicel Chiralpack AD-H column (250 × 4.6 mm, 5 μm) under the HPLC condition of n-hexane/ isopropanol (90:10) solution.

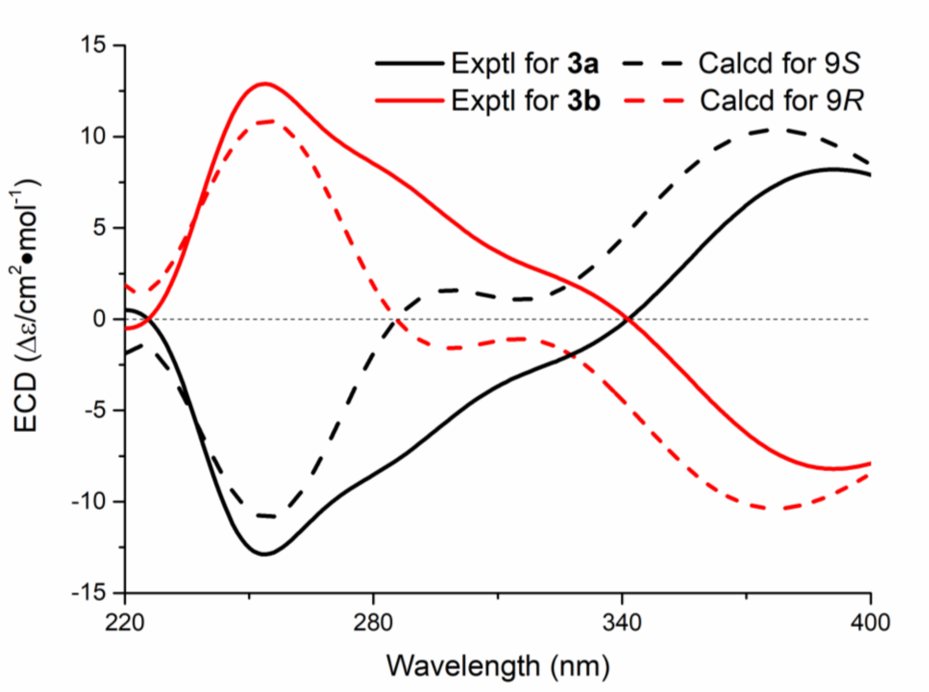

Figure S6. Calculated and experimental ECD spectra of compounds **3a** and **3b**.

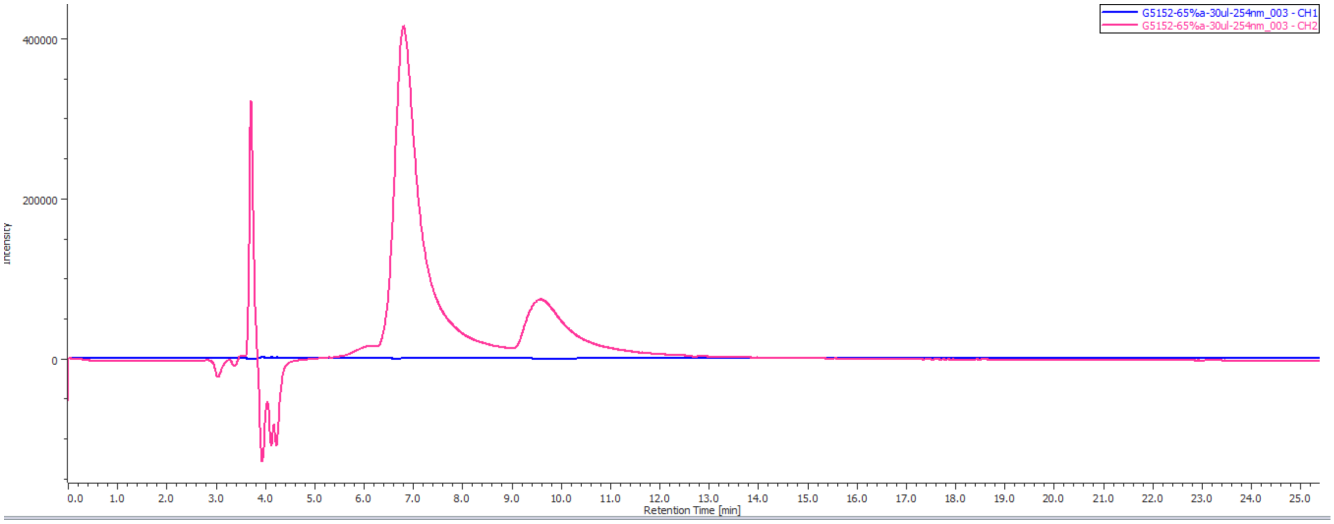

Figure S7. The LC-CD analysis figure of compounds **4a** (R_f=6.8 min), **4b** (R_f=9.6 min) on chiral Daicel Chiralpack AD-H column (250 × 4.6 mm, 5 μm) under the HPLC condition of n-hexane/ isopropanol (65:35) solution.

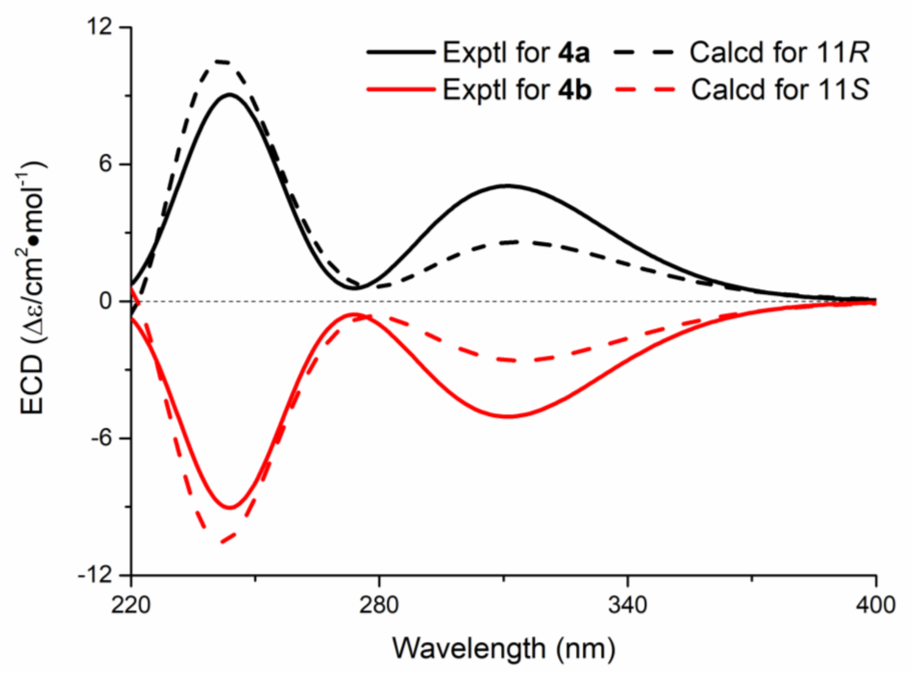

Figure S8. Calculated and experimental ECD spectra of compounds **4a** and **4b**.

Functional		Solvent?		Basis Set		Type of Data	
B3LYP		PCM		6-311+G(d,p)		Unscaled Shifts	
		DP4+	0.00%	100.00%	0.00%	0.00%	-
Nuclei	sp2?	xperimenta	Isomer 1	Isomer 2	Isomer 3	Isomer 4	Isomer 5
C		19.7	27.59	25.50	26.09	25.66	
C		39.5	35.56	47.03	42.95	37.60	
C		45	51.41	49.54	48.60	47.91	
C		50.6	51.91	53.95	56.95	53.91	
C		63.9	79.12	70.24	78.50	77.12	
C		81.3	96.07	100.50	101.41	98.46	
C		89.7	99.07	95.95	94.28	100.98	
C	x	120.3	146.74	146.56	149.06	149.78	
C	x	144.2	162.61	157.12	154.44	157.88	
C	x	156.4	170.28	168.51	168.75	171.22	
C	x	161.9	185.46	172.47	173.68	173.31	
C	x	163.1	178.53	170.58	167.35	176.91	
H		1.98	2.48	1.96	1.95	0.99	
H		1.98	1.51	2.12	1.95	1.54	
H		2.25	2.12	2.01	2.09	1.95	
H		2.25	2.21	2.38	2.30	2.09	
H		3.15	3.57	3.61	3.51	3.35	
H		3.35	3.99	3.53	3.85	4.24	
H		5.8	4.69	5.78	5.31	5.31	
H	x	7.79	7.68	7.55	7.82	7.58	
H		3.15	3.39	3.42	3.60	3.44	

	A–B	C	D	E	F	G	H
1	Functional	Solvent?		Basis Set		Type of Data	
2	B3LYP	PCM		6-311+G(d,p)		Unscaled Shifts	
3							
4		Isomer 1	Isomer 2	Isomer 3	Isomer 4	Isomer 5	Isomer 6
5	sDP4+ (H data)	0.00%	99.93%	0.07%	0.00%	-	-
6	sDP4+ (C data)	0.70%	98.75%	0.00%	0.54%	-	-
7	sDP4+ (all data)	0.00%	100.00%	0.00%	0.00%	-	-
8	uDP4+ (H data)	0.00%	83.90%	16.10%	0.00%	-	-
9	uDP4+ (C data)	0.00%	100.00%	0.00%	0.00%	-	-
10	uDP4+ (all data)	0.00%	100.00%	0.00%	0.00%	-	-
11	DP4+ (H data)	0.00%	99.99%	0.01%	0.00%	-	-
12	DP4+ (C data)	0.00%	100.00%	0.00%	0.00%	-	-
13	DP4+ (all data)	0.00%	100.00%	0.00%	0.00%	-	-

Figure S9. Detailed DP4+ probability (calculated at PCM/b3lyp/6-311+G(d,p) level) for compounds **6** and **16** (Isomer 1: 2*R*6*R*10*R*, Isomer 2: 2*R*6*R*10*S*, Isomer 3: 2*R*6*S*10*R*, Isomer 4: 2*R*6*S*10*S*).

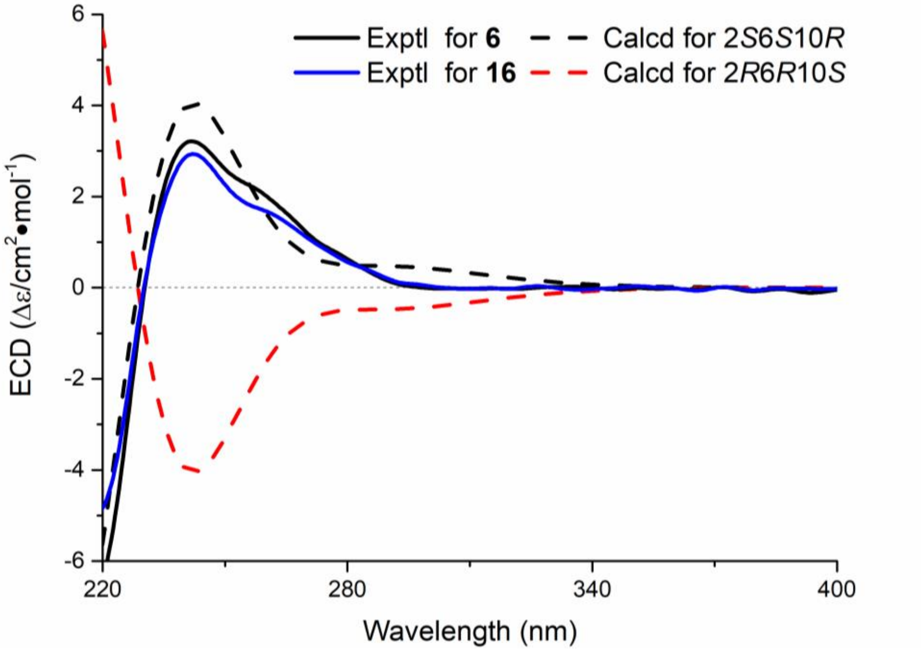

Figure S10. Calculated and experimental ECD spectra of compounds **6** and **16**.

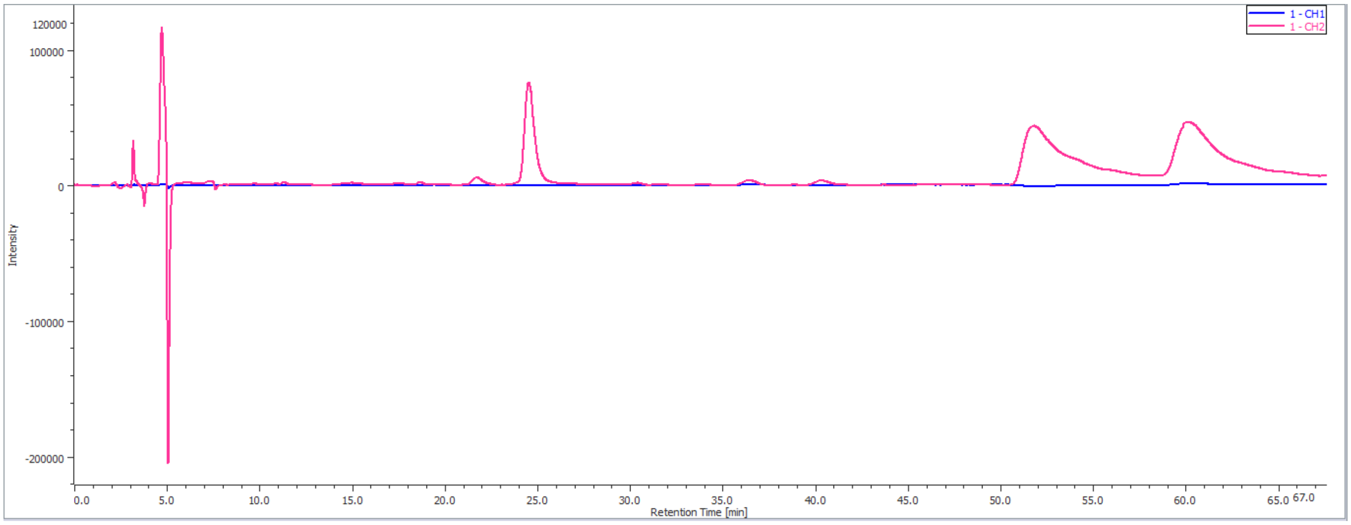

Figure S11. The LC-CD analysis figure of compounds **7a** (R_f=52.2 min), **7b** (R_f=60.5 min) on chiral Daicel Chiralpack AD-H column (250 × 4.6 mm, 5 μm) under the HPLC condition of n-hexane/ isopropanol (90:10) solution.

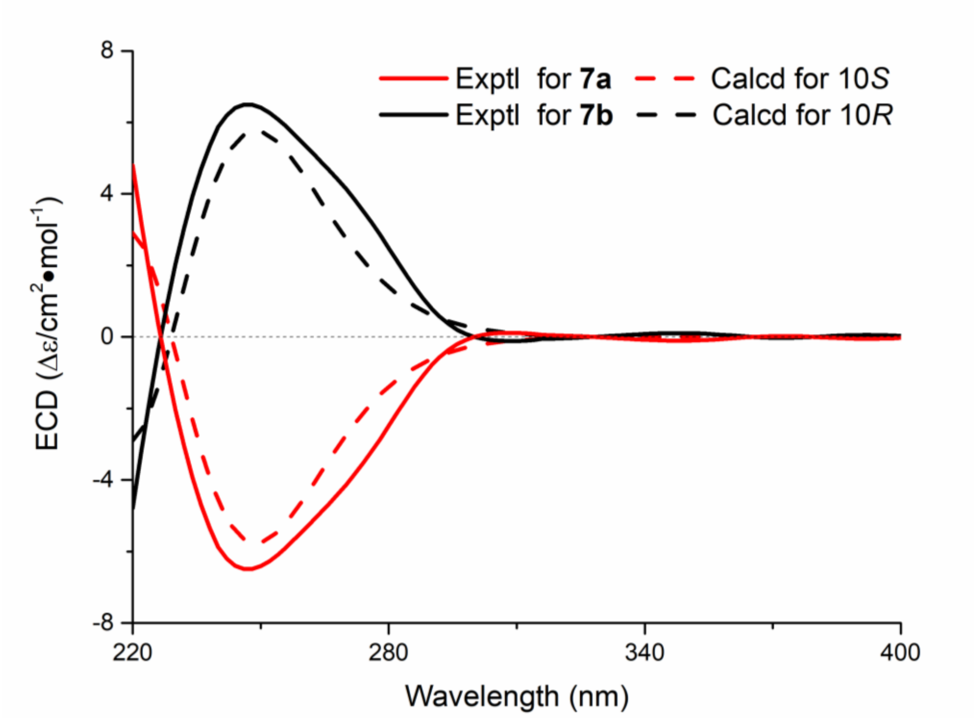

Figure S12. Calculated and experimental ECD spectra of compounds **7a** and **7b**.

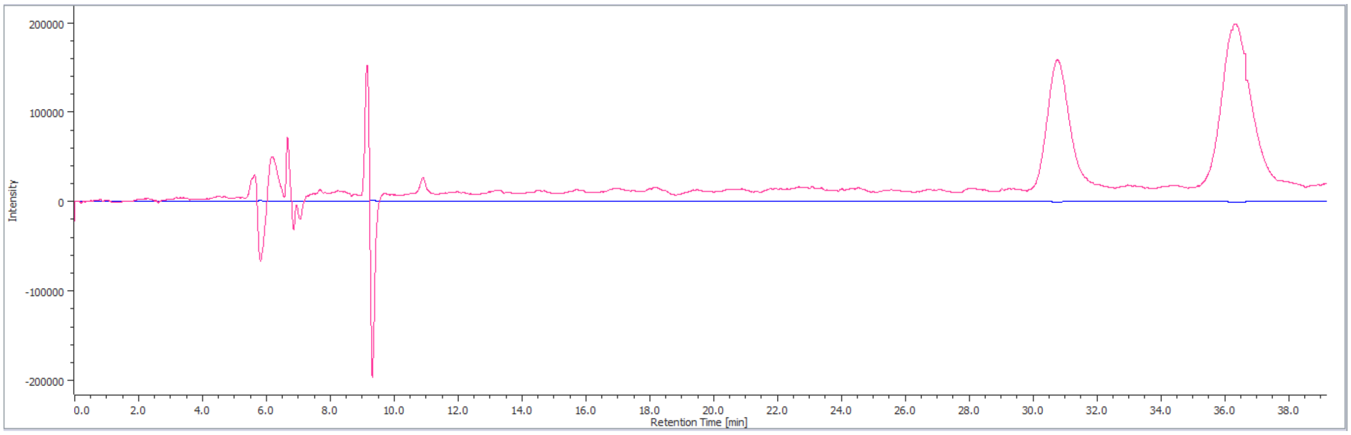

Figure S13. The LC-CD analysis figure of compounds **8a** (R_f=30.6 min), **8b** (R_f=36.2 min) on chiral Daicel Chiralpack AD-H column (250 × 4.6 mm, 5 μm) under the HPLC condition of n-hexane/ isopropanol (90:10) solution.

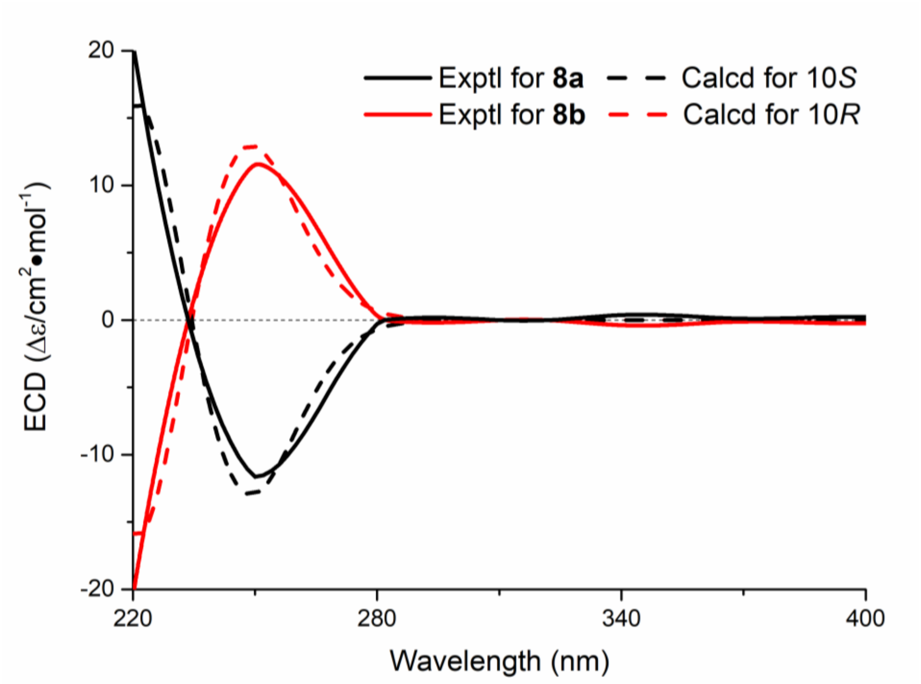

Figure S14. Calculated and experimental ECD spectra of compounds **8a** and **8b**.

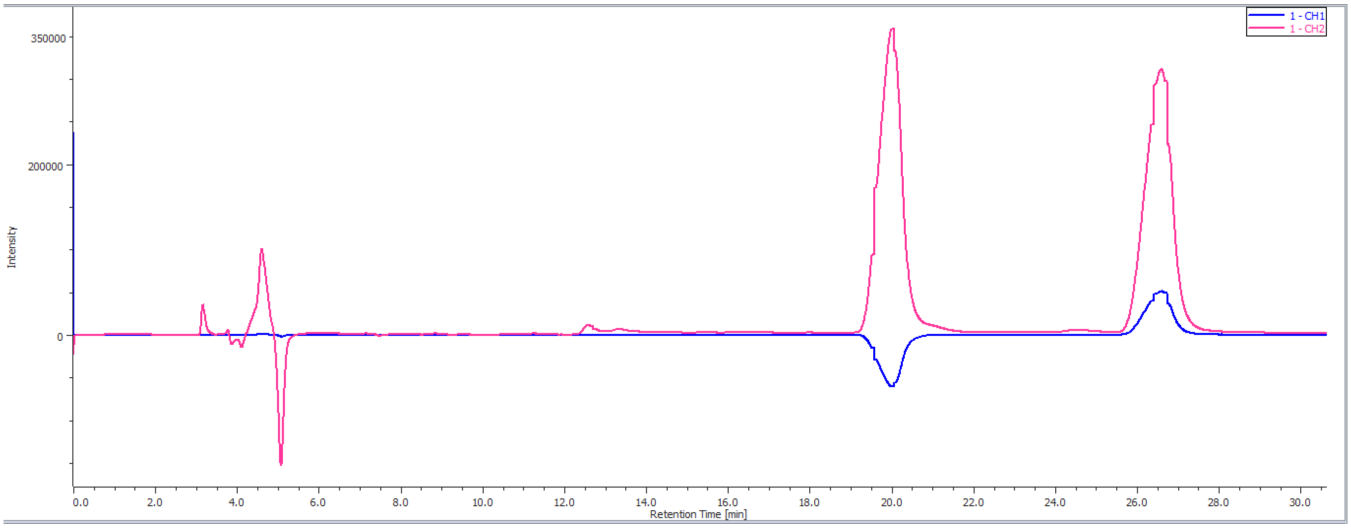

Figure S15. The LC-CD analysis figure of compounds **10a** (R_f=19.8 min), **10b** (R_f=26.6 min) on chiral Daicel Chiralpack AD-H column (250 × 4.6 mm, 5 μm) under the HPLC condition of n-hexane/ isopropanol (90:10) solution.

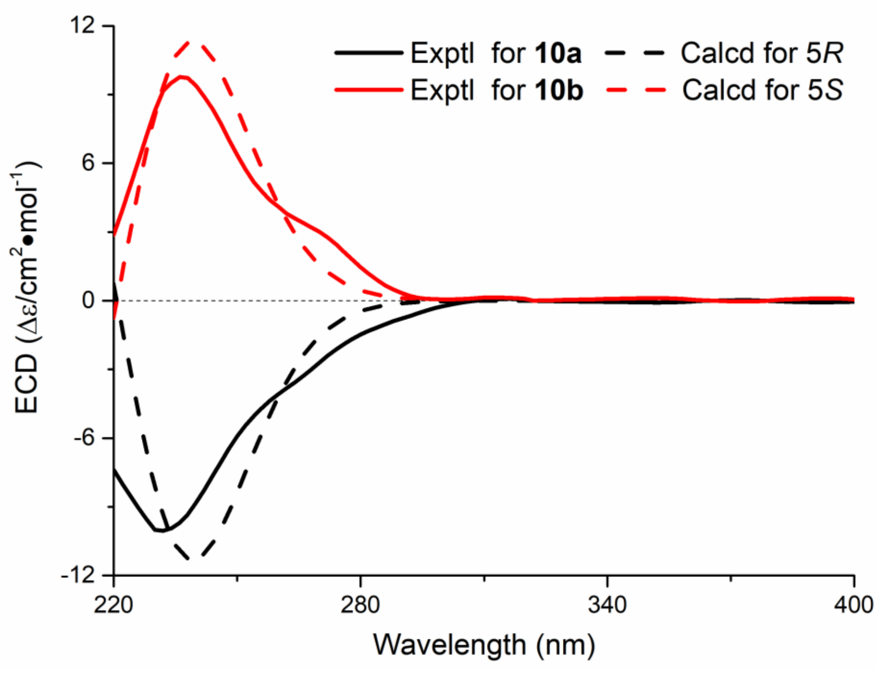

Figure S16. Calculated and experimental ECD spectra of compounds **10a** and **10b**.

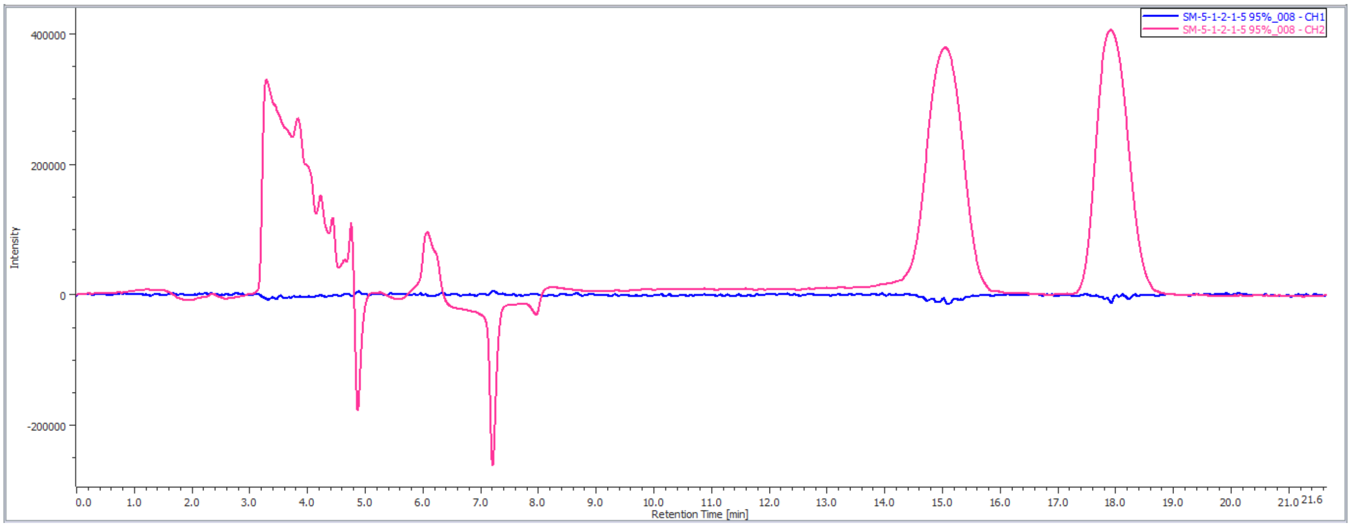

Figure S17. The LC-CD analysis figure of compounds **11a** (R_f=14.9 min), **11b** (R_f=17.8 min) on chiral Daicel Chiralpack AD-H column (250 × 4.6 mm, 5 μm) under the HPLC condition of n-hexane/ isopropanol (90:10) solution.

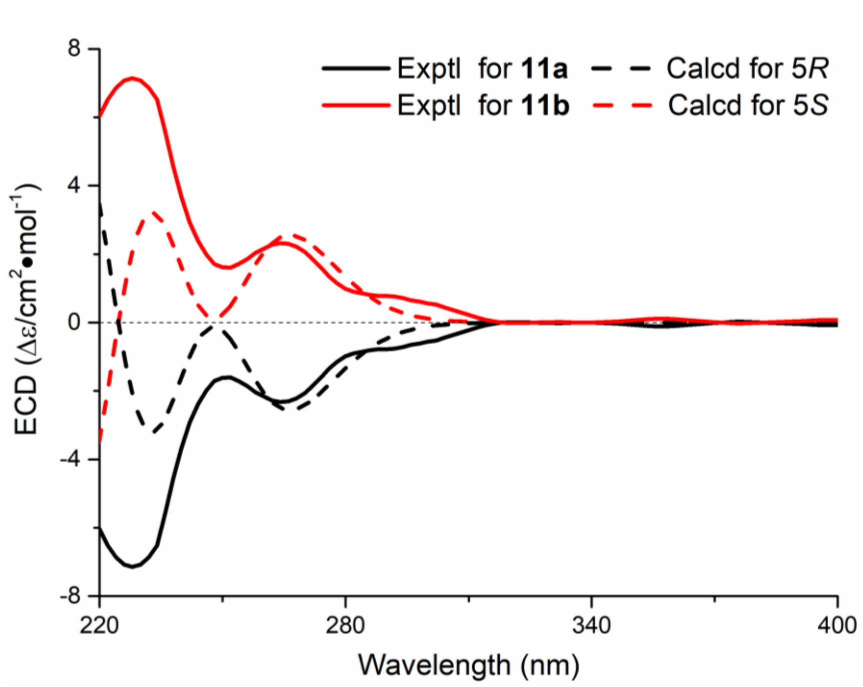

Figure S18. Calculated and experimental ECD spectra of compounds **11a** and **11b**.

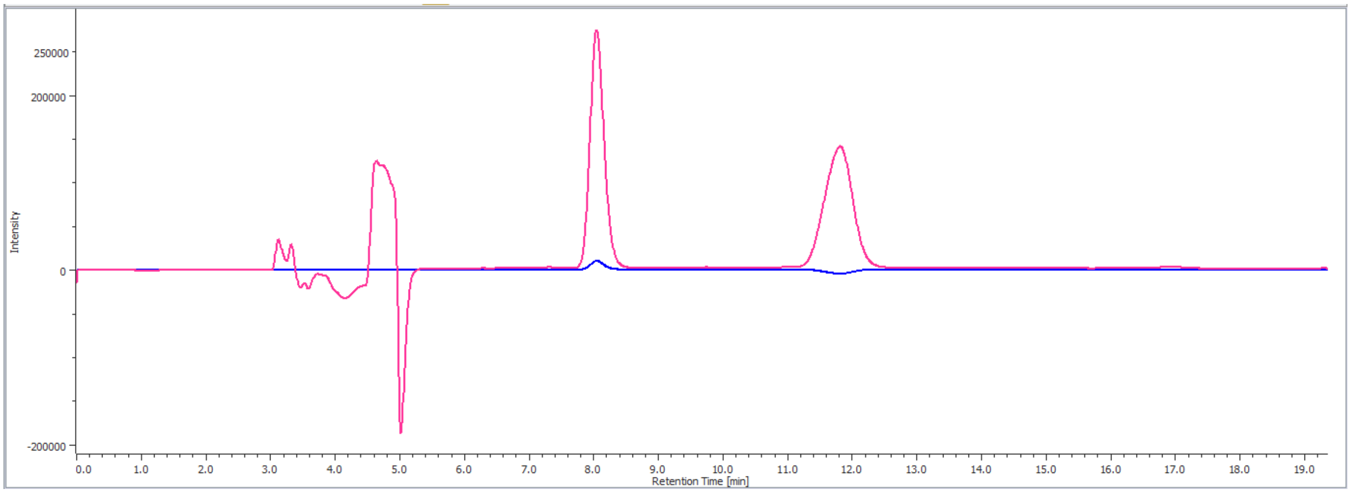

Figure S19. The LC-CD analysis figure of compounds **12a** (R_f=8.1 min), **12b** (R_f=11.8 min) on chiral Daicel Chiralpack AD-H column (250 × 4.6 mm, 5 μm) under the HPLC condition of n-hexane/ isopropanol (80:20) solution.

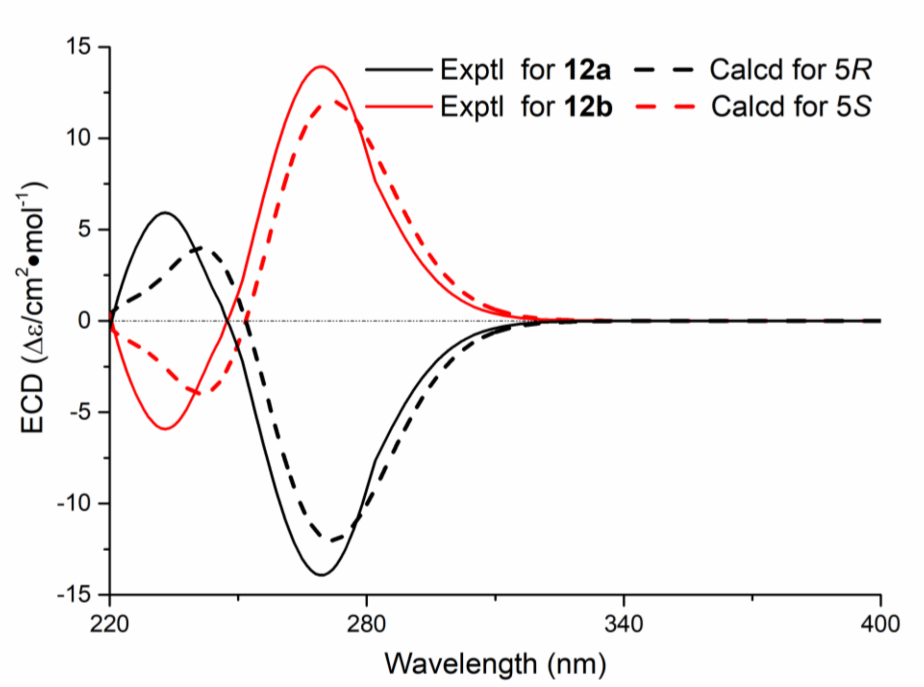

Figure S20. Calculated and experimental ECD spectra of compounds **12a** and **12b**.

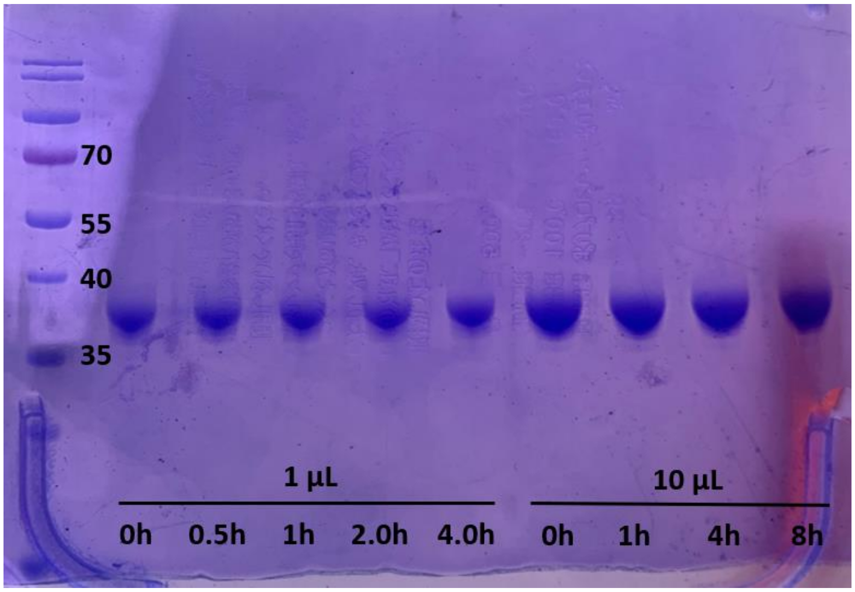

Figure S21. SDS PAGE of the ALR2 stability investigation for surface plasmon resonance (SPR) experiment during 0-8h at room temperature.

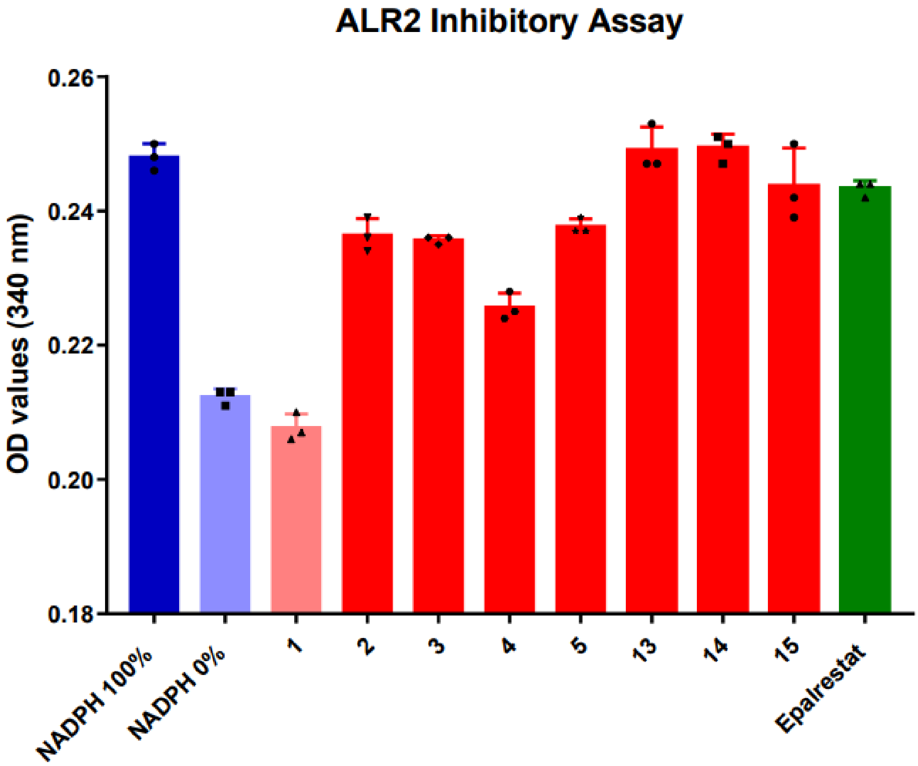

Figure S22. Compounds **2-5** and **13-15**, representative of 5/7/5 tricyclic spogiacidin-type PIA compounds, displayed the superior activities (>50% inhibition rate) at 20 μM.

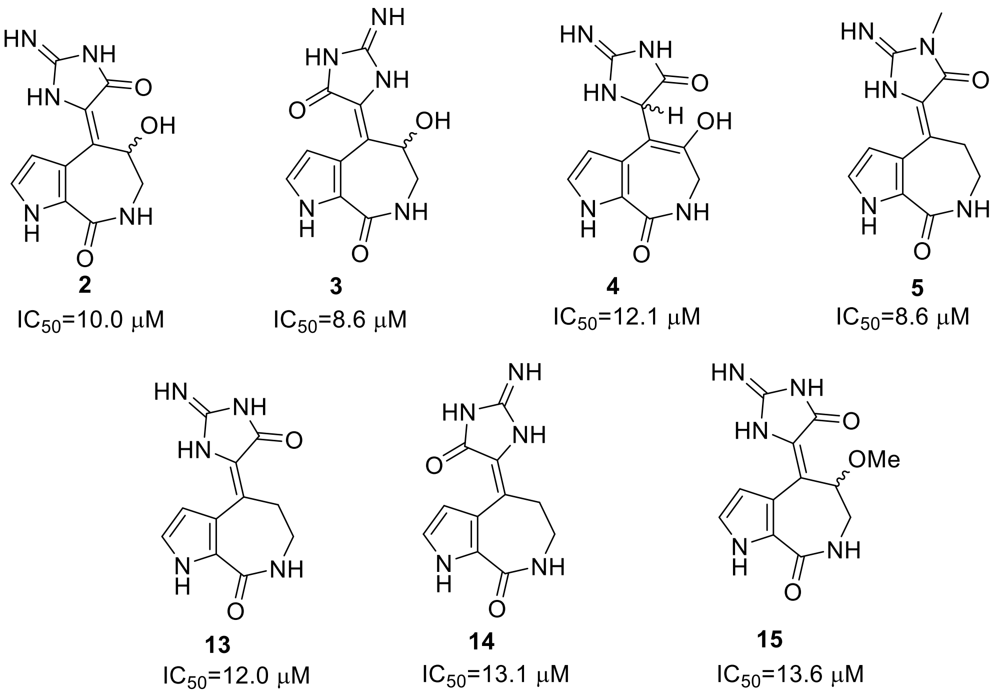

Figure S23. IC_{50} values of ALR2 inhibitory activities for spongiacidin-type PIAs (compounds **2-5** and **13-15**).

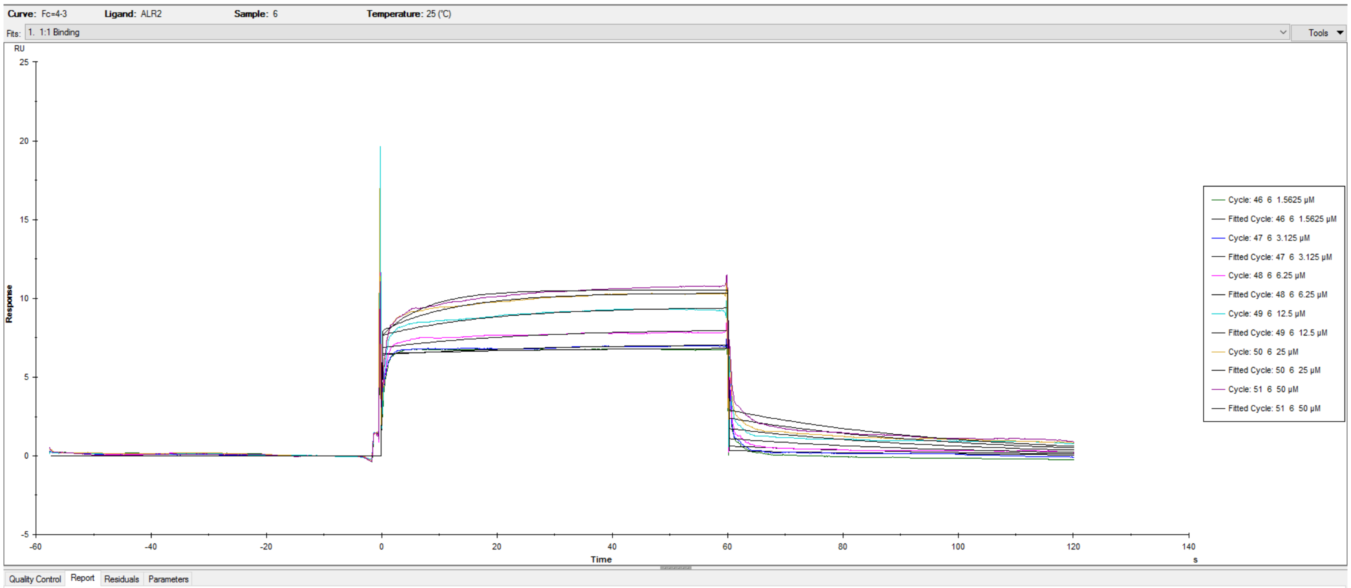

Curve	ka (1/Ms)	kd (1/s)	KD (M)	Rmax (RU)	Conc (M)	tc	Flow (ul/min)	kt (RU/Ms)	RI (RU)	Chi² (RU²)	U-value
	2081	0.02593	1.246E-5	3.646		1.327E+10				0.266	12
Cycle: 46 1.5625 μM					1.563E-6		30.00	4.123E+10	6.493		
Cycle: 47 3.125 μM					3.125E-6		30.00	4.123E+10	6.420		
Cycle: 48 6.25 μM					6.250E-6		30.00	4.123E+10	6.870		
Cycle: 49 12.5 μM					1.250E-5		30.00	4.123E+10	7.647		
Cycle: 50 25 μM					2.500E-5		30.00	4.123E+10	7.911		
Cycle: 51 50 μM					5.000E-5		30.00	4.123E+10	7.615		

Figure S24. SPR experiment of compound **13** using the Biacore T200 instrument.

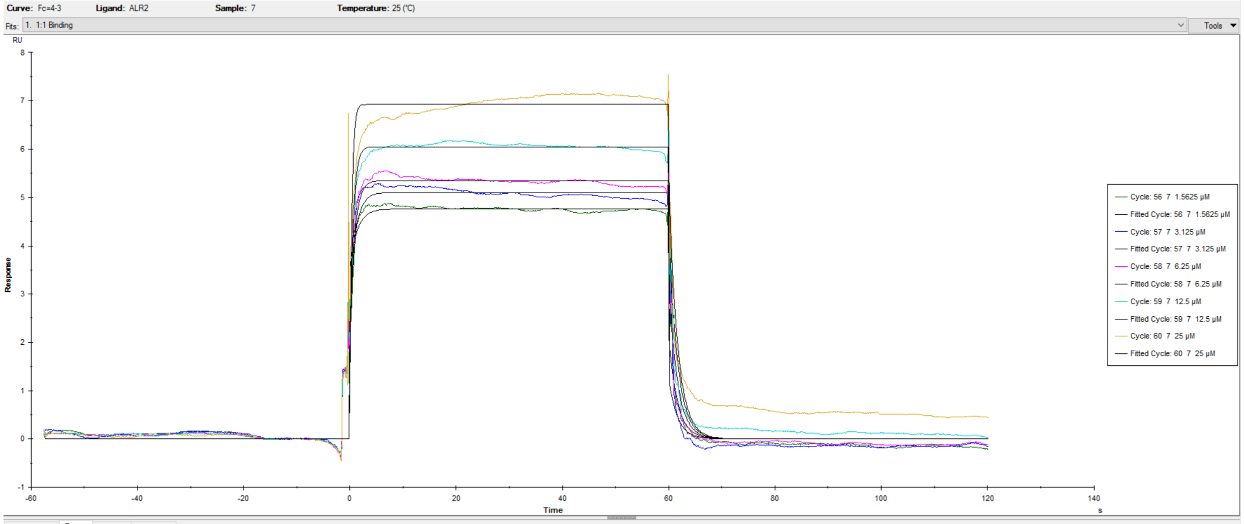

Quality Control | Report | Residuals | Parameters

Curve	ka (1/Ms)	kd (1/s)	KD (M)	Rmax (RU)	Conc (M)	tc	Flow (ul/min)	kt (RU/Ms)	RI (RU)	Chi² (RU²)	U-value
	8.480E+4	0.5839	6.886E-6	6.098		1.153E+13				0.0797	15
Cycle: 56 1.5625 µM					1.563E-6		30.00	3.583E+13	3.633		
Cycle: 57 3.125 µM					3.125E-6		30.00	3.583E+13	3.200		
Cycle: 58 6.25 µM					6.250E-6		30.00	3.583E+13	2.451		
Cycle: 59 12.5 µM					1.250E-5		30.00	3.583E+13	2.119		
Cycle: 60 25 µM					2.500E-5		30.00	3.583E+13	2.148		

Figure S25. SPR experiment of compound **14** using the Biacore T200 instrument.

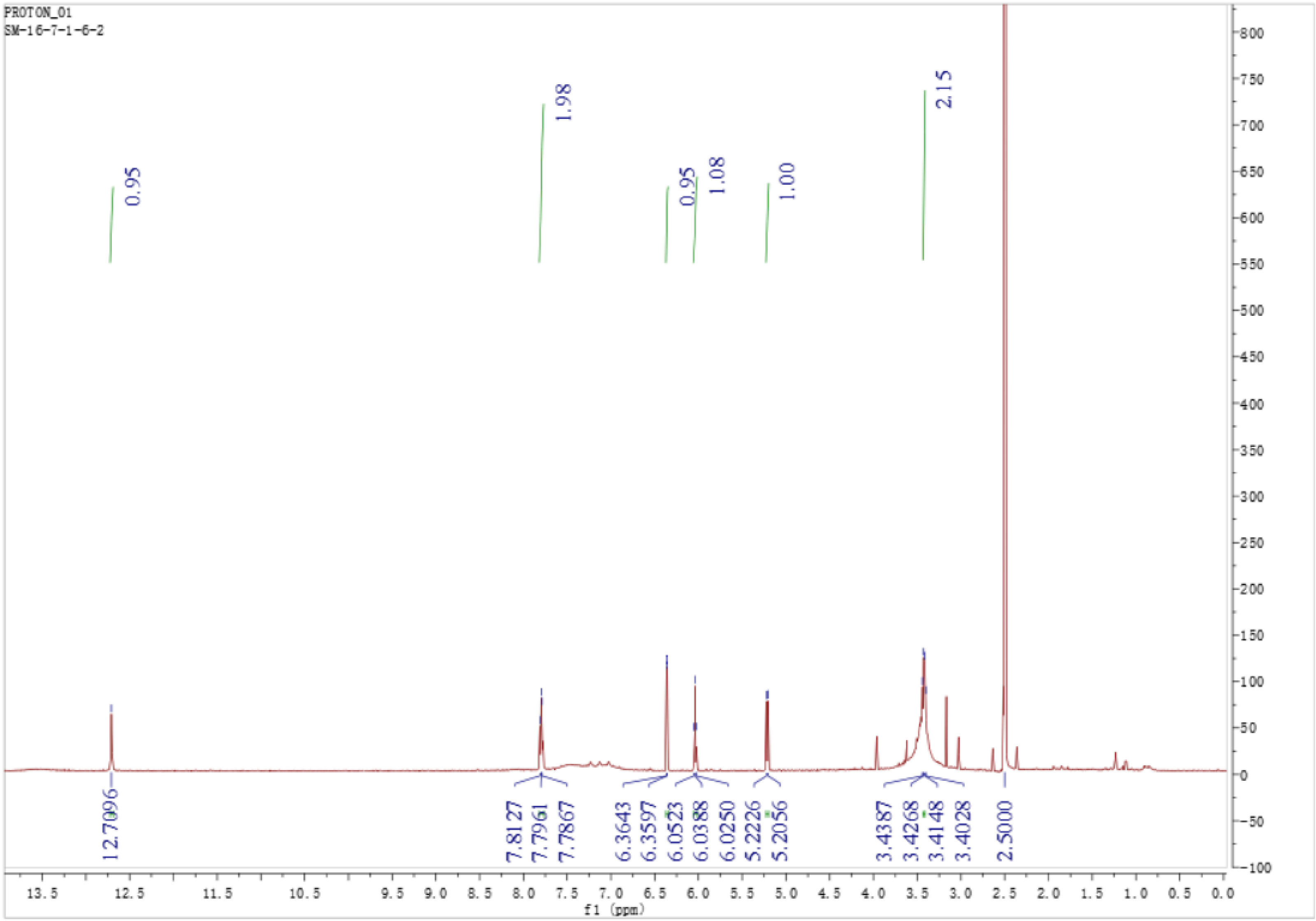

Figure S26. ^{1}H NMR spectrum of compound **1** in DMSO-d_6 (500 MHz).

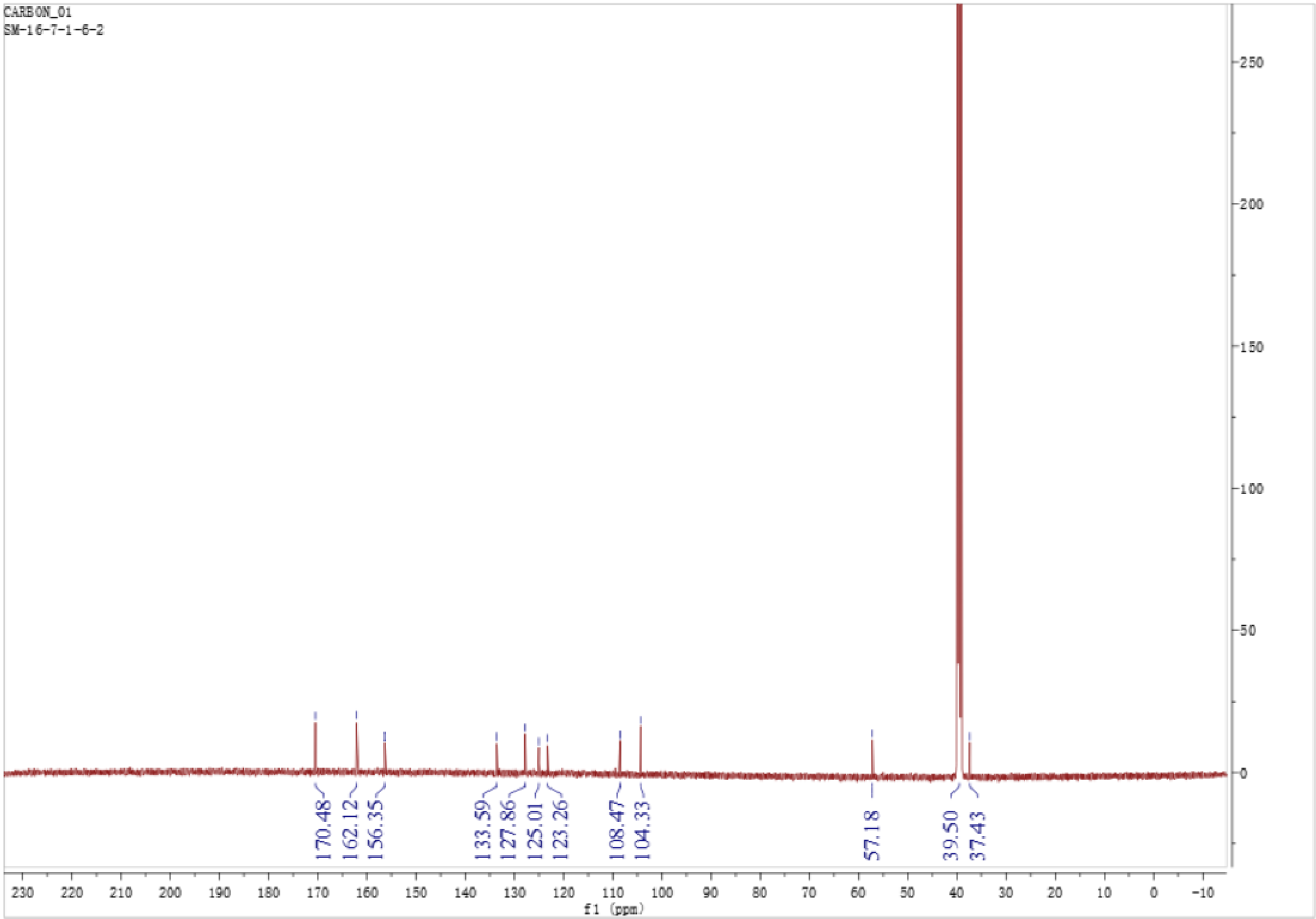

Figure S27. ^{13}C NMR spectrum of compound **1** in DMSO-d_6 (125 MHz).

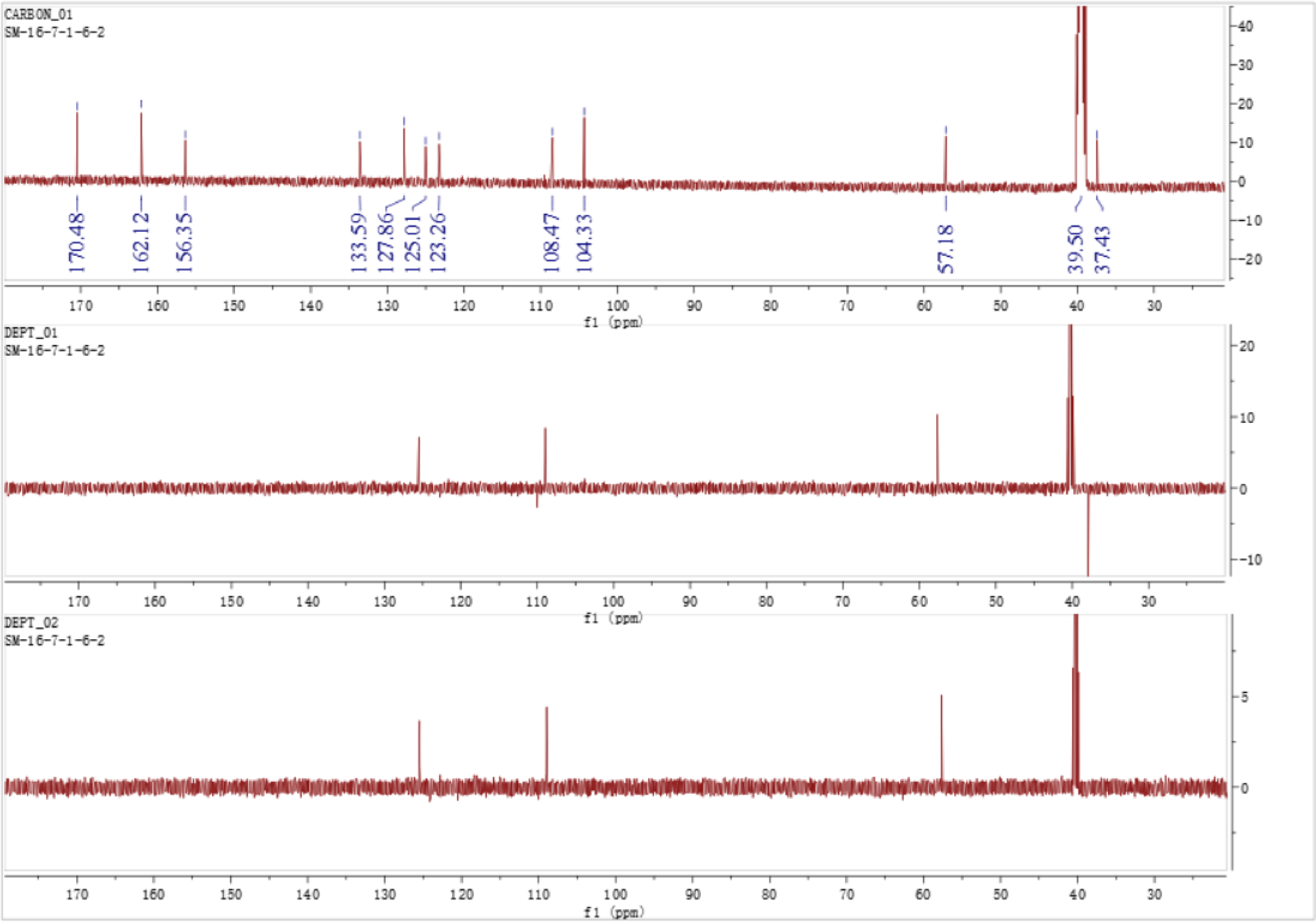

Figure S28. ^{13}C NMR and DEPT spectrum of compound **1** in DMSO-d_6 (125 MHz).

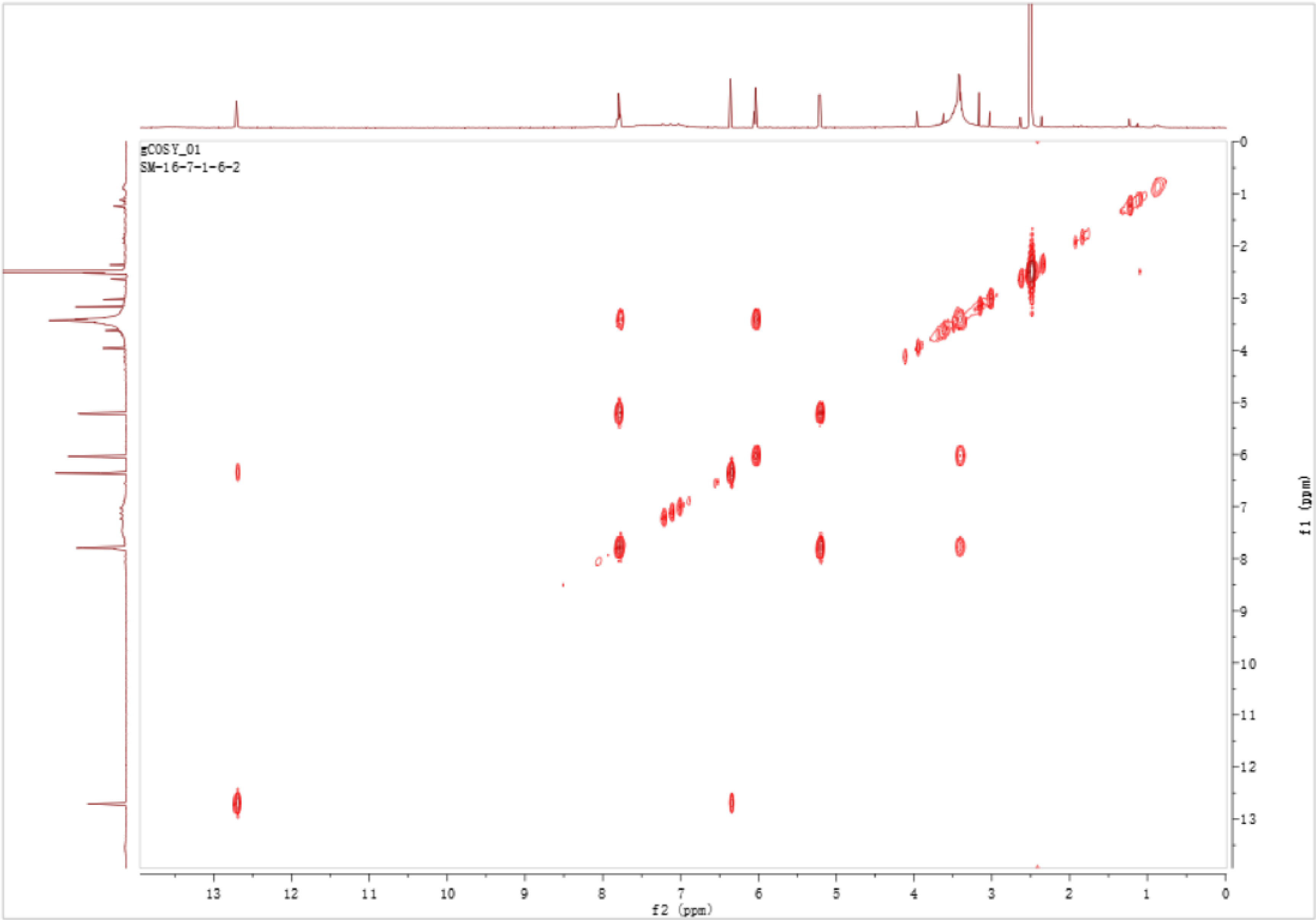

Figure S29. ^{1}H-^{1}H COSY spectrum of compound **1** in DMSO-d_6 (500 MHz).

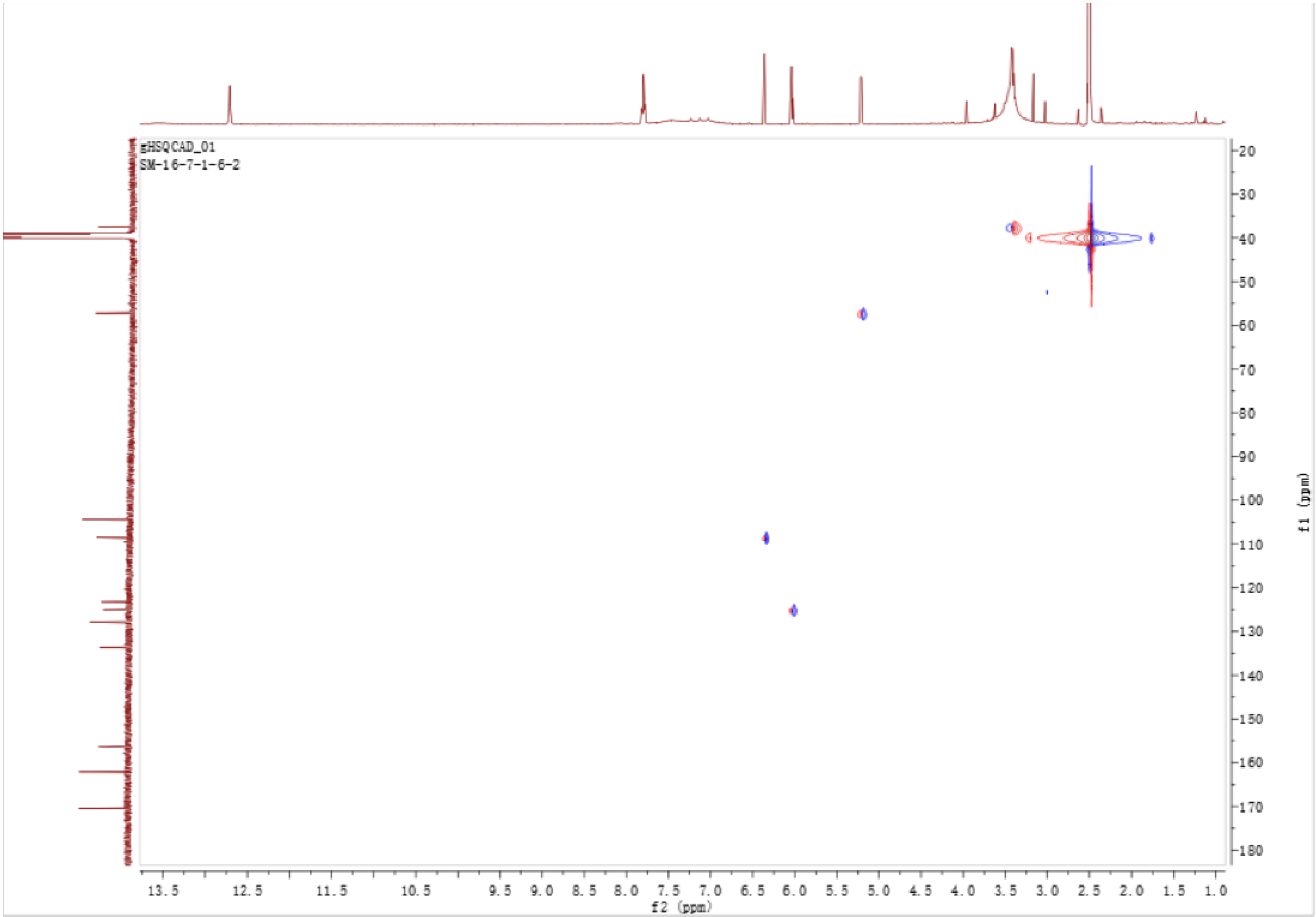

Figure S30. HSQC spectrum of compound **1** in DMSO-d_6 (500 MHz).

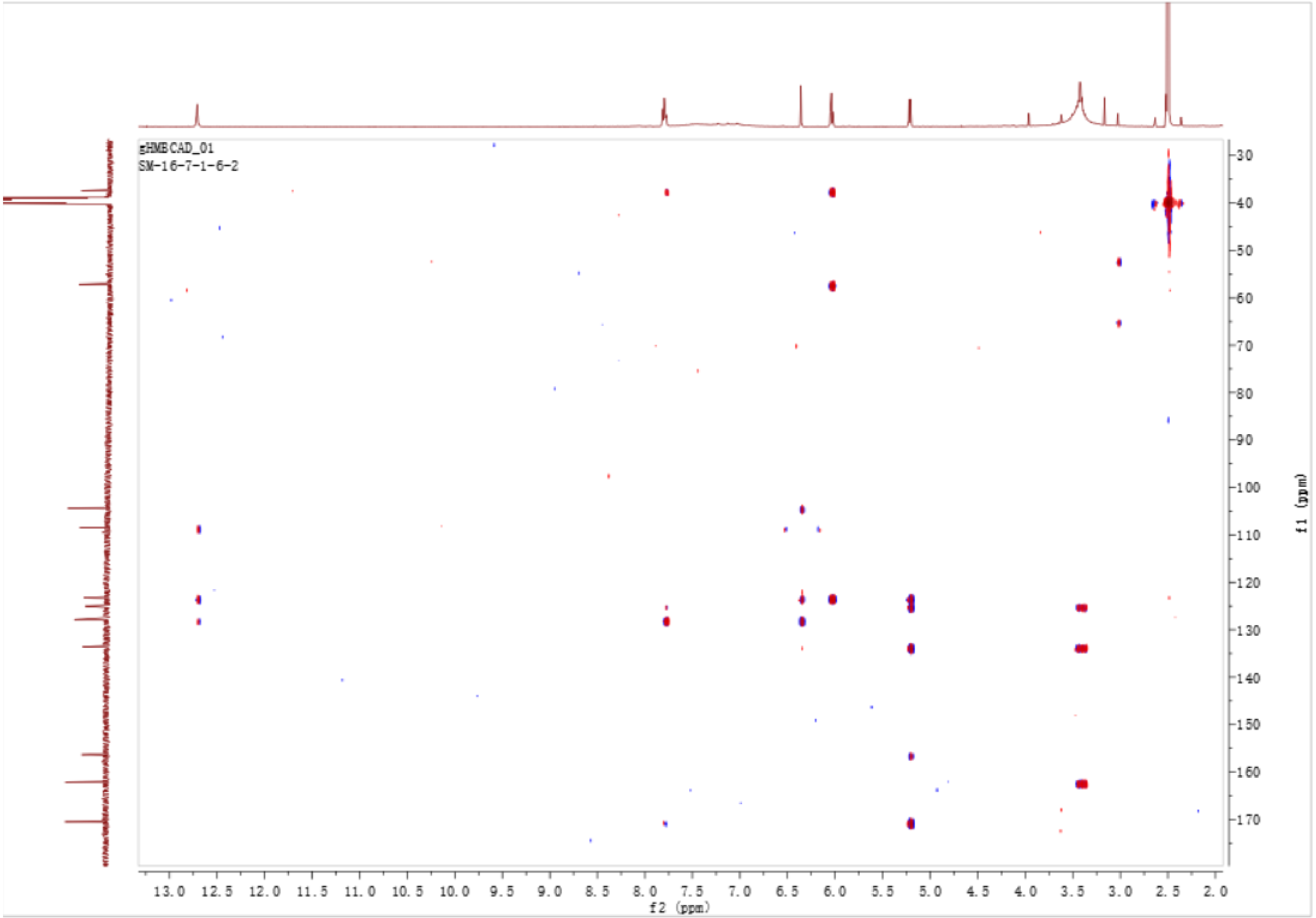

Figure S31. HMBC spectrum of compound **1** in DMSO-d_6 (500 MHz).

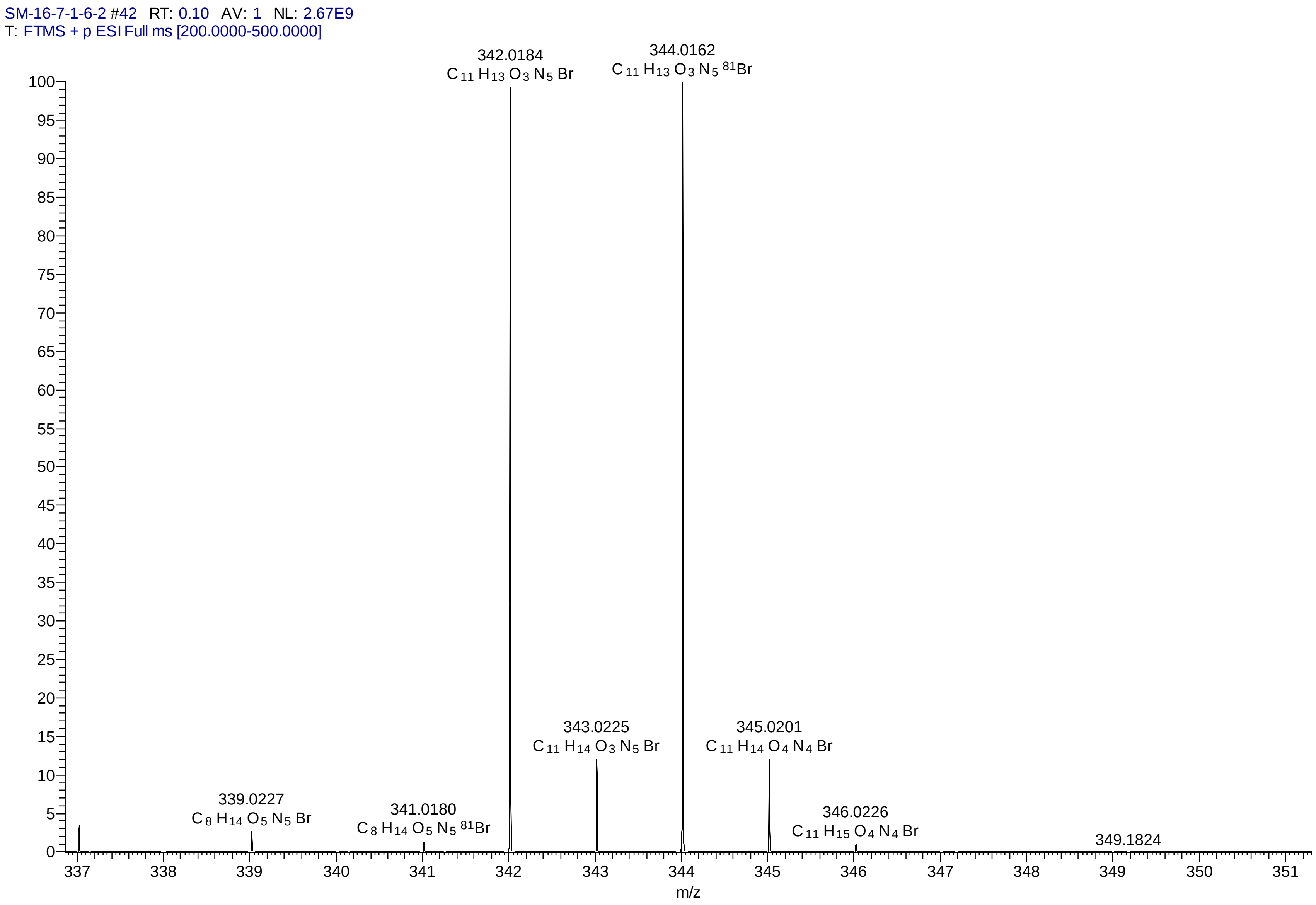

Figure S32. HRESIMS data of compound **1**.

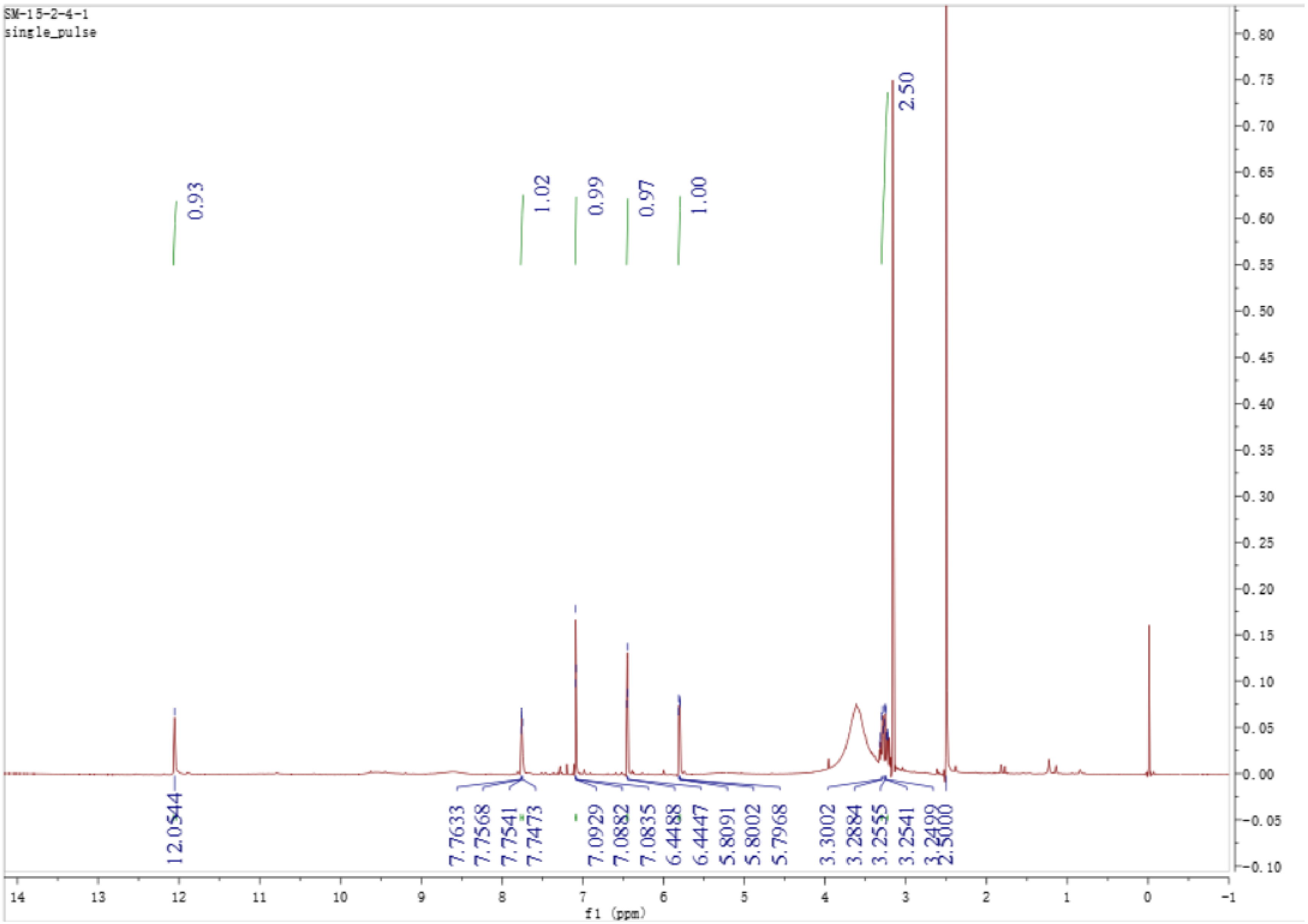

Figure S33. ^{1}H NMR spectrum of compound **2** in DMSO-d_6 (500 MHz).

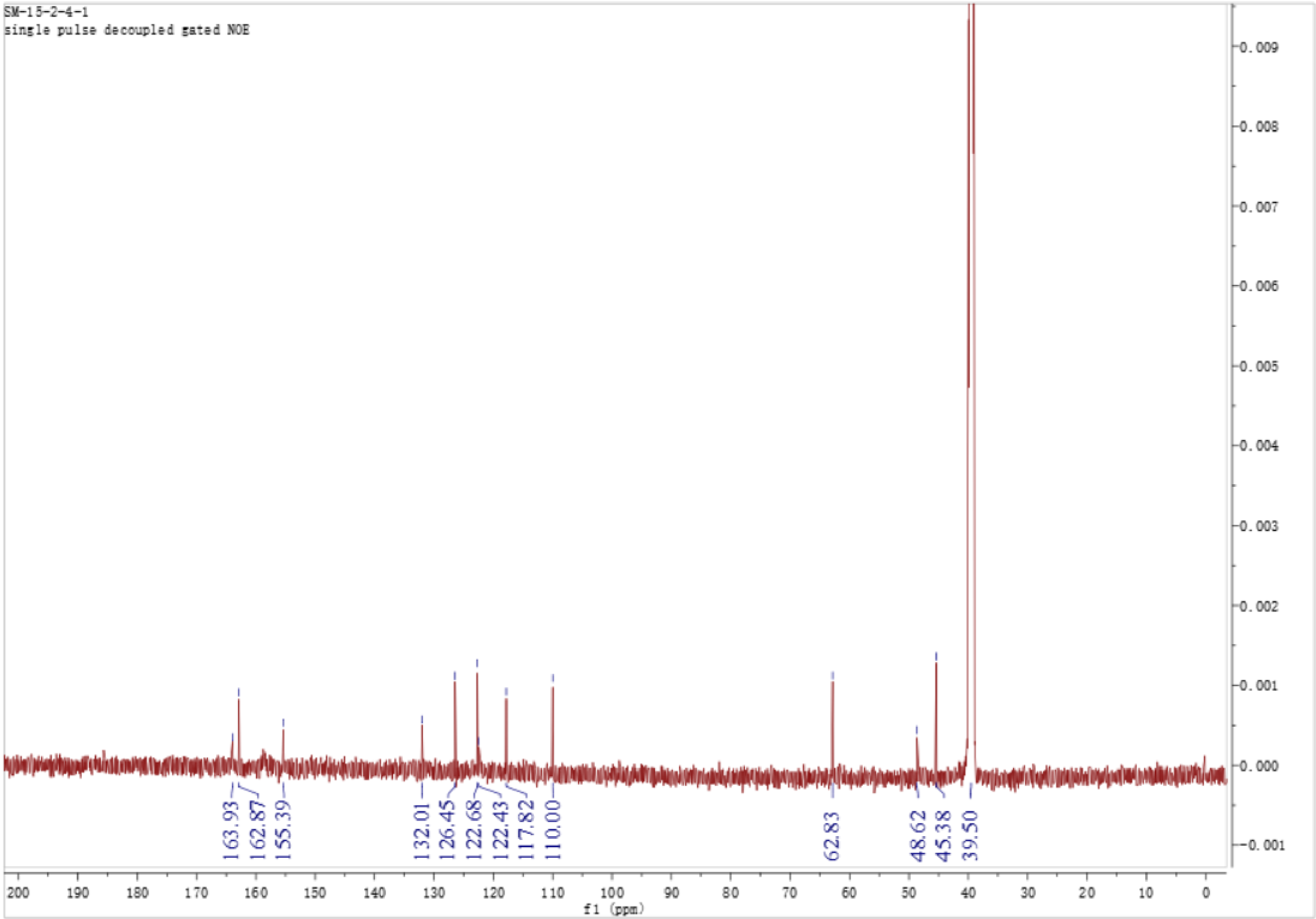

Figure S34. ^{13}C NMR spectrum of compound **2** in DMSO-d_6 (125 MHz).

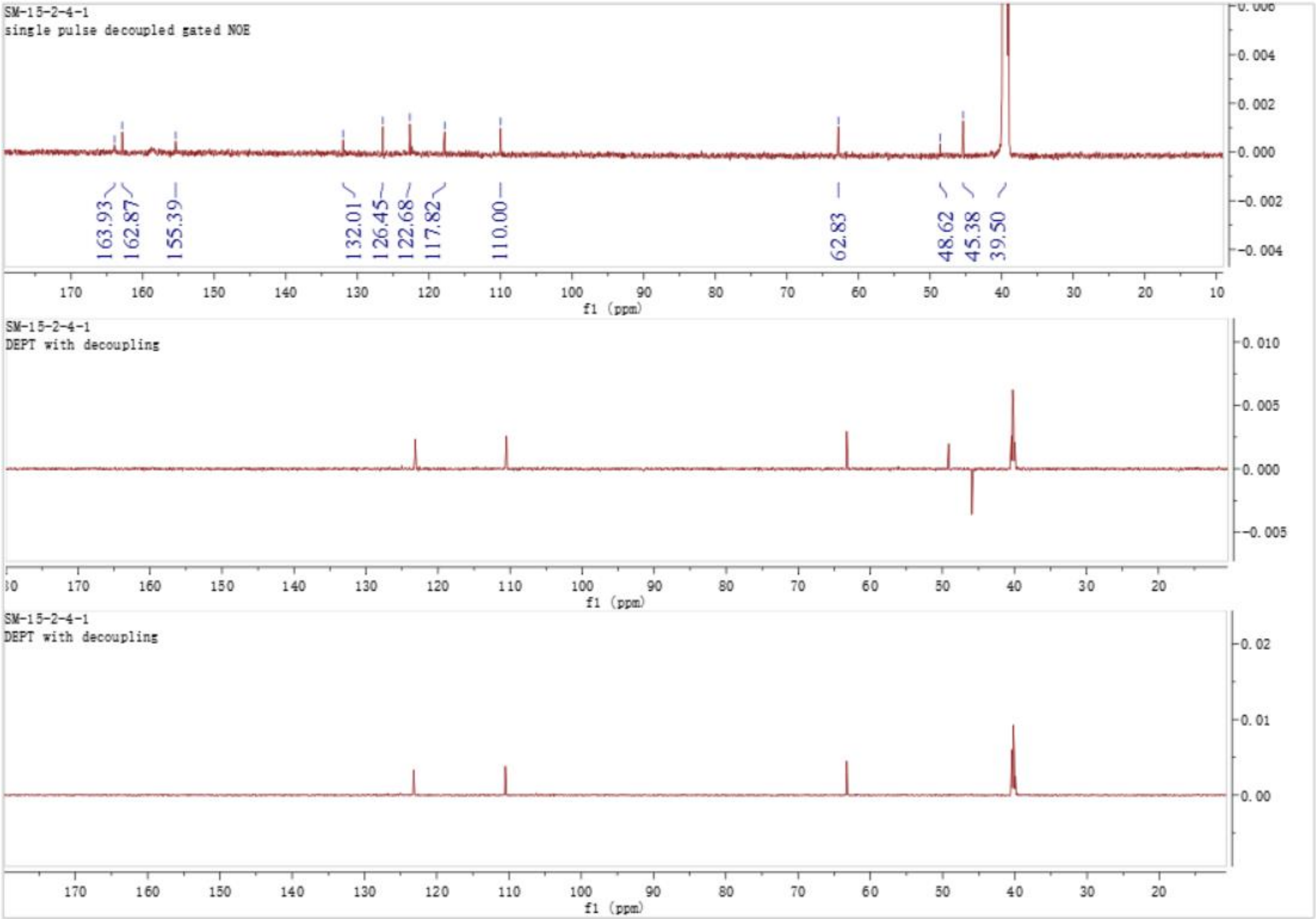

Figure S35. ^{13}C NMR and DEPT spectrum of compound **2** in DMSO-d_6 (125 MHz).

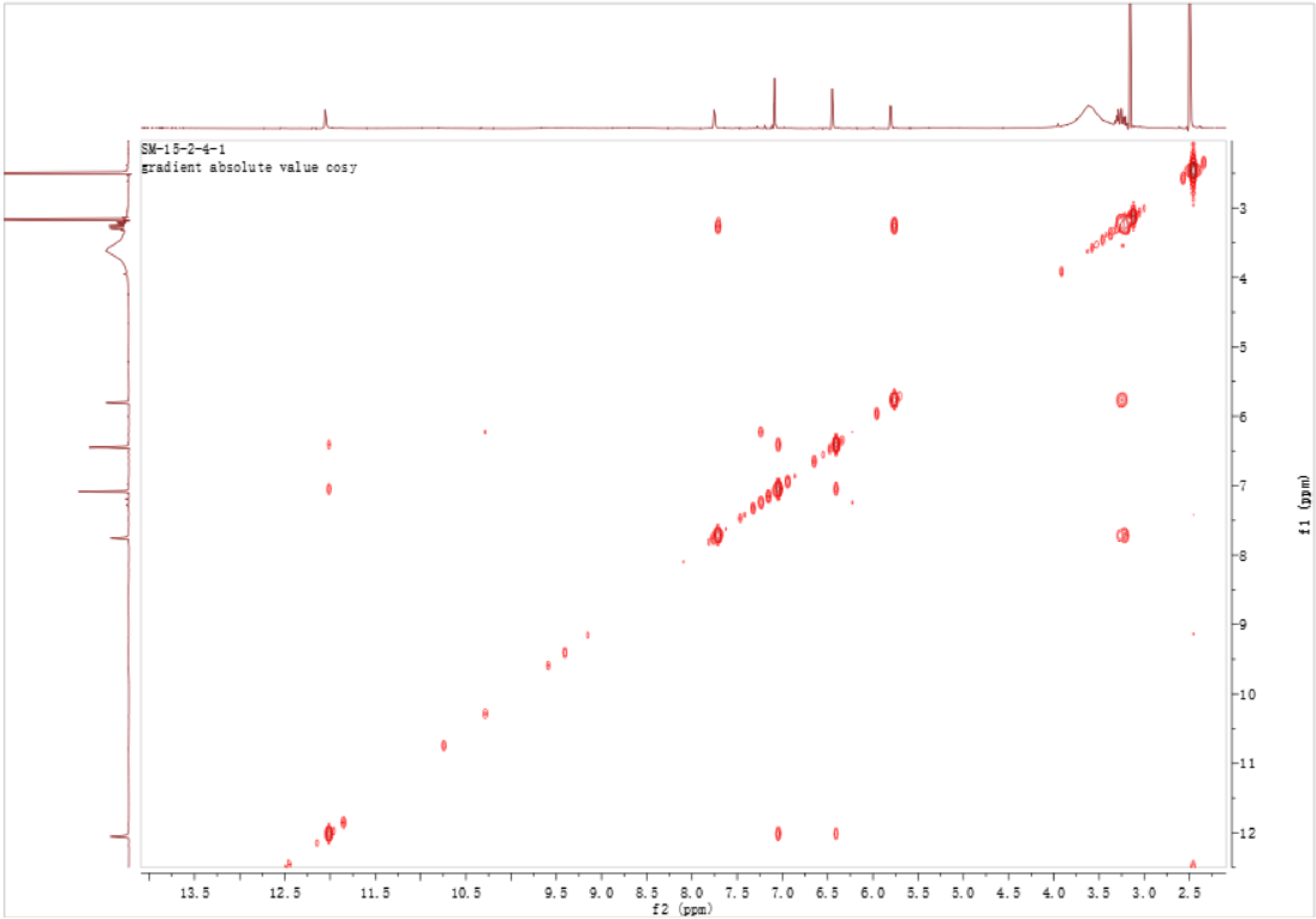

Figure S36. ^{1}H-^{1}H COSY spectrum of compound **2** in DMSO-d_6 (500 MHz).

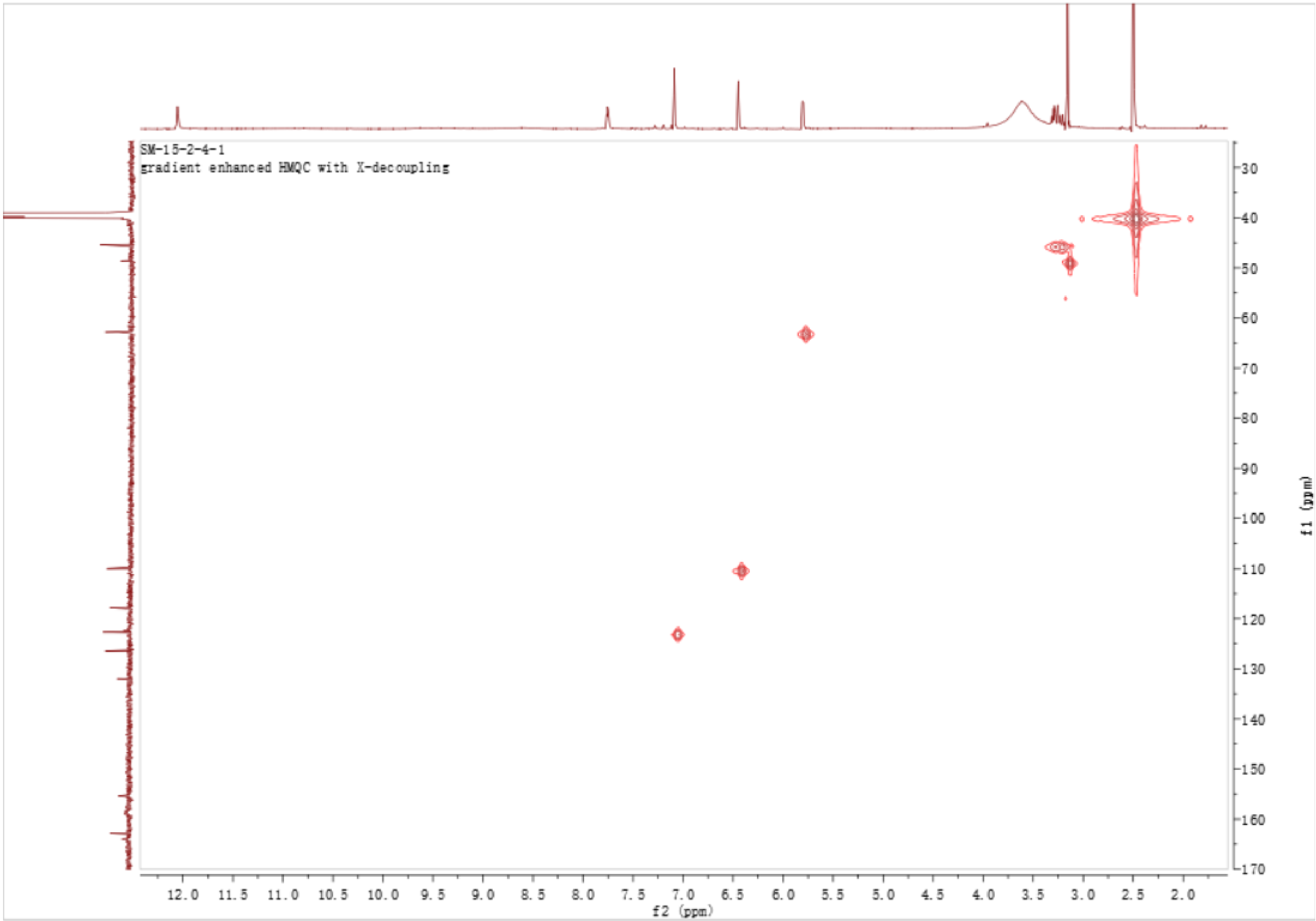

Figure S37. HSQC spectrum of compound **2** in DMSO-d_6 (500 MHz).

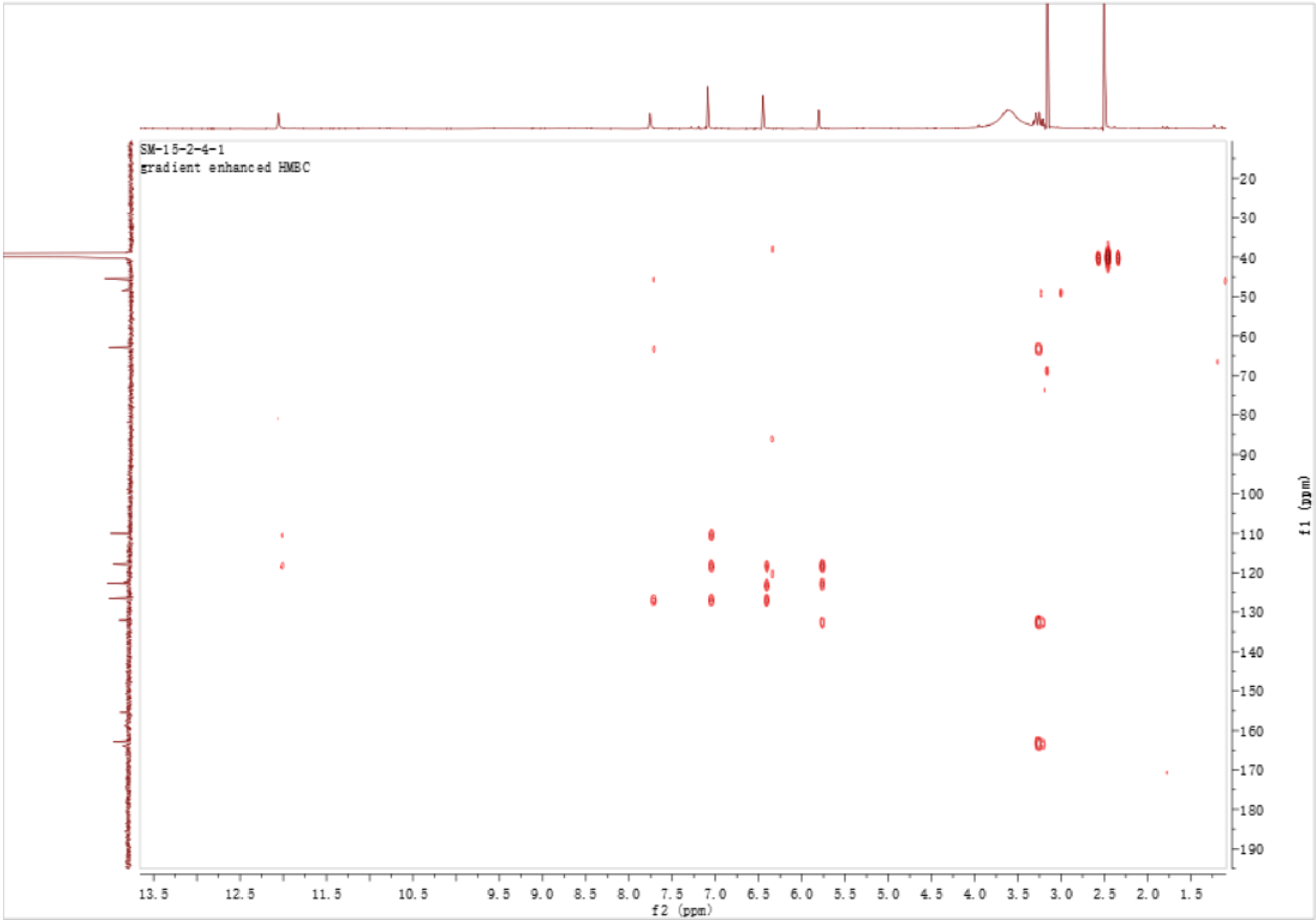

Figure S38. HMBC spectrum of compound **2** in DMSO-d_6 (500 MHz).

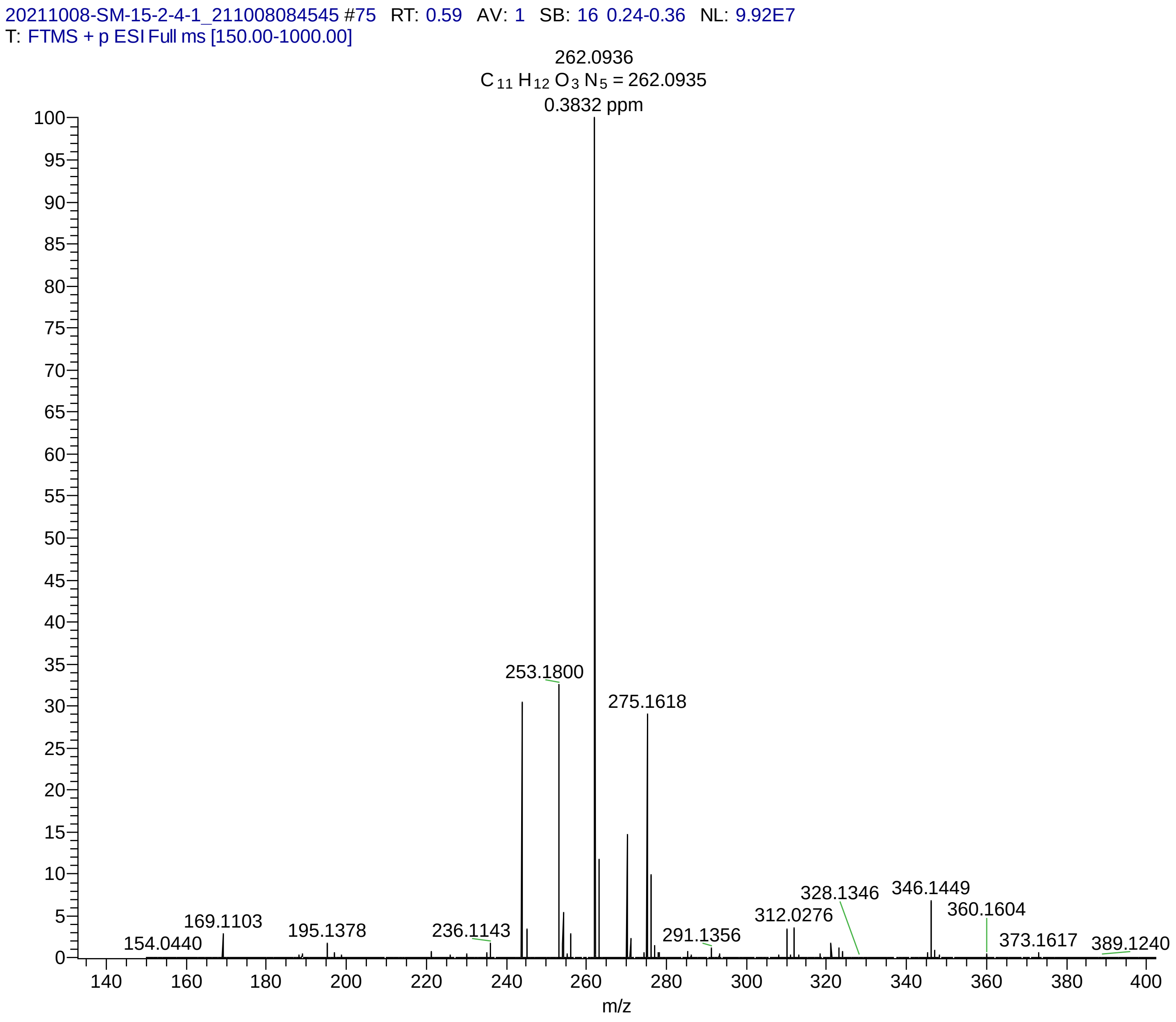

Figure S39. HRESIMS data of compound **2**.

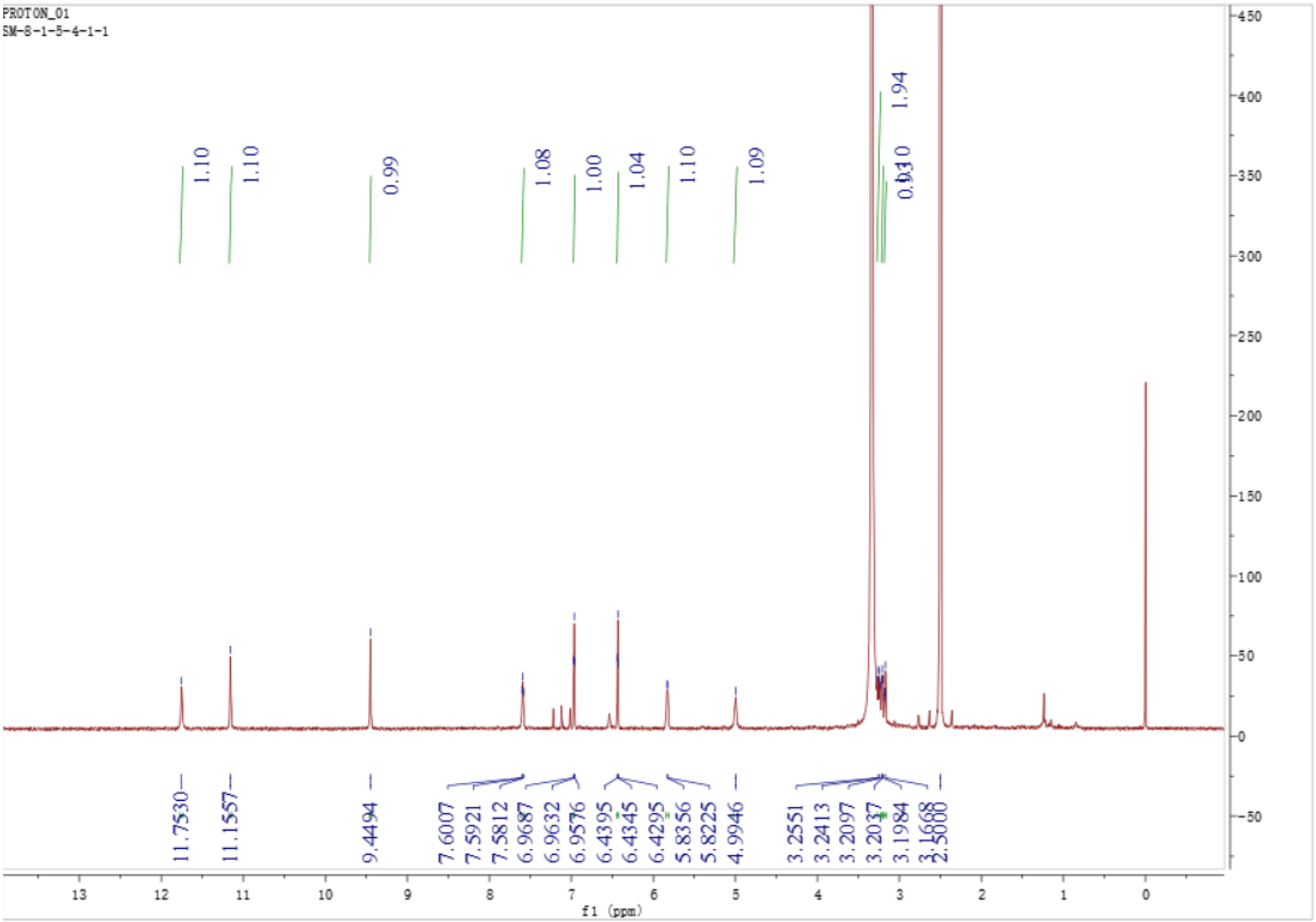

Figure S40. ^{1}H NMR spectrum of compound **3** in DMSO-d_6 (500 MHz).

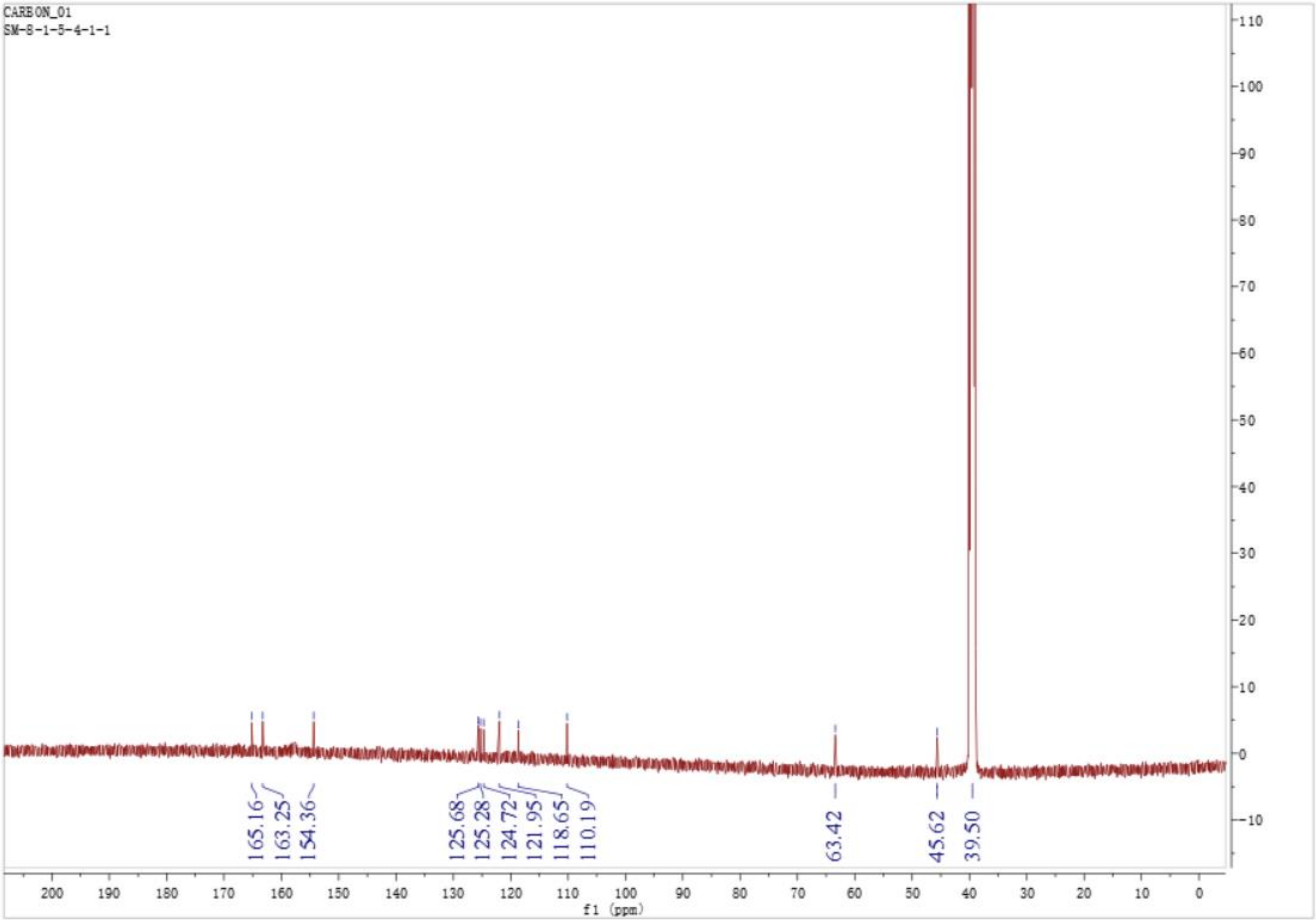

Figure S41. ^{13}C NMR spectrum of compound **3** in DMSO-d_6 (125 MHz).

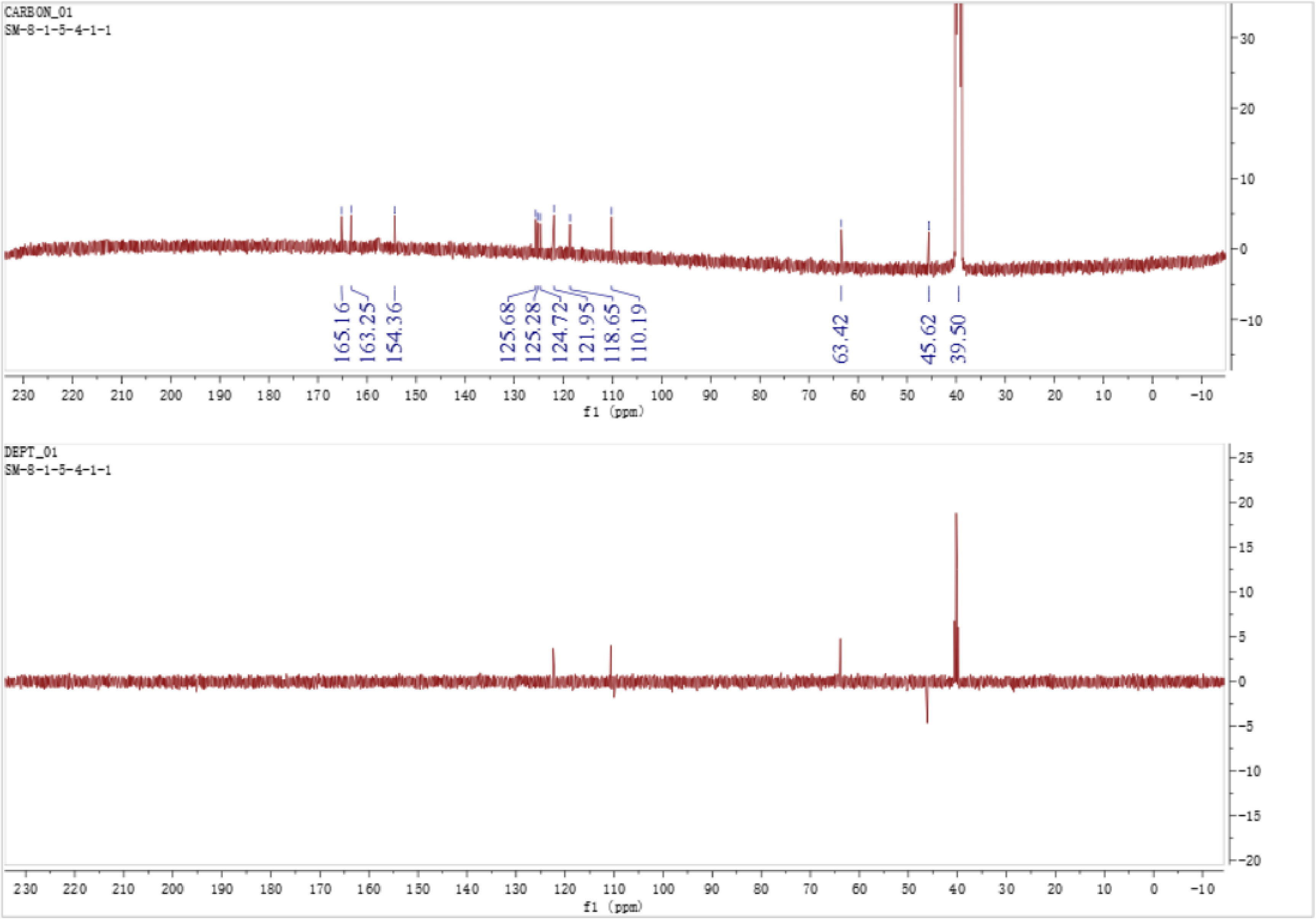

Figure S42. ^{13}C NMR and DEPT spectrum of compound **3** in DMSO-d_6 (125 MHz).

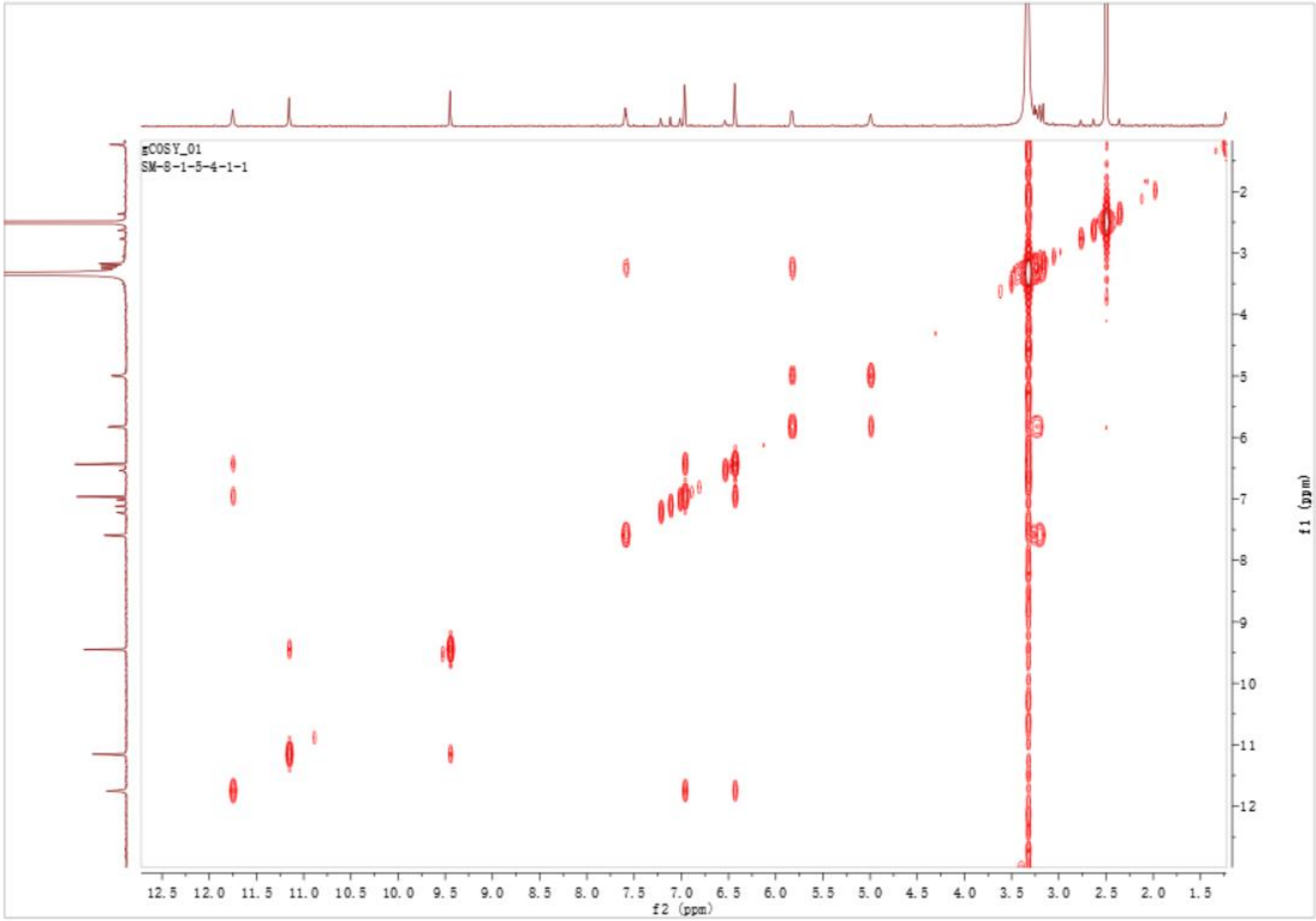

Figure S43. ^{1}H-^{1}H COSY spectrum of compound **3** in DMSO-d_6 (500 MHz)

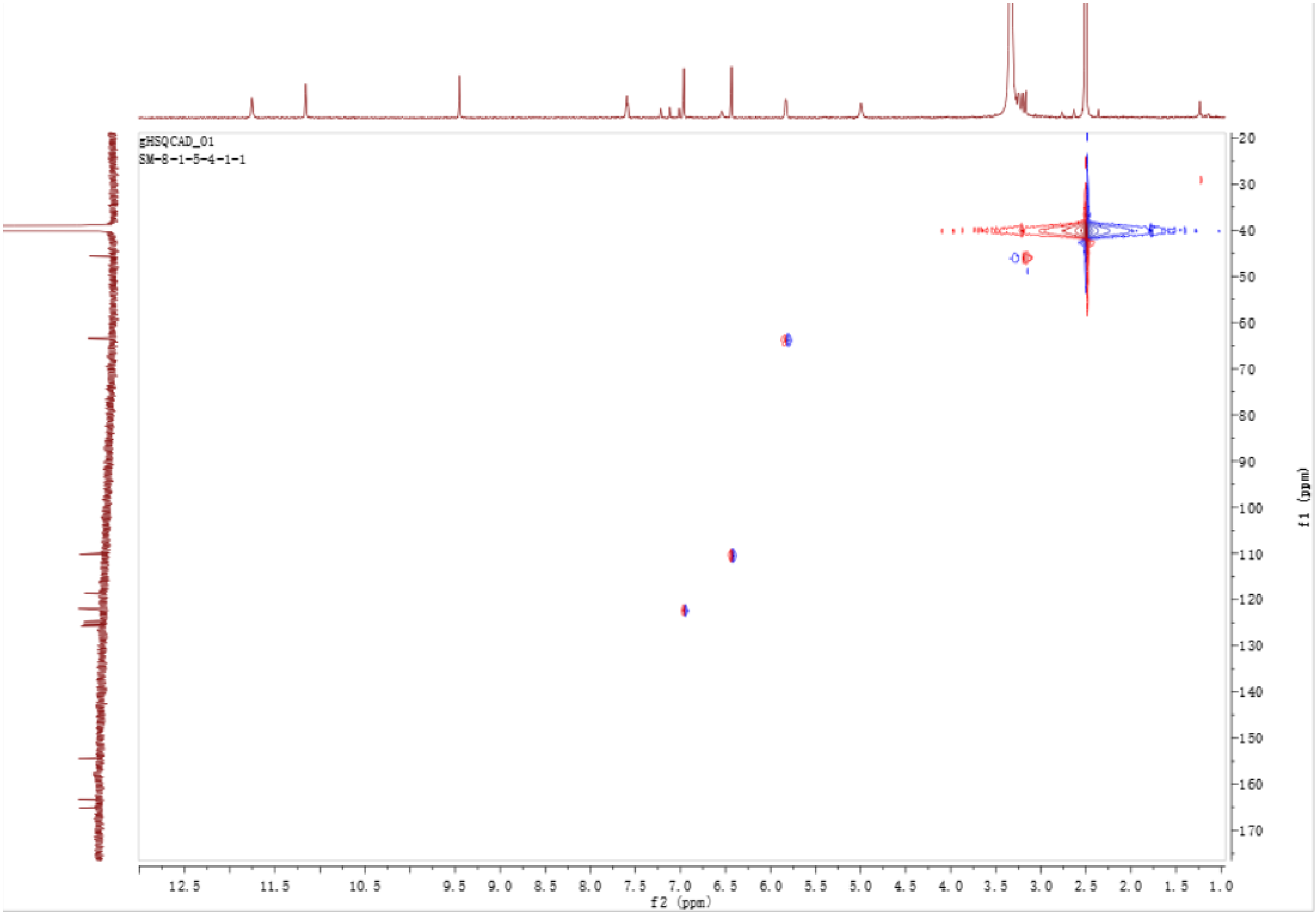

Figure S44. HSQC spectrum of compound **3** in DMSO-d_6 (500 MHz).

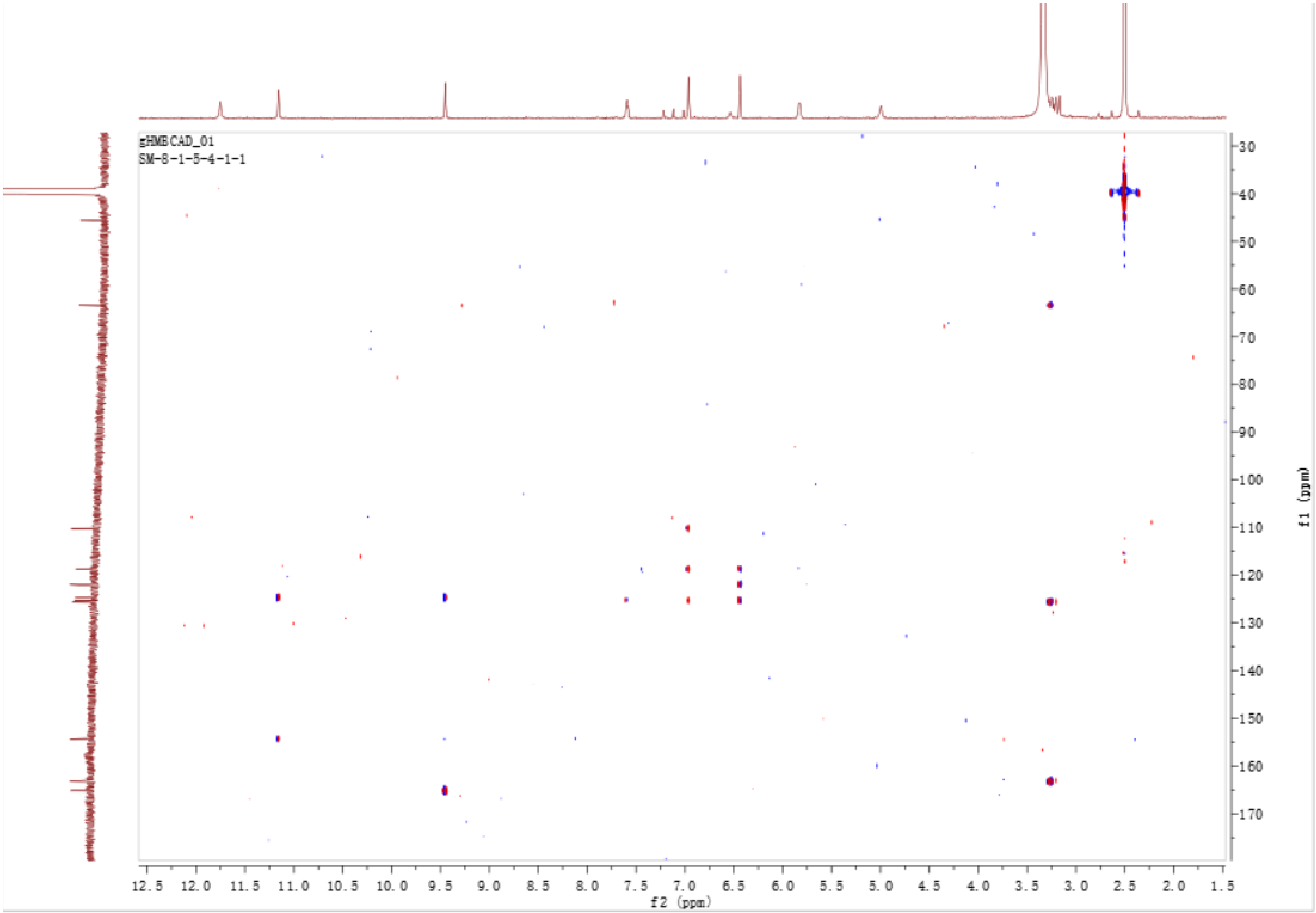

Figure S45. HMBC spectrum of compound **2** in DMSO-d_6 (500 MHz).

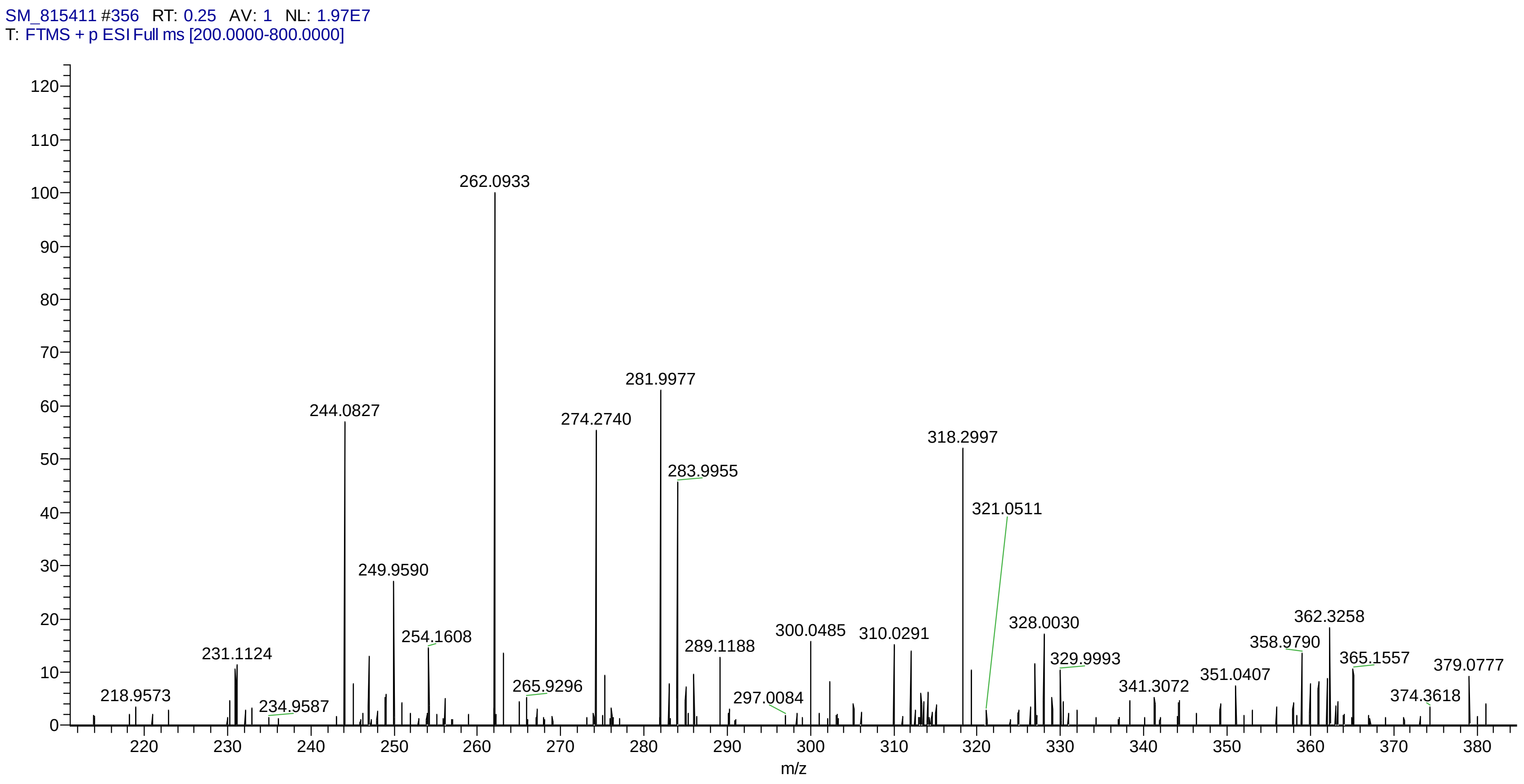

Figure S46. HRESIMS data of compound **3**.

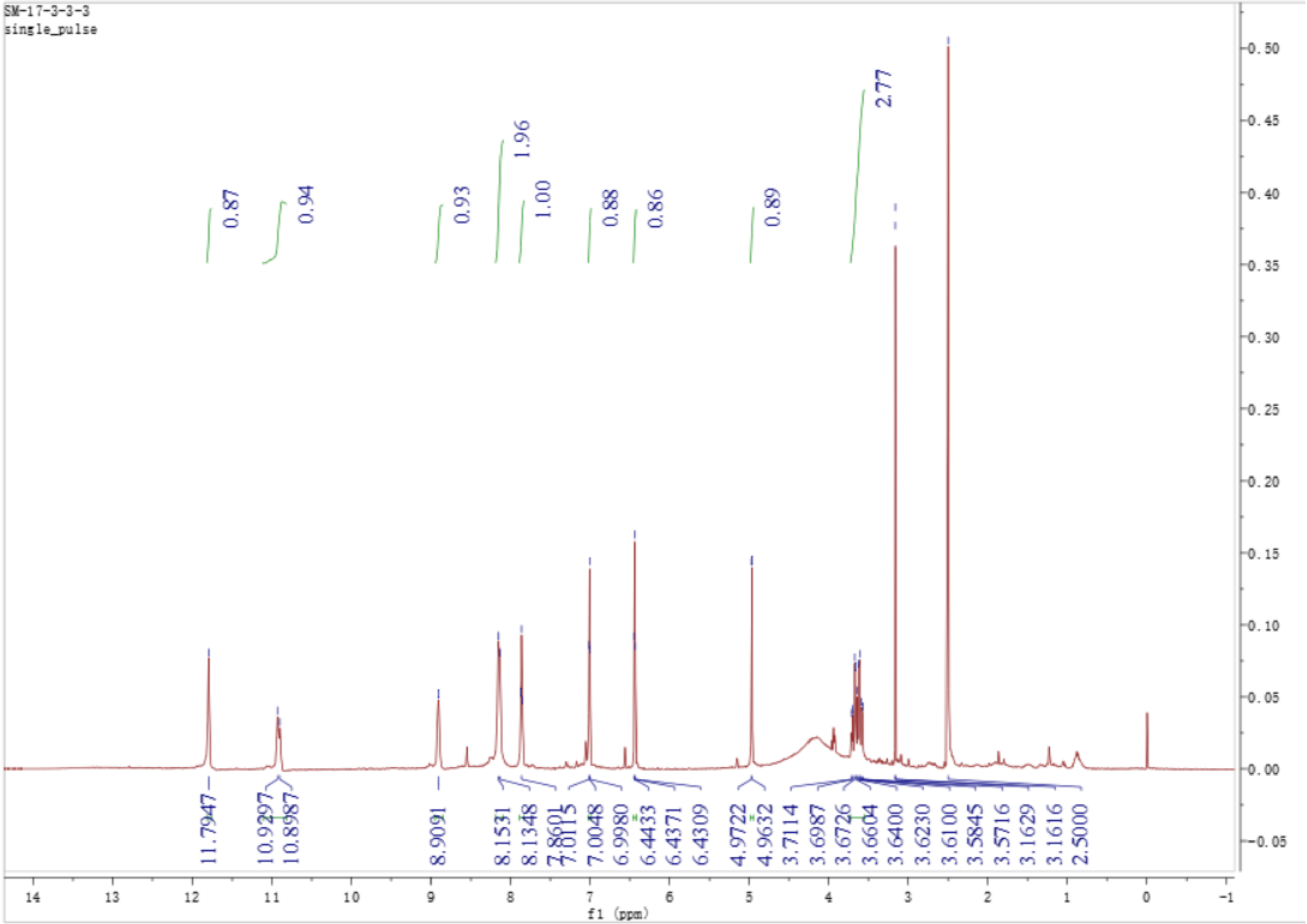

Figure S47. ^{1}H NMR spectrum of compound **4** in DMSO-d_6 (500 MHz)

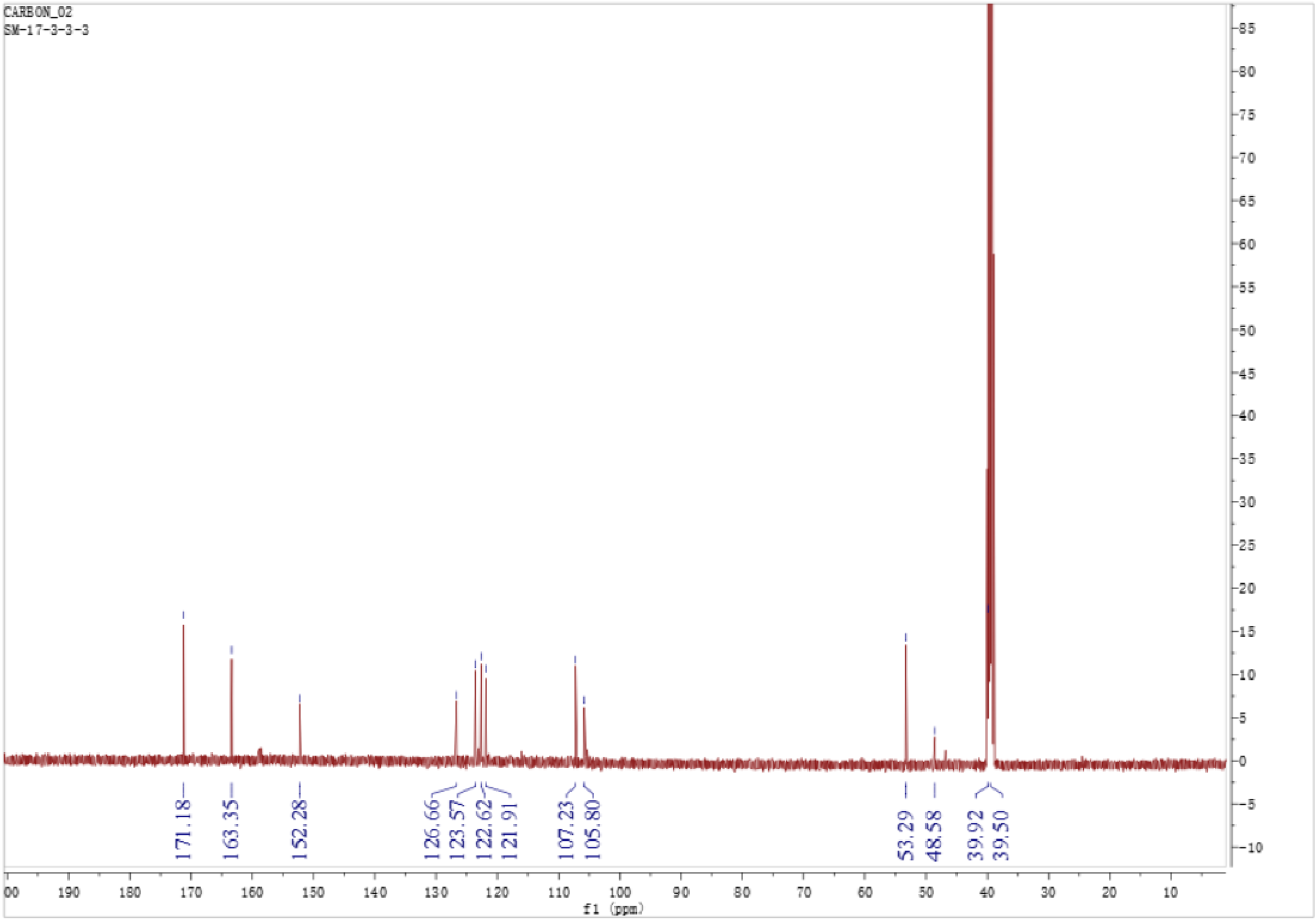

Figure S48. ^{13}C NMR spectrum of compound **4** in DMSO-d_6 (125 MHz).

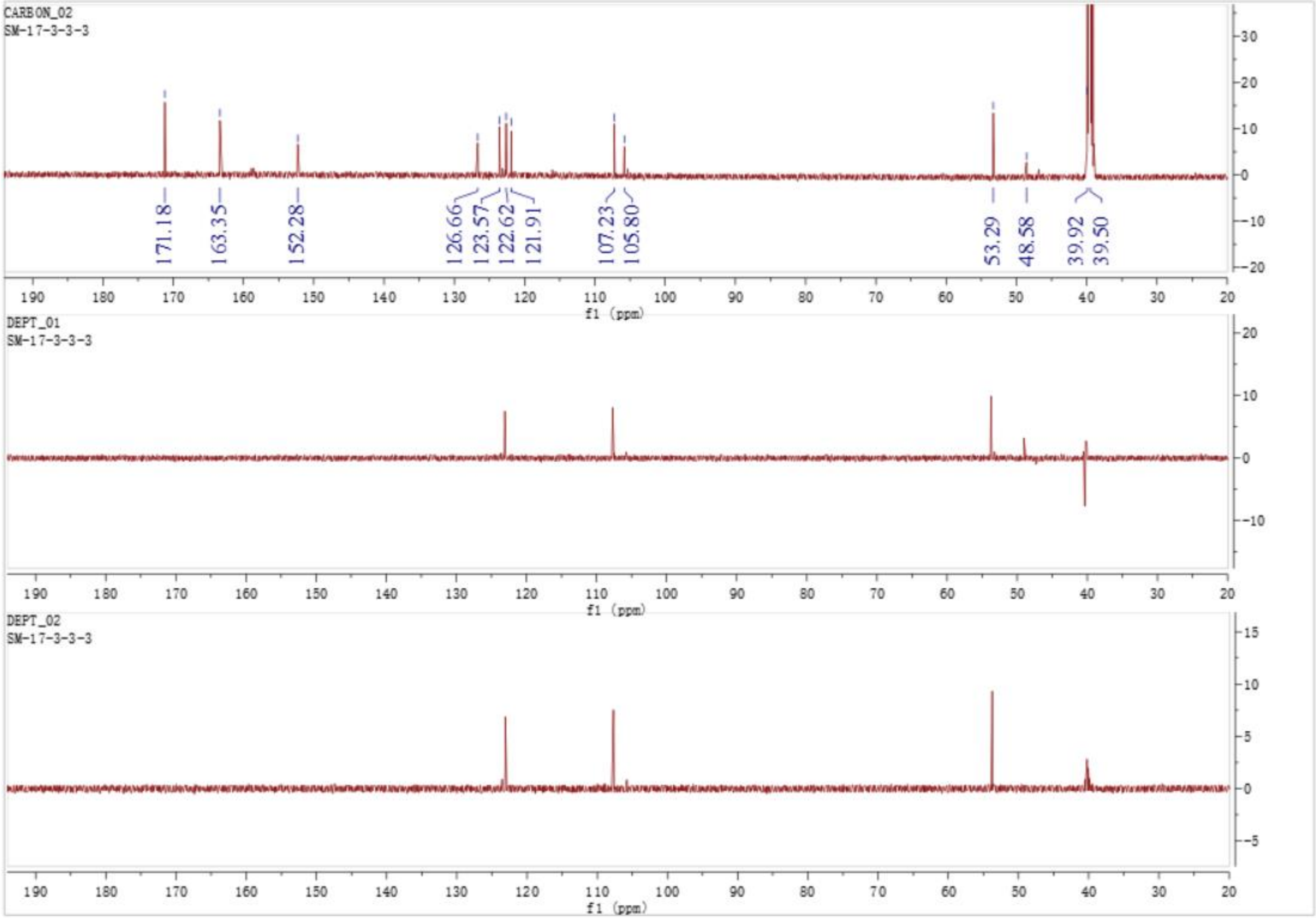

Figure S49. ^{13}C NMR and DEPT spectrum of compound **4** in DMSO-d_6 (125 MHz)

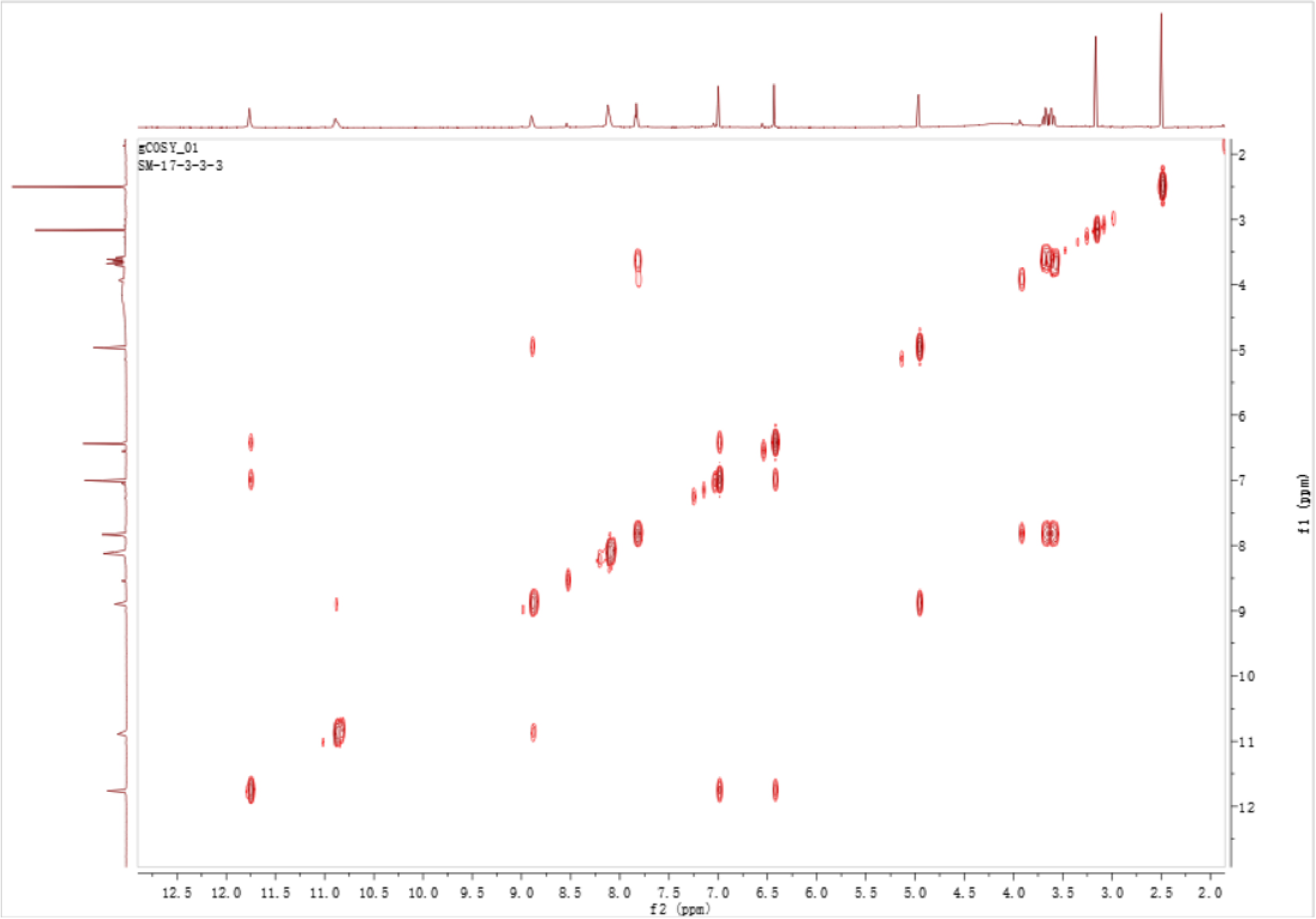

Figure S50. ^1H-^1H COSY spectrum of compound **4** in DMSO-d_6 (500 MHz).

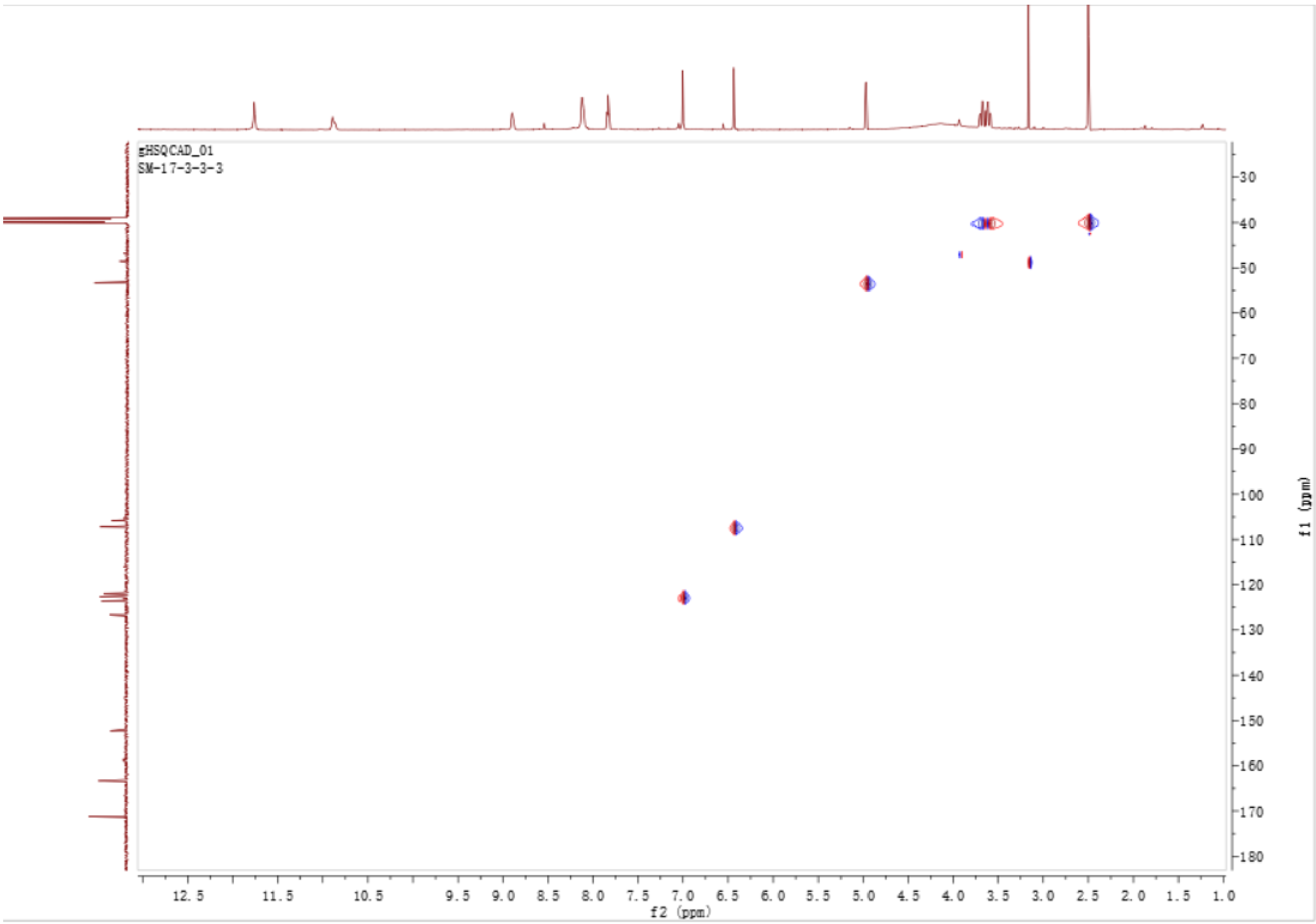

Figure S51. HSQC spectrum of compound **4** in DMSO-d_6 (500 MHz).

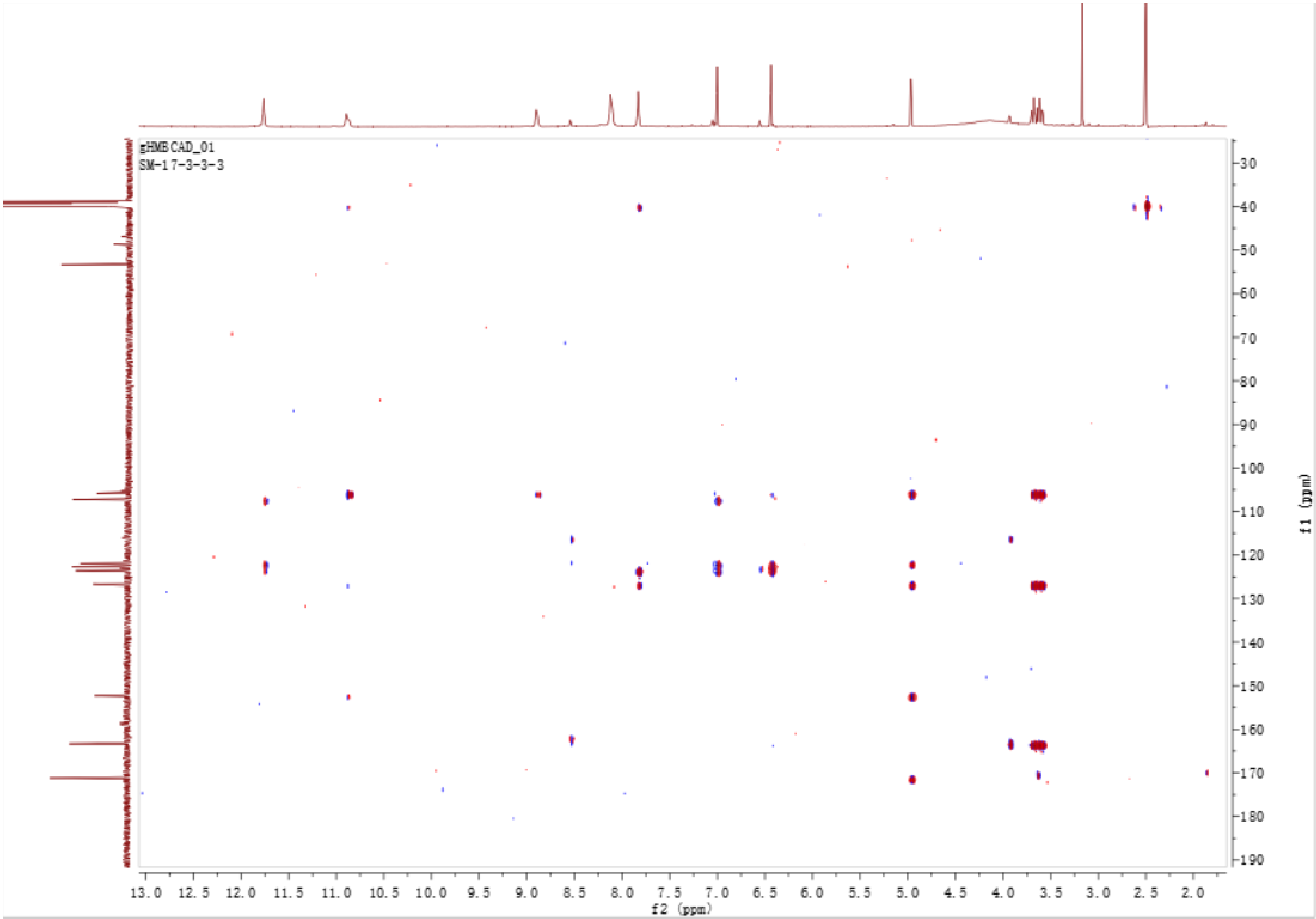

Figure S52. HMBC spectrum of compound **4** in DMSO-d_6 (500 MHz).

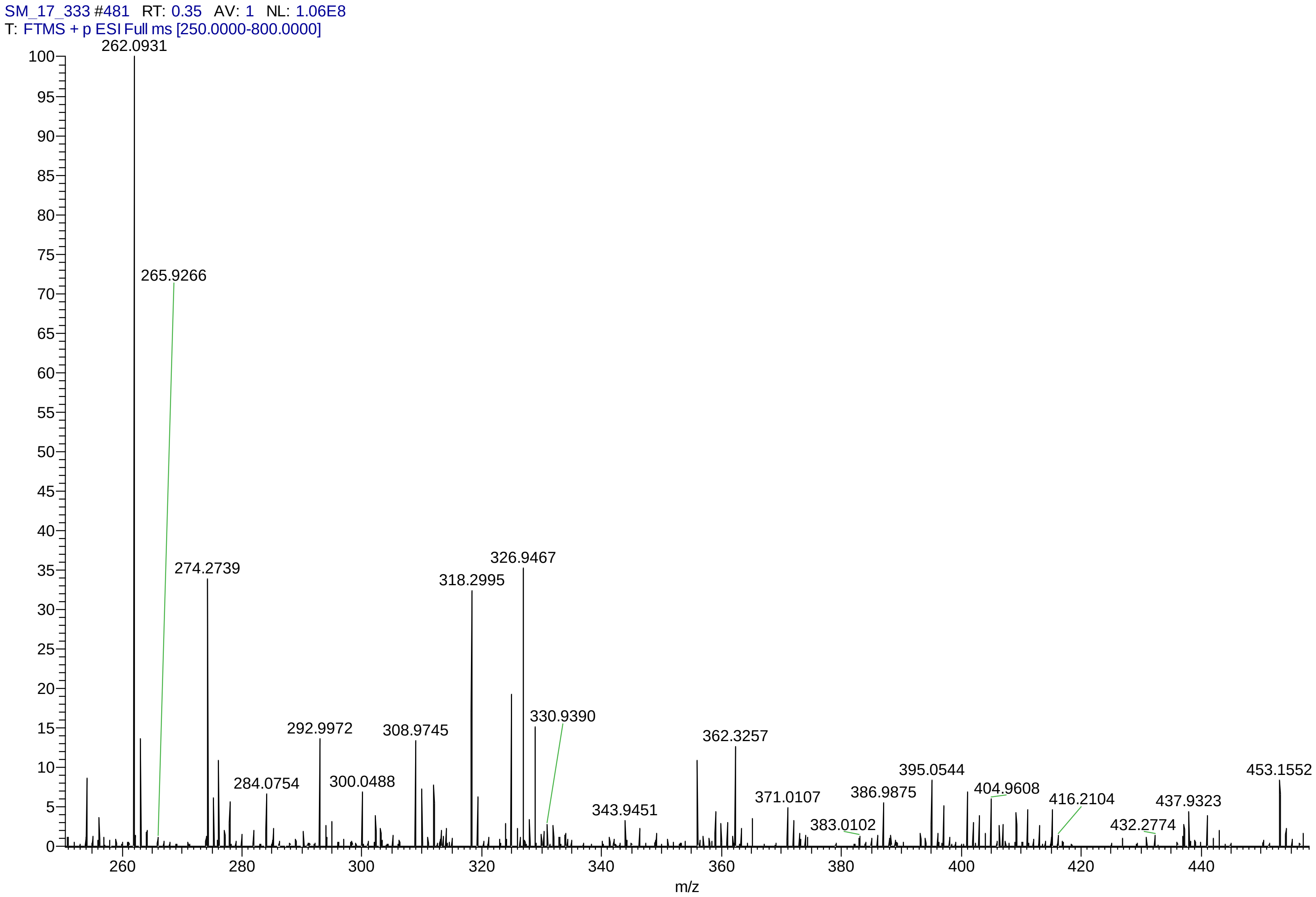

Figure S53. HRESIMS data of compound **4**.

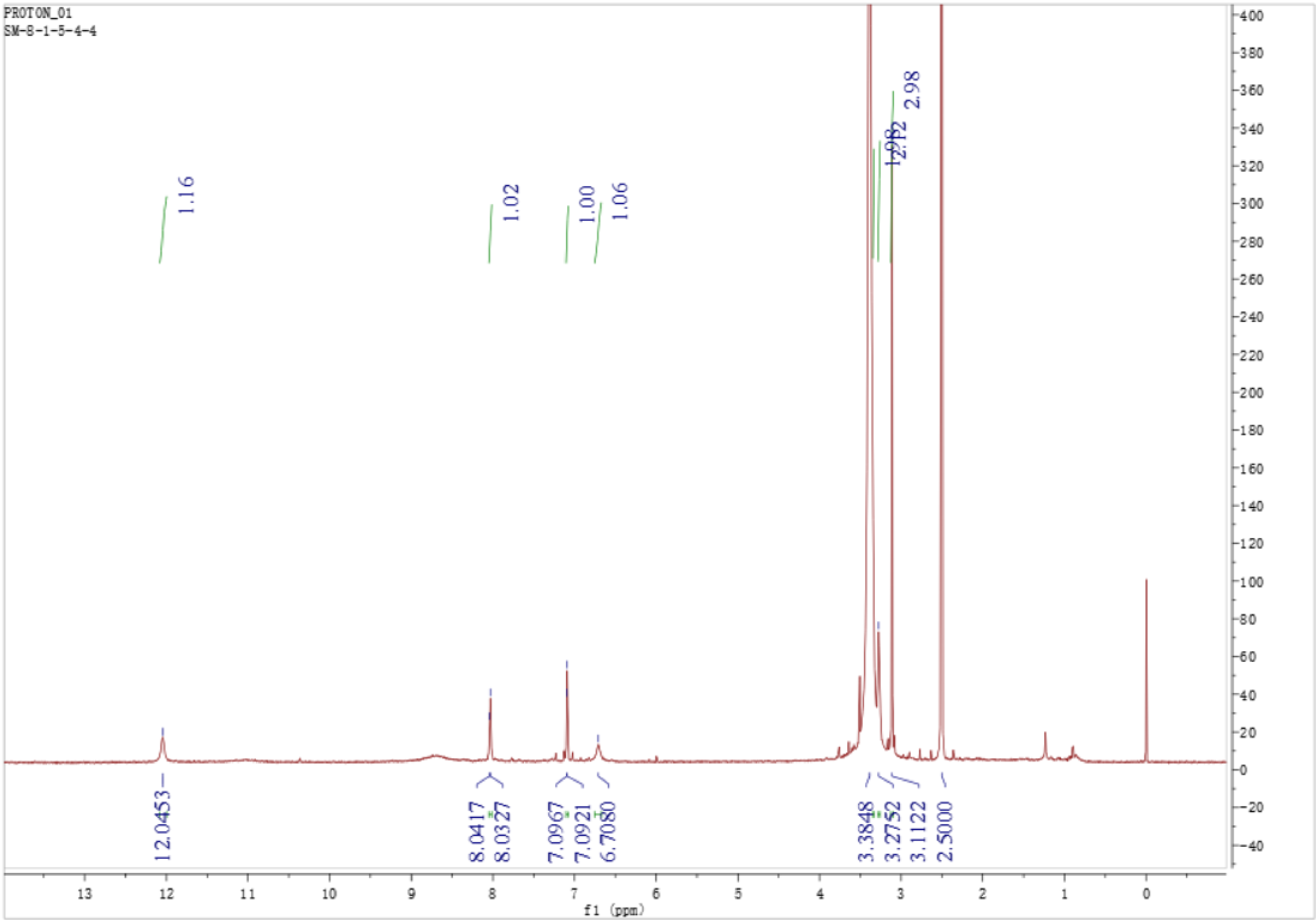

Figure S54. ^{1}H NMR spectrum of compound **5** in DMSO-d_6 (500 MHz).

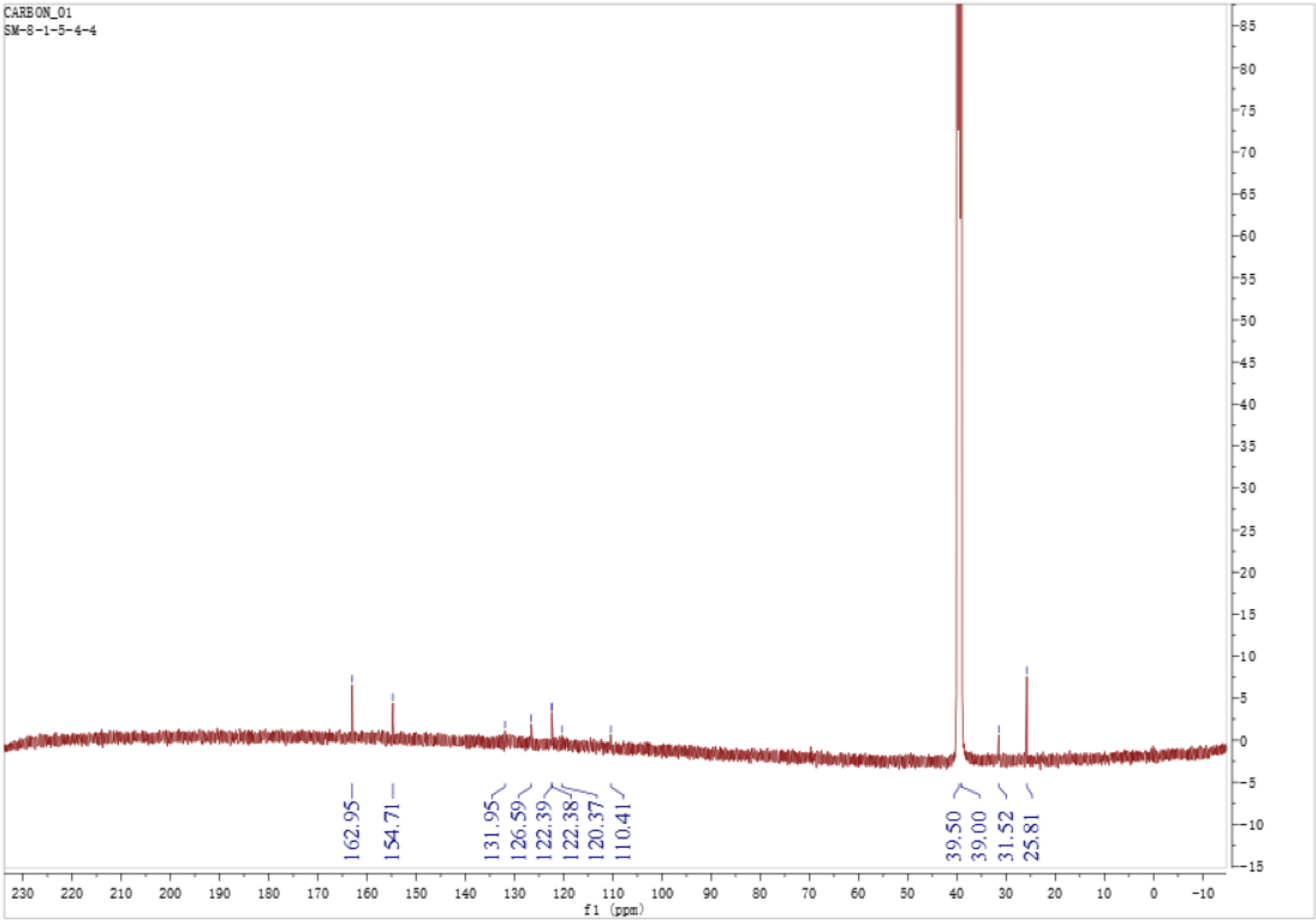

Figure S55. ^{13}C NMR spectrum of compound **5** in DMSO-d_6 (125 MHz).

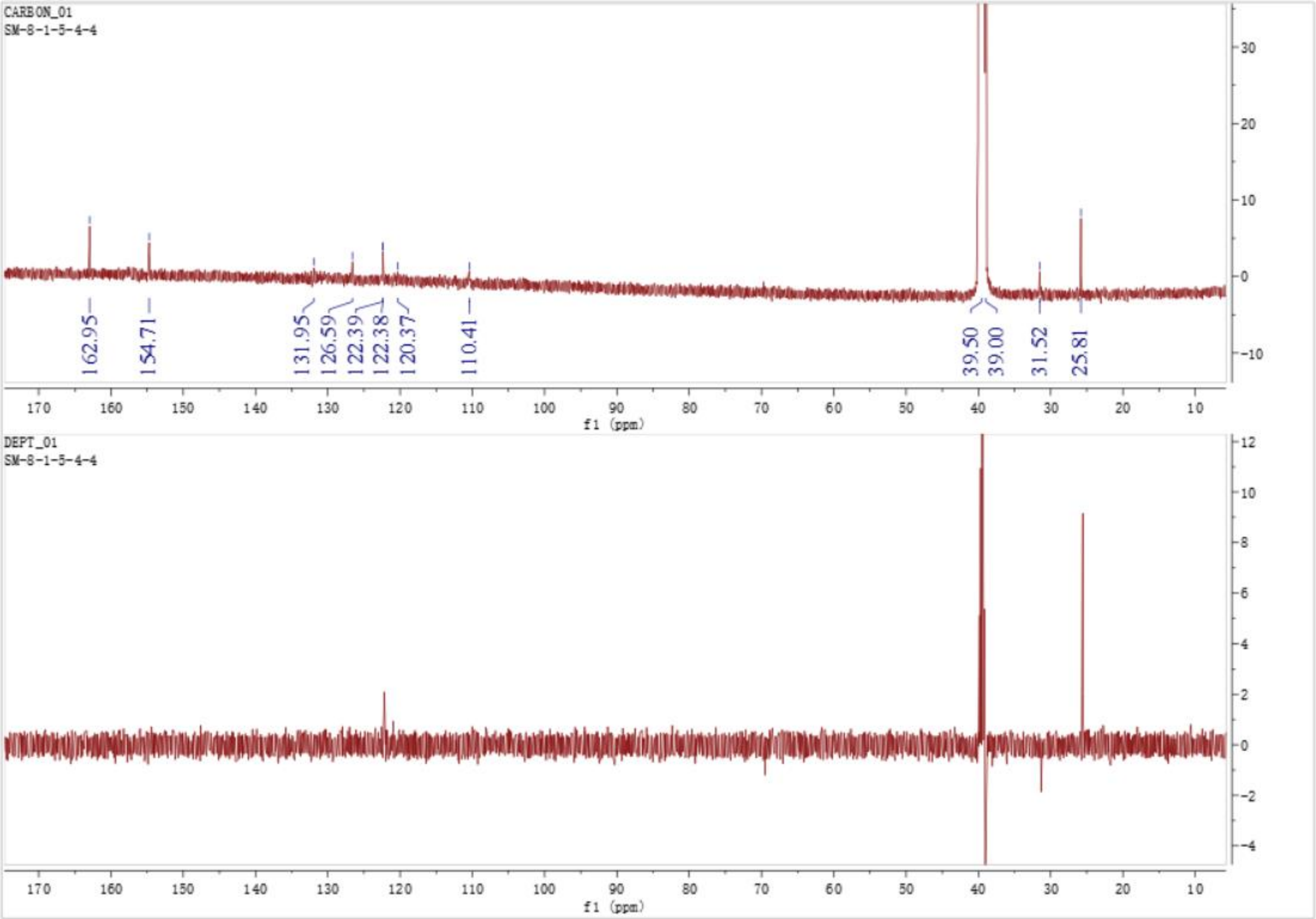

Figure S56. ^{13}C NMR and DEPT spectrum of compound **5** in DMSO-d_6 (125 MHz).

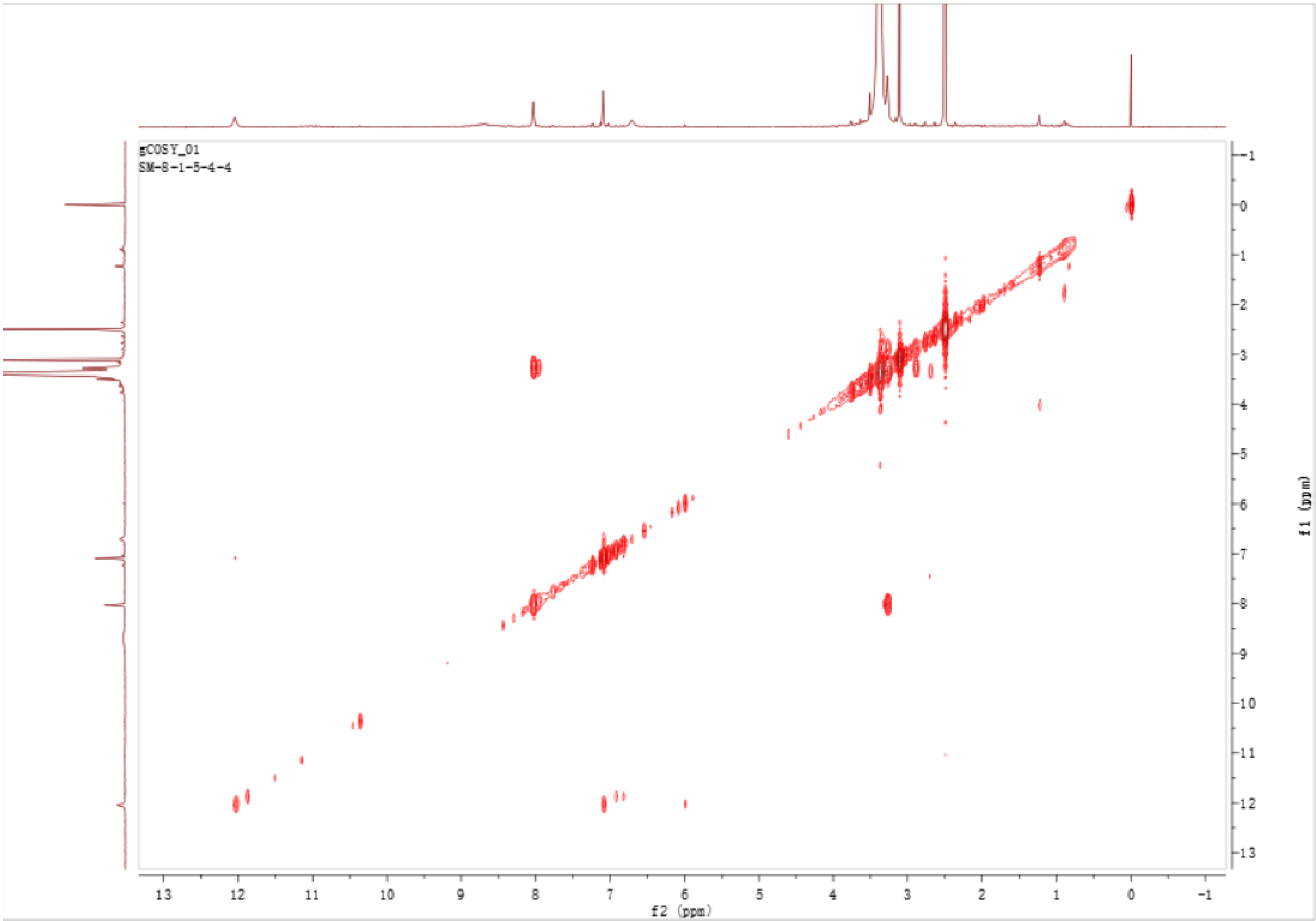

Figure S57. ^{1}H-^{1}H COSY spectrum of compound **5** in DMSO-d_6 (500 MHz).

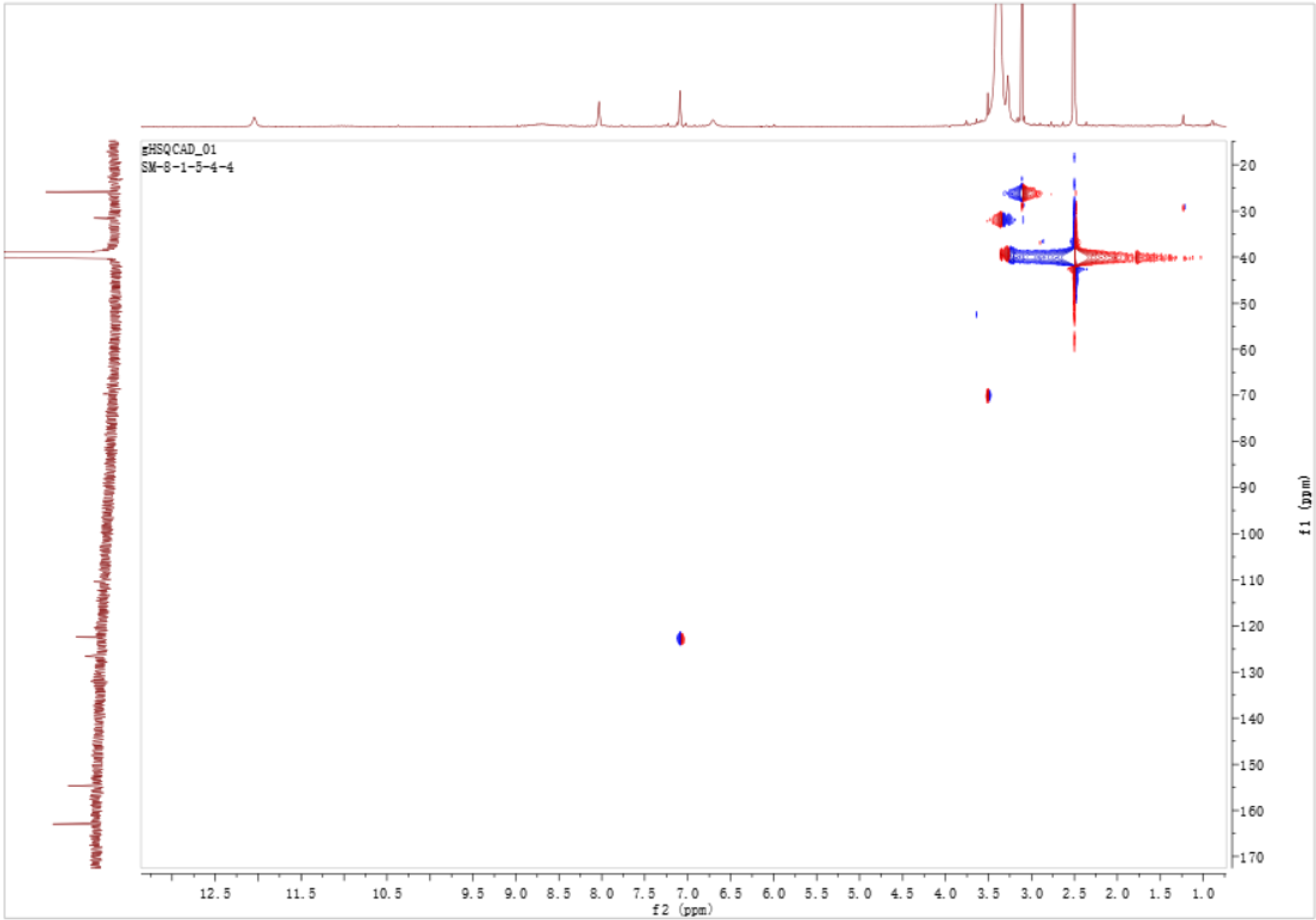

Figure S58. HSQC spectrum of compound **5** in DMSO-d_6 (500 MHz)

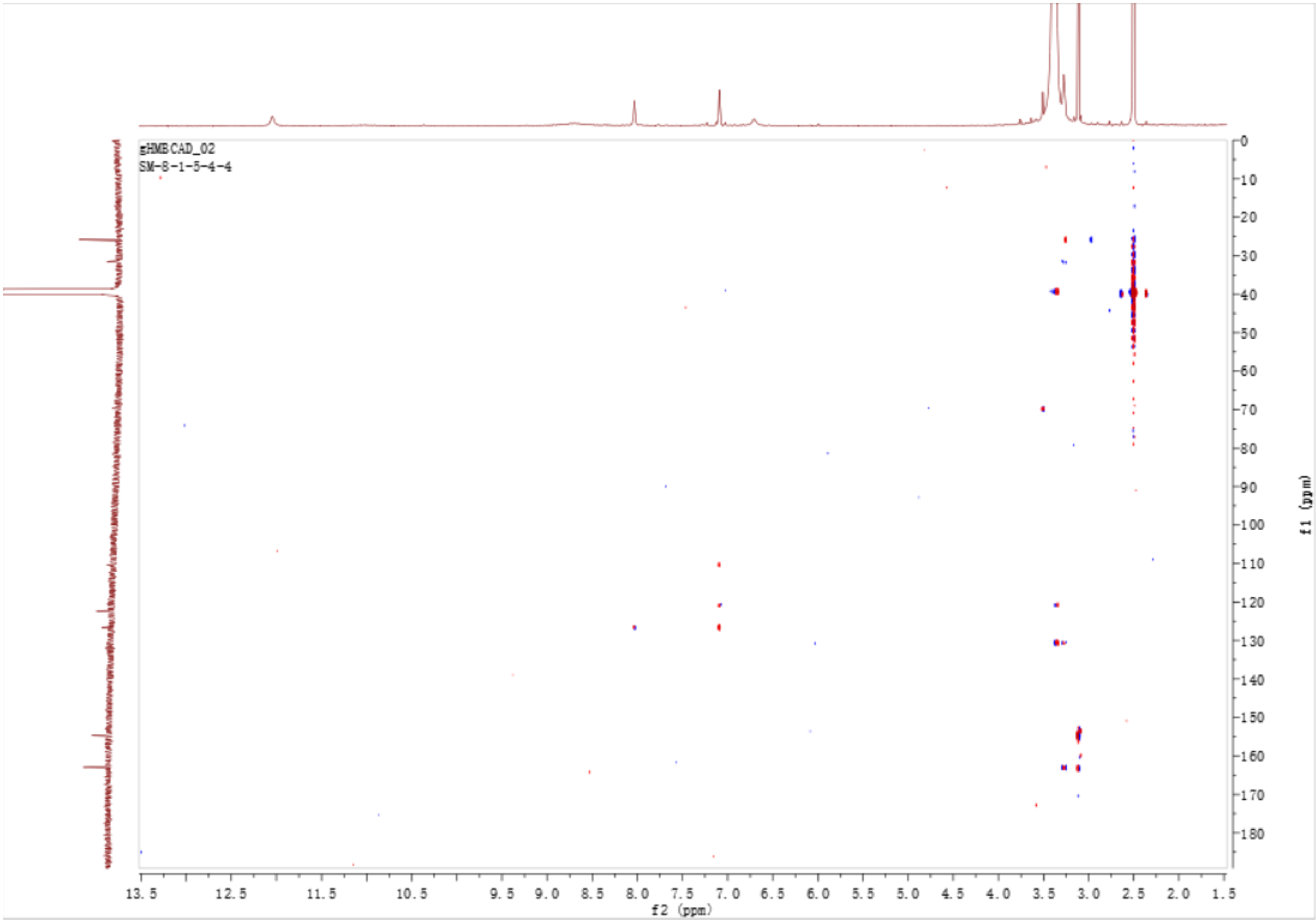

Figure S59. HMBC spectrum of compound **5** in DMSO-d_6 (500 MHz)

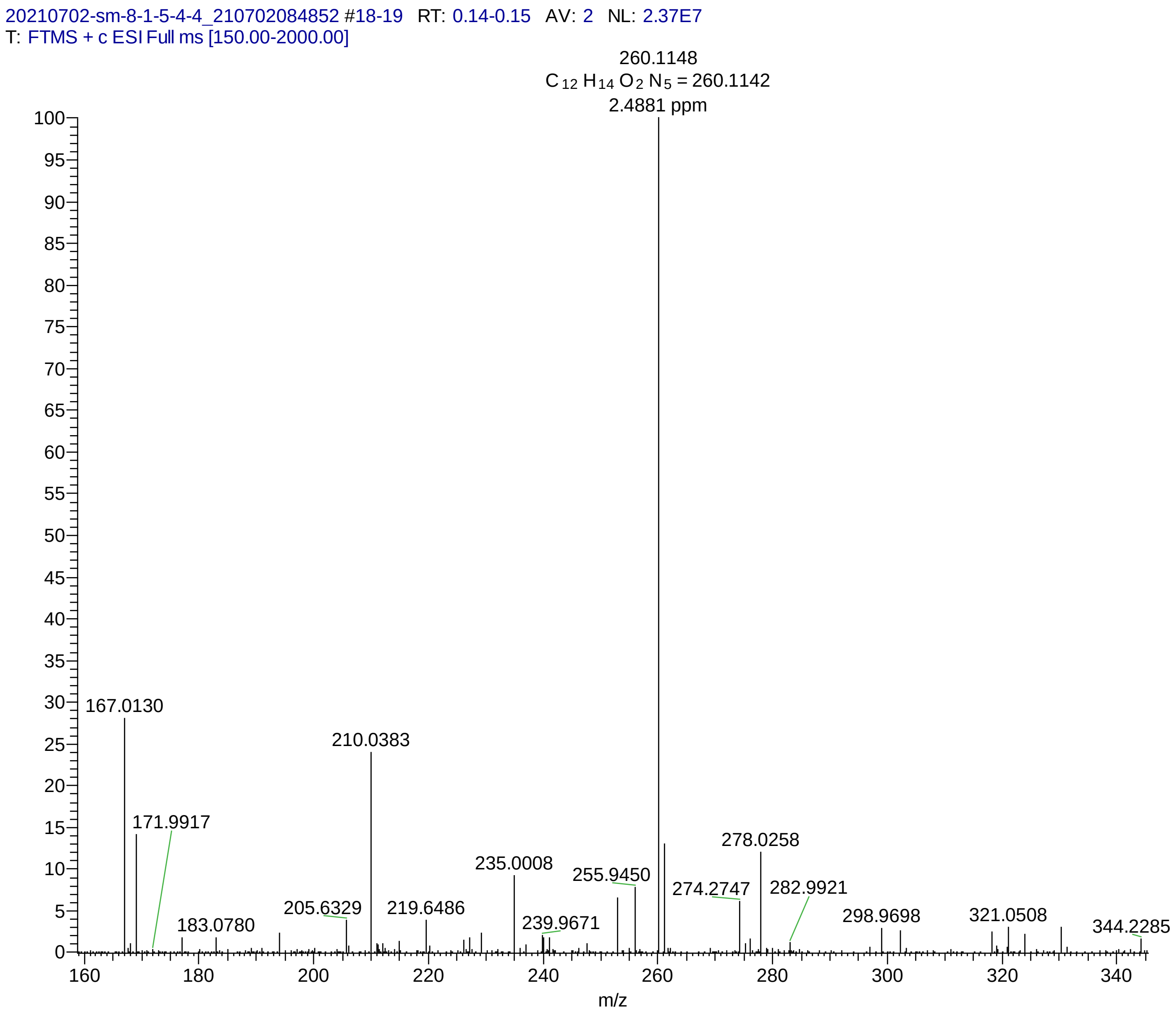

Figure S60. HRESIMS data of compound **5**.

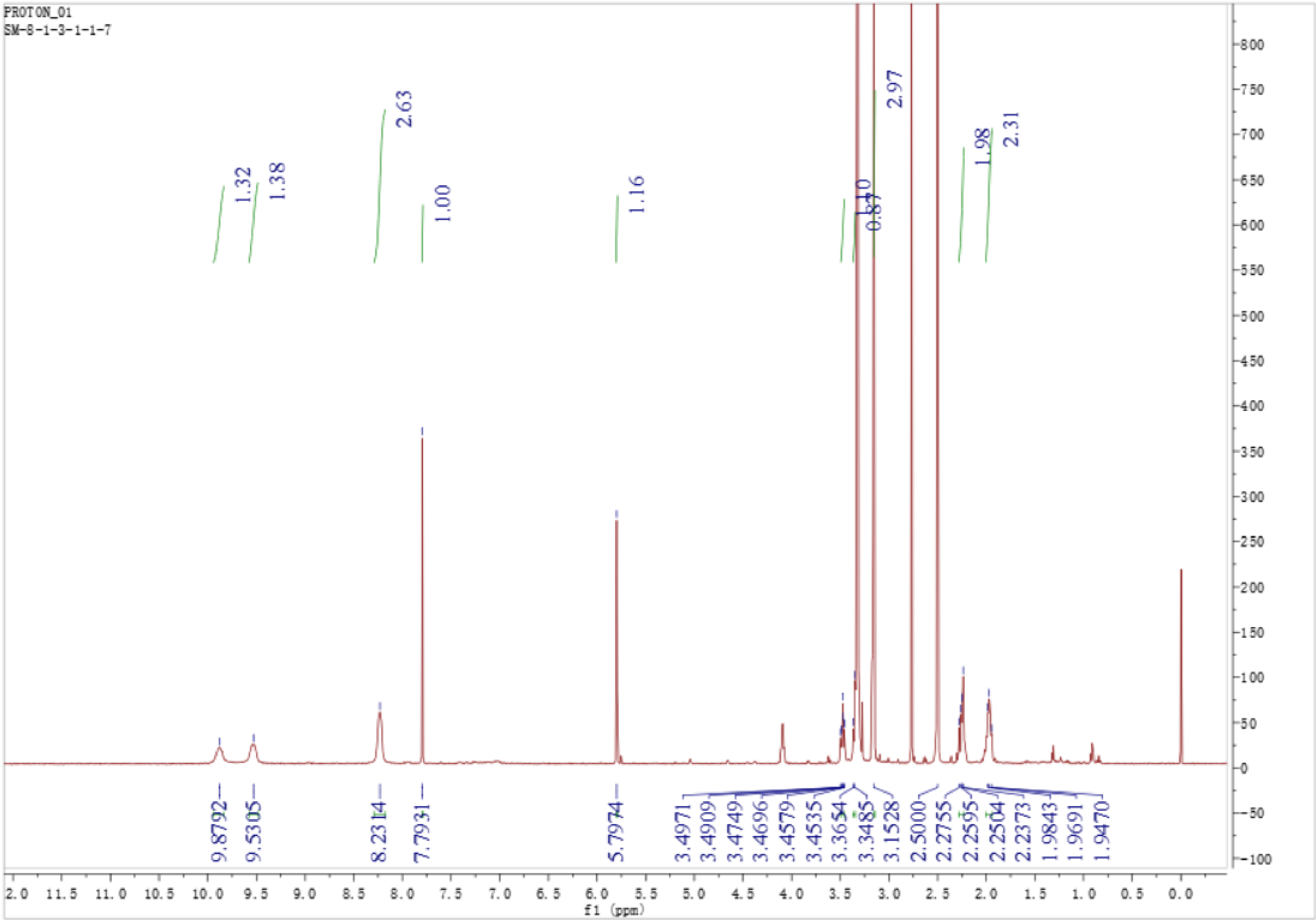

Figure S61. ^{1}H NMR spectrum of compound **6** in DMSO-d_6 (500 MHz).

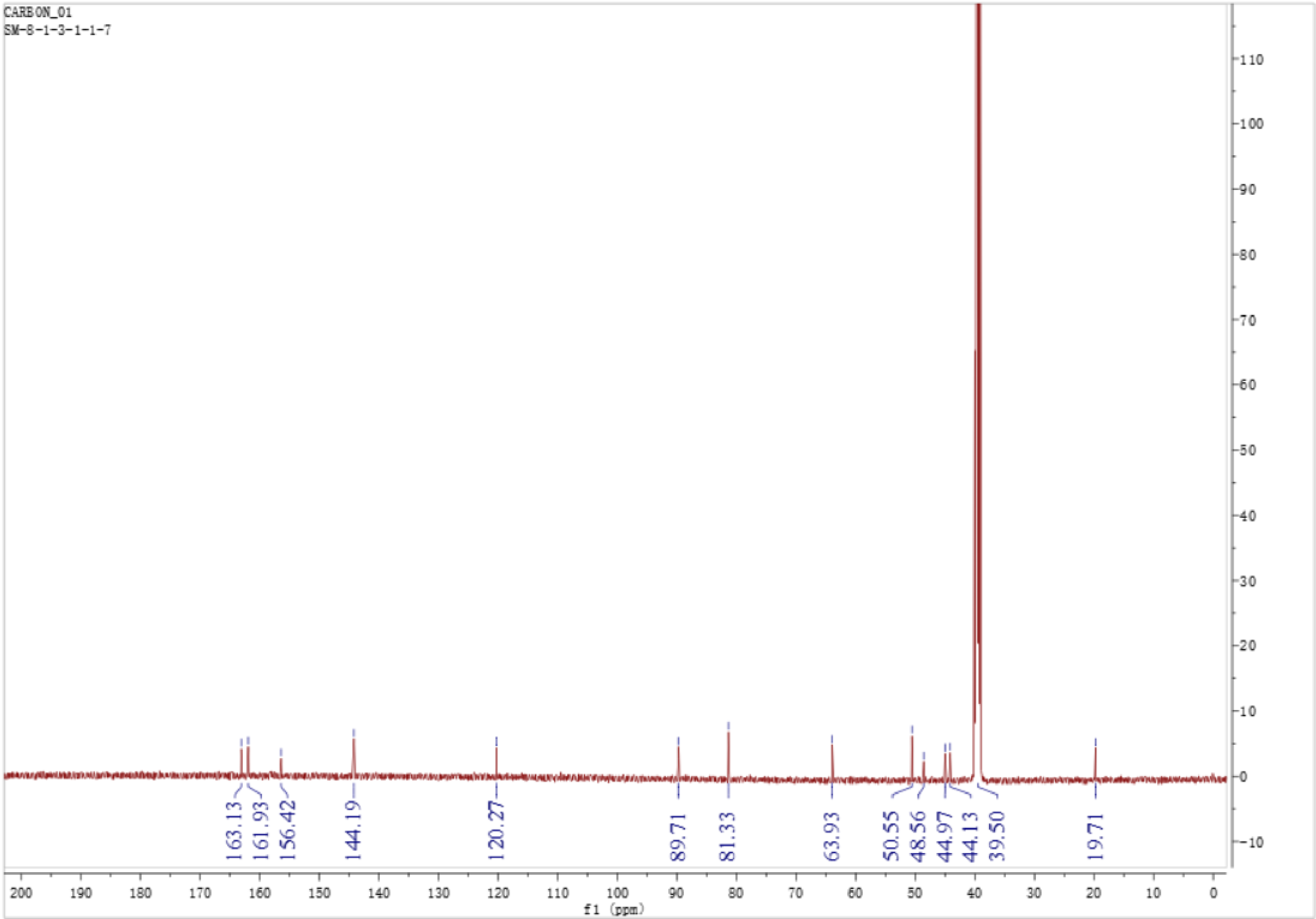

Figure S62. ^{13}C NMR spectrum of compound **6** in DMSO-d_6 (125 MHz).

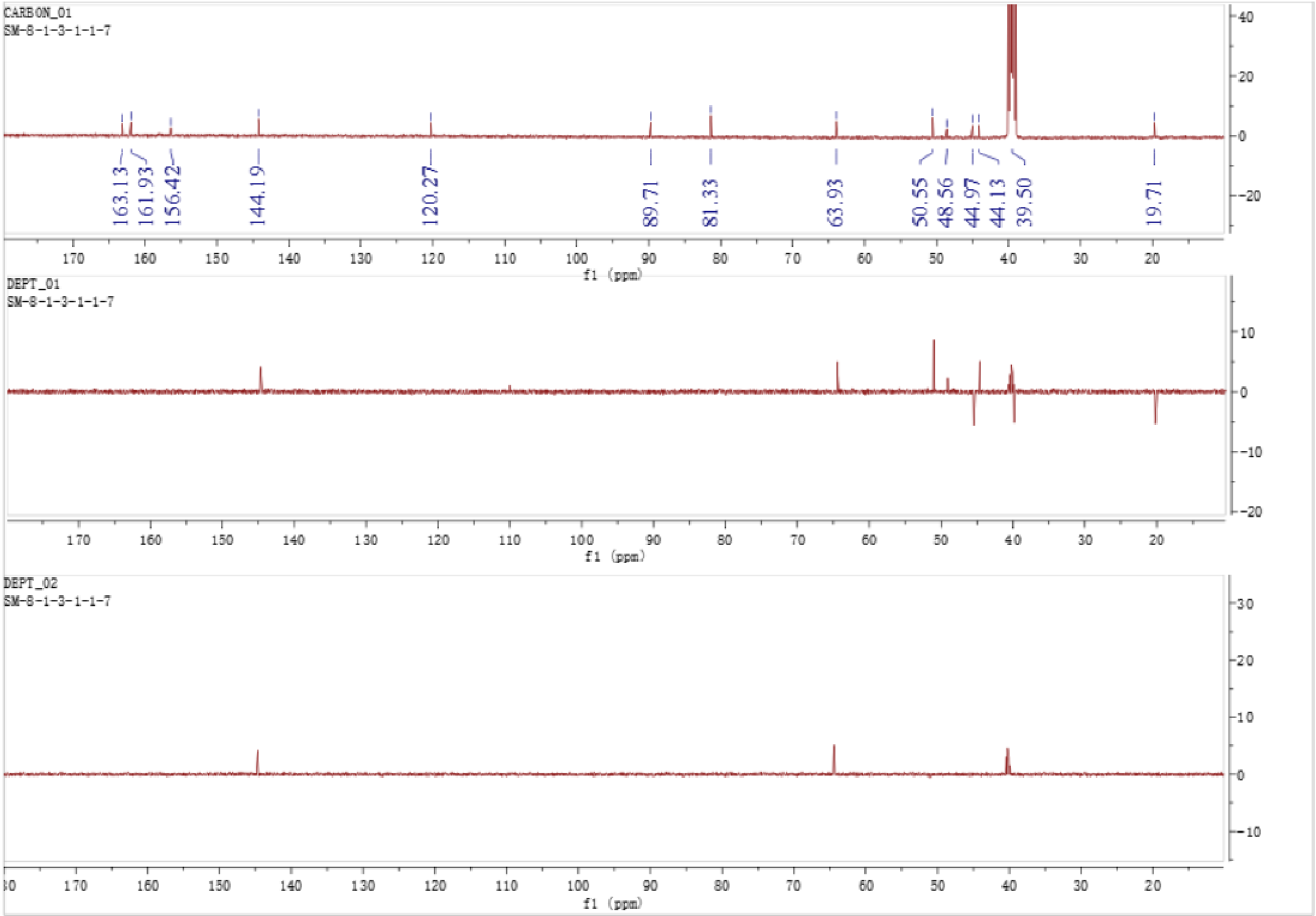

Figure S63. ^{13}C NMR and DEPT spectrum of compound **6** in DMSO-d_6 (125 MHz).

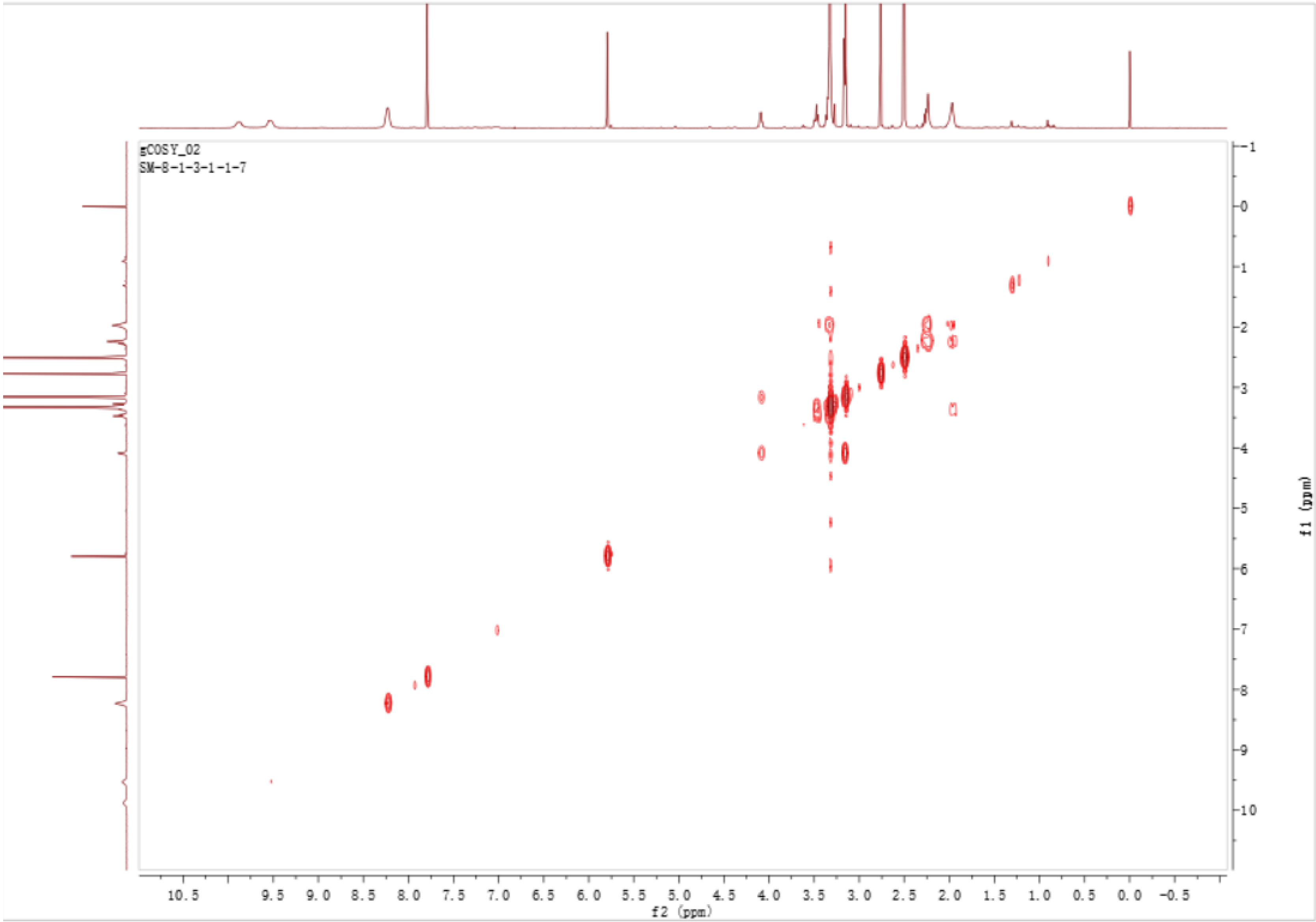

Figure S64. ^{1}H-^{1}H COSY spectrum of compound **6** in DMSO-d_6 (500 MHz).

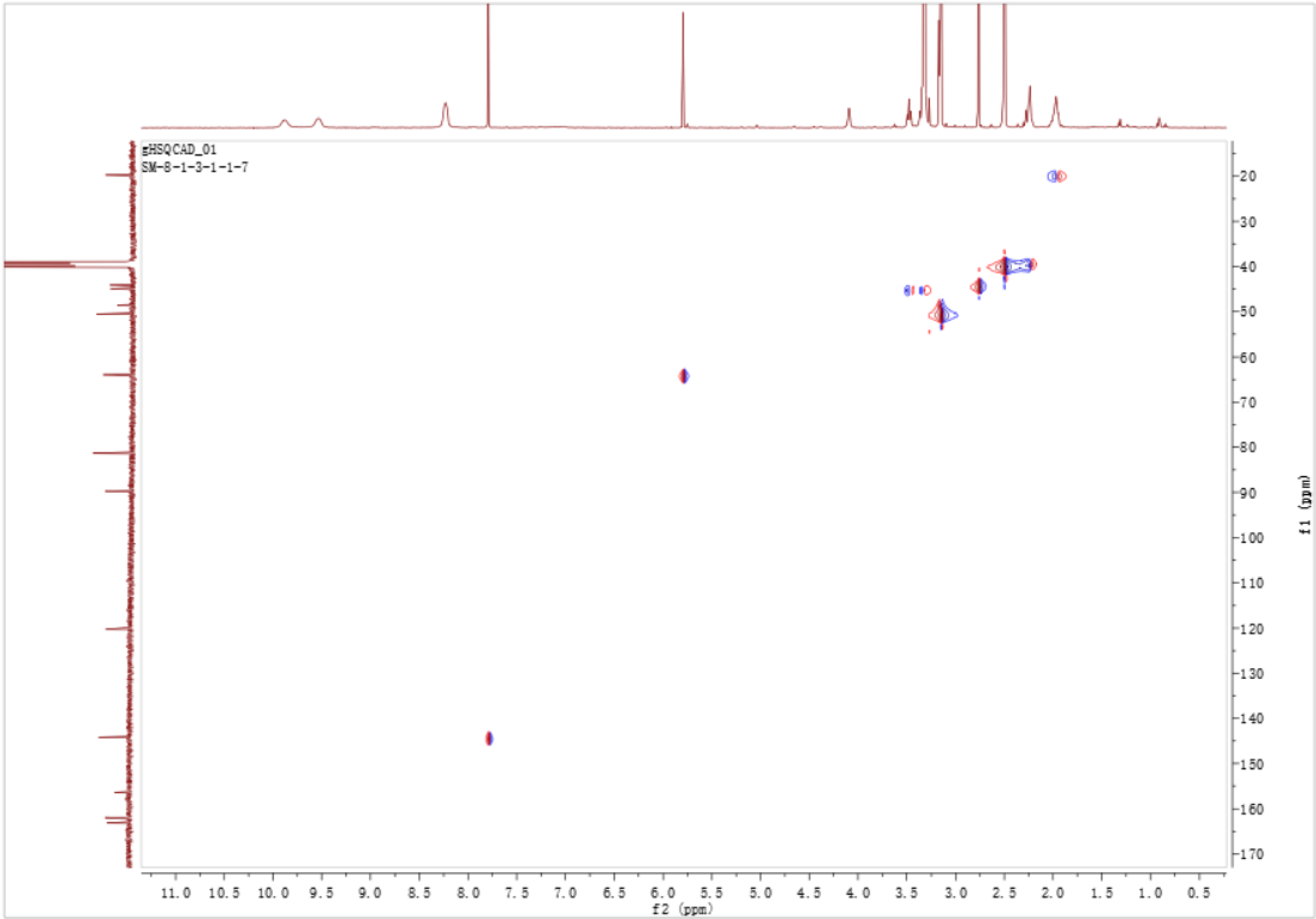

Figure S65. HSQC spectrum of compound **6** in DMSO-d_6 (500 MHz).

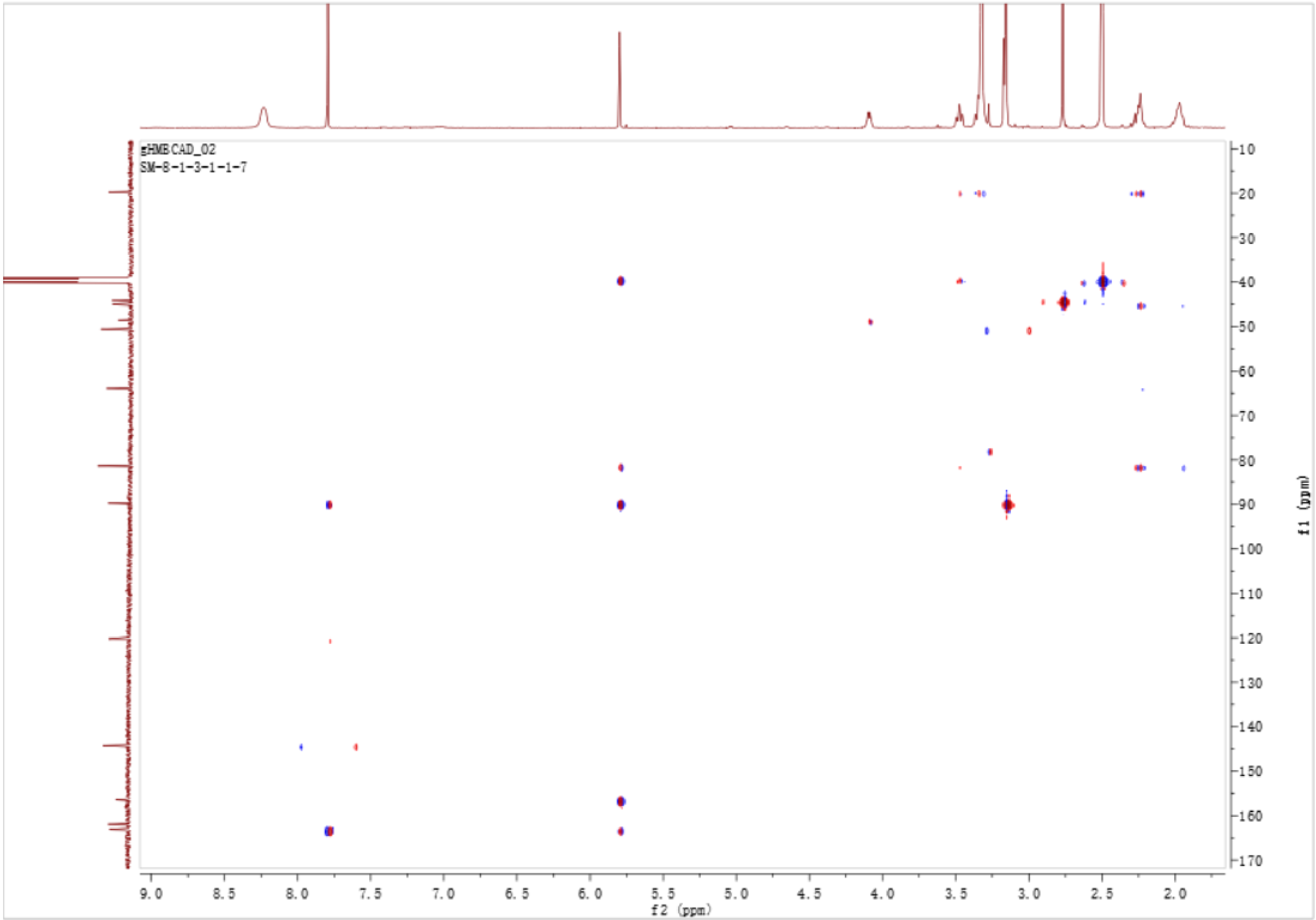

Figure S66. HMBC spectrum of compound **6** in DMSO-d_6 (500 MHz)

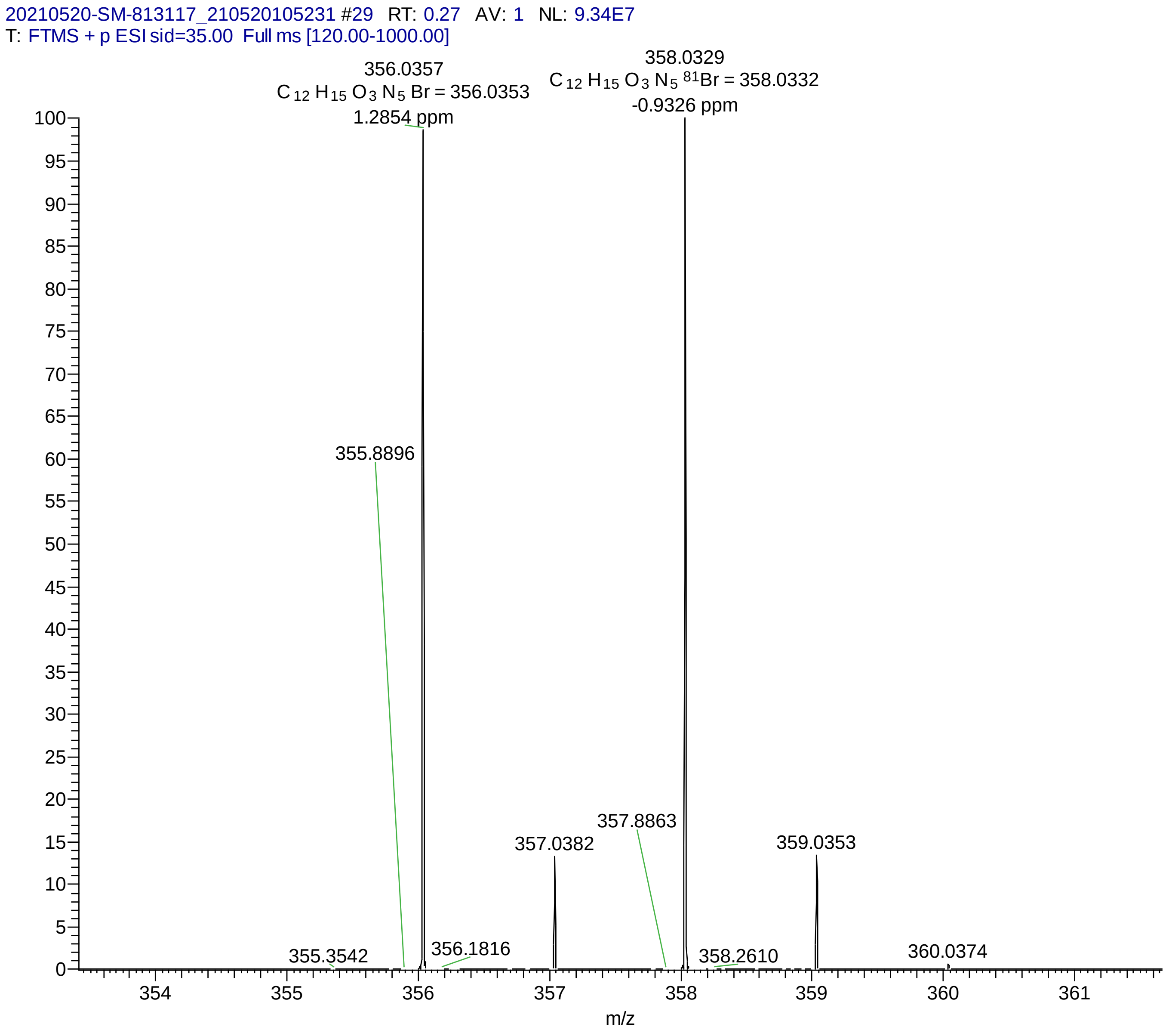

Figure S67. HRESIMS data of compound **6**.

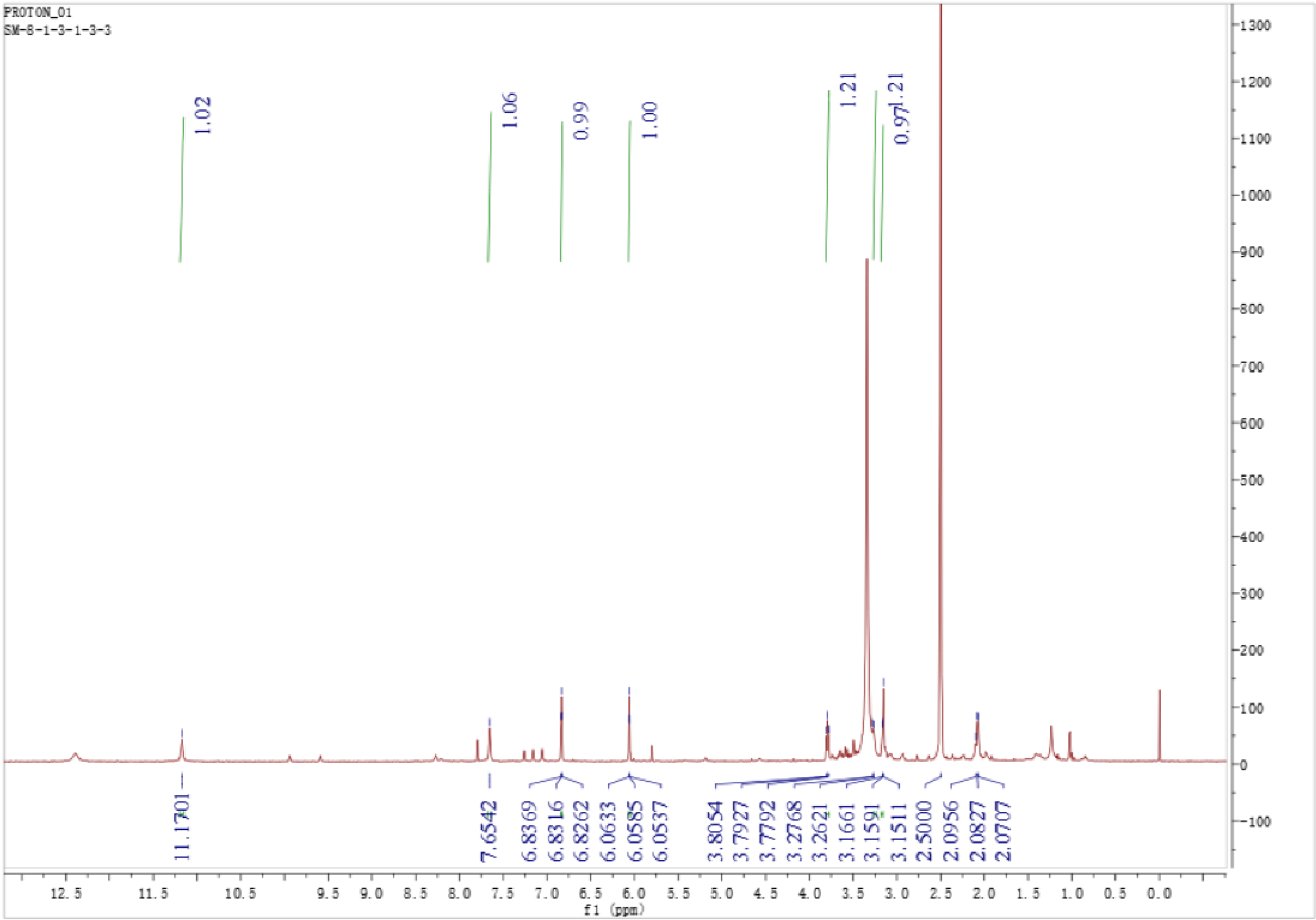

Figure S68. ^{1}H NMR spectrum of compound **7** in DMSO-d_6 (500 MHz).

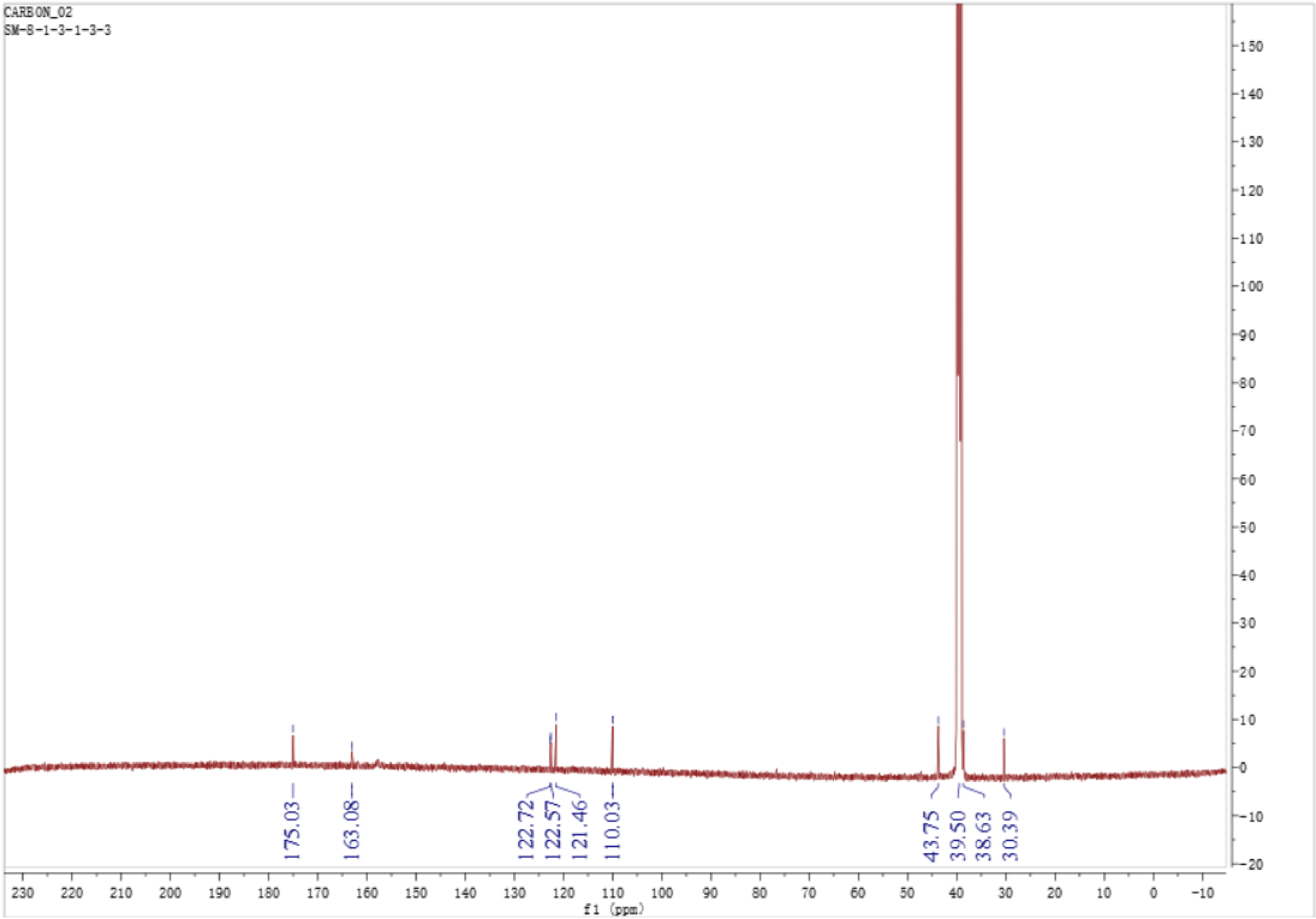

Figure S69. ^{13}C NMR spectrum of compound **7** in DMSO-d_6 (125 MHz).

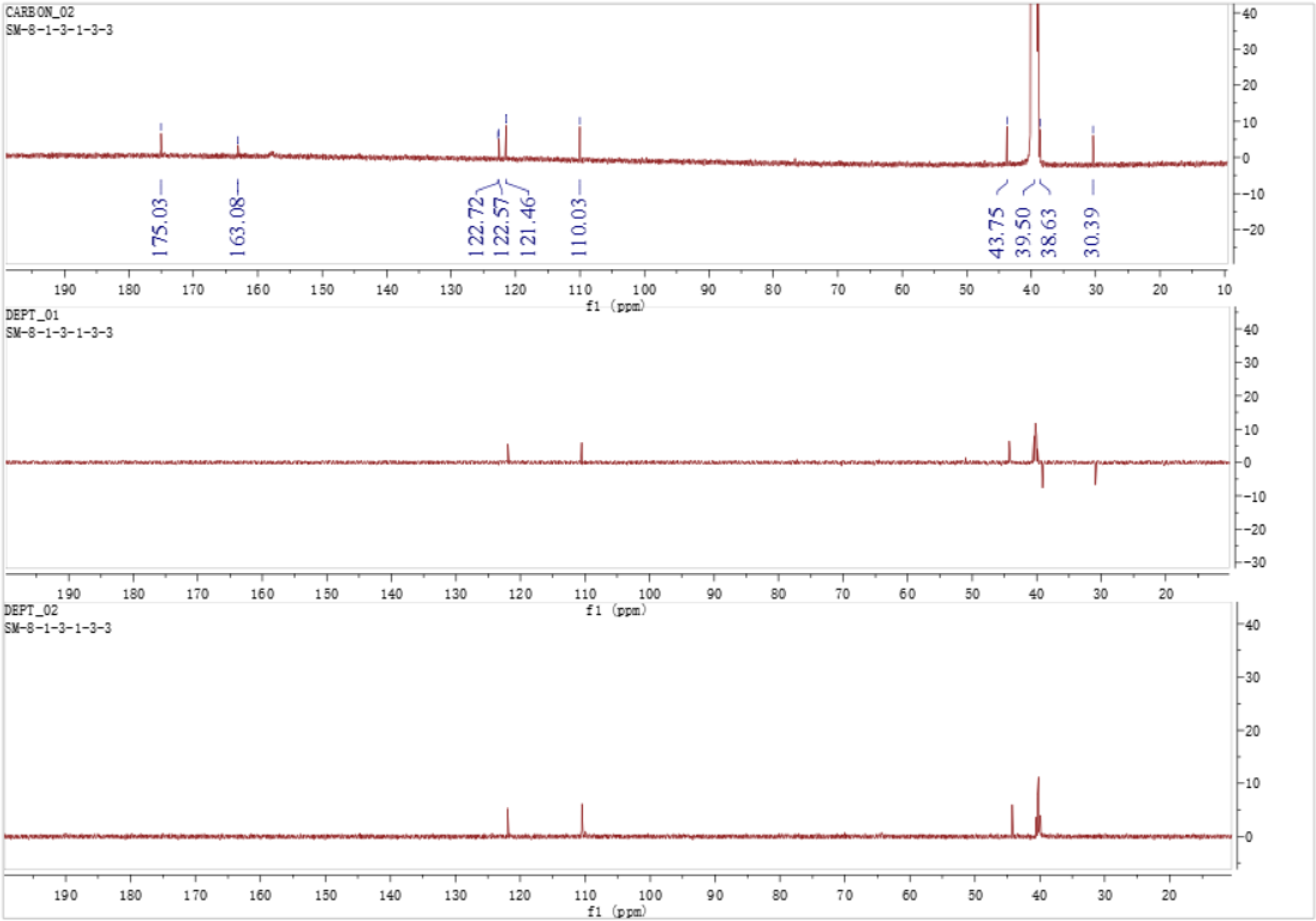

Figure S70. ^{13}C NMR and DEPT spectrum of compound **7** in DMSO-d_6 (125 MHz).

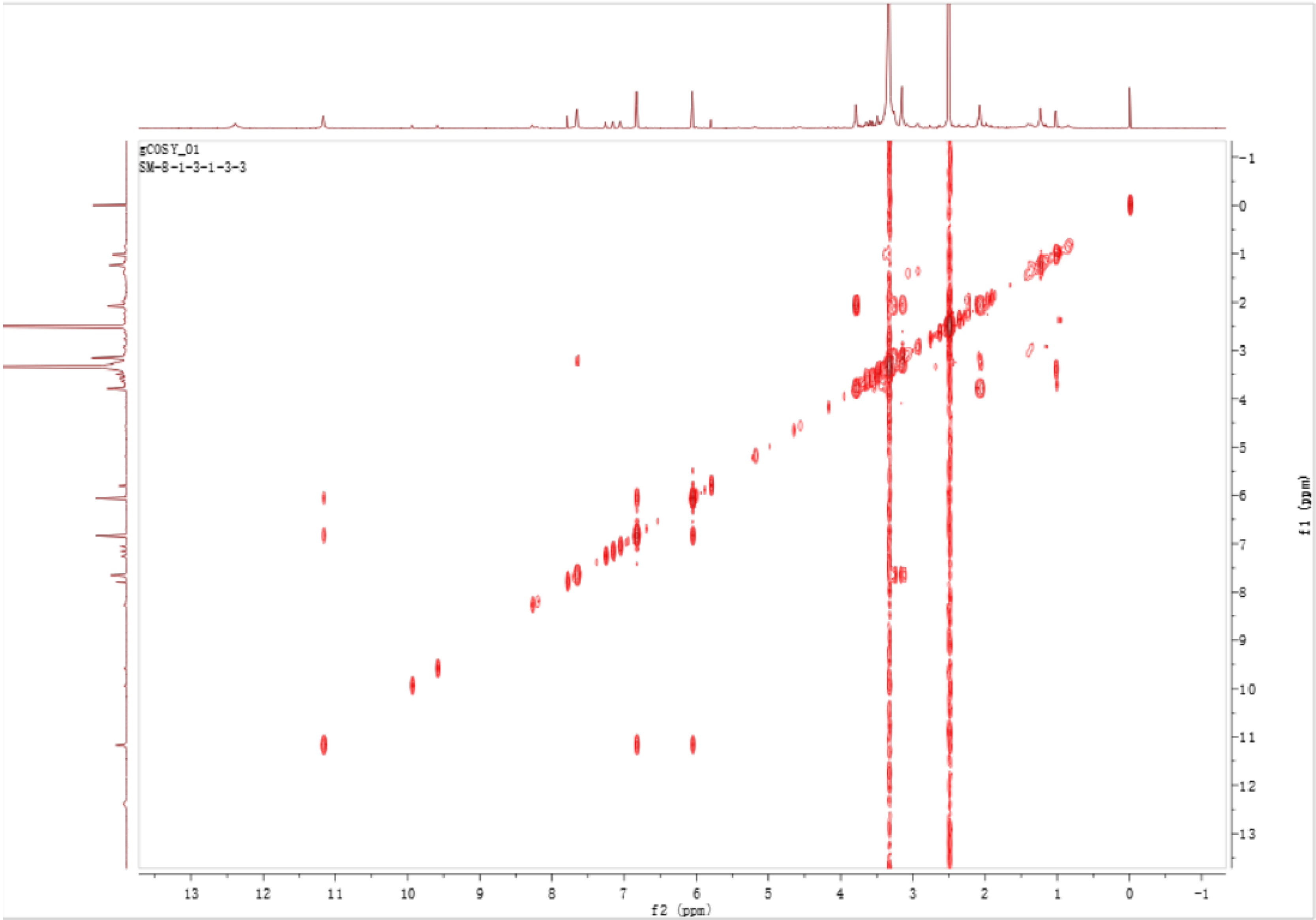

Figure S71. ^{1}H-^{1}H COSY spectrum of compound **7** in DMSO-d_6 (500 MHz)

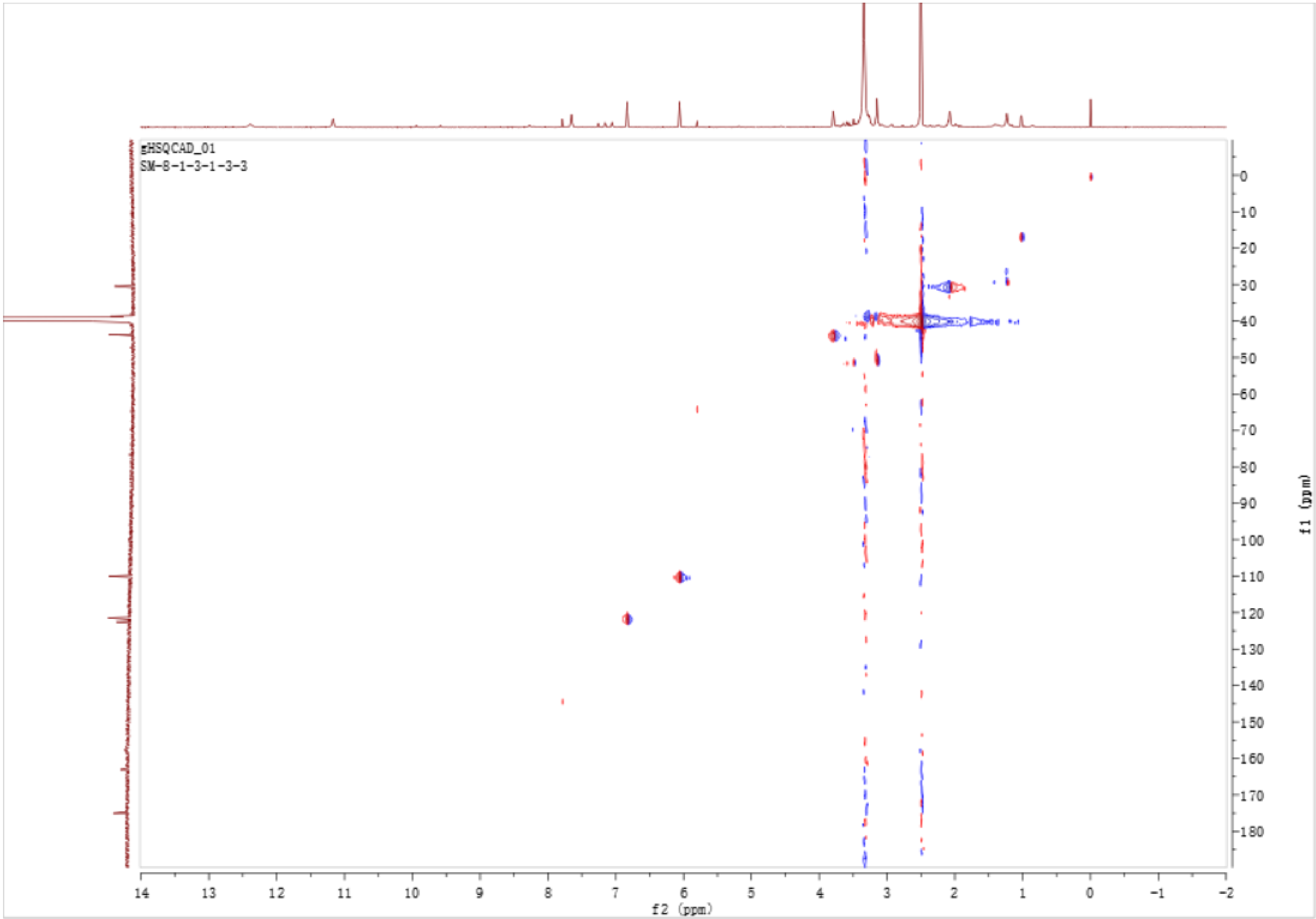

Figure S72. HSQC spectrum of compound **7** in DMSO-d_6 (500 MHz).

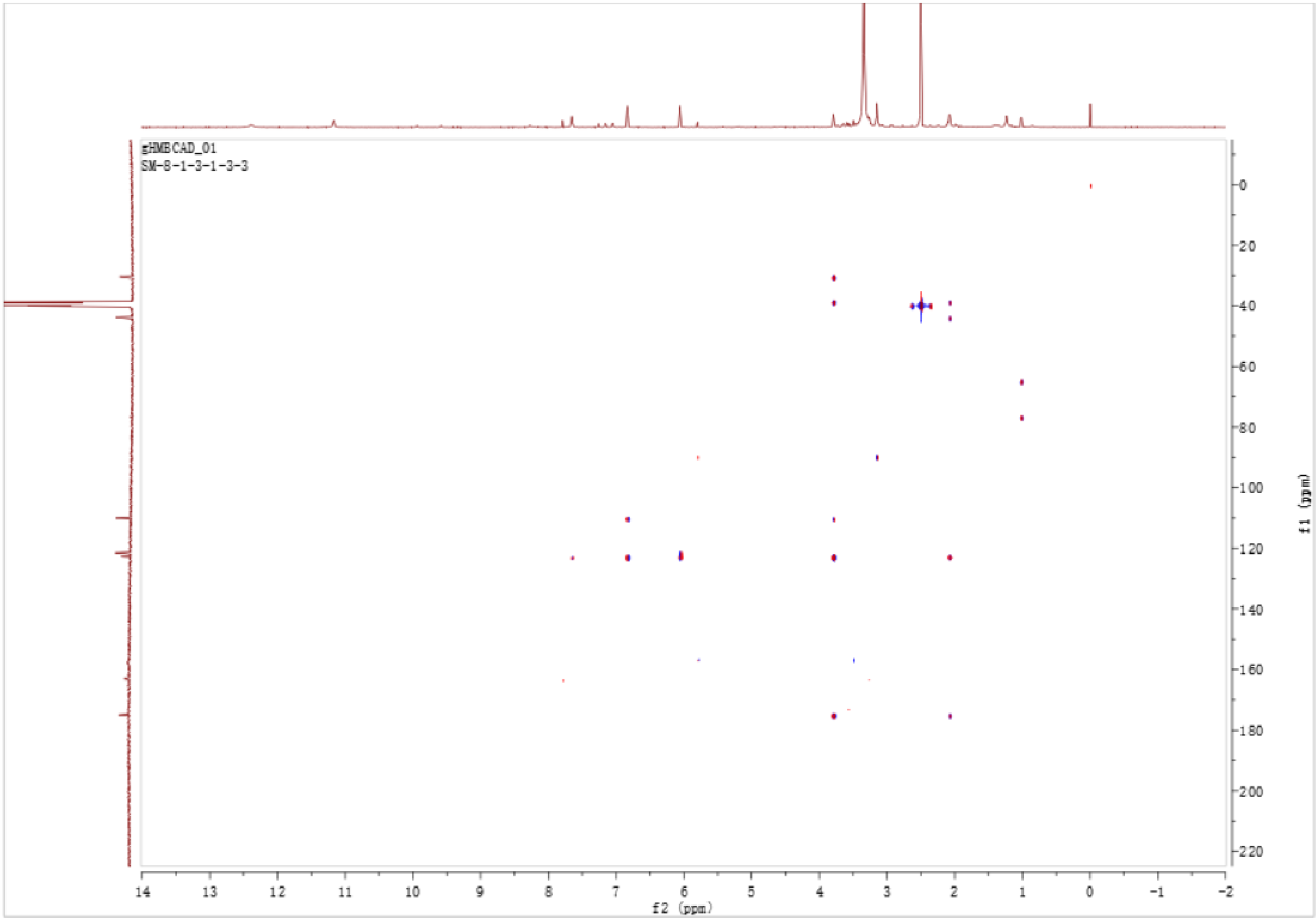

Figure S73. HMBC spectrum of compound **7** in DMSO-d_6 (500 MHz).

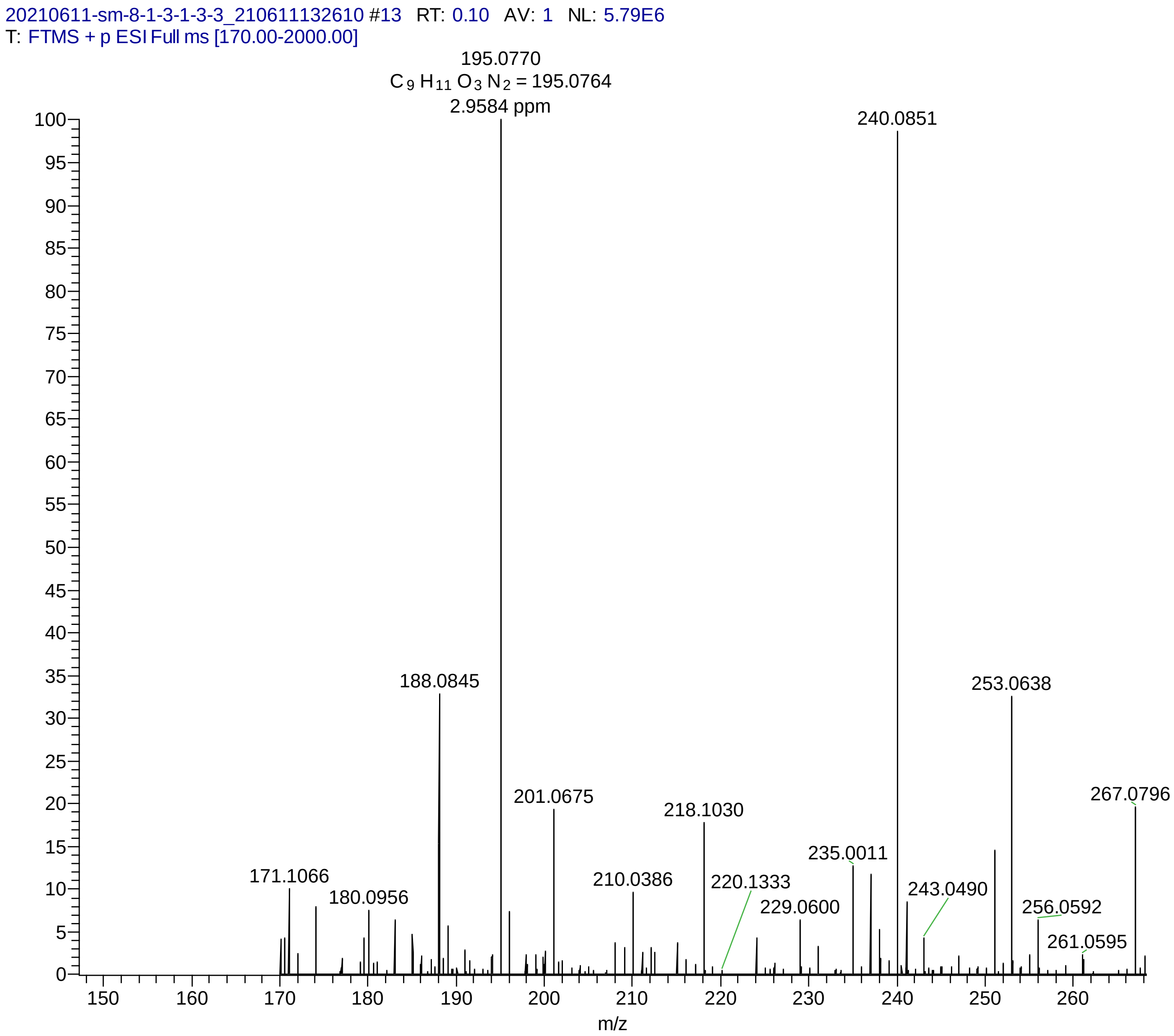

Figure S74. HRESIMS data of compound **7**.

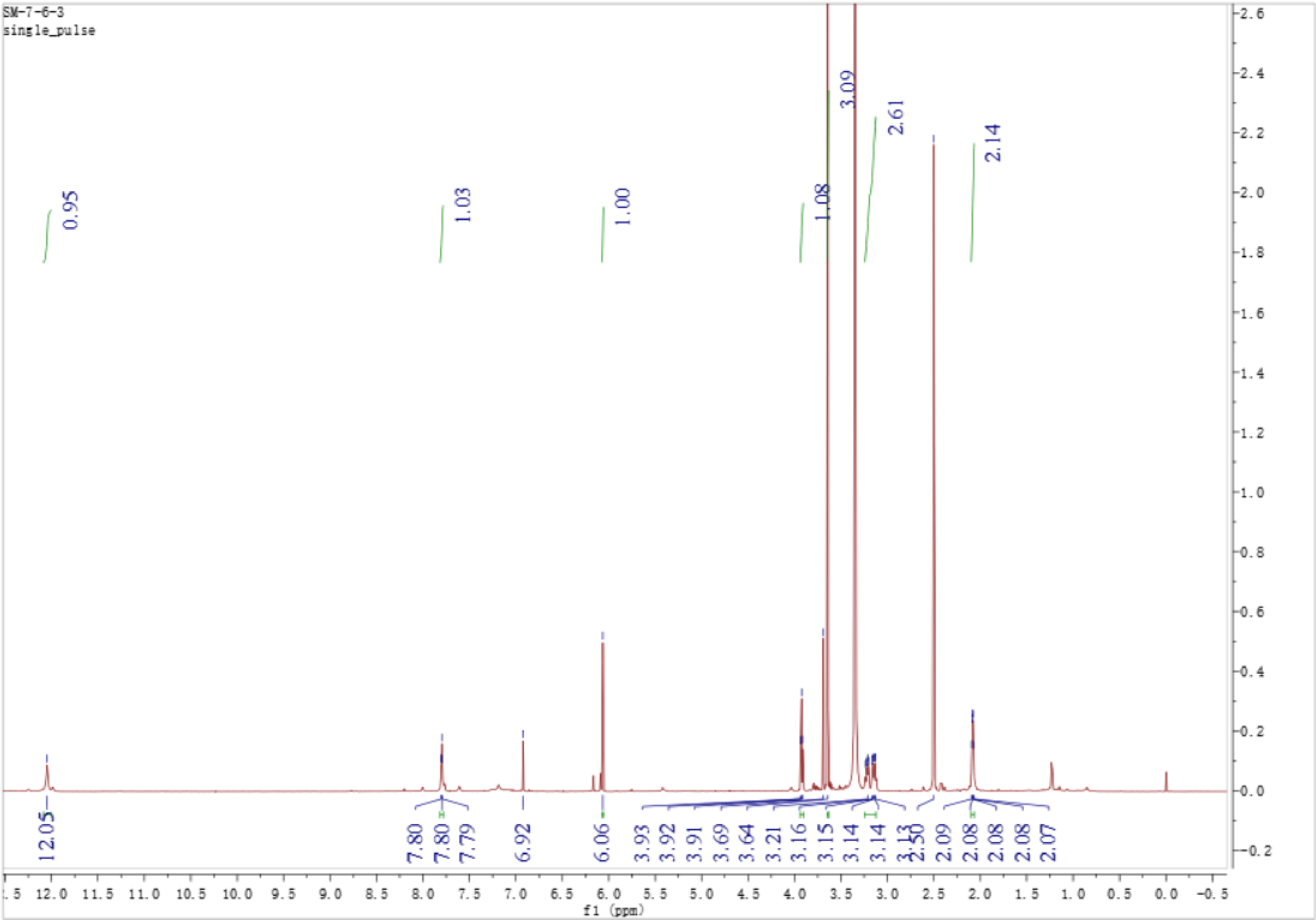

Figure S75. ^{1}H NMR spectrum of compound **8** in DMSO-d_6 (500 MHz).

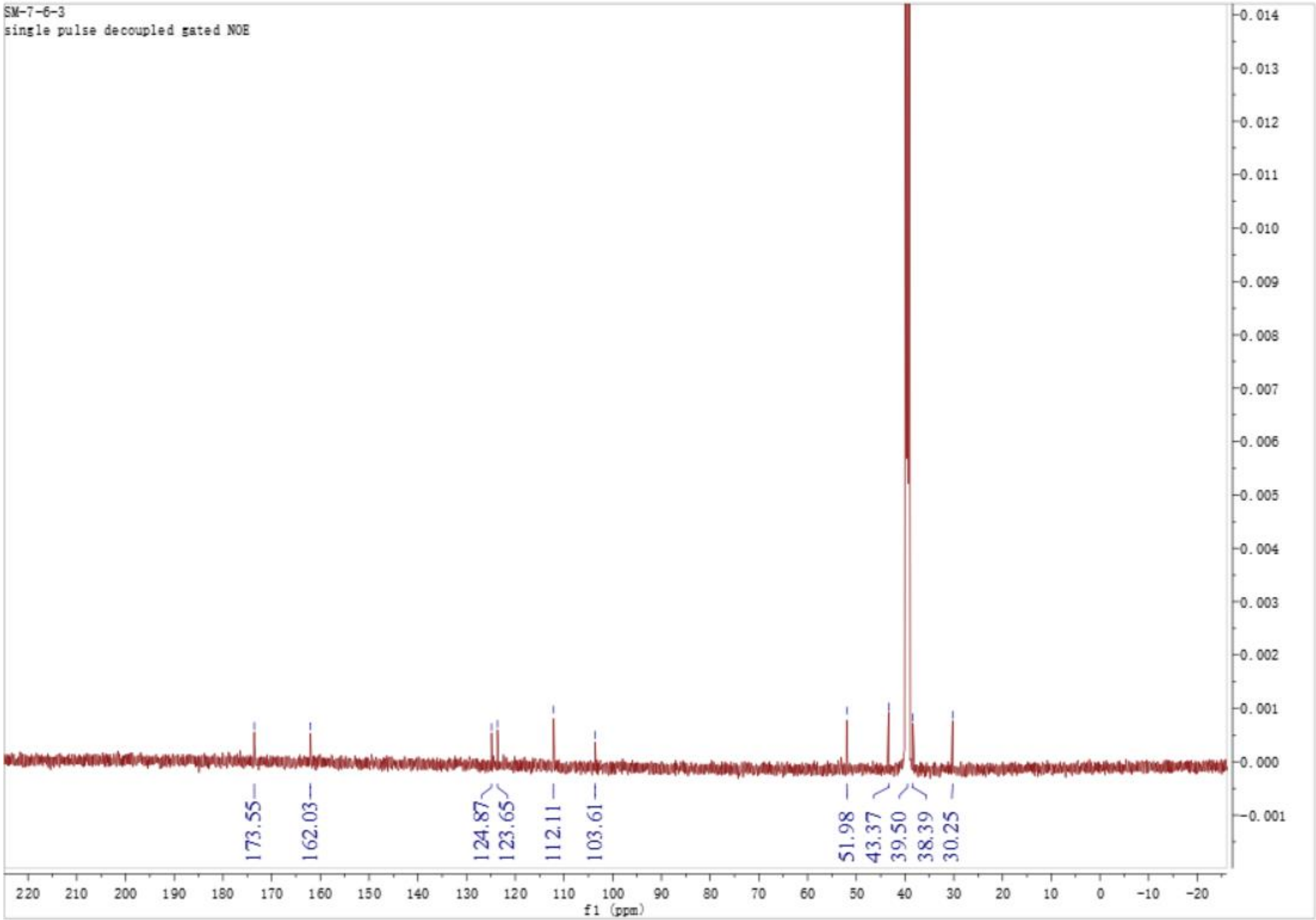

Figure S76. ^{13}C NMR spectrum of compound **8** in DMSO-d_6 (125 MHz).

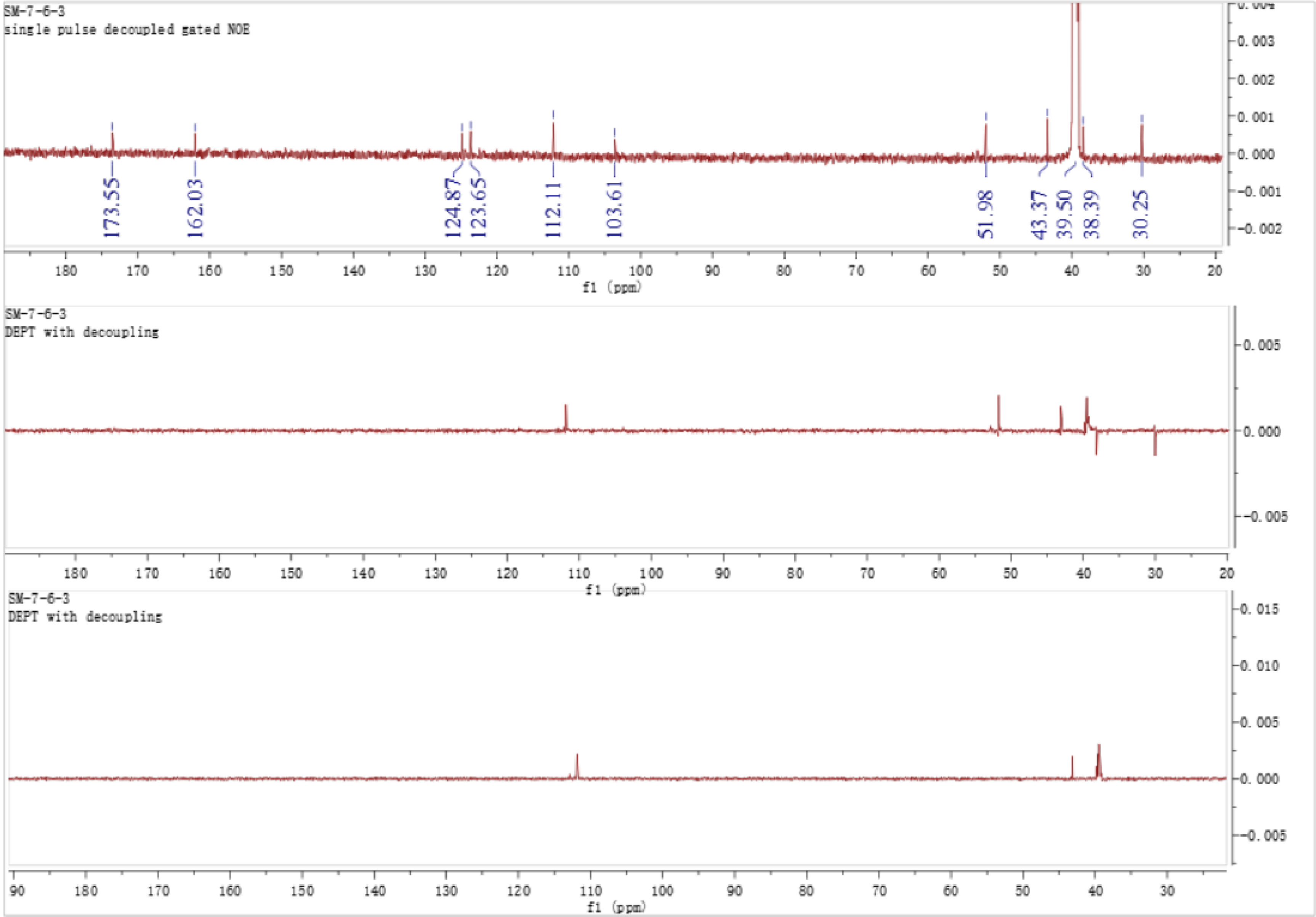

Figure S77. ^{13}C NMR and DEPT spectrum of compound **8** in DMSO-d_6 (125 MHz)

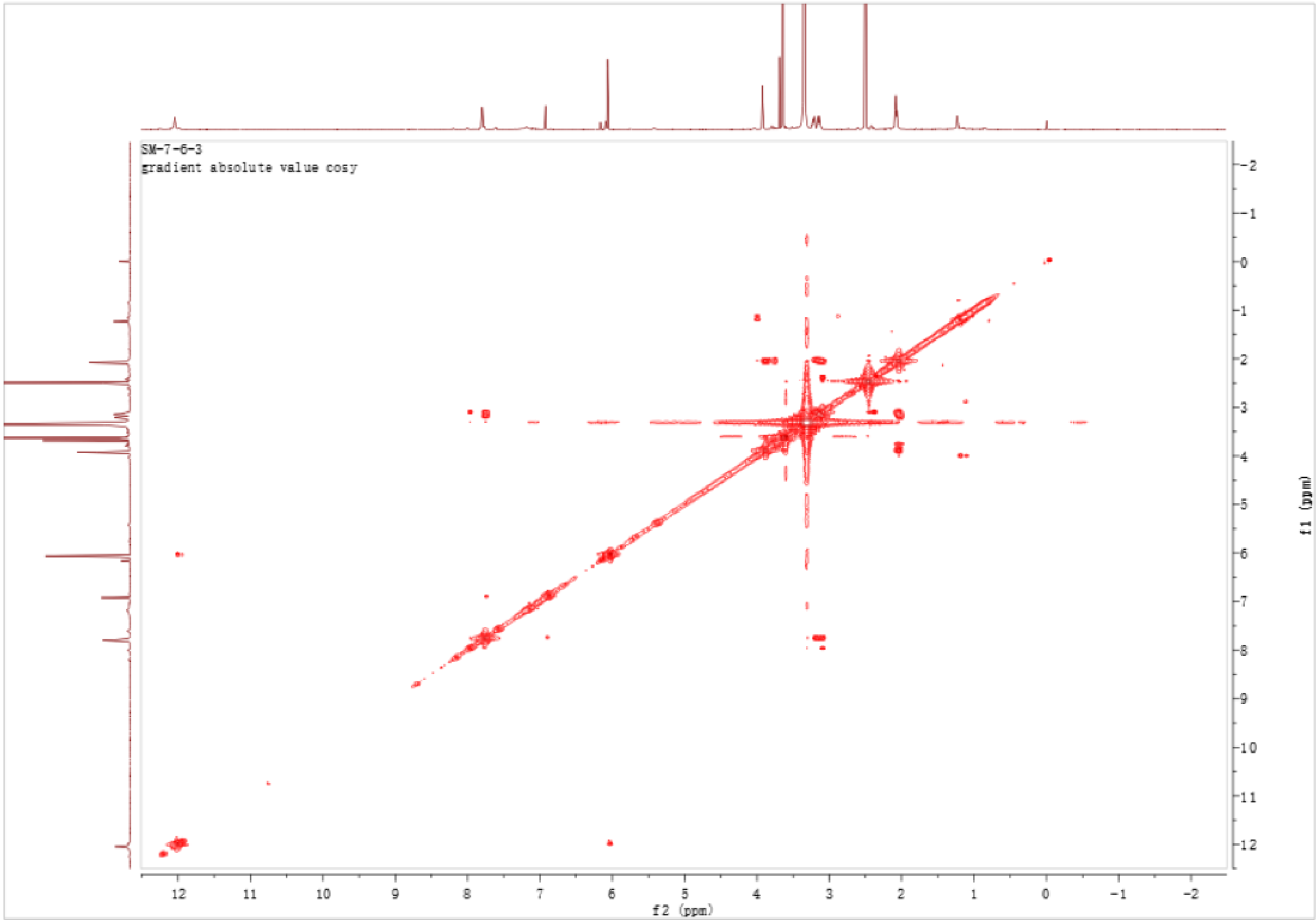

Figure S78. ^{1}H-^{1}H COSY spectrum of compound **8** in DMSO-d_6 (500 MHz).

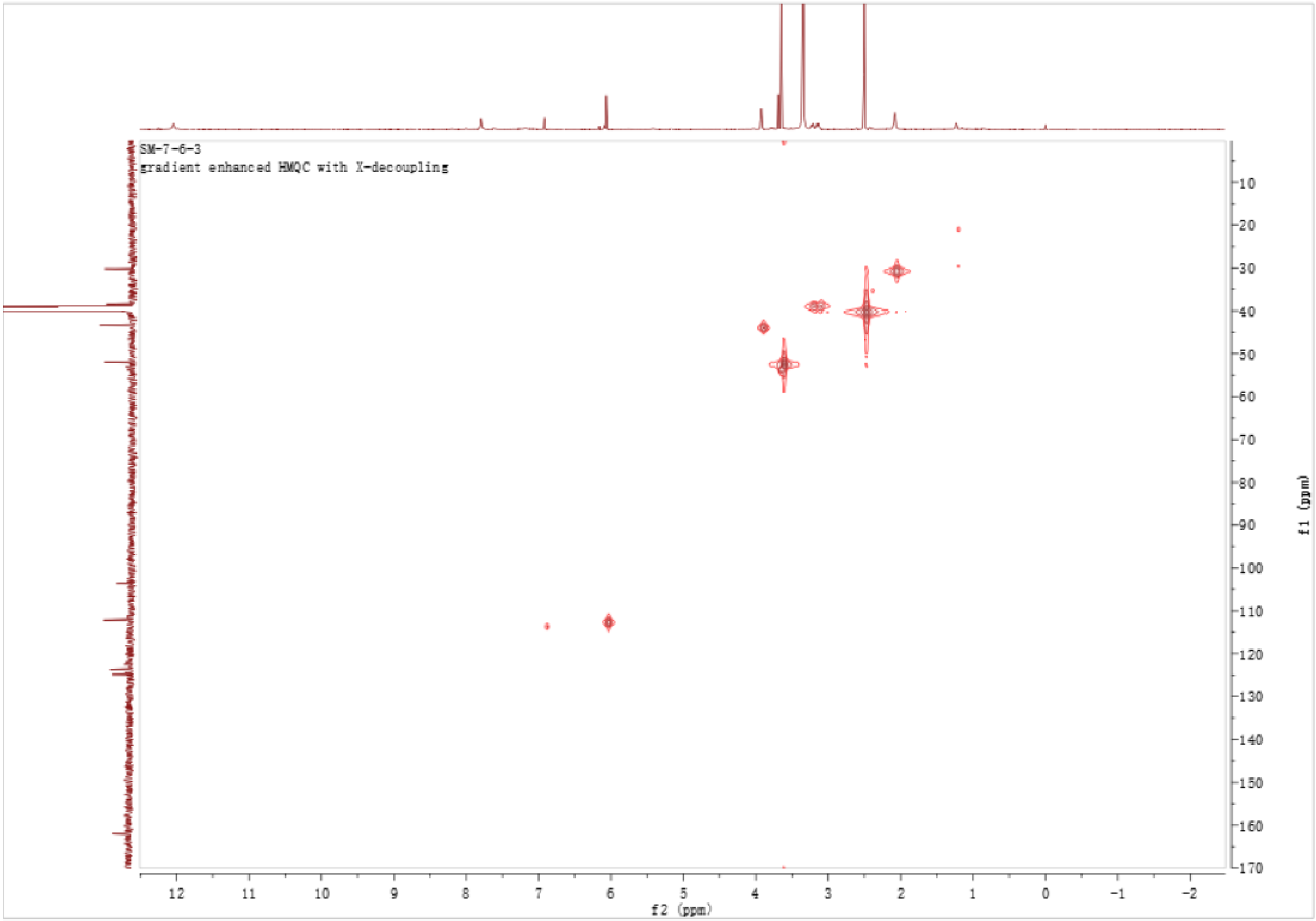

Figure S79. HSQC spectrum of compound **8** in DMSO-d_6 (500 MHz).

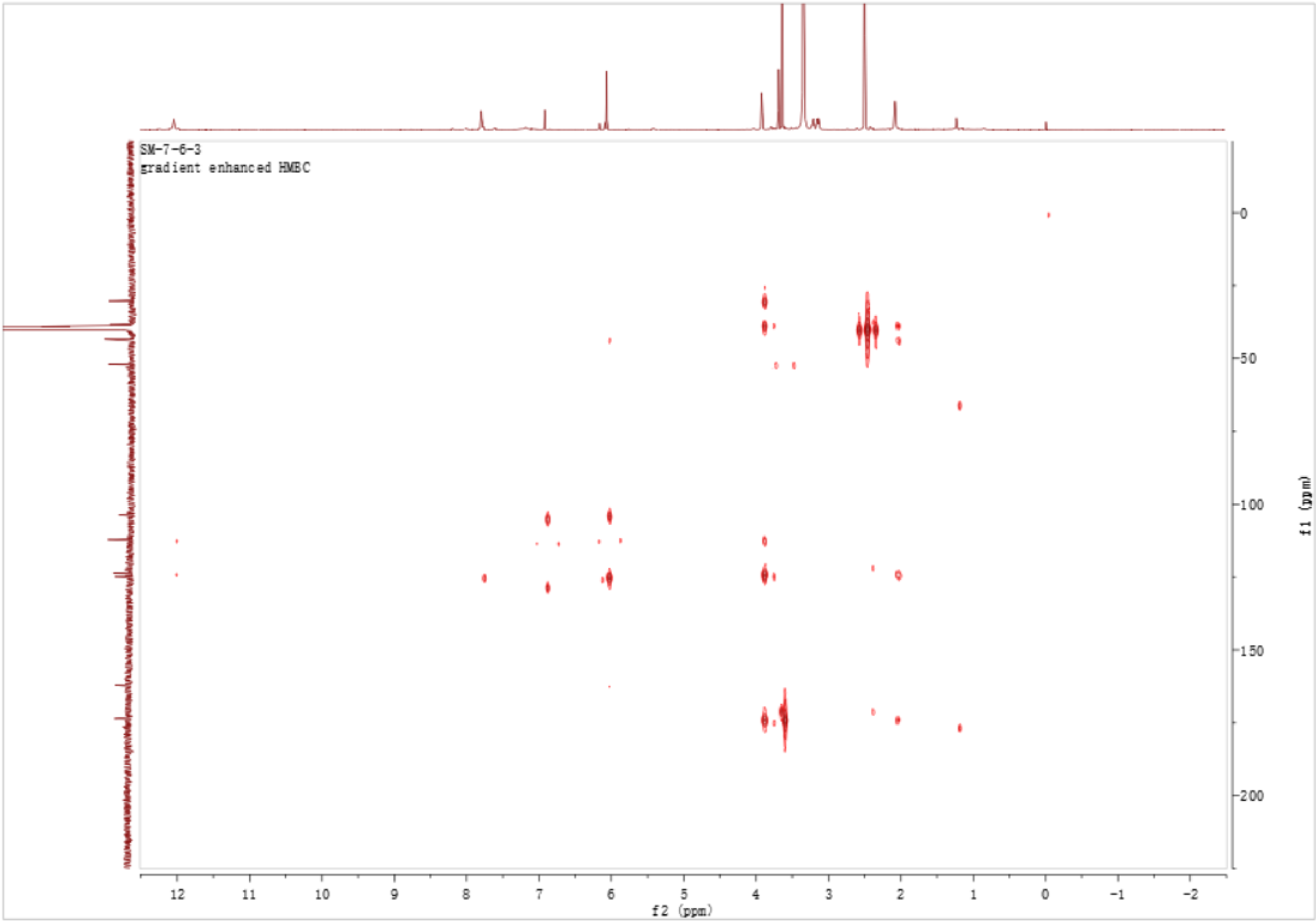

Figure S80. HMBC spectrum of compound **8** in DMSO-d_6 (500 MHz).

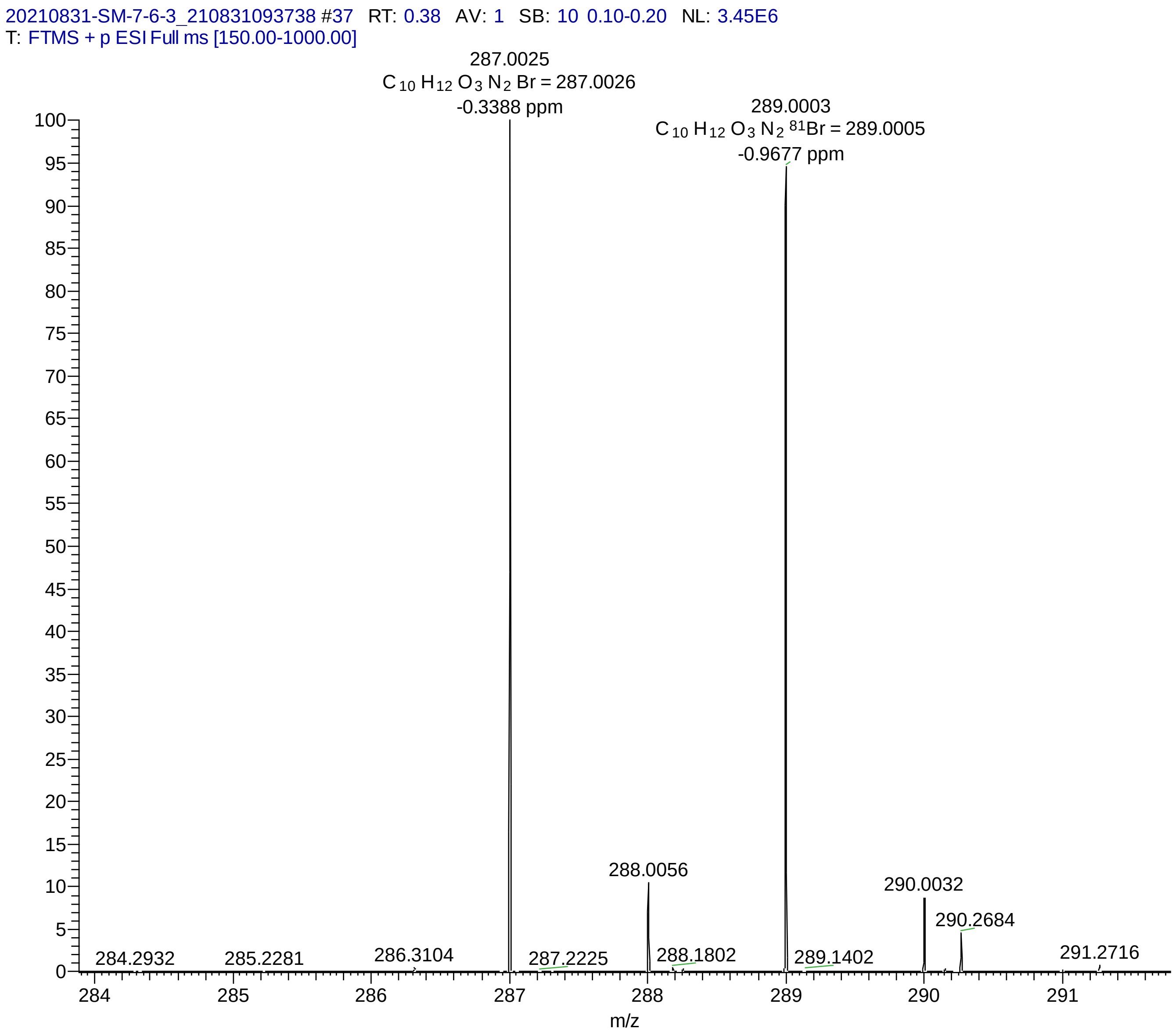

Figure S81. HRESIMS data of compound **8**.

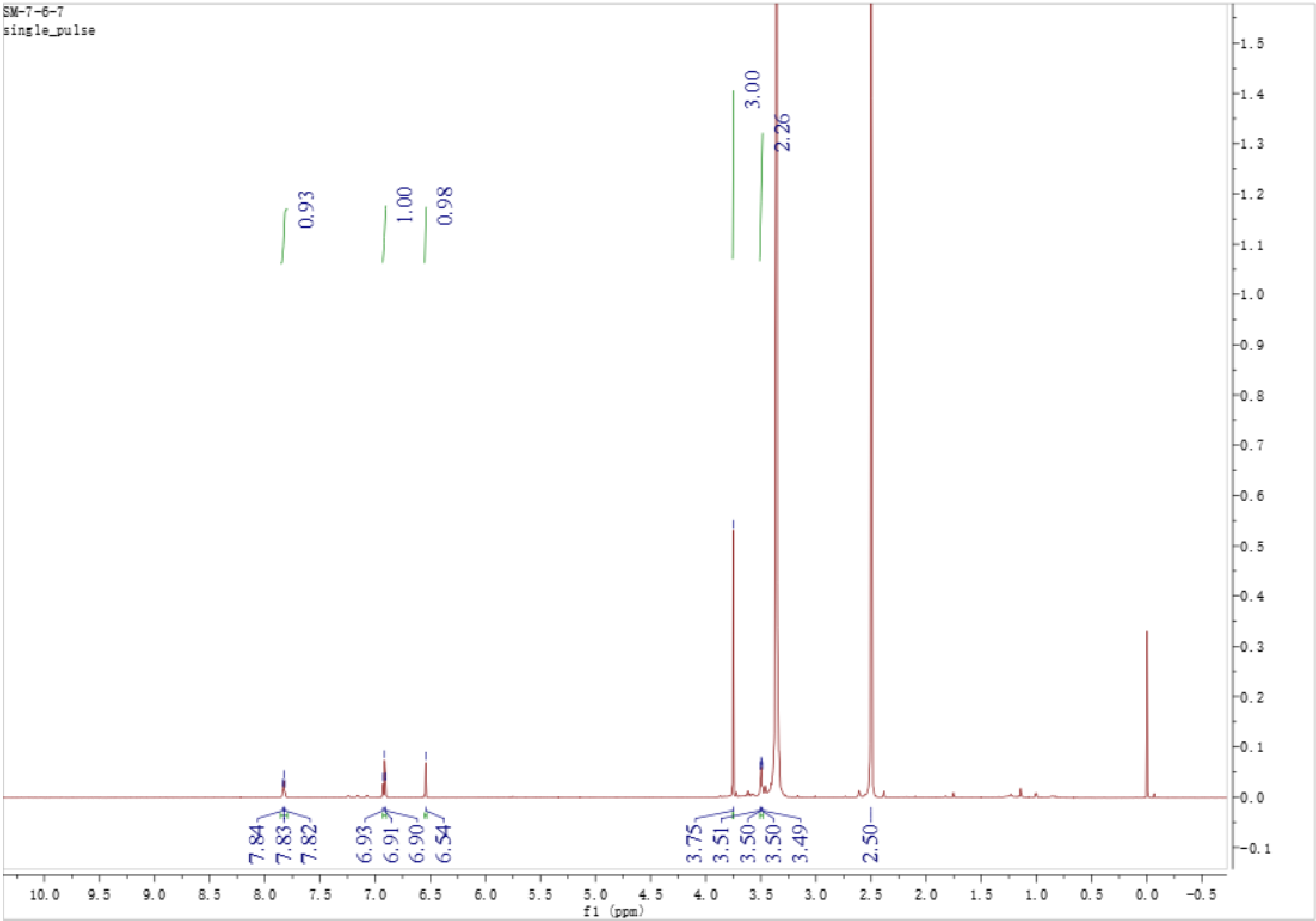

Figure S82. ^{1}H NMR spectrum of compound **9** in DMSO-d_6 (500 MHz).

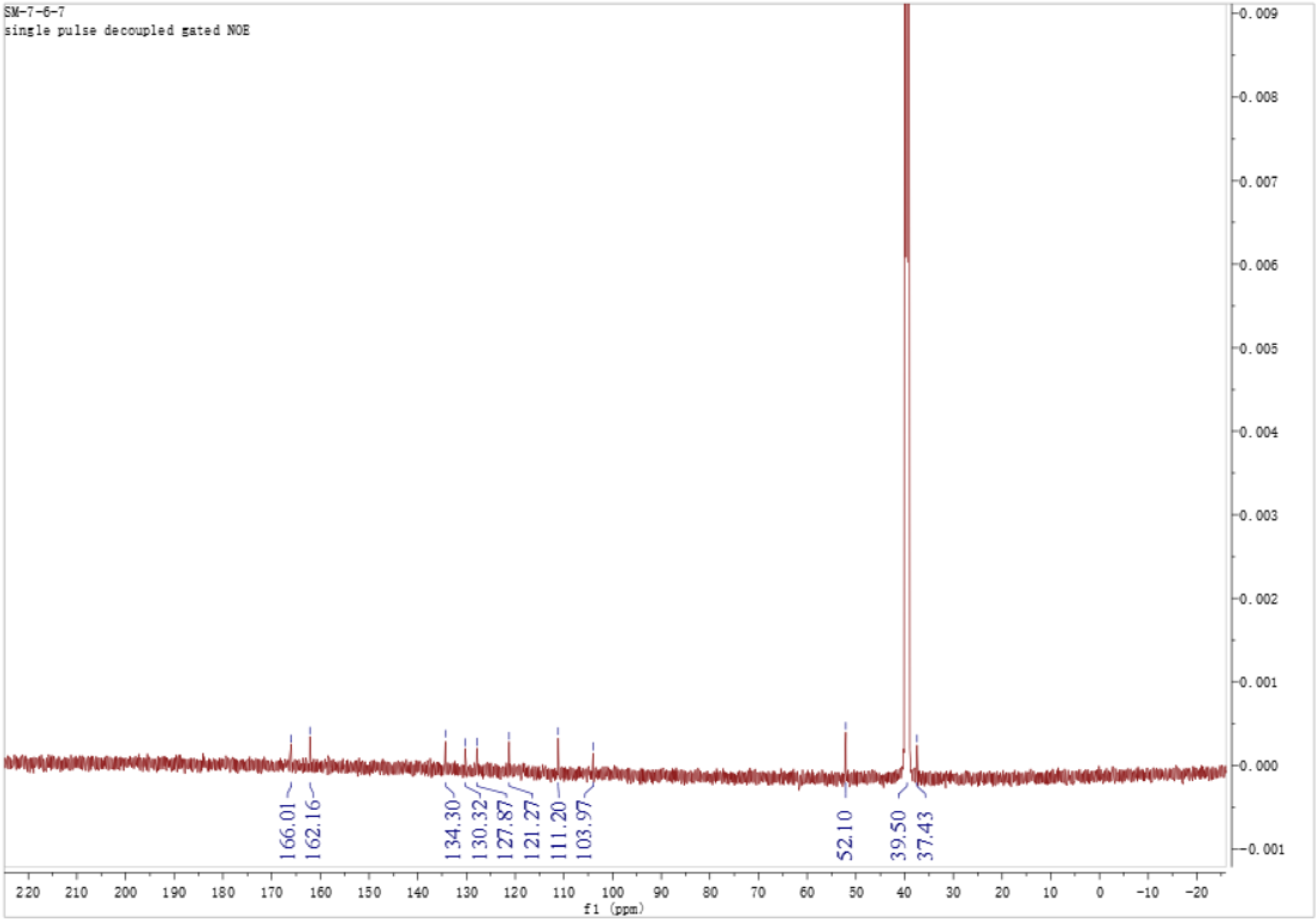

Figure S83. ^{13}C NMR spectrum of compound **9** in DMSO-d_6 (125 MHz).

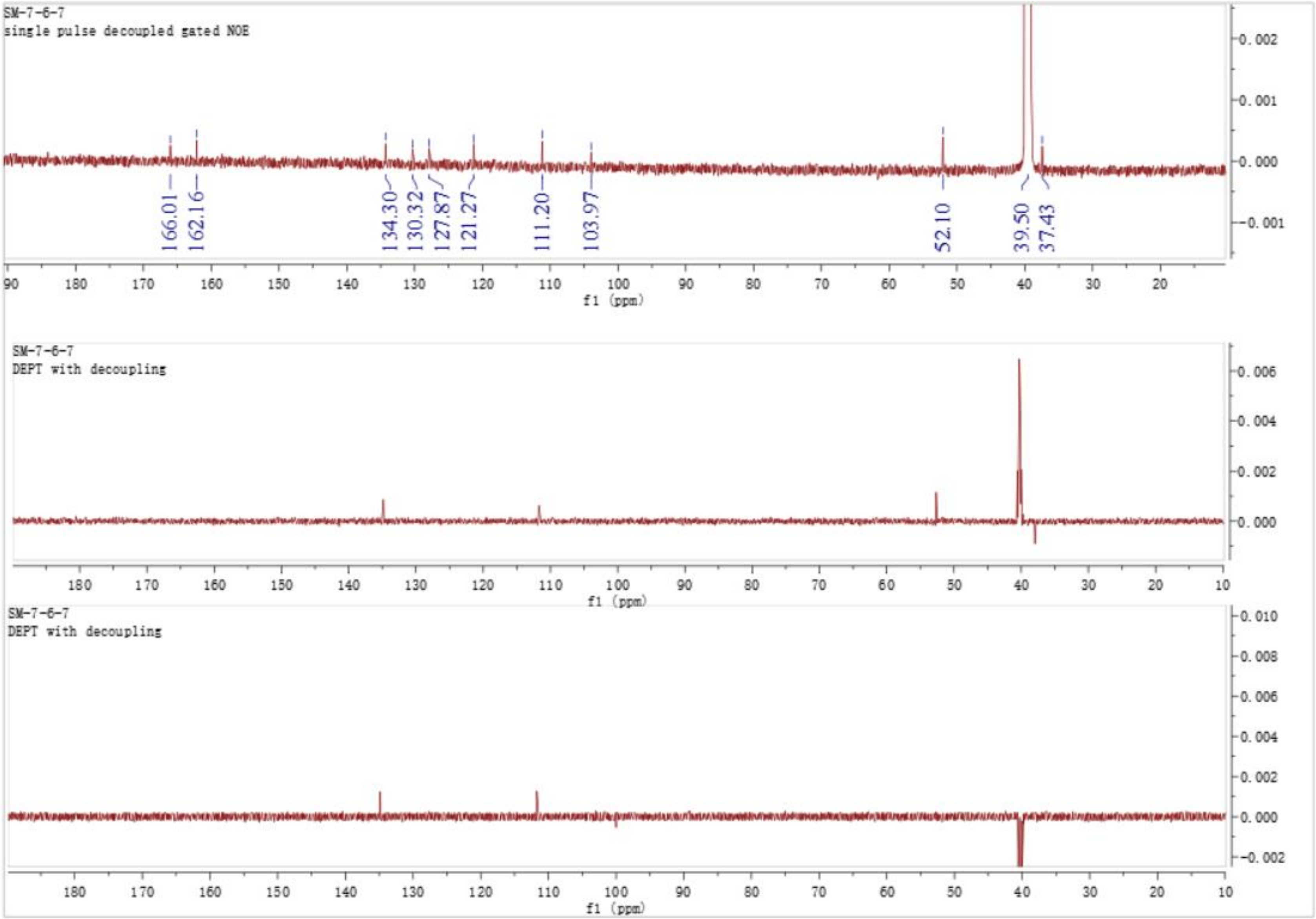

Figure S84. ^{13}C NMR and DEPT spectrum of compound **9** in DMSO-d_6 (125 MHz).

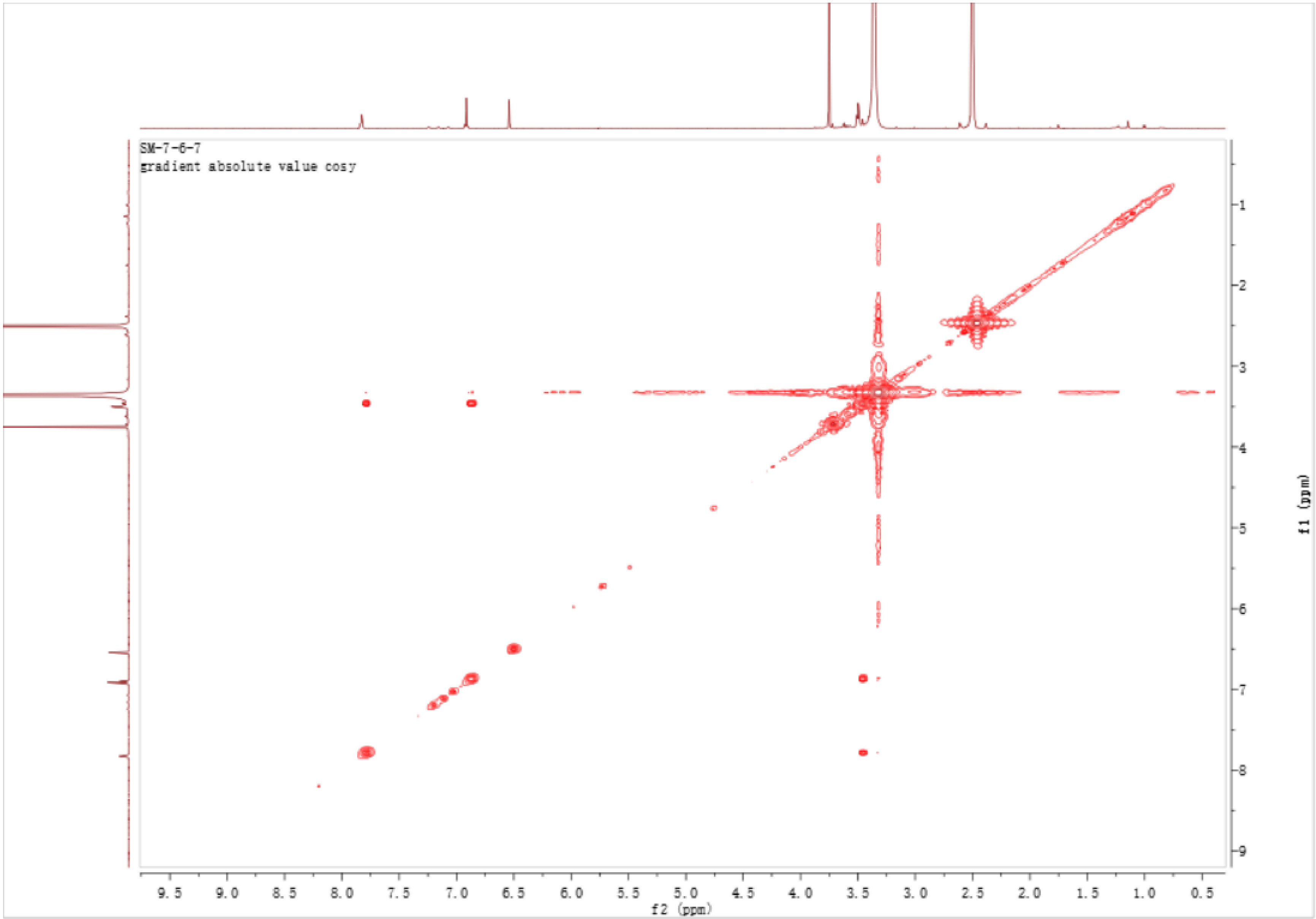

Figure S85. ^{1}H-^{1}H COSY spectrum of compound **9** in DMSO-d_6 (500 MHz).

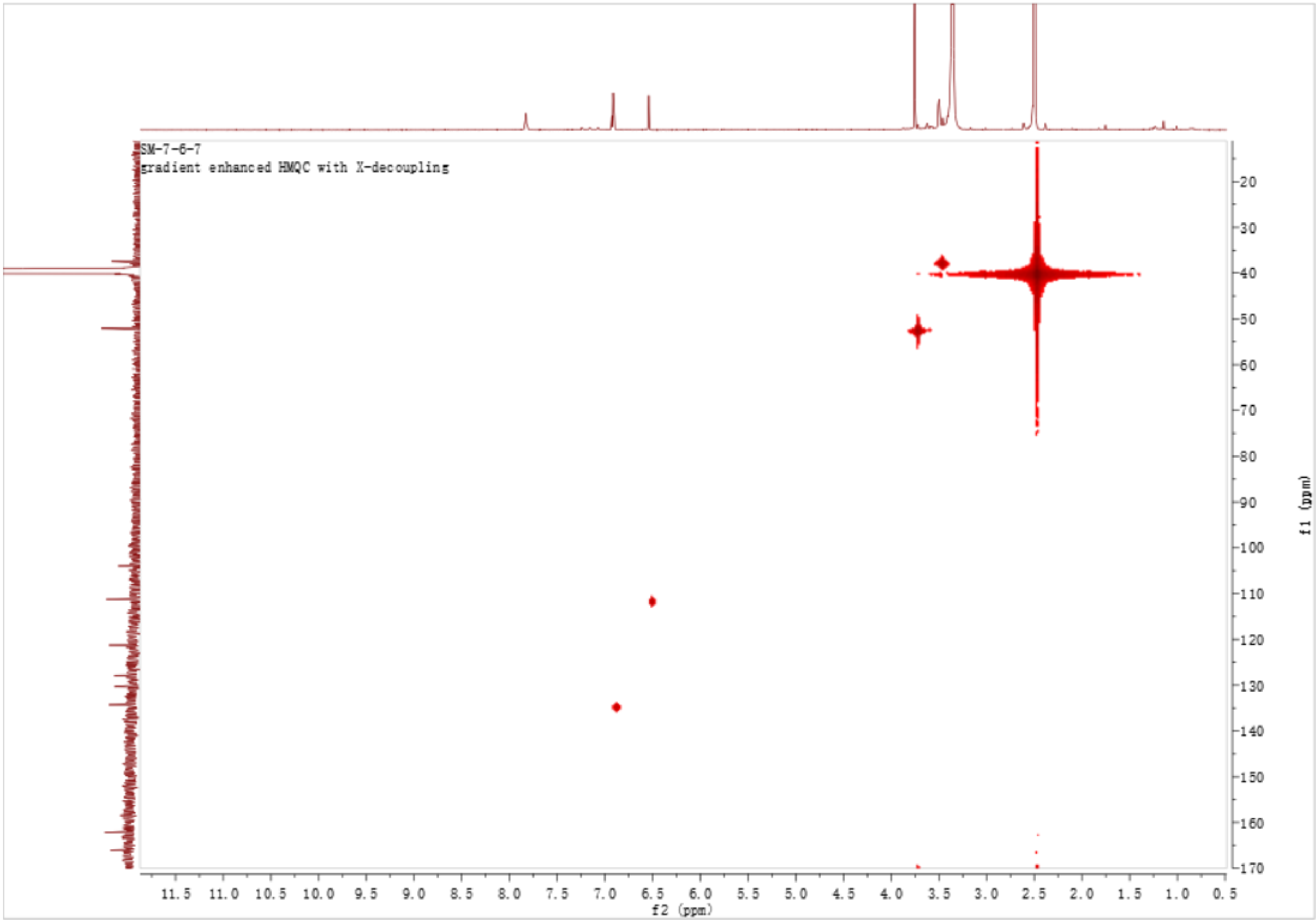

Figure S86. HSQC spectrum of compound **9** in DMSO-d_6 (500 MHz).

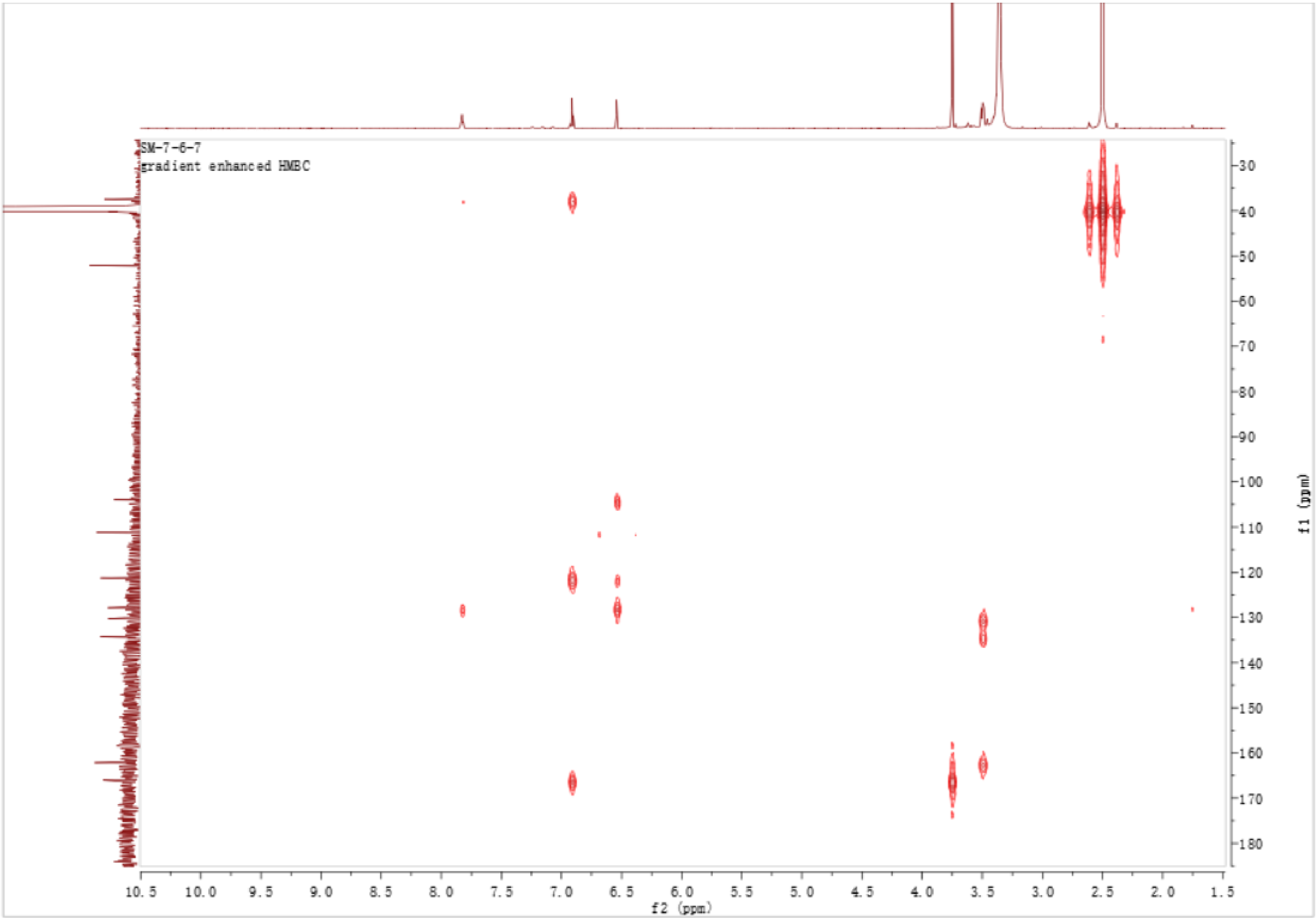

Figure S87. HMBC spectrum of compound **9** in DMSO-d_6 (500 MHz).

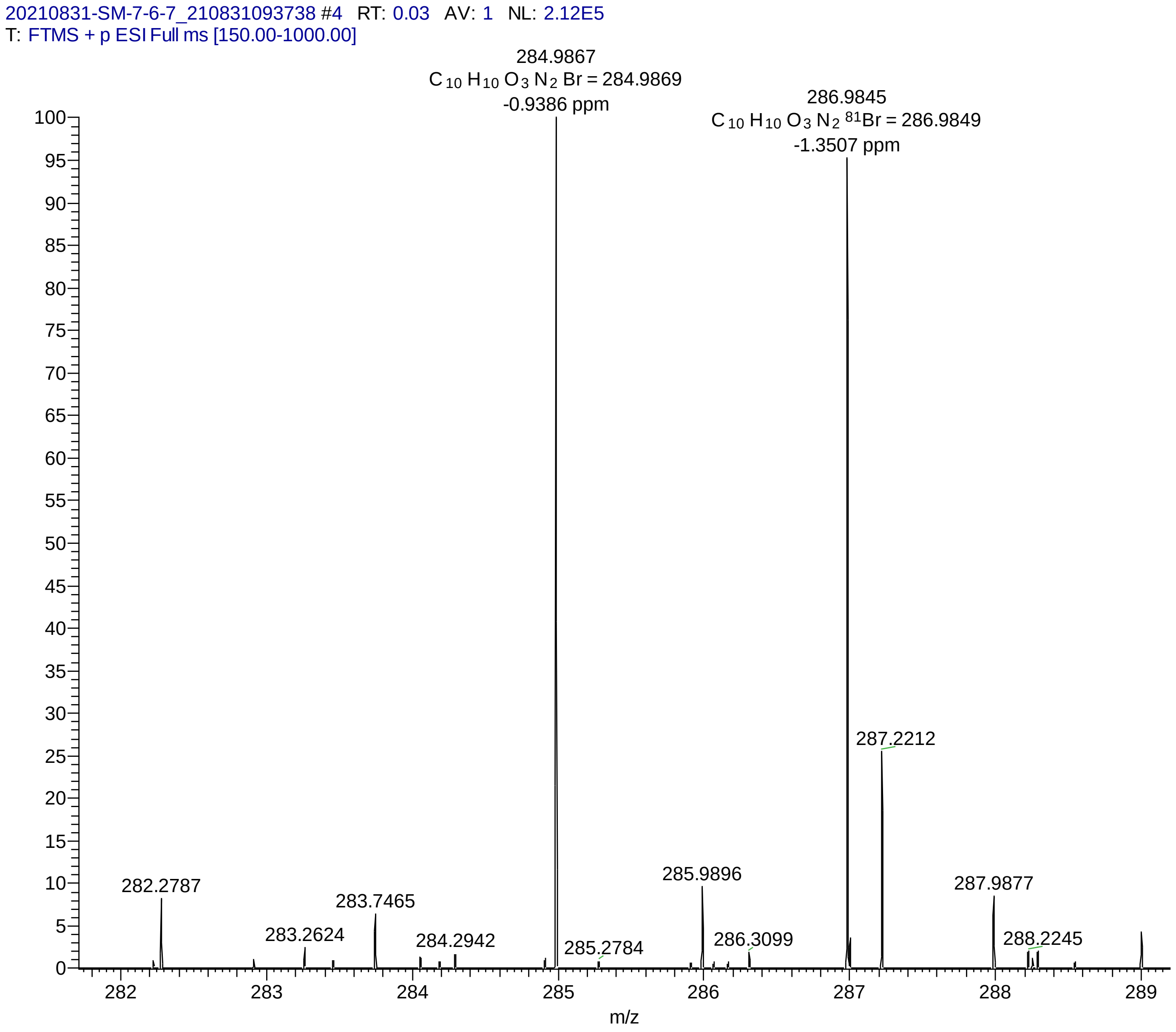

Figure S88. HRESIMS data of compound **9**.

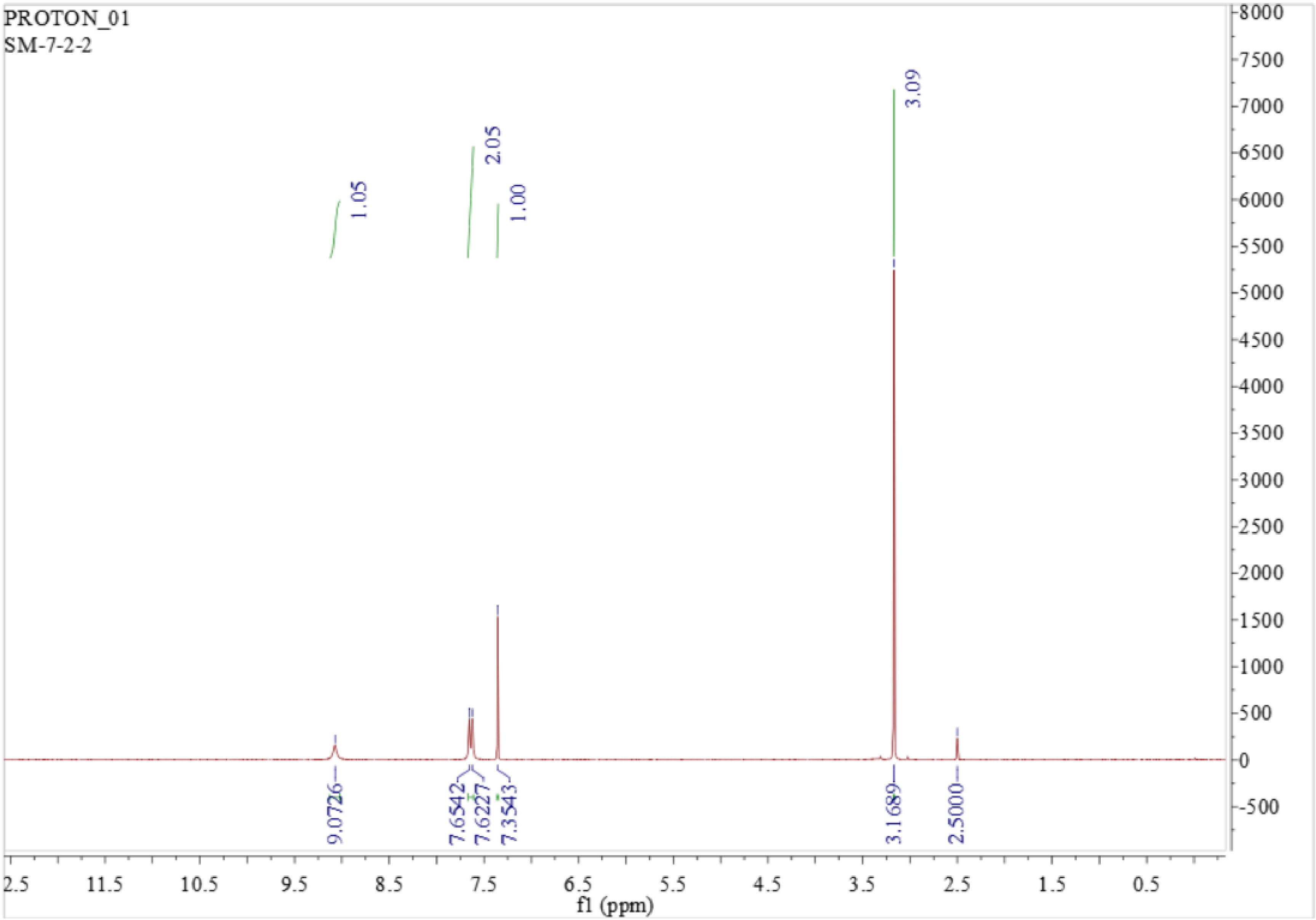

Figure S89. ^{1}H NMR spectrum of compound **10** in DMSO-d_6 (500 MHz).

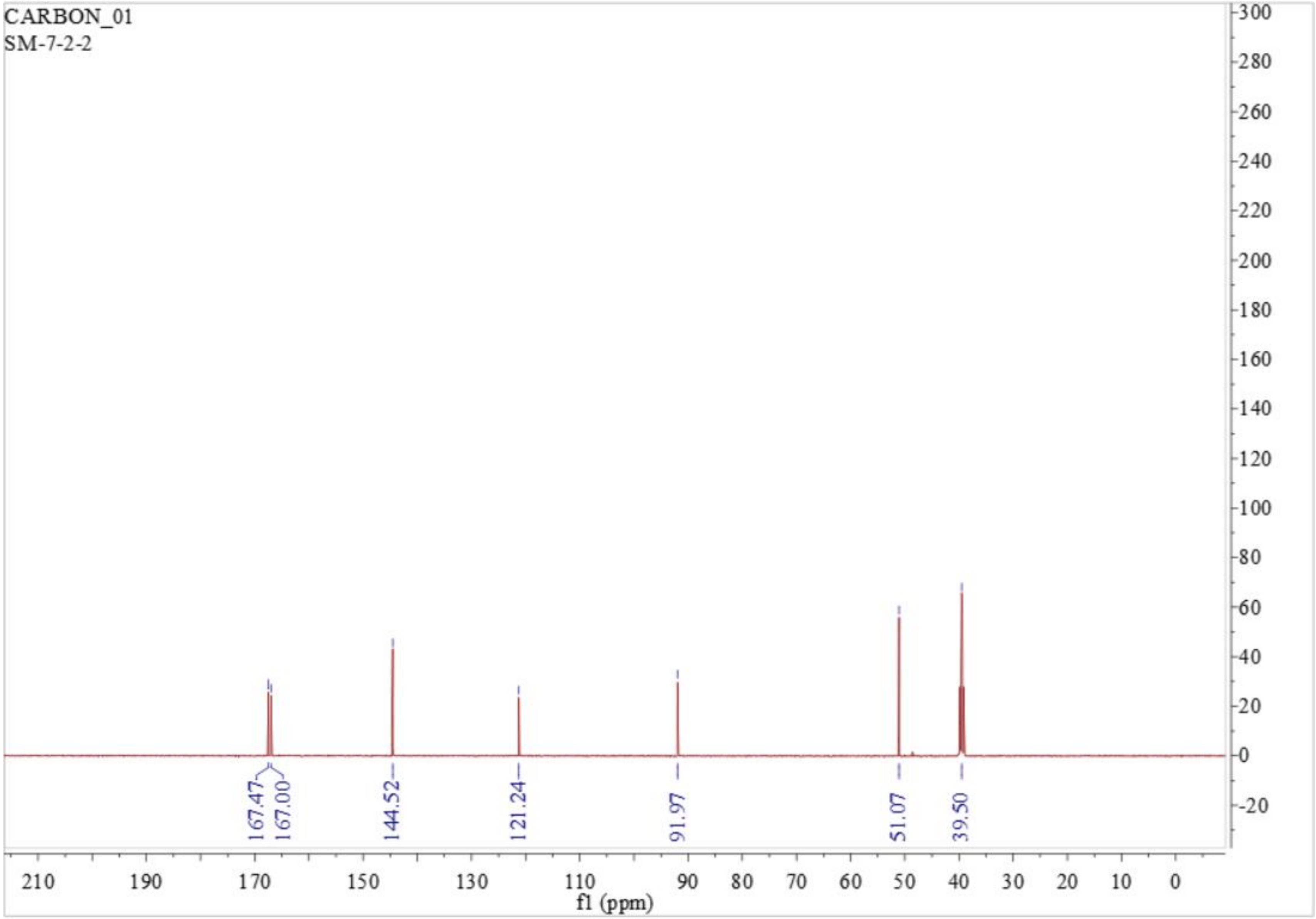

Figure S90. ^{13}C NMR spectrum of compound **10** in DMSO-d_6 (125 MHz).

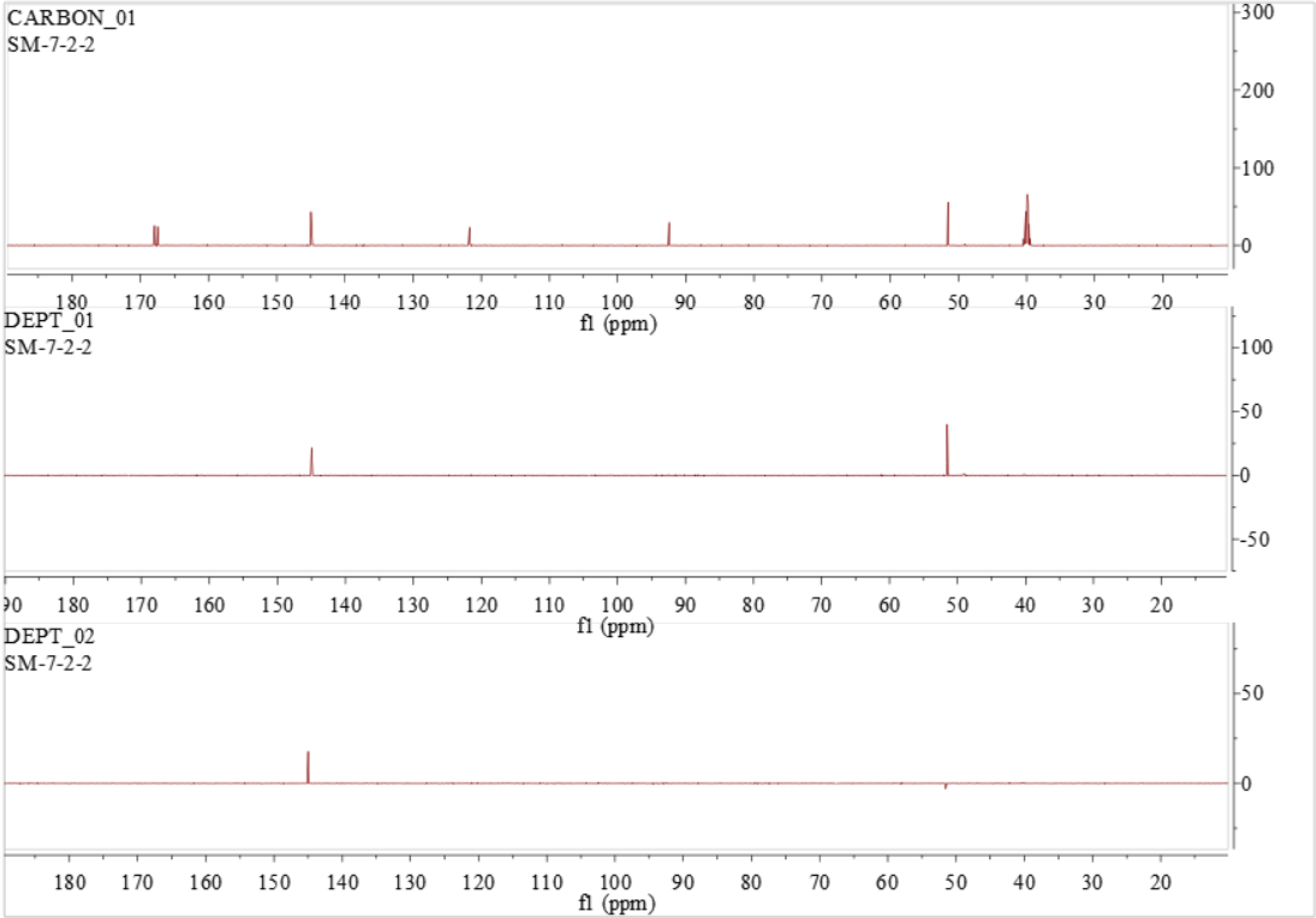

Figure S91. ^{13}C NMR and DEPT spectrum of compound **10** in DMSO-d_6 (125 MHz).

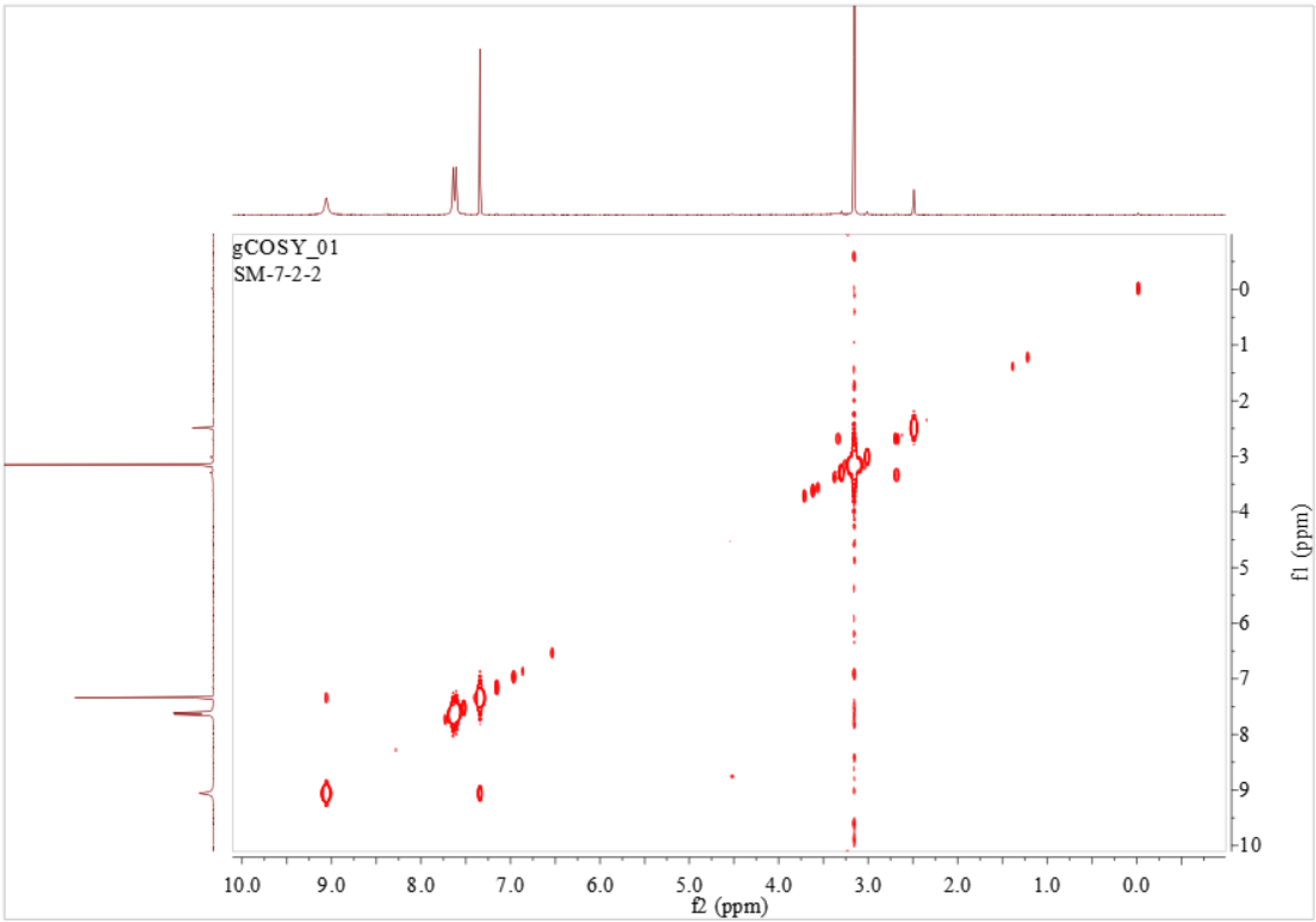

Figure S92. ${}^{1}H$-${}^{1}H$ COSY spectrum of compound **10** in DMSO-d_6 (500 MHz).

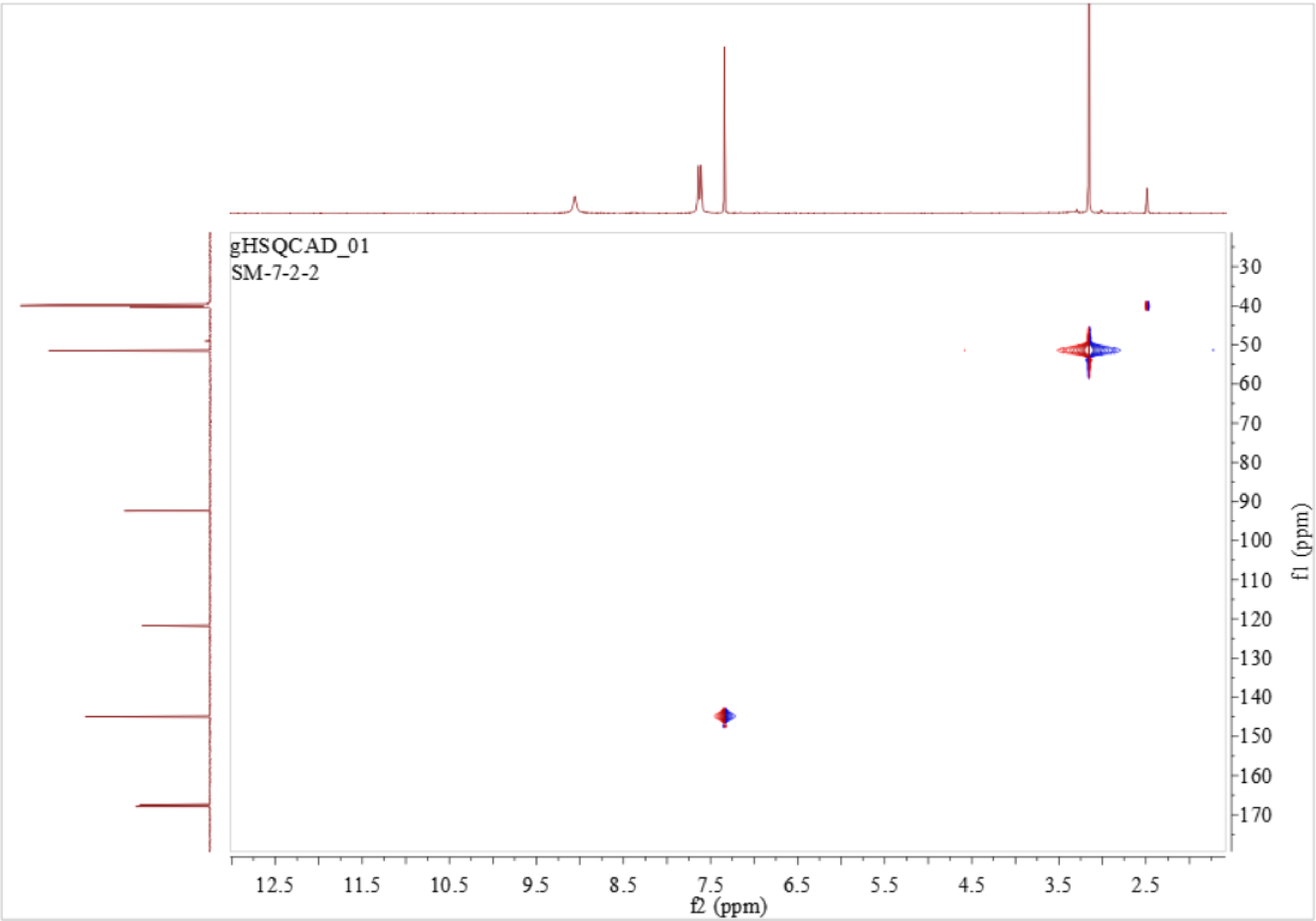

Figure S93. HSQC spectrum of compound **10** in DMSO-d_6 (500 MHz).

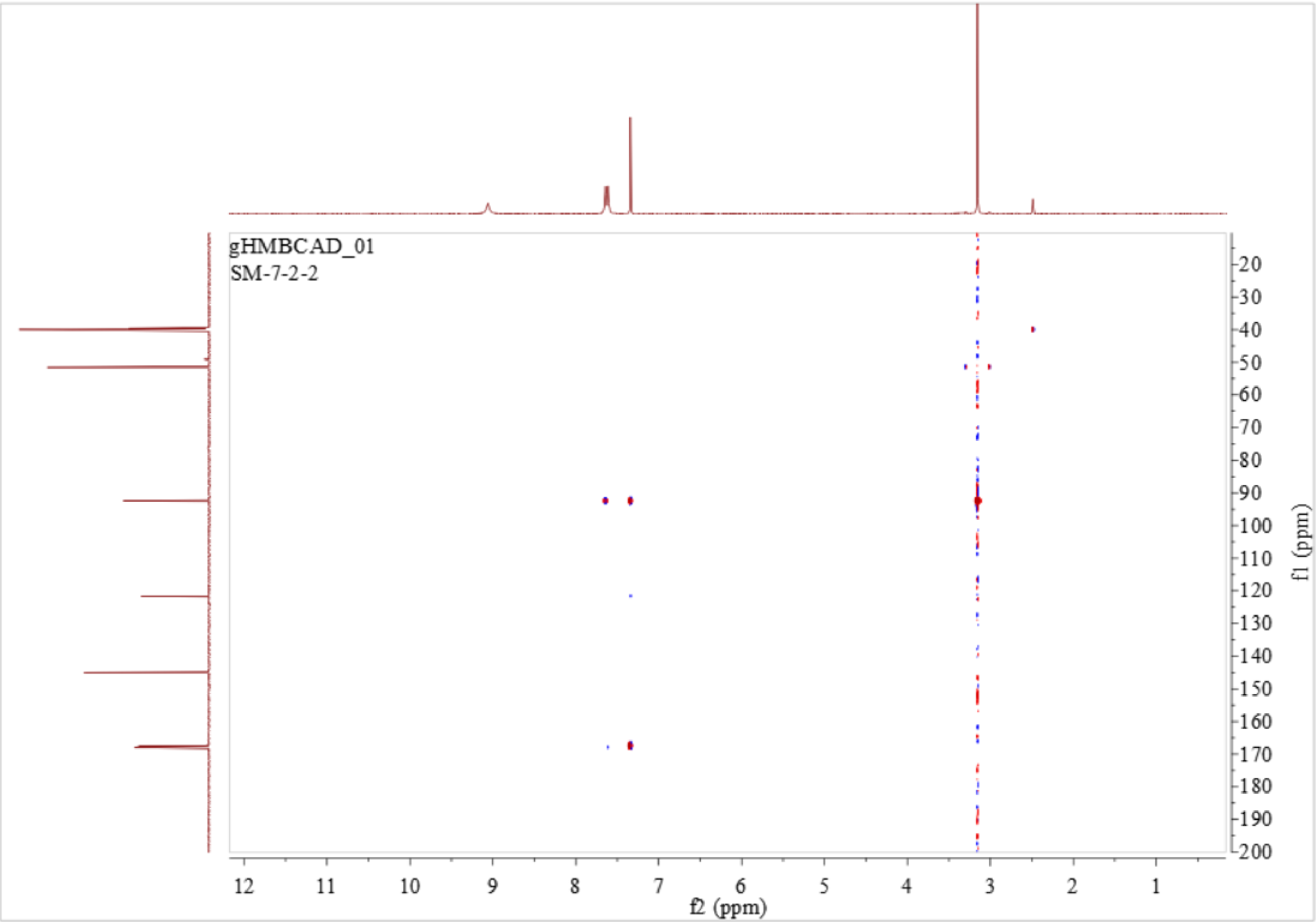

Figure S94. HMBC spectrum of compound **10** in DMSO-d_6 (500 MHz).

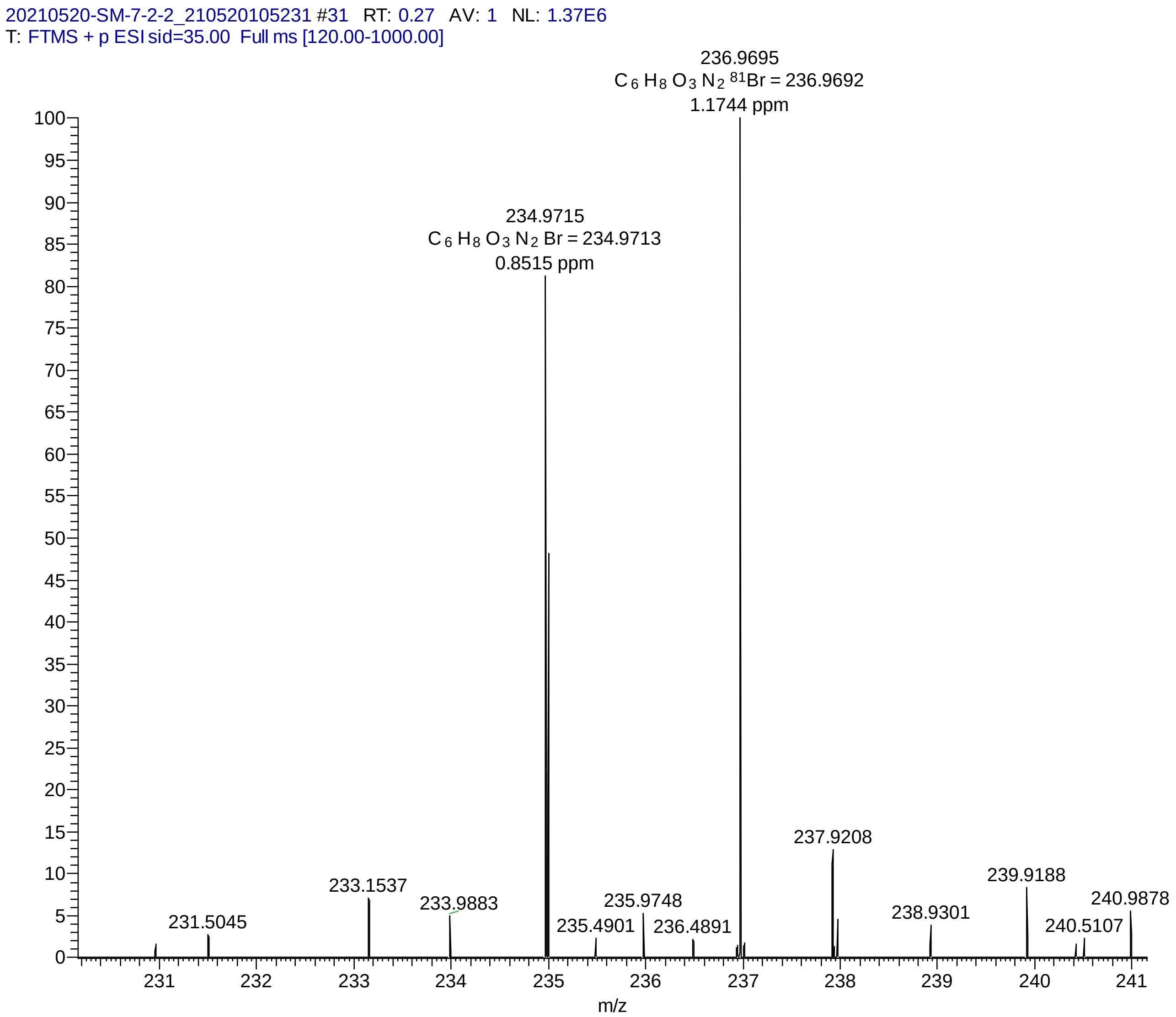

Figure S95. HRESIMS data of compound **10**.

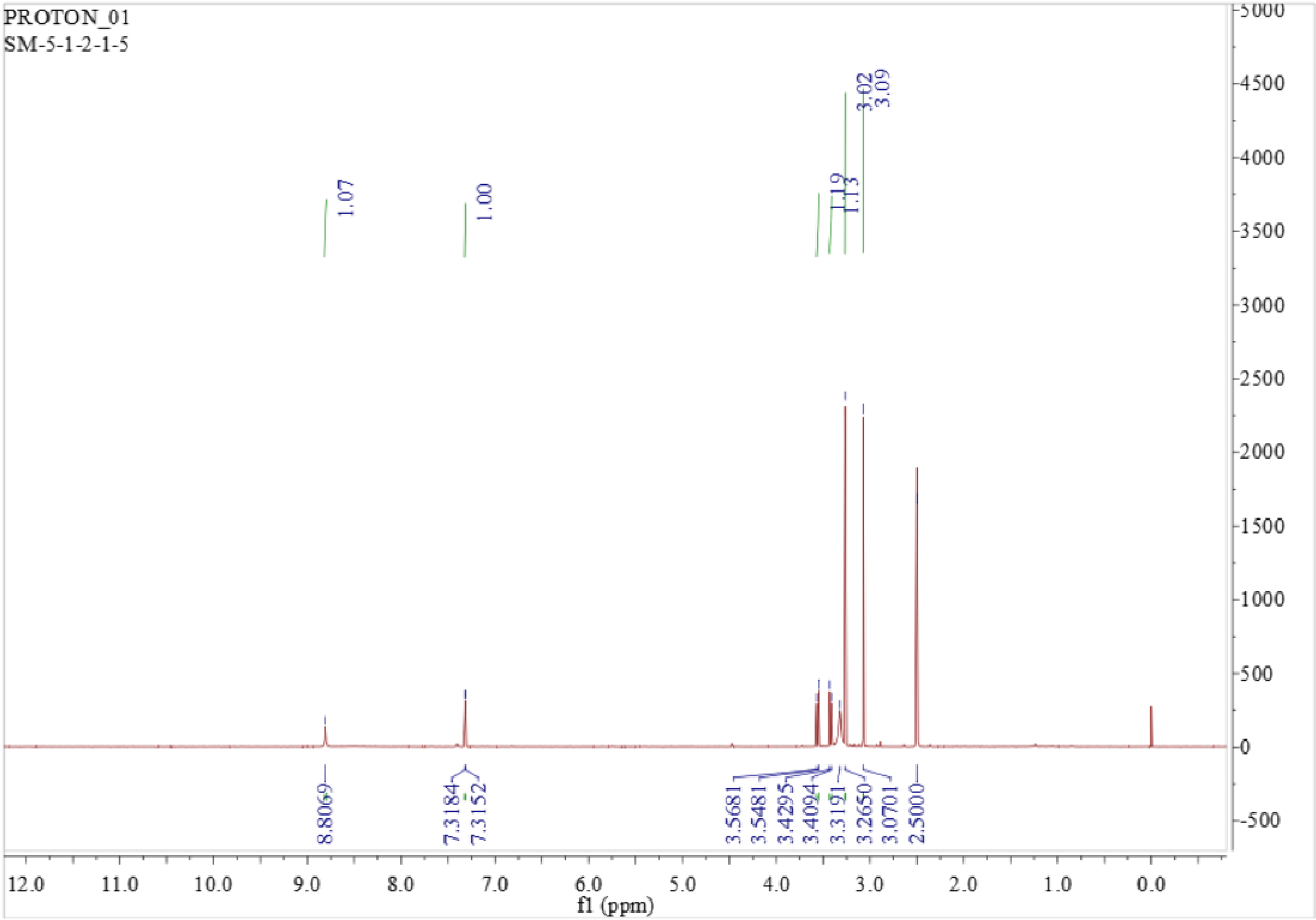

Figure S96. ^{1}H NMR spectrum of compound **11** in DMSO-d_6 (500 MHz).

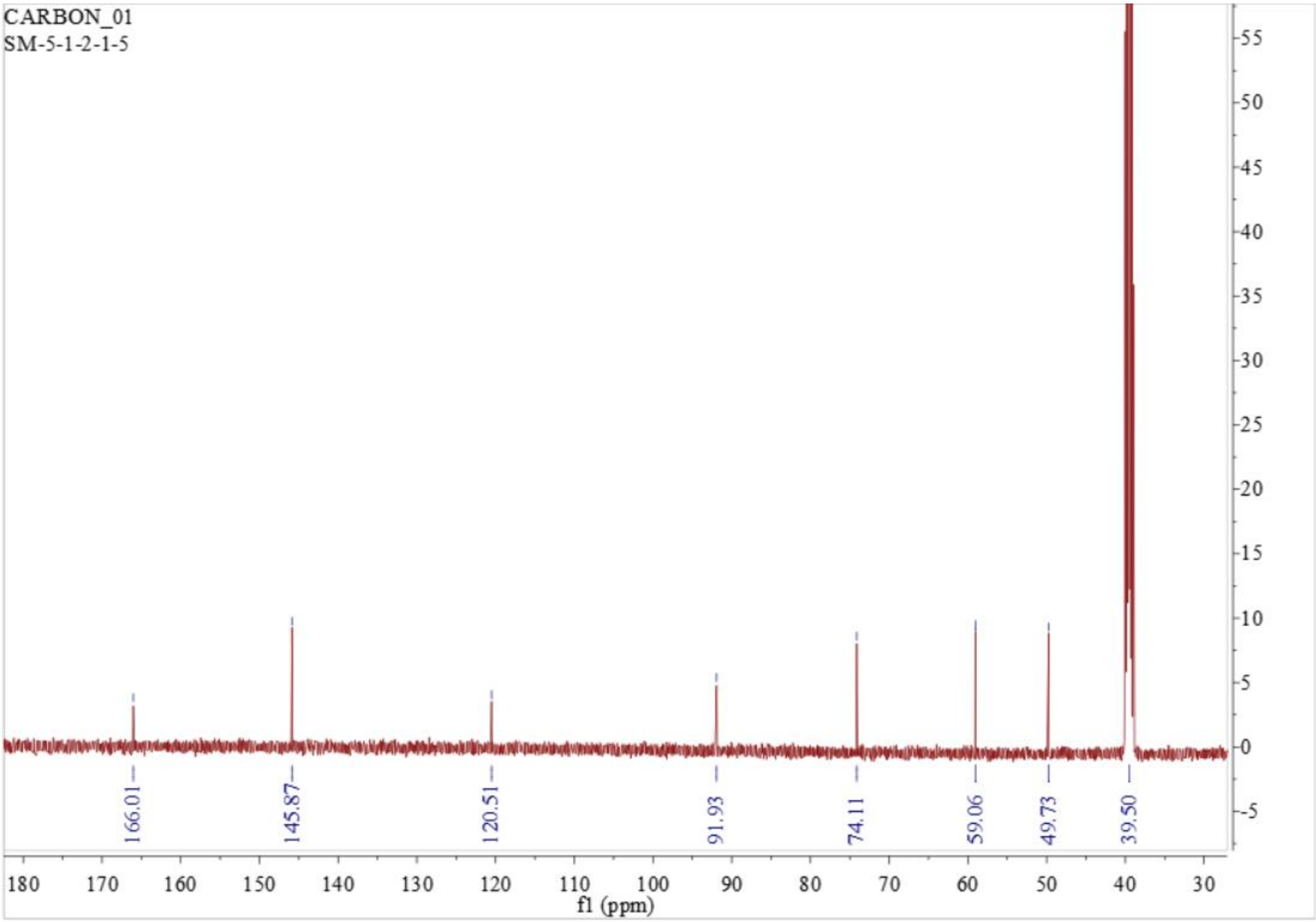

Figure S97. ^{13}C NMR spectrum of compound **11** in DMSO-d_6 (125 MHz).

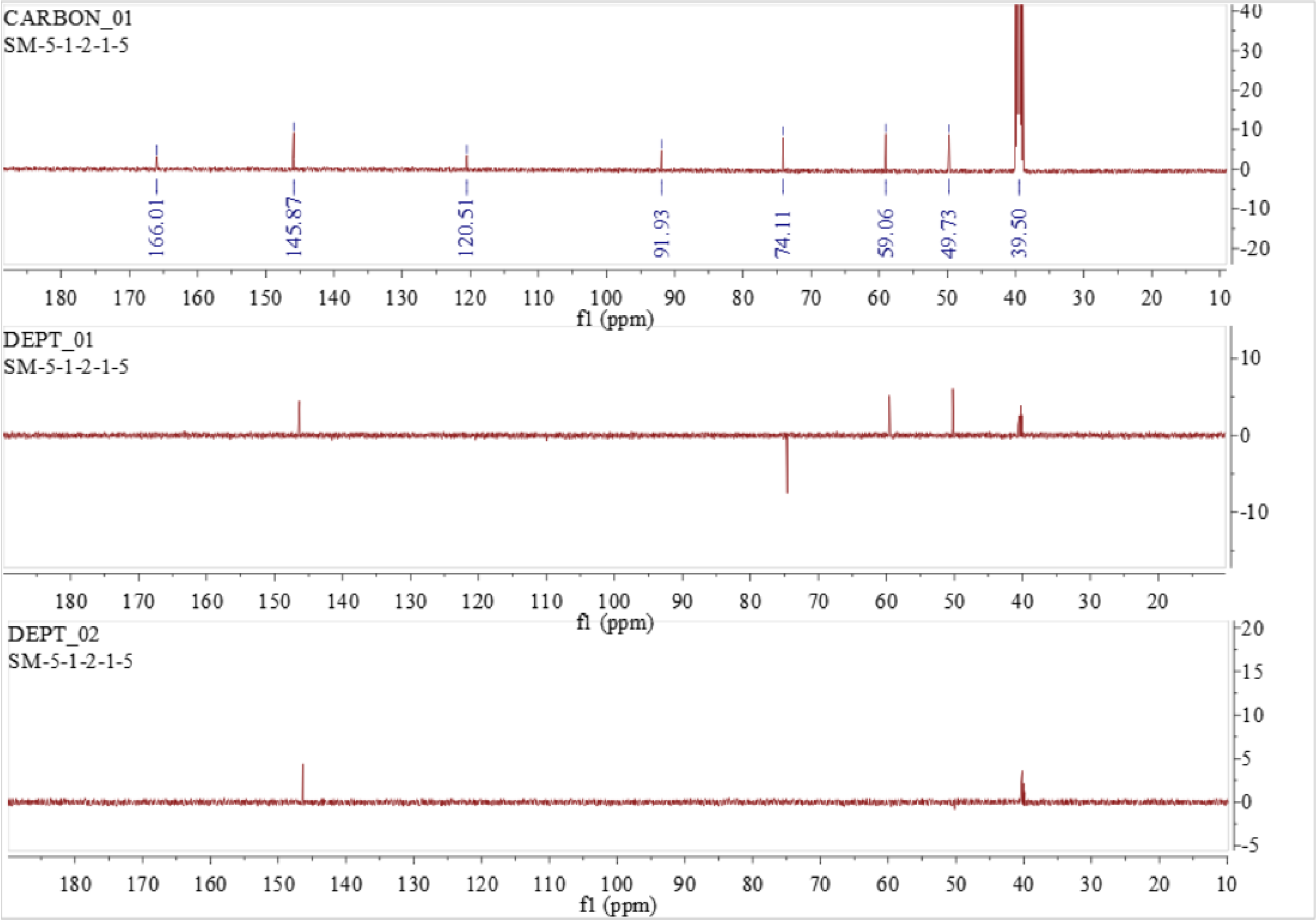

Figure S98. ^{13}C NMR and DEPT spectrum of compound **11** in DMSO-d_6 (125 MHz).

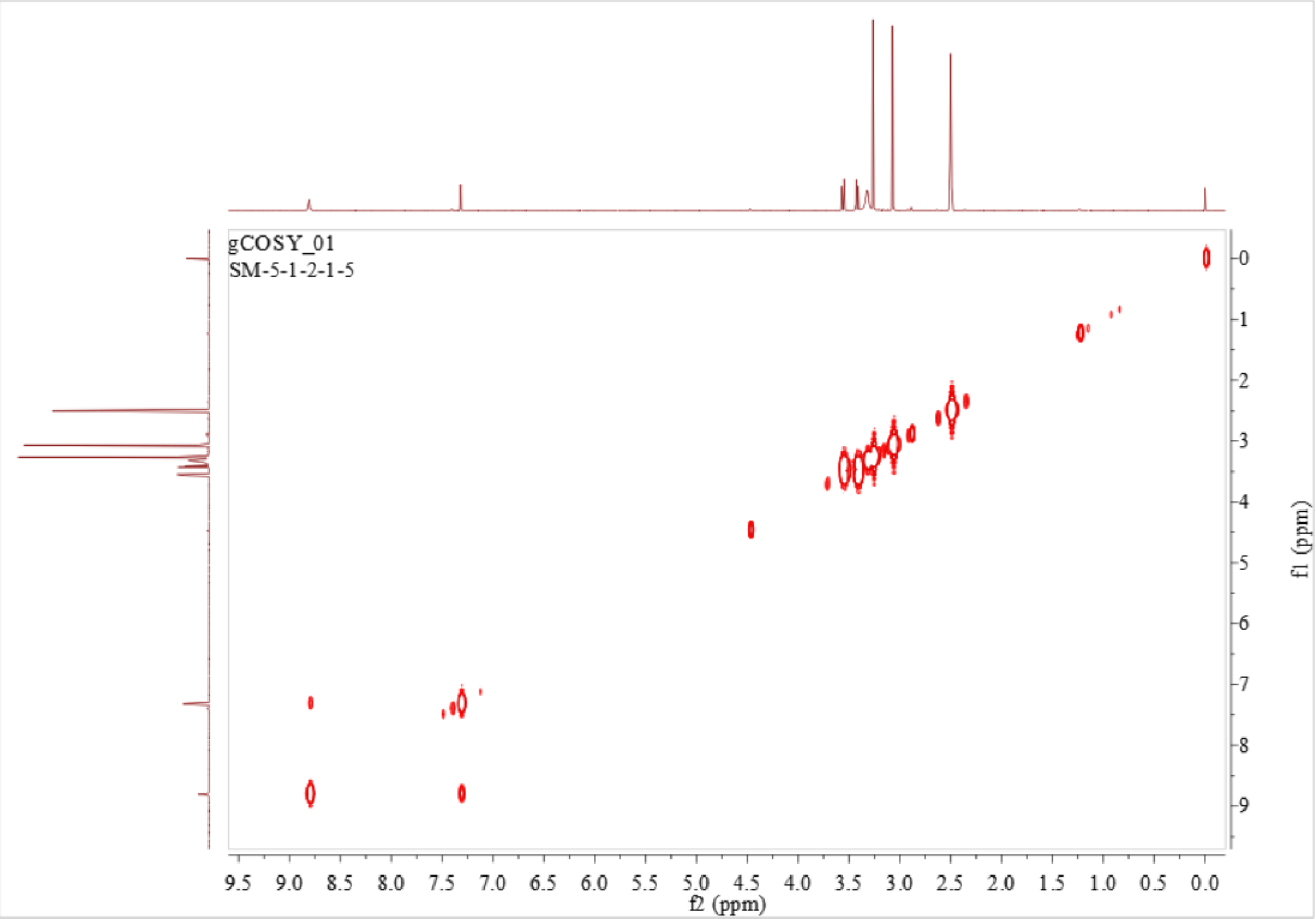

Figure S99. ^1H-^1H COSY spectrum of compound **11** in DMSO-d_6 (500 MHz).

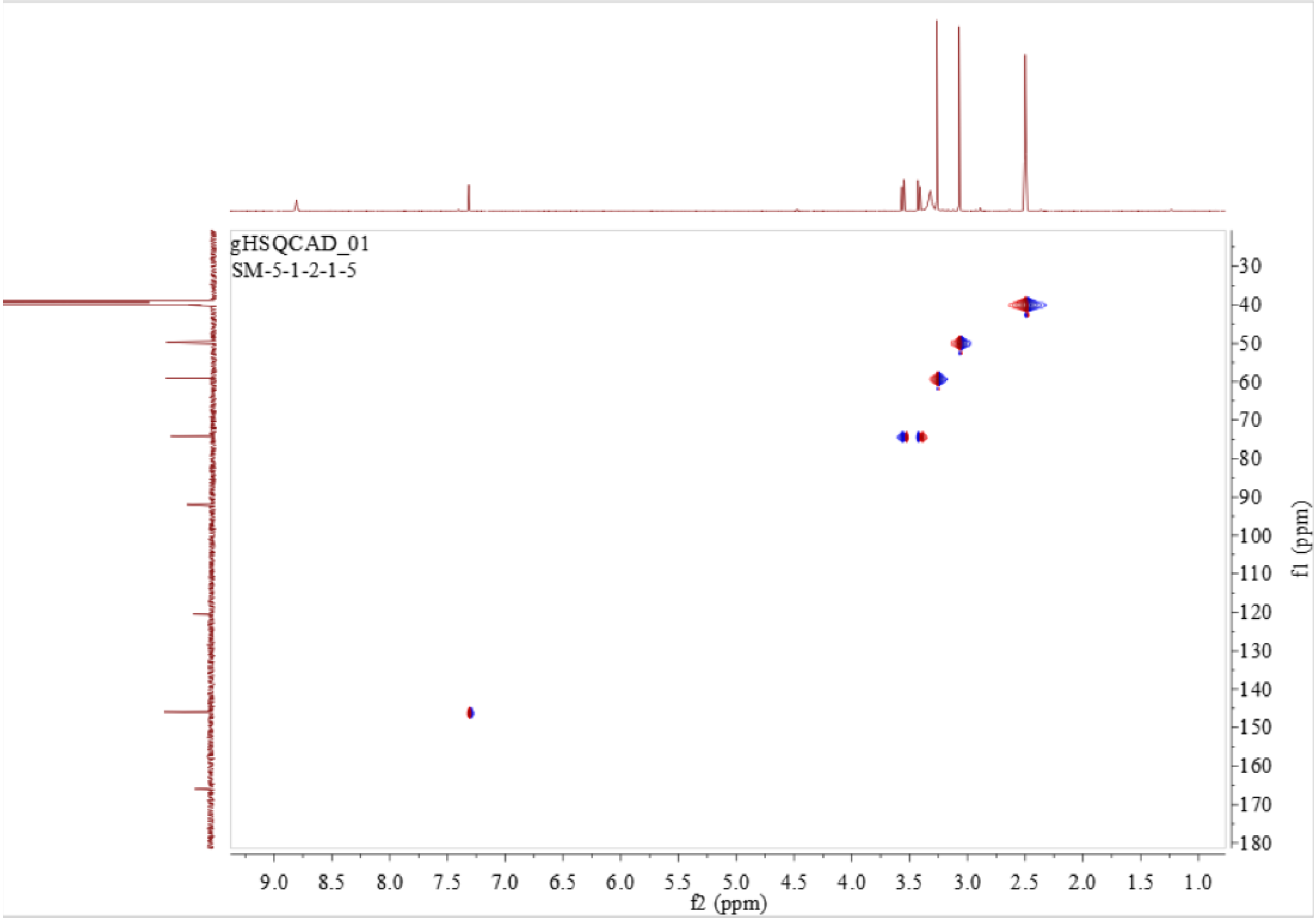

Figure S100. HSQC spectrum of compound **11** in DMSO-d_6 (500 MHz).

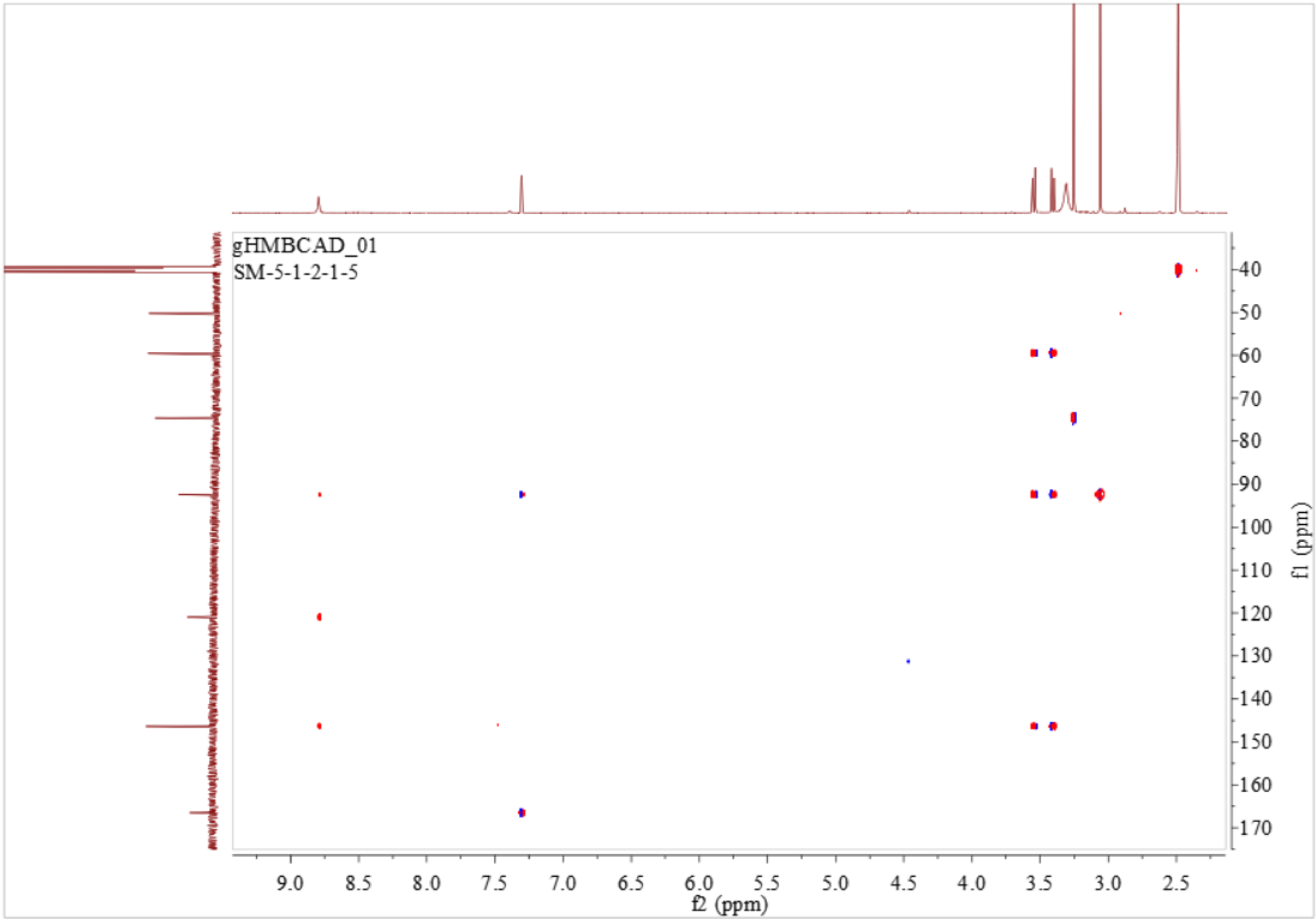

Figure S101. HMBC spectrum of compound **11** in DMSO-d_6 (500 MHz).

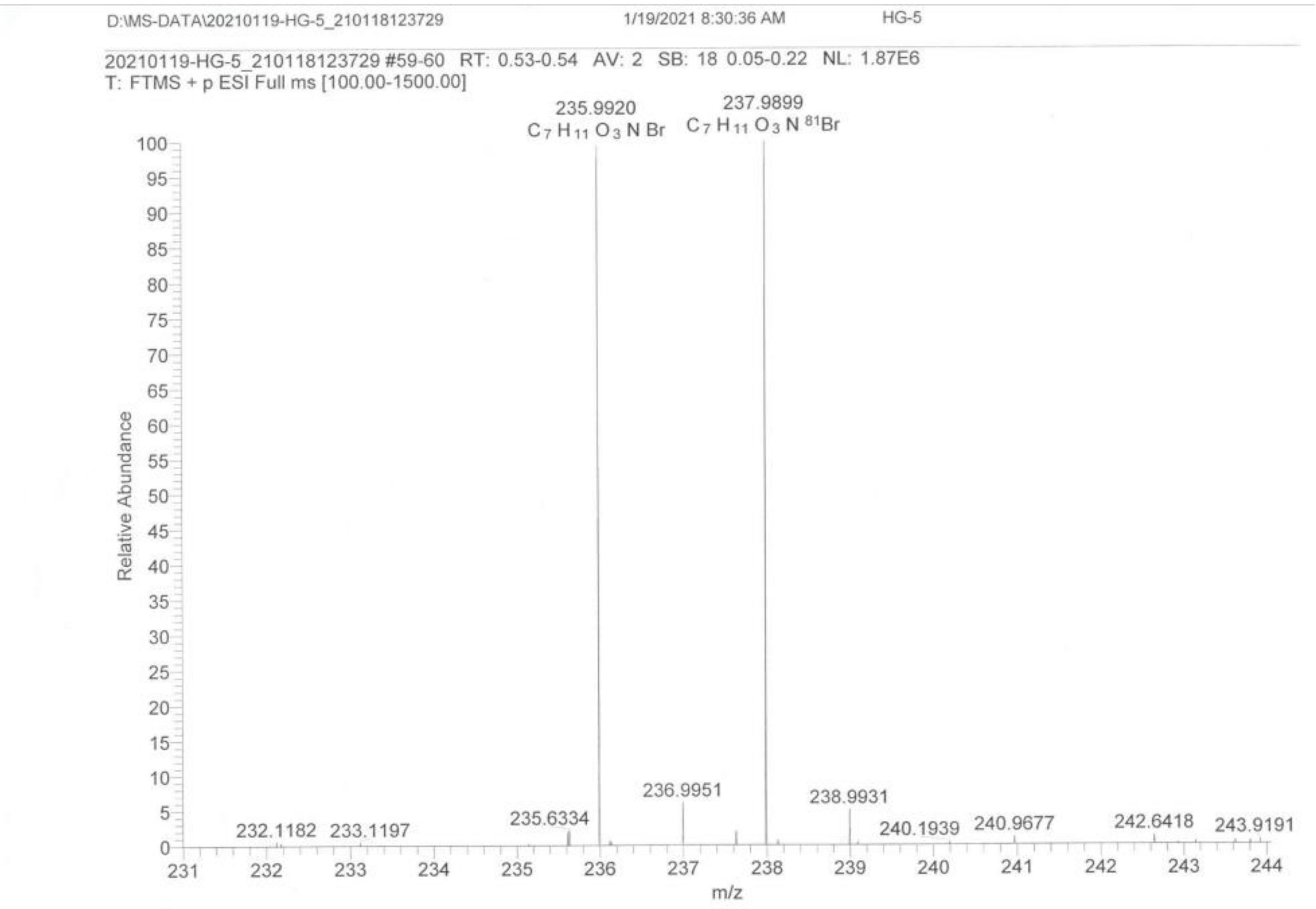

Figure S102. HRESIMS data of compound **11**.

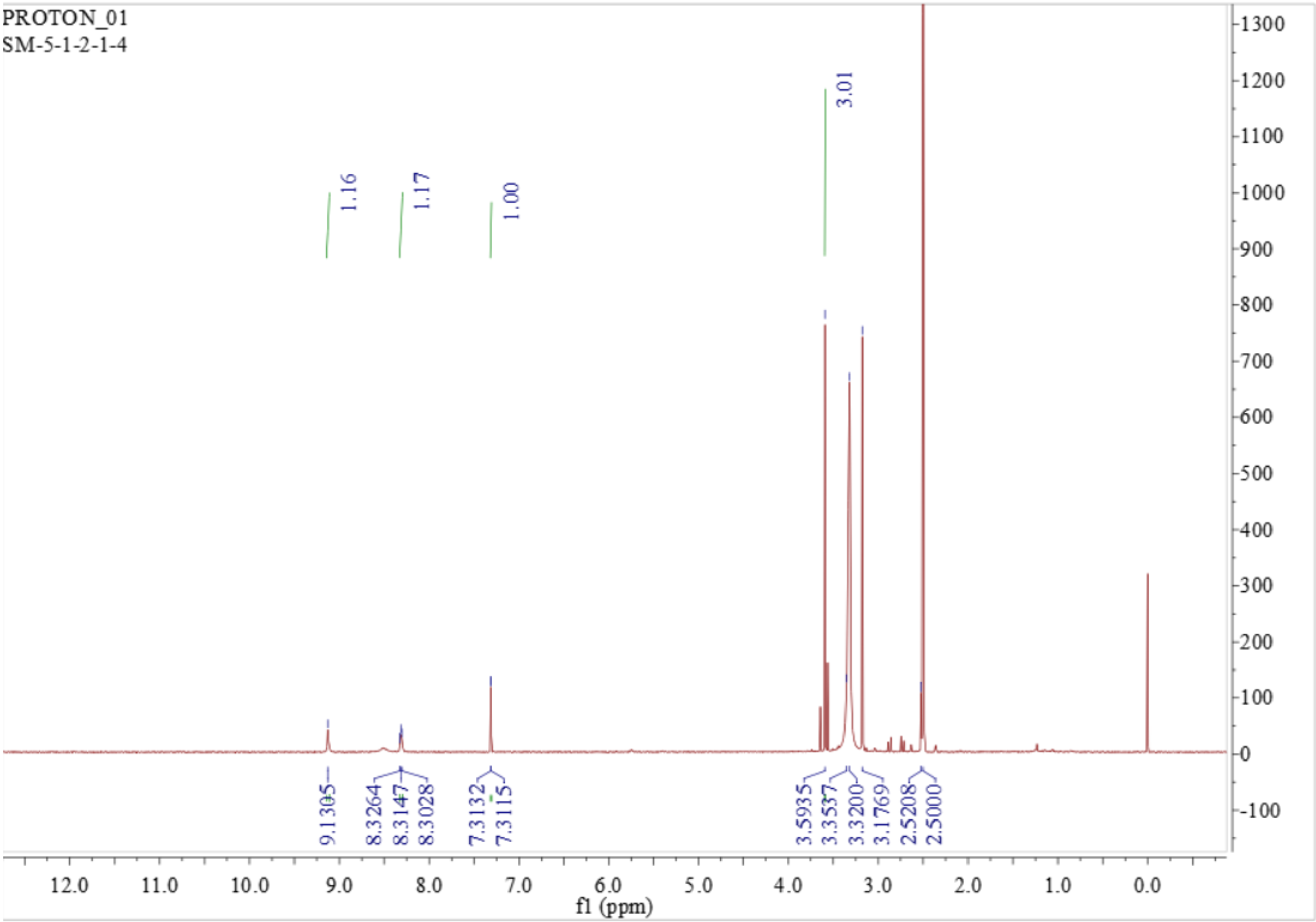

Figure S103. ^{1}H NMR spectrum of compound **12** in DMSO-d_6 (500 MHz).

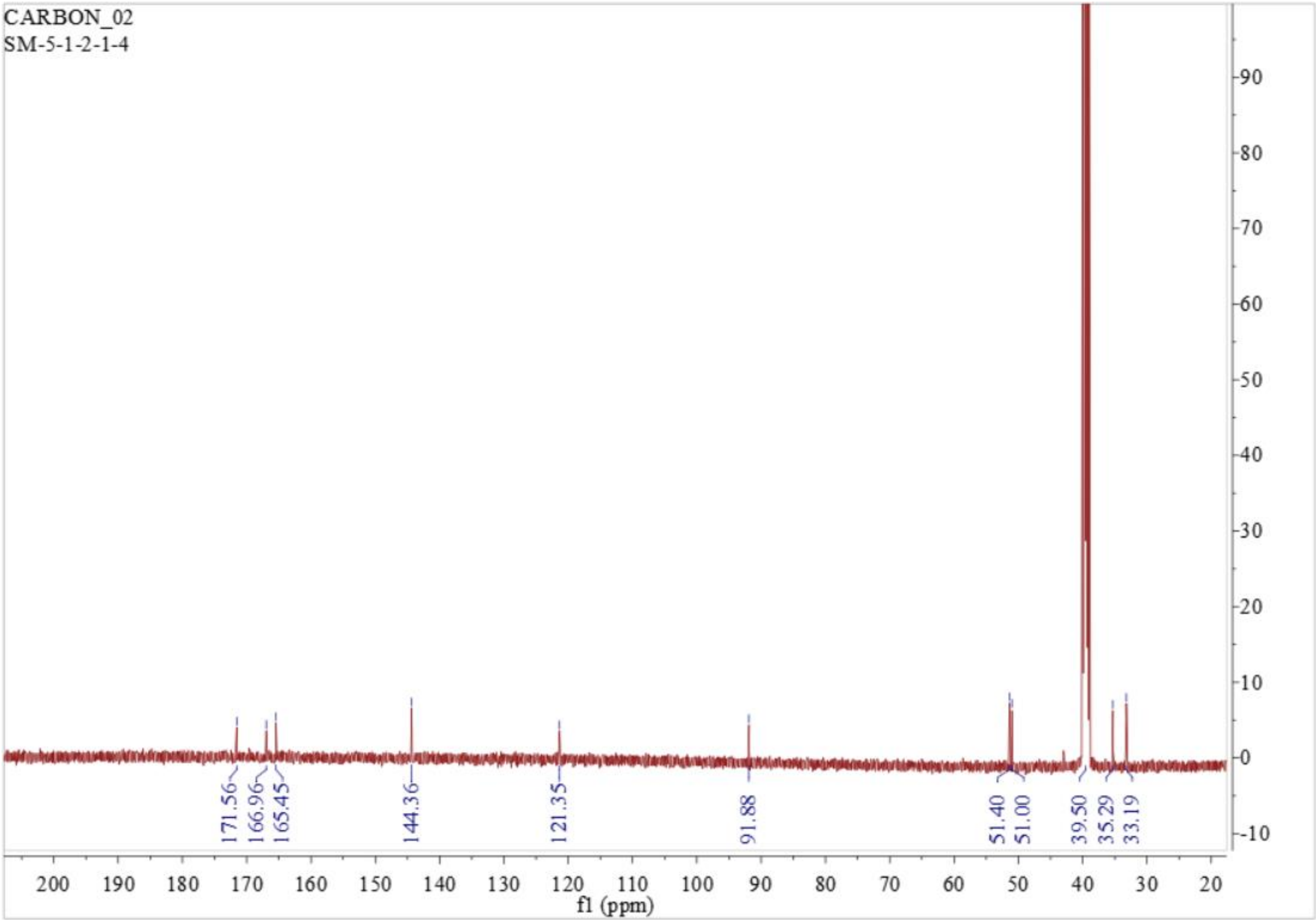

Figure S104. ^{13}C NMR spectrum of compound **12** in DMSO-d_6 (125 MHz).

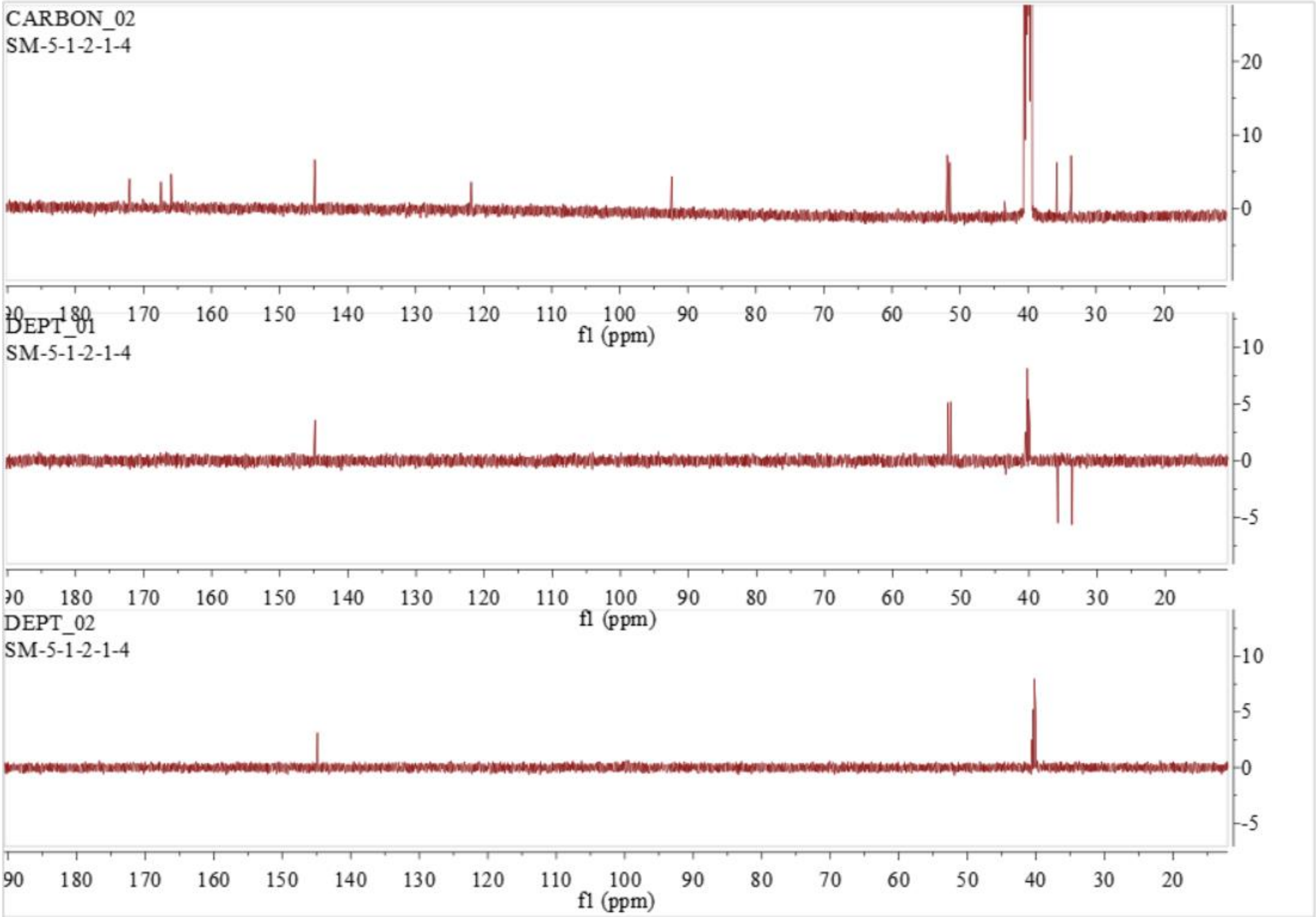

Figure S105. ^{13}C NMR and DEPT spectrum of compound **12** in DMSO-d_6 (125 MHz).

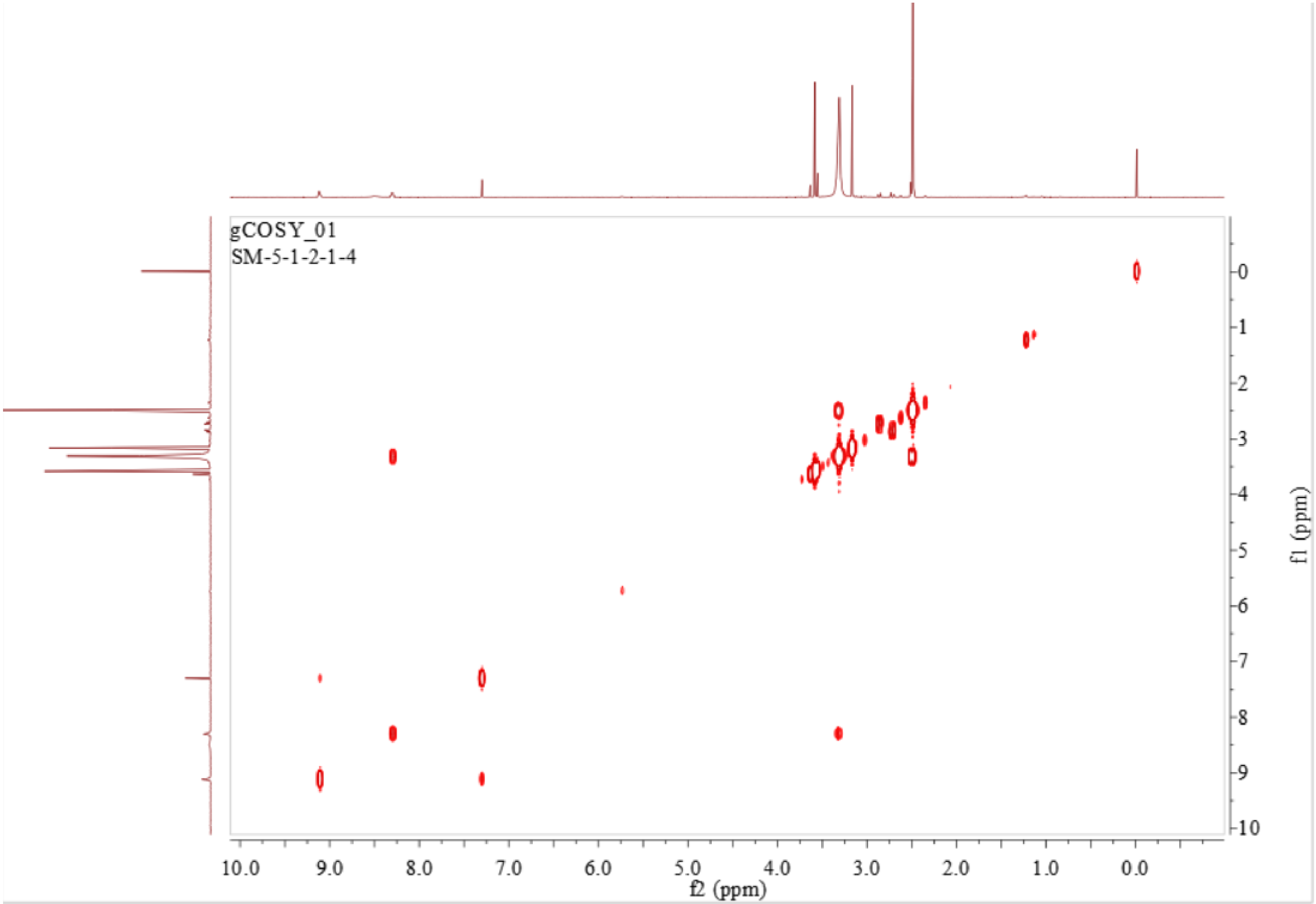

Figure S106. ^{1}H-^{1}H COSY spectrum of compound **12** in DMSO-d_6 (500 MHz).

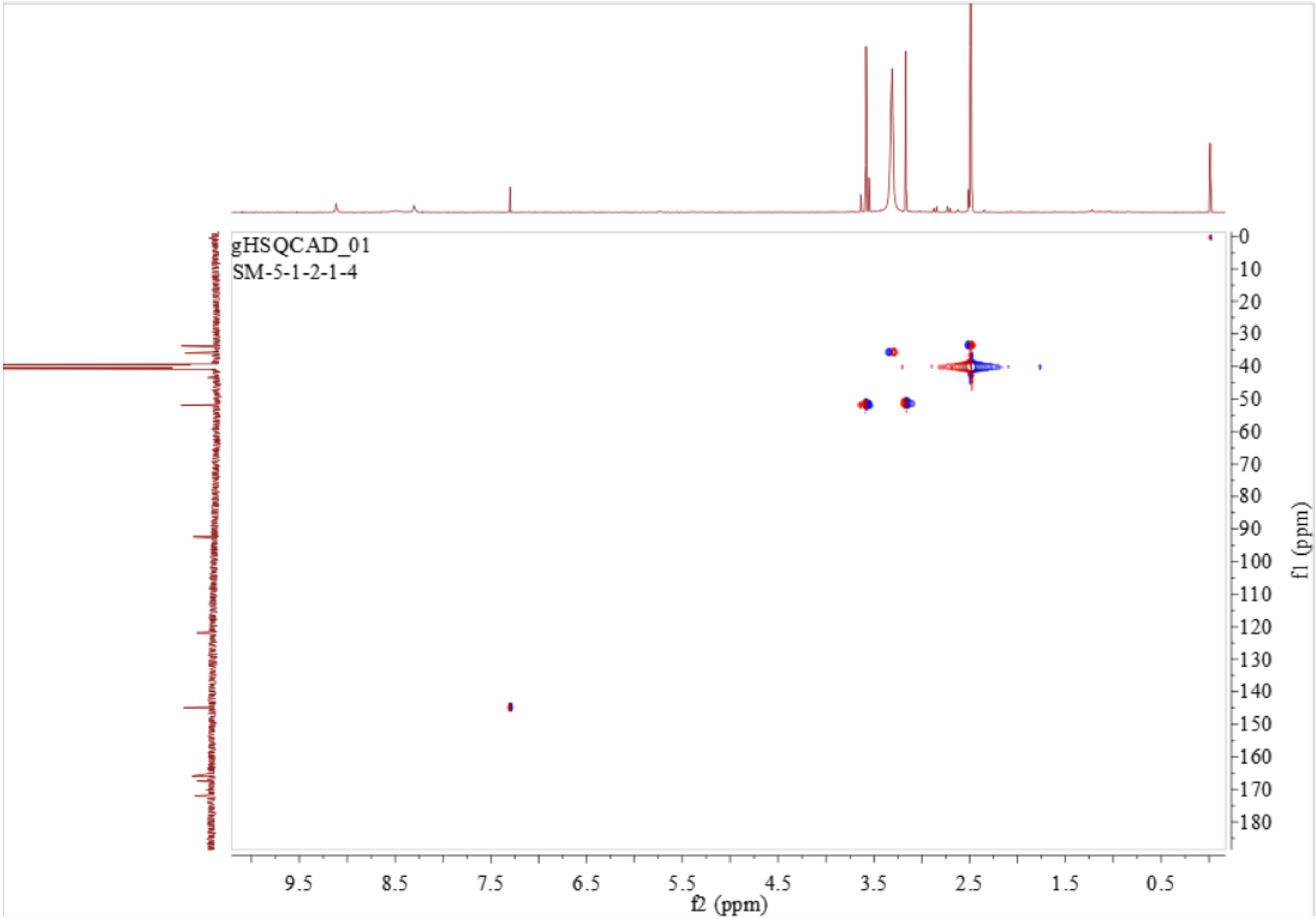

Figure S107. HSQC spectrum of compound **12** in DMSO-d_6 (500 MHz).

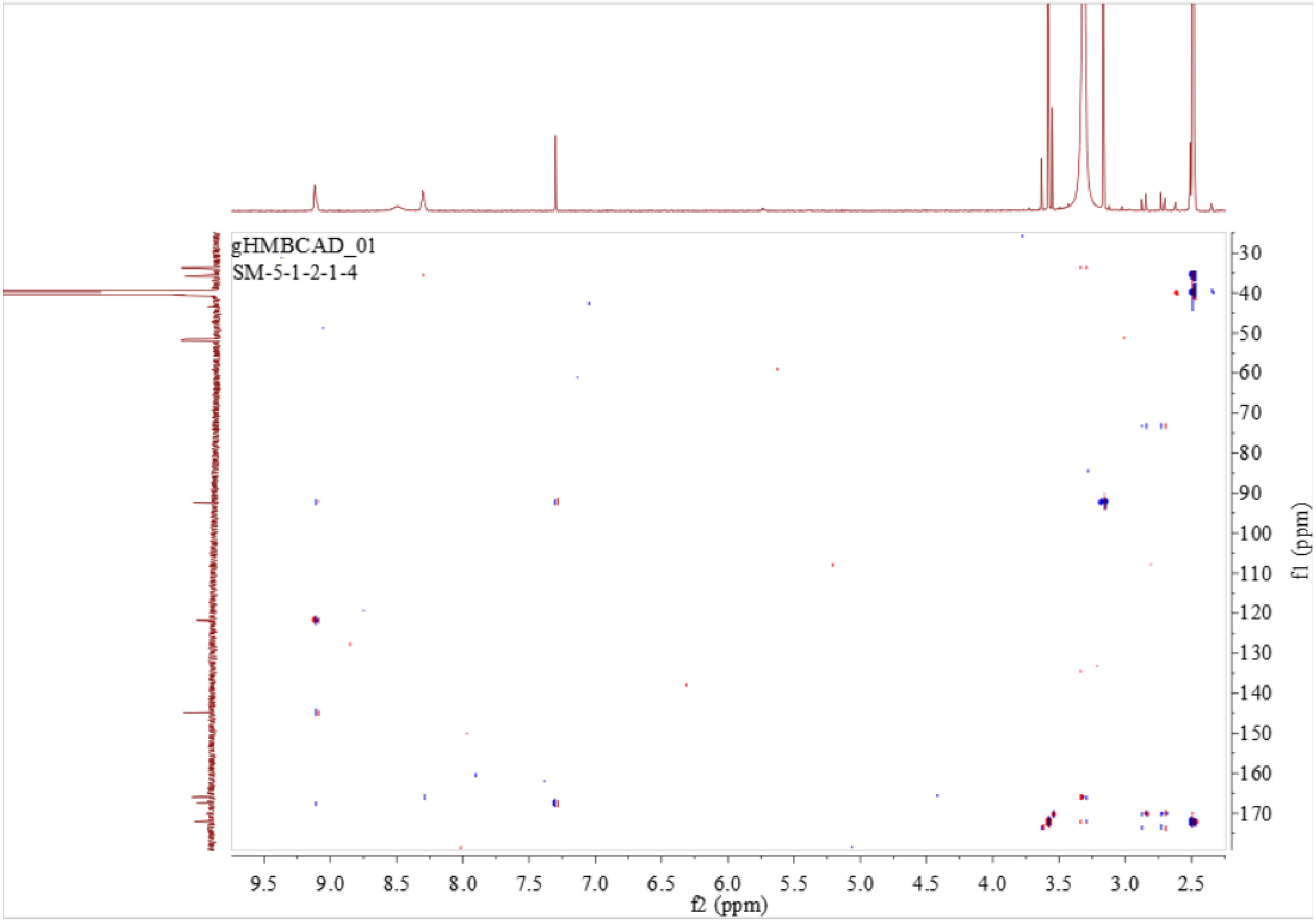

Figure S108. HMBC spectrum of compound **12** in DMSO-d_6 (500 MHz).

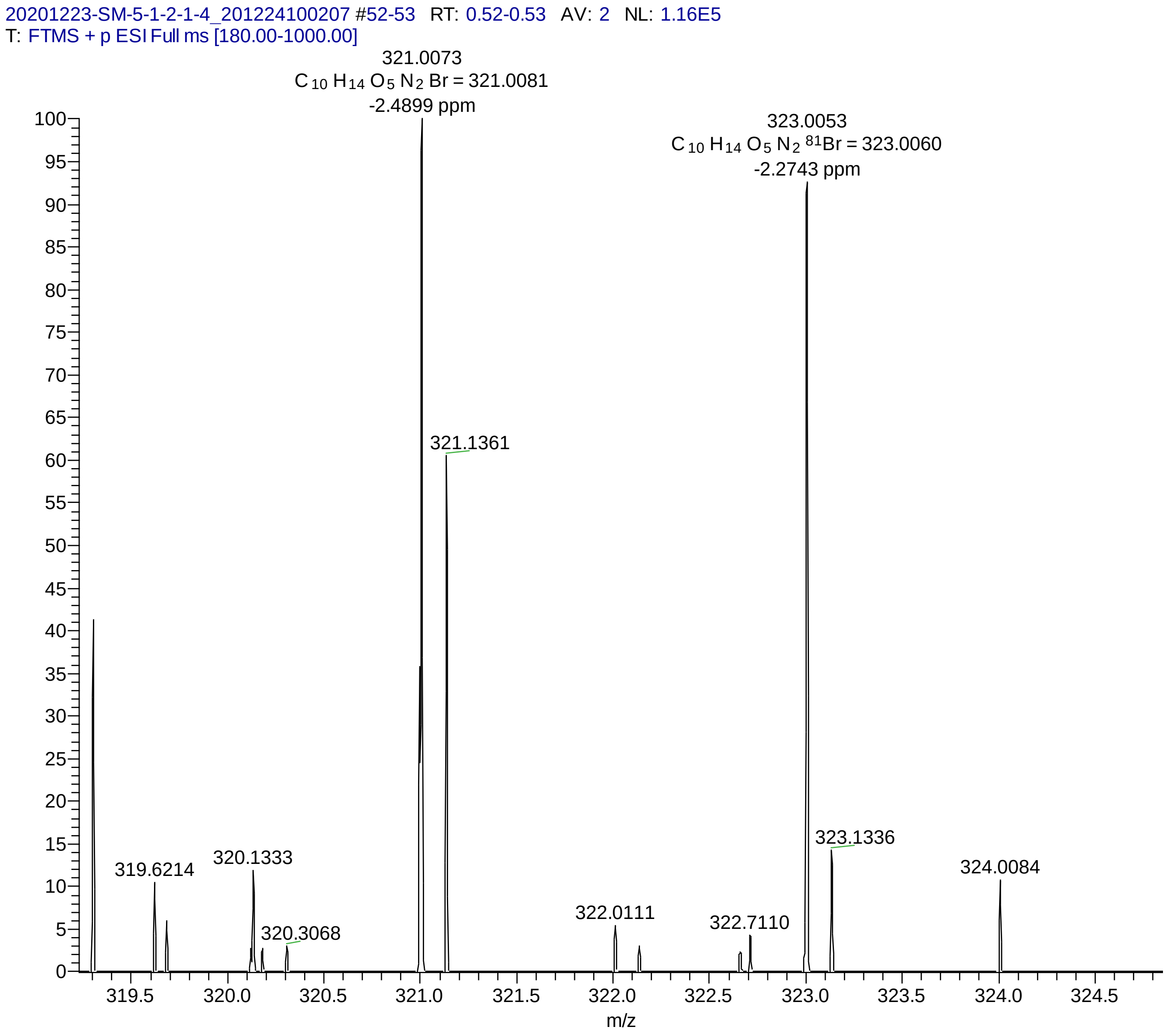

Figure S109. HRESIMS data of compound **12**.

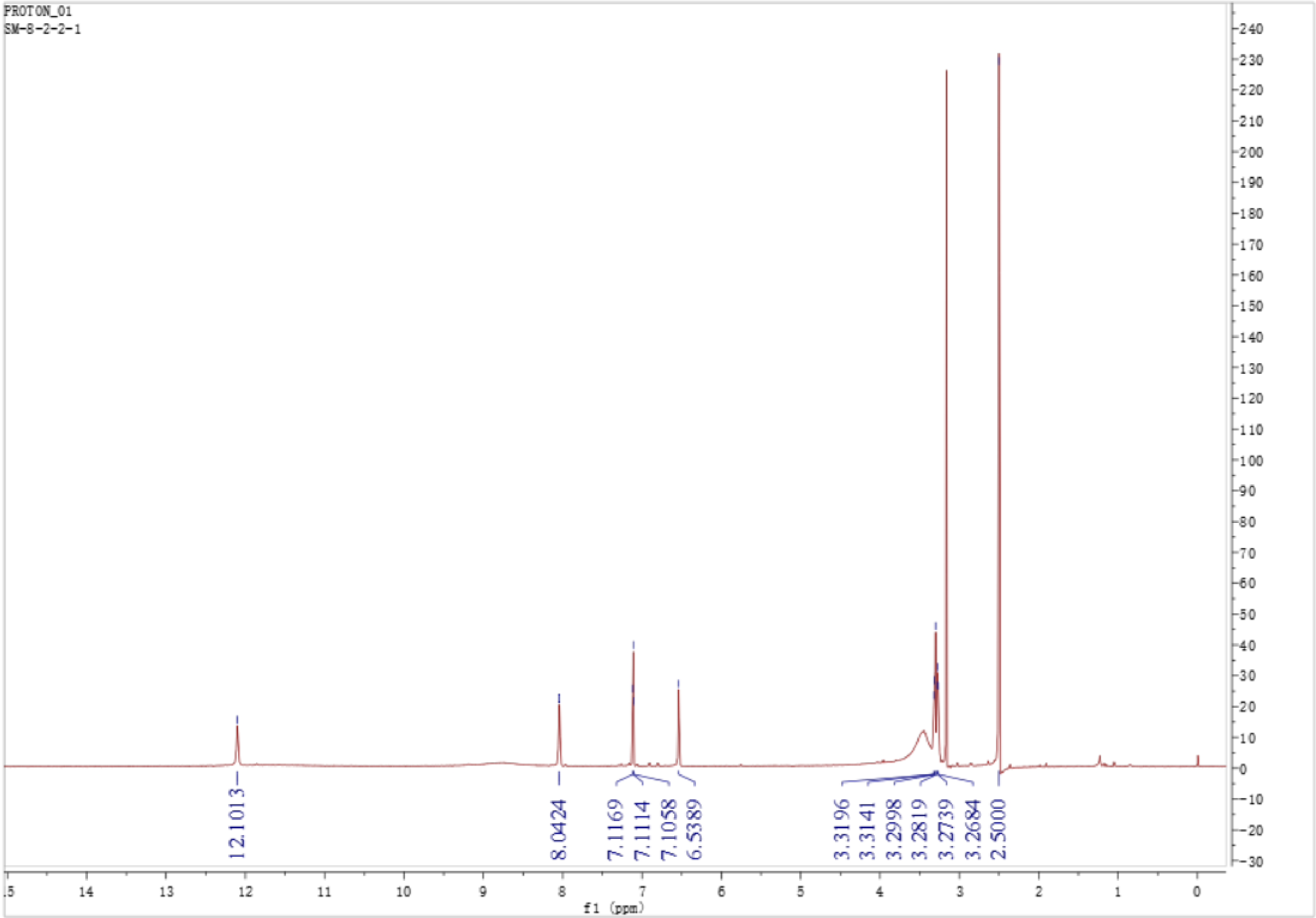

Figure S110. ^{1}H NMR spectrum of compound **13** in DMSO-d_6 (500 MHz).

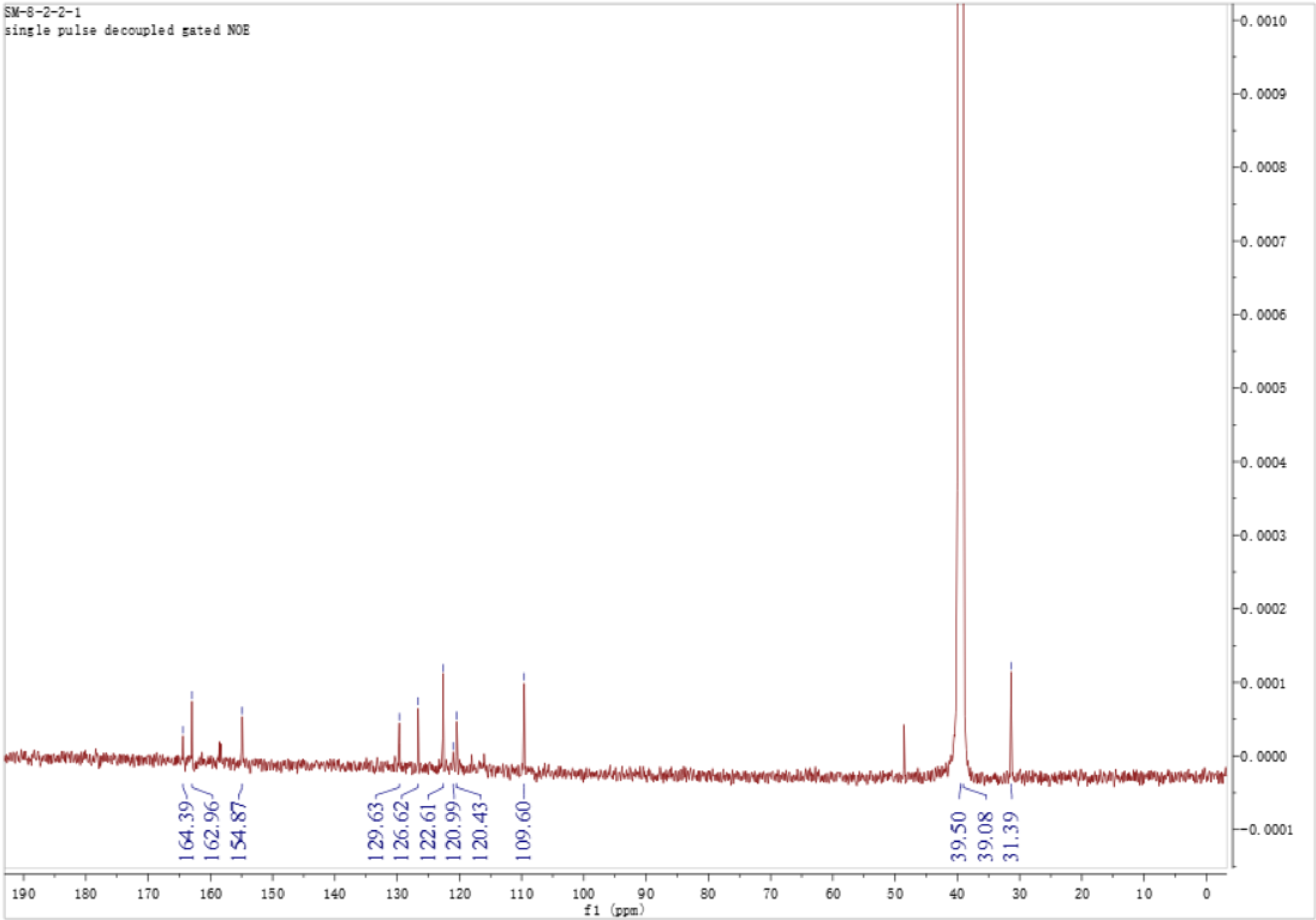

Figure S111. ^{13}C NMR spectrum of compound **13** in DMSO-d_6 (125 MHz).

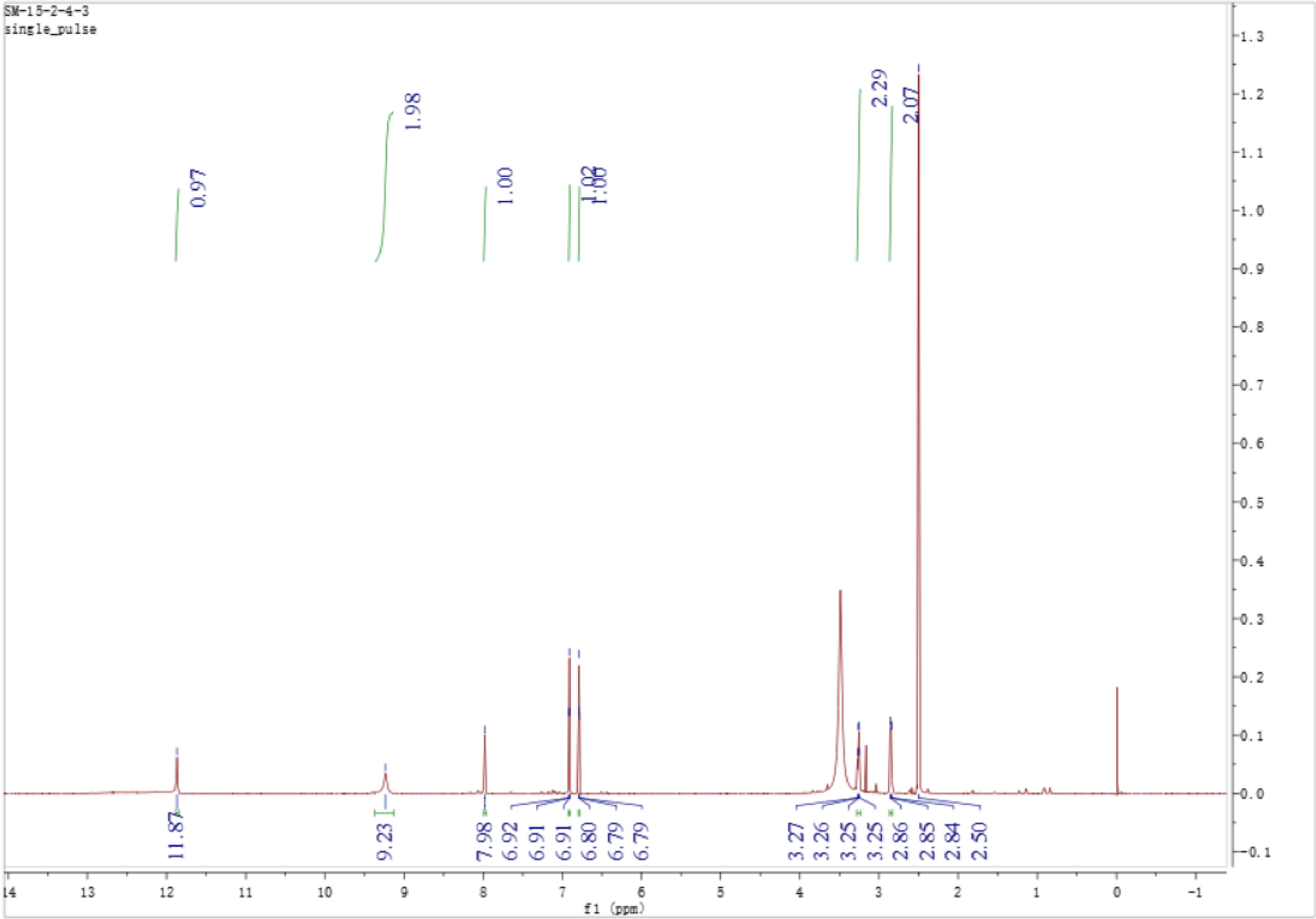

Figure S112. ^{1}H NMR spectrum of compound **14** in DMSO-d_6 (500 MHz).

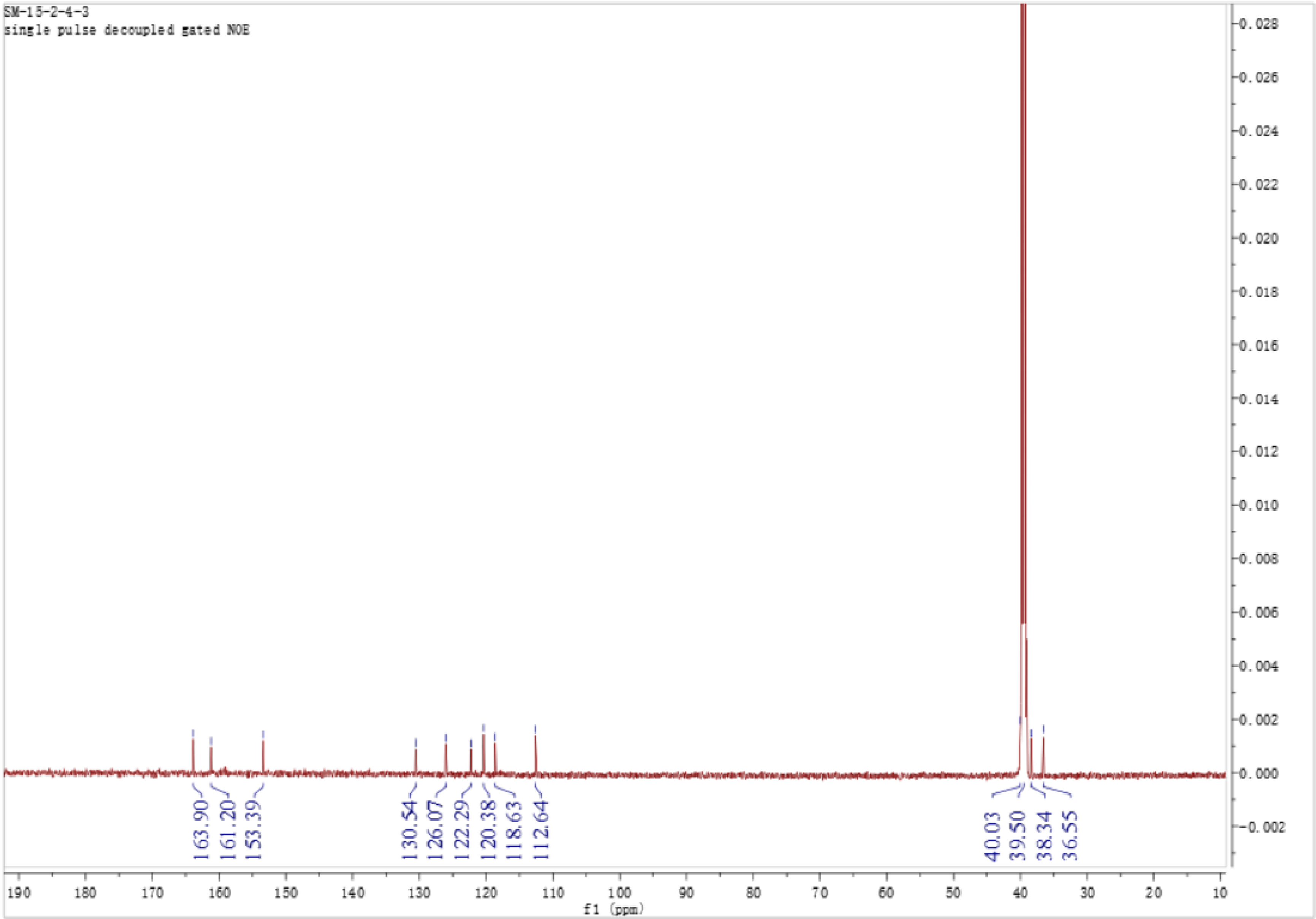

Figure S113. ^{13}C NMR spectrum of compound **14** in DMSO-d_6 (125 MHz).

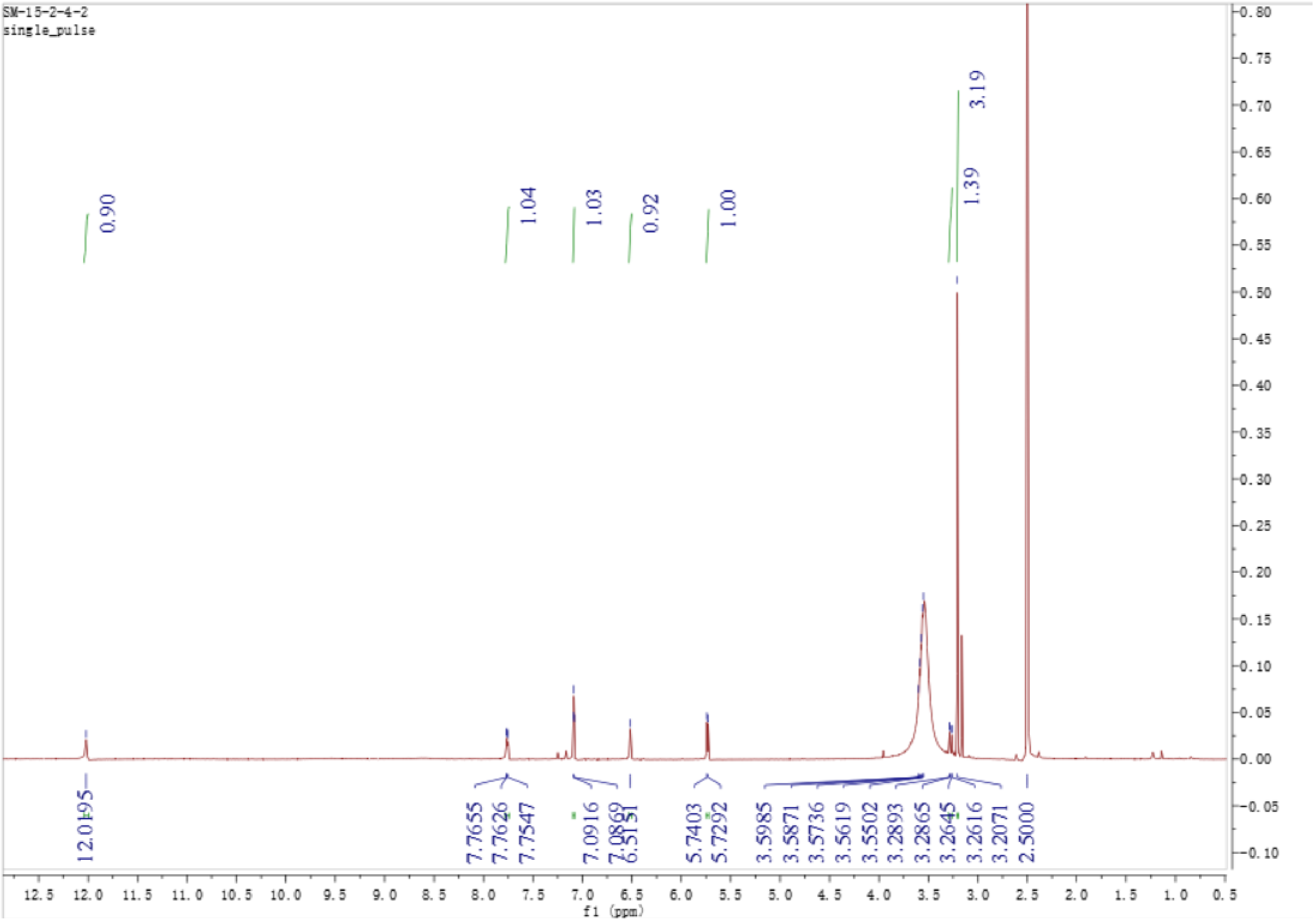

Figure S114. ^{1}H NMR spectrum of compound **15** in DMSO-d_6 (500 MHz).

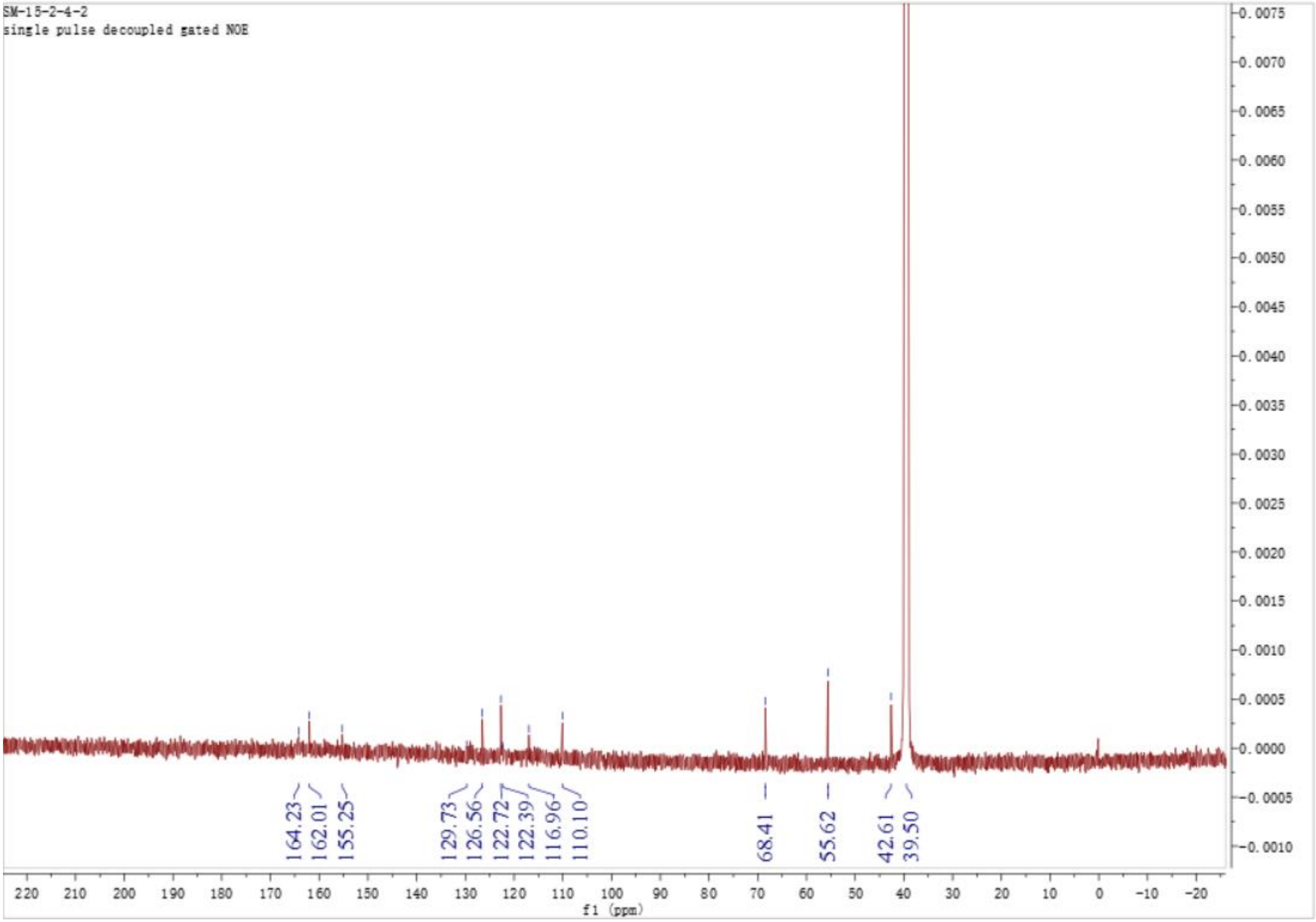

Figure S115. ^{13}C NMR spectrum of compound **15** in DMSO-d_6 (125 MHz).

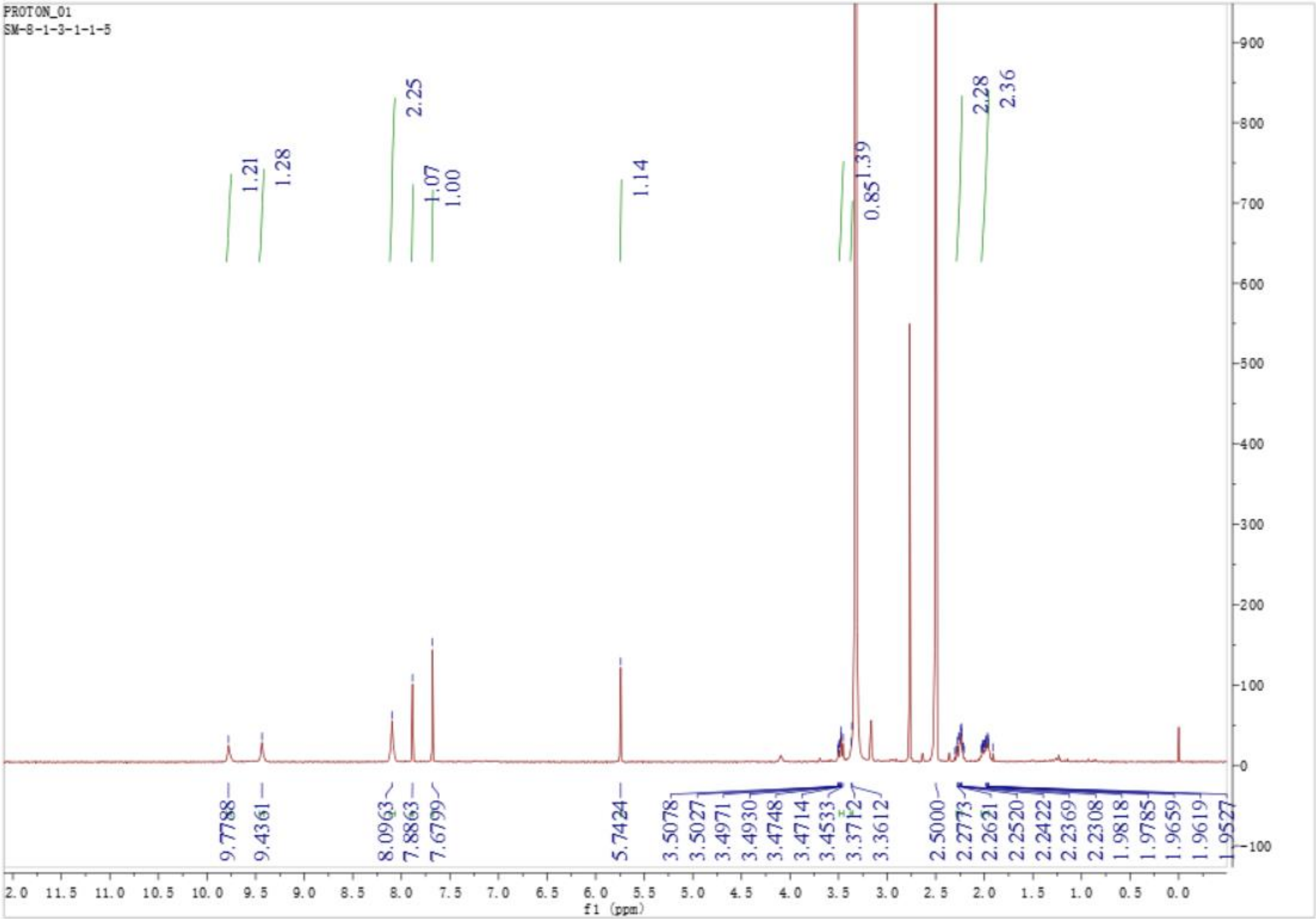

Figure S116. ^{1}H NMR spectrum of compound **16** in DMSO-d_6 (500 MHz).

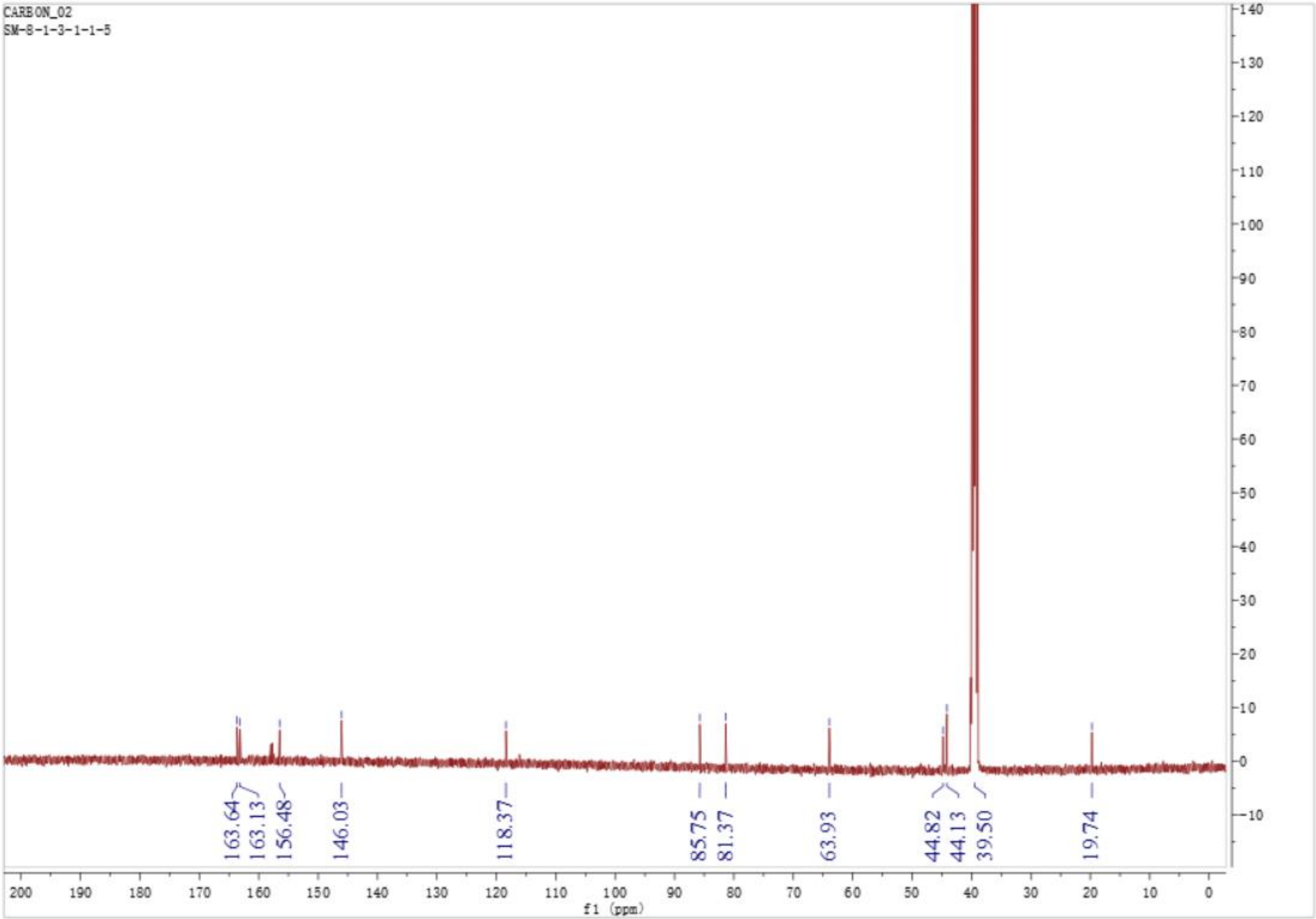

Figure S117. ^{13}C NMR spectrum of compound **16** in DMSO-d_6 (125 MHz).
