# Supplementary figures and images for: A Series of New Pyrrole Alkaloids with ALR2 Inhibitory Activities from the Sponge Stylissa massa
Source: Mar Drugs. 2022 Jul 12;20(7):454. doi: 10.3390/md20070454 (PMC9320028; doi:10.3390/md20070454)

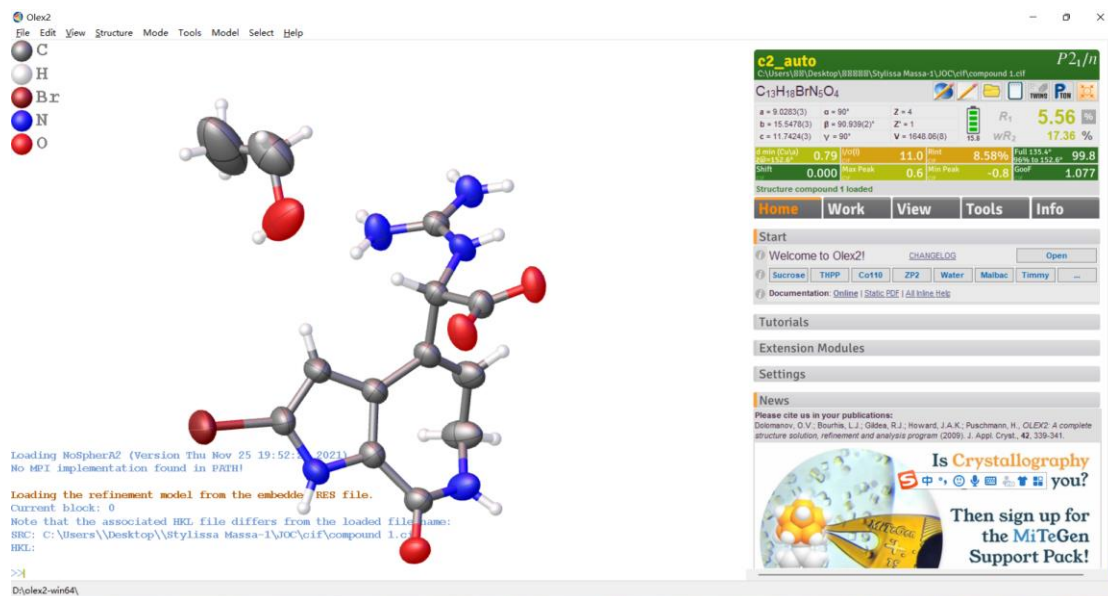

compound 1

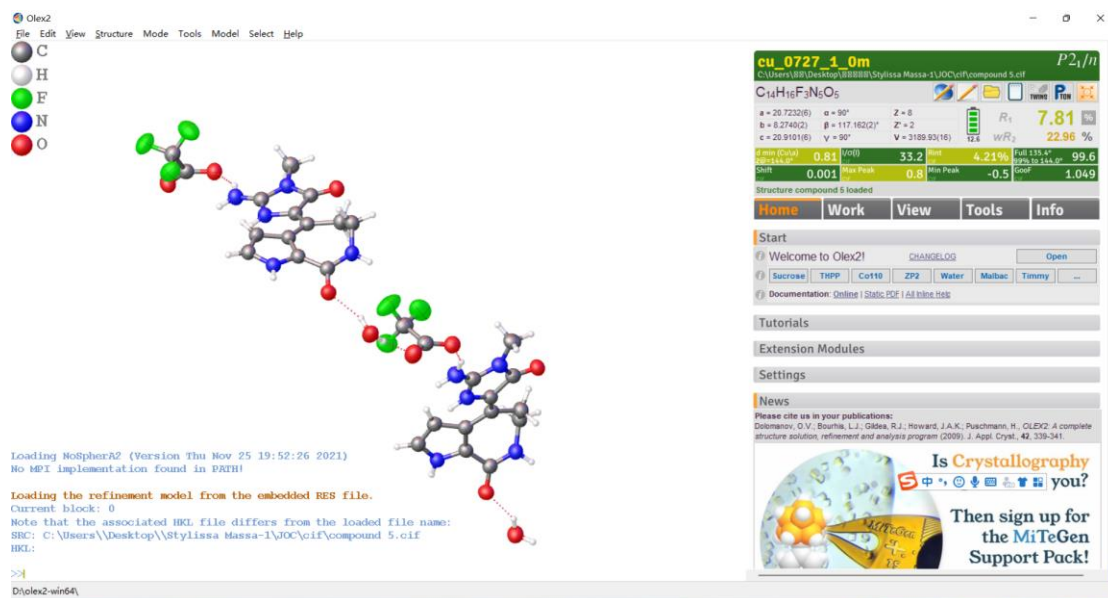

compound 5

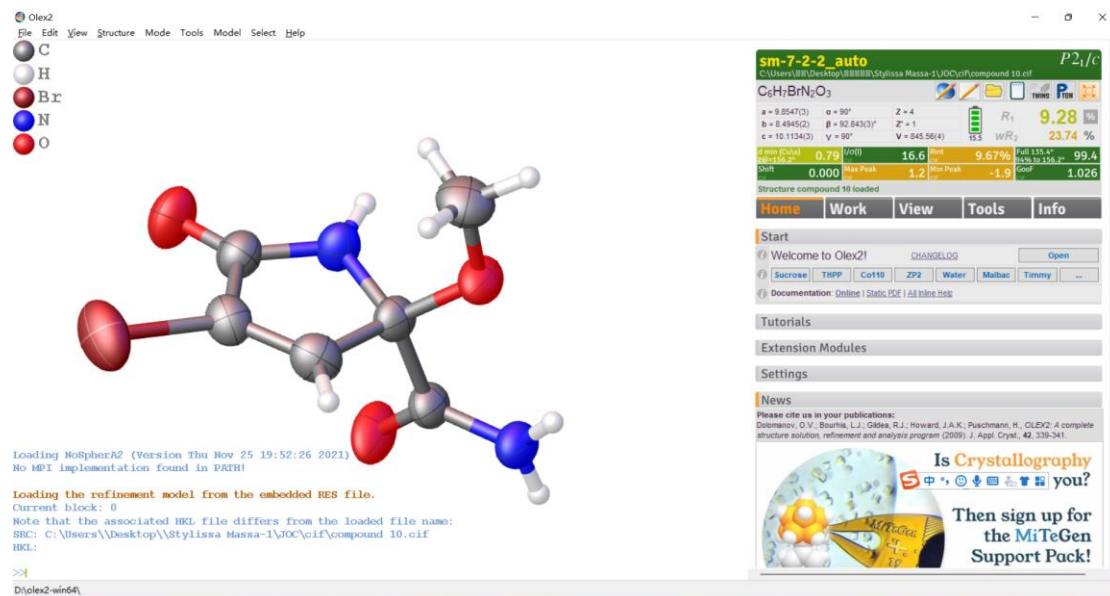

compound 10

Supplement: Supplementary file 1 [file marinedrugs-20-00454-s001.zip › mrinedrugs-1802470 Supplementary Material-X ray data.pdf]
